# Bio-Catalytic Structural Transformation of Anti-cancer Steroid, Drostanolone Enanthate with *Cephalosporium aphidicola* and *Fusarium lini*, and Cytotoxic Potential Evaluation of Its Metabolites against Certain Cancer Cell Lines

**DOI:** 10.3389/fphar.2017.00900

**Published:** 2017-12-20

**Authors:** M. Iqbal Choudhary, Mahwish Siddiqui, Atia-tul-Wahab, Sammer Yousuf, Narjis Fatima, Malik S. Ahmad, Hani Choudhry

**Affiliations:** ^1^H. E. J. Research Institute of Chemistry, International Center for Chemical and Biological Sciences, University of Karachi, Karachi, Pakistan; ^2^Dr. Panjwani Center for Molecular Medicine and Drug Research, International Center for Chemical and Biological Sciences, University of Karachi, Karachi, Pakistan; ^3^Department of Biochemistry, Faculty of Science, King Abdulaziz University, Jeddah, Saudi Arabia; ^4^Cancer and Mutagenesis Unit, King Fahd Center for Medical Research, King Abdulaziz University, Jeddah, Saudi Arabia

**Keywords:** drostanolone heptanoate, microbial transformation, *Cephalosporium aphidicola*, *Fusarium lini*, anti-cancer, cytotoxicity

## Abstract

In search of selective and effective anti-cancer agents, eight metabolites of anti-cancer steroid, drostanolone enanthate (**1**), were synthesized *via* microbial biotransformation. Enzymes such as reductase, oxidase, dehydrogenase, and hydrolase from *Cephalosporium aphidicola*, and *Fusarium lini* were likely involved in the biotransformation of **1** into new metabolites at pH 7.0 and 26°C, yielding five new metabolites, 2α-methyl-3α,14α,17β-trihydroxy-5α-androstane (**2**), 2α-methyl-7α-hydroxy-5α-androstan-3,17-dione (**3**), 2-methylandrosta-11α-hydroxy-1, 4-diene-3,17-dione (**6**), 2-methylandrosta-14α-hydroxy-1,4-diene-3,17-dione (**7**), and 2-methyl-5α-androsta-7α-hydroxy-1-ene-3,17-dione (**8**), along with three known metabolites, 2α-methyl-3α,17β-dihydroxy-5α-androstane (**4**), 2-methylandrosta-1, 4-diene-3,17-dione (**5**), and 2α-methyl-5α-androsta-17β-hydroxy-3-one (**9**), on the basis of NMR, and HREI-MS data, and single-crystal X-ray diffraction techniques. Interestingly, *C. aphidicola* and *F. lini* were able to catalyze hydroxylation only at alpha positions of **1**. Compounds **1–9** showed a varying degree of cytotoxicity against HeLa (human cervical carcinoma), PC3 (human prostate carcinoma), H460 (human lung cancer), and HCT116 (human colon cancer) cancer cell lines. Interestingly, metabolites **4** (IC_50_ = 49.5 ± 2.2 μM), **5** (IC_50_ = 39.8 ± 1.5 μM), **6** (IC_50_ = 40.7 ± 0.9 μM), **7** (IC_50_ = 43.9 ± 2.4 μM), **8** (IC_50_ = 19.6 ± 1.4 μM), and **9** (IC_50_ = 25.1 ± 1.6 μM) were found to be more active against HeLa cancer cell line than the substrate **1** (IC_50_ = 54.7 ± 1.6 μM). Similarly, metabolites **2** (IC_50_ = 84.6 ± 6.4 μM), **3** (IC_50_ = 68.1 ± 1.2 μM), **4** (IC_50_ = 60.4 ± 0.9 μM), **5** (IC_50_ = 84.0 ± 3.1 μM), **6** (IC_50_ = 58.4 ± 1.6 μM), **7** (IC_50_ = 59.1 ± 2.6 μM), **8** (IC_50_ = 51.8 ± 3.4 μM), and **9** (IC_50_ = 57.8 ± 3.2 μM) were identified as more active against PC-3 cancer cell line than the substrate **1** (IC_50_ = 96.2 ± 3.0 μM). Metabolite **9** (IC_50_ = 2.8 ± 0.2 μM) also showed potent anticancer activity against HCT116 cancer cell line than the substrate **1** (IC_50_ = 3.1 ± 3.2 μM). In addition, compounds **1–7** showed no cytotoxicity against 3T3 normal cell line, while compounds **8** (IC_50_ = 74.6 ± 3.7 μM), and **9** (IC_50_ = 62.1 ± 1.2 μM) were found to be weakly cytotoxic.

## Introduction

Microbial transformation is one of the most important approaches for the structural transformation of various classes of organic compounds. This technique has been successfully employed in green chemistry, *i.e*. the drug discovery and development programs, providing an excellent source of compounds around core structures, followed by screening for various biological activities. In several instances, microbial transformation has been used as an important tool for the regio-, chemo-, and stereo-selective conversions of organic compounds which are difficult to achieve by conventional methods (Holland and Weber, 2000; Fernandes et al., 2003; Mihovilovic et al., 2003; Yildirim et al., 2003; Bartmanska et al., 2005; Borges et al., 2009; Choudhary et al., 2011; Ravindran et al., 2012). Due to the inactive nature of hydrocarbon skeleton of steroids, they are often difficult to be derivatized by conventional synthetic methods. Therefore, microbial transformation is often used for the structural alteration of steroids. The presence of P450 cytochrome enzyme systems in fungi, makes whole-cell biocatalysis an efficient tool for stereo-, and regio-specific hydroxylation (Choudhary et al., 2005a; Tong and Dong, 2009; Kristan and RiŽner, 2012; Baydoun et al., 2014).

Drostanolone, and its esters derivatives, such as drostanolone propionate, drostanolone pentanoate, and drostanolone enanthate (**1**) are anabolic-androgenic steroids (AASs) used by athletes to strengthen their muscles without gaining fat. In addition, drostanolone, and its esters derivatives have the ability to inhibit the production of estrogen. Propionate ester of drostanolone is also used for the treatment of breast cancer, under the brand name of Masteron (Chowdhury et al., 1976; Clavel et al., 1982; Marinov et al., 1986; Vardar et al., 2002; Bahrke and Yesalis, 2004).

Cancer is currently recognized as a major public health challenge. Cancers are the second leading cause of death in the United States, and all across the world. According to the World Health Organization (WHO), the prevalence of cancer is exceeding 6 million cases per year. Cancer cells have high proliferation rate. They spread rapidly in the living system, and can survive against strong chemotherapeutics and DNA damaging agents. Similarly, cytotoxic drugs have many adverse effects on normal cells, and thus their use in cancer chemotherapy is a therapeutic challenge. Due to this, development of safe, effective and selective chemotherapeutic agents is urgently needed against various cancers (Munoz-Pinedo et al., 2012; Su et al., 2015; Swadogo et al., 2015; Rebecca et al., 2016).

Breast cancer is one of the most common cancers in 140 countries of the world. It is a major cause of cancer-related death in females all across the world, characterized by the abnormal growth of cells in the breast lobules or ducts with the high proliferation rate (Hanahan and Weinberg, 2000; Ferlay et al., 2015).

Cervical cancer is the second most predominant cancer in females all across the world, after the breast cancer. The main cause of cervical cancer is the formation of malignant cells in tissues of the cervix (Wang et al., 2013; Hafiza and Latifah, 2014; Pariente et al., 2016). The HeLa cancer cell line, obtained from human cervical cancer cells, is a common cellular model to evaluate the cytotoxic potential of test compounds.

Prostate cancer is the second leading cancer in male worldwide after the bronchus cancer, and the third most common cause of cancer death. It is the most common reason of malignancy in men. Its incidence increases with age, more common over the age of 50 years (Henry and Omahony, 1999; De-Bono et al., 2010; Wenbin et al., 2015). The PC-3 cancer cell line, obtained from male prostate cancer cells, is a widely used model to investigate the toxicity of test compounds.

Lung cancer is a highly prevalent cancer among men in the United States since the mid-1950s and among women, since the late 1980s. It is a leading cause of cancer related death. Lung cancer is mostly attributed to smoking (Travis et al., 2002; Villanti et al., 2013; Mishra et al., 2016). The H460 cancer cell line, obtained from human lung cancer cells (lymphogenous metastatic subline of human large cell lung carcinoma), are often used to evaluate cytotoxicity of test compounds.

After the breast, lung, and prostate cancers, colon cancer is the fourth most prevalent cancer all across the world. It is the cancer found in approximately equal frequency in males and females. It is the second most common cause of cancer-related deaths in western countries (Levin et al., 2008; Andre et al., 2009; Ahearn et al., 2012). The HCT116 cancer cell line, obtained from human colon cancer cells, is commonly used to assess the cytotoxicity of test compounds.

In continuation of our studies on the fungal transformation of bioactive steroids (Choudhary et al., 2005b,c, 2007, 2010; Ahmad et al., 2014; Siddiqui et al., 2017), we synthesized analogs of drostanolone enanthate (**1**) through its transformation with *Cephalosporium aphidicola*, and *Fusarium lini*. Metabolites **2–9**, as well as substrate **1**, were evaluated against HeLa (cervical carcinoma), PC-3 (prostate carcinoma), H460 (lung cancer), HCT116 (colon cancer), and 3T3 (mouse fibroblast normal) cell lines, using high-throughput cell-based assay, the most efficient and convenient laboratory method, the MTT assay to predict the response of test compounds in malignancies where they showed specificity against the cancer cells. This study has thus identified anti-cancer metabolites of drostanolone enanthate (**1**) for further studies.

## Materials and methods

### Instrumental analysis

Thin layer chromatography (TLC) (silica gel, 20 × 20, 0.25 mm thick, PF_254_, Merck, Germany) was used for the analysis of degree of transformation and purity. Silica gel column chromatography (70–230 mesh, E. Merck, Germany) was used for the initial purification of metabolites. In addition, preparative reverse phase recycling (JAI LC-908W, Japan), equipped with YMC L-80 (4–5 μm, 20–50 mm i.d.), was used for the final purification of metabolites. Structures of metabolites were elucidated with the help of ^1^H- (400, 500, and 600 MHz), and ^13^C-NMR (100, 125, and 150 MHz) spectra, which were recorded on Bruker Avance-NMR spectrometers (France) in CD_3_OD, CD_3_COCD_3_ or DMSO-*d*_6_. HREI-MS and IR spectra were performed on JEOL JMS-600H (Japan) (double-focusing magnetic sector mass analyzer) mass spectrometer (EI, electron impact ionization), and Bruker Vector 22 FT-IR spectrophotometer, respectively. Optical rotations of metabolites were recorded on JASCO P-2000 polarimeter (Japan). Evolution 300 UV-visible spectrophotometer was used to record the UV spectra. The melting points of the transformed products were measured by using Buchi M-560 (Switzerland) instrument. Single-crystal X-ray diffraction data was collected on Bruker APEXII D8 Venture diffractometer, fitted with PHOTON 100 detector (CMOS Technology) and fine-focus sealed tube having X-ray source (Cu Kα radiations α = 1.54178 Å). Reflection intensities were integrated using SAINT software. Absorption correction was done on Multi-scan, and structure was solved by SHELXTL program (Gerlier and Thomasset, 1986; Sheldrick, 2008; Spek, 2009).

### Microbial cultures

Microbial culture of *C. aphidicola* ATCC 28300 and *F. lini* NRRL 2204 were obtained from the American Type Culture Collection (ATCC) and Northern Regional Research Laboratories (NRRL), respectively. Cultures were grown on Sabouraud dextrose agar (SDA) slant, and maintained at 4°C.

### Media preparation

Four liter of media for the growth of *C. aphidicola* ATCC-28300 was prepared by mixing 200 g of glucose, 4 g of KH_2_PO_4_, 8 g of glycine, 4 g of KCl, 8 g of MgSO_4_.7H_2_O, and 8 mL of trace elements in 4 L of distilled water. Similarly, four liter of media was prepared for *F. lini* NRRL 2204 by mixing 40 g of glucose, 20 g of yeast extract, 20 g of peptone, 20 g of KH_2_PO_4_, 20 g of NaCl, and 40 mL of glycerol in 4 L of distilled water.

### Fermentation of drostanolone enanthate (1) with *C. aphidicola* ATCC 28300, and *F. lini* NRRL 2204

Medium for the growth of *C. aphidicola* and *F. lini* was prepared by mixing above mentioned ingredients in 4 L of distilled water for each fungus, which was then dispensed equally into 40 Erlenmeyer flasks of 250 mL (100 mL in each flask). All these flasks were autoclaved at 121°C, and were inoculated with SDA slants of *C. aphidicola* ATCC 28300 and *F. lini* NRRL 2204 under sterilized conditions. These fungal culture containing flasks were left on the rotary shaker (121 rpm) at 26 ± 2°C for 3–4 days to obtain maximum growth of *C. aphidicola* and *F. lini*. Compound **1** (1 g) was dissolved in 20 mL of acetone for each fungus, and was dispensed 0.5 mL in each flask, and again placed on rotary shaker for 12 days. A negative control (medium + fungal culture), as well as positive control (medium + substrate) were also prepared analogously to evaluate the fungal metabolites and degradation of compound **1**, respectively. After incubation of 12 days, all the flasks were filtered and extracted with 20 L of dichloromethane (DCM), organic phase was separated, and then was evaporated under reduced pressure on rotary evaporator. This yielded 1.5 g of pale yellowish solid crude. This crude material was fractionated over silica gel column chromatography by elution with gradient hexanes and acetone solvent systems. Column chromatography yielded four main fractions (1–4), which were then purified by recycling HPLC. Metabolites **2** (*t*_*R*_ = 16 min, 8 mg, 0.8%), **3** (*t*_*R*_ = 19 min, 11 mg, 1.1%), **4** (*t*_*R*_ = 17 min, 35 mg, 3.5%), **5** (*t*_*R*_ = 23 min, 42 mg, 4.2%), **6** (*t*_*R*_ = 20 min, 9 mg, 0.9%), **7** (*t*_*R*_ = 21 min, 12 mg, 1.2%), and **9** (*t*_*R*_ = 18 min, 5 mg, 0.5%) were purified from fractions 1–7, respectively, through reverse phase recycling HPLC using methanol- water as solvent system (MeOH: H_2_O; 70: 30). Whereas, metabolite **8** was obtained from fraction 8 through normal phase recycling HPLC (*t*_*R*_ = 32 min, CHCl_3_: MeOH; 95: 5, 7.5 mg, 0.75%).

#### 2α-methyl-3α,14α,17β-trihydroxy-5α-androstane (2)

White solid; m. p.: 274–375°C; ${\left[\alpha \right]}_{\text{D}}^{25}$ = −14.8 (*c* 0.0046, CH_3_OH); IR (CHCl_3_): υ_max_ (cm^−1^), 3531 (OH), 3423 (OH); HREI-MS: *m/z* 322.2517 [M^+^], (calcd. 322.2508, C_20_H_34_O_3_); EI-MS: *m/z* (%); 322.2 [M^+^] (2), 304.2 (74), 271.2 (36), 264.2 (82), 110.0 (99); ^1^H-NMR (CD_3_OD, 600 MHz): Table 1; ^13^C-NMR (CD_3_OD, 150 MHz): Table 1.

**Table 1:** ^13^C- and ^1^H-NMR chemical shift data (*J* and *W*_1/2_ in Hz) of compounds **1–3** (δ ppm).

**Carbon**	**1**		**2**		**3**	
	**δ_C_**	**δ_H_ (*J* in Hz)**	**δ_C_**	**δ_H_ (*J* in Hz)**	**δ_C_**	**δ_H_ (*J* in Hz)**
1	49.8	2.10, overlap;	42.3	1.33, overlap;	49.4	2.06, overlap;
		1.07, overlap		1.11, t (*J*_1b, 1a/1b, 2_ = 12.6)		1.09, m
2	42.1	2.55, m	33.1	1.64, overlap	41.9	2.56, m
3	215.4	–	71.4	3.69, br. s ($W_{\frac{1}{2}}$ = 14.3)	215.4	–
4	45.5	2.42, t (*J*_4a, 4b/4a, 5_ = 13.6); 1.98, dd (*J*_4b, 5_ = 14.0, *J*_4b, 4a_ = 3.2)	37.8	1.40, overlap; 1.51, overlap	45.1	2.43, overlap;1.95, dd (*J*_4b, 5_ = 14.0, *J*_4b, 4a_ = 3.6)
5	49.4	1.49, overlap	39.7	1.52, overlap	42.1	2.04, overlap
6	29.7	1.39, overlap;	29.2	1.24, overlap 2 (H)	37.5	1.63, m;
		1.31, overlap				1.48, m
7	32.4	1.74, overlap;	27.0	1.50, overlap 2 (H)	67.1	3.92, br. s ($W_{\frac{1}{2}}$ = 11.6)
		0.95, overlap				
8	36.3	1.50, m	40.1	1.71, overlap	40.2	1.70, overlap
9	55.2	0.80, m	48.8	1.31, overlap	47.0	1.56, m
10	37.7	–	37.5	–	37.8	–
11	22.1	1.63, m;	20.6	1.56, overlap;	21.6	2.08, overlap;
		1.42, m		1.31, overlap		1.72, overlap
12	38.2	1.73, m;	30.0	1.67, overlap;	32.5	1.76, overlap;
		1.19, m		1.48, m		1.19, m
13	43.9	–	48.3	–	49.1	–
14	51.9	1.09, overlap	84.8	–	47.1	1.68, overlap
15	24.5	1.65, m;	32.7	1.62, overlap;	22.1	1.58, m;
		1.33, m		1.53, overlap		1.47, m
16	28.5	2.13, m;	29.9	2.12, m;	36.6	2.42, overlap;
		1.49, m		1.49, overlap		2.10, overlap
17	84.0	4.60, t (*J*_17, 16ab_ = 8.4)	79.6	4.19, t (*J*_17, 16ab_ = 7.8)	223.8	–
18	12.7	0.84, s	15.7	0.81, s	14.9	0.88, s
19	12.6	1.12, s	12.3	0.82, s	11.5	1.12, s
20	14.9	0.96, d (*J*_20, 2_ = 6.4)	19.0	0.91, d (*J*_20, 2_ = 7.2)	13.8	0.97, d (*J*_20, 2_ = 6.4)
21	175.5	–				
22	35.3	2.29, t 2(H)				
		(*J*_22/23_ = 7.2)				
23	26.2	1.59, m 2(H)				
24	29.8	1.38, overlap;				
		1.32, overlap				
25	32.6	1.29, overlap 2(H)				
26	23.5	1.34, m;				
		1.30, overlap				
27	14.3	0.90, t (*J*_27, 26ab_ = 6.4)				

#### 2α-methyl-7α-hydroxy-5α-androstan-3,17-dione (3)

White solid; m. p.: 230–234°C; ${\left[\alpha \right]}_{\text{D}}^{25}$ = −34.1 (*c* 0.0017, CH_3_OH); IR (CHCl_3_): υ_max_ (cm^−1^), 3436 (O-H), 1711 (C = O); HREI-MS: *m/z* 318.2192 [M^+^] (calcd. 318.2195, C_20_H_30_O_3_); EI-MS *m/z* (%): 318.1 [M^+^] (7), 300.1 (36), 147.1 (16.2), 136.1 (100); ^1^H-NMR (CD_3_OD, 400 MHz): Table 1; ^13^C-NMR (CD_3_OD, 150 MHz): Table 1.

#### 2α-methyl-3α,17β-dihydroxy-5α-androstane (4)

White solid; m. p.: 242–245°C; ${\left[\alpha \right]}_{\text{D}}^{25}$ = +34.1 (*c* 0.0077, CH_3_OH), IR (CHCl_3_): υ_max_ (cm^−1^), 3315 (OH); HREI-MS: *m/z* 306.2553 [M^+^] (calcd. 306.2559, C_20_H_34_O_2_); EI-MS *m/z* (%): 306.3 [M^+^] (75), 291.2 (61), 229.2 (98), 179.1 (93), 121.1 (100); ^1^H-NMR (CDCl_3_, 600 MHz): Table 2; ^13^C-NMR (CDCl_3_, 150 MHz): Table 2.

**Table 2:** ^13^C- and ^1^H-NMR chemical shift data (*J* and *W*_1/2_ in Hz) of compounds **4**–**6** (δ ppm).

**Carbon**	**4**		**5**		**6**	
	**δ_C_**	**δ_H_ (*J* in Hz)**	**δ_C_**	**δ_H_ (*J* in Hz)**	**δ_C_**	**δ_H_ (*J* in Hz)**
1	40.8	1.34, m;	154.4	7.06, s	158.0	7.69, s
		0.98, m				
2	31.7	1.40, overlap	134.3	–	131.3	–
3	70.7	3.75, br. s ($W_{\frac{1}{2}}$ = 14.2)	188.9	–	189.4	–
4	36.7	1.77, td (*J*_4a, 5_ = 9.0,	123.8	6.05, s	124.3	6.05, s
		*J*_4a, 4b_ = *J*_4a, 3_ = 3.6); 1.51, m				
5	38.7	1.43, overlap	172.5	–	171.7	–
6	28.0	1.22, m;	33.2	2.57, m;	33.5	2.58, m;
		1.16, m		2.45, overlap		2.14, m
7	31.5	0.88, overlap 2(H)	32.4	1.80, overlap;	36.6	1.98, m;
				1.26, overlap		1.91, m
8	35.3	1.37, m	36.2	1.91, m	35.0	1.93, m
9	54.5	0.69, m	54.2	1.05, m	62.0	1.10, m
10	36.5	–	45.0	–	45.6	–
11	20.3	1.63, m;	22.8	1.69, m;	68.2	4.05, td (*J*_11, 9_ = *J*_11, 12a_ = 10.5, *J*_11, 12b_ = 5.1)
		1.58, m		1.62, m		
12	36.4	1.44, m;	33.8	2.10, m;	33.7	2.09, m;
		1.01, overlap		1.13, m		1.16, m
13	42.9	–	49.5	–	49.4	–
14	51.0	0.94, m	51.6	1.33, m	50.8	1.38, m
15	23.3	1.56, m;	23.3	1.94, overlap;	22.7	1.92, overlap;
		1.25, overlap		1.76, m		1.61, m
16	30.5	2.02, m;	36.5	2.47, overlap;	43.0	2.10, m;
		1.39, overlap		2.07, m		1.19, m
17	81.9	3.61, t (*J*_17, 16ab_ = 8.4)	223.0	–	221.4	–
18	11.1	0.71, s	14.1	0.96, s	14.8	0.96, s
19	12.1	0.77, s	19.1	1.29, overlap	19.1	1.33, overlap
20	18.4	0.91, d (*J*_20, 2_ = 7.0)	15.8	1.85, s	15.9	1.82, s

#### 2-methylandrosta-1,4-diene-3,17-dione (5)

White solid; m. p.: 198–202°C; UV λ_max_: 229 nm (CH_3_OH, log ε 2.04); ${\left[\alpha \right]}_{\text{D}}^{25}$ = +39.0 (*c* 0.018, CH_3_OH); IR (CHCl_3_): υ_max_ (cm^−1^), 1737 (C = O stretching), 1665, 1625 (α, β-unsaturated ketone); HREI-MS: *m/z* 298.1944 [M^+^] (calcd. 298.1933, C_20_H_26_O_2_); EI-MS *m/z* (%): 298.0 [M^+^] (55), 280.0 (8), 197.9 (28), 169.9 (23), 152.9 (99), 136.0 (100); ^1^H-NMR (CD_3_OD, 500 MHz): Table 2; ^13^C-NMR (CD_3_OD, 125 MHz): Table 2.

#### 2-methylandrosta-11α-hydroxy-1,4-diene-3,17-dione (6)

White solid; m. p.: 230–234°C; UV λ_max_: 248 nm (CH_3_OH, log ε 6.91); ${\left[\alpha \right]}_{\text{D}}^{25}$ = −21 (*c* 0.0012, CH_3_OH); IR (CHCl_3_): υ_max_ (cm^−1^), 3436 (OH),1736 (C = O), 1661, 1621 (α, β-unsaturated ketone); HREI-MS: *m/z* 314.1897 [M^+^] (calcd. 314.1882, C_20_H_26_O_3_); EI-MS *m/z* (%): 314.2 [M^+^] (45), 296.2 (15), 148.1 (11), 136.1 (100), 135.1 (50), 121.1 (16); ^1^H-NMR (CD_3_OD, 300 MHz): Table 2; ^13^C-NMR (CD_3_OD, 150 MHz): Table 2.

#### 2-methylandrosta-14α-hydroxy-1,4-diene-3,17-dione (7)

White solid; m. p.: 224–228°C; UV λ_max_: 248 nm (CH_3_OH, log ε 6.98); ${\left[\alpha \right]}_{\text{D}}^{25}$ = −52 (*c* 0.0014, CH_3_OH); IR (CHCl_3_): υ_max_ (cm^−1^), 3468 (OH), 1727 (C = O), 1666, 1628 (α, β-unsaturated ketone); HREI-MS: *m/z* 314.1861 [M^+^] (calcd. 314.1882, C_20_H_26_O_3_); EI-MS *m/z* (%): 314.2 [M^+^] (57), 286.1 (8), 136.1 (62), 135.1 (100), 105.0 (23). ^1^H-NMR (CD_3_OD, 600 MHz): Table 3; ^13^C-NMR (CD_3_OD, 125 MHz): Table 3.

**Table 3:** ^13^C- and ^1^H-NMR chemical shift data (*J* and *W*_1/2_ in Hz) of compounds **7**–**9** (δ ppm).

**Carbon**	**7**		**8**		**9**	
	**δ_C_**	**δ_H_ (*J* in Hz)**	**δ_C_**	**δ_H_ (*J* in Hz)**	**δ_C_**	**δ_H_ (*J* in Hz)**
1	153.8	7.05, s	155.5	7.02, s	49.9	2.09, dd (*J*_1a, 2_ = 12.8, *J*_1a, 1b_ = 5.2); 1.07, overlap
2	134.6	–	134.3	–	42.1	2.56, m
3	188.8	–	202.3	–	215.7	–
4	123.7	6.05, s	41.3	2.40, m;	45.6	2.41, t (*J*_4a, 4b/4a, 5_ = 16.0);
				2.13, overlap		1.98, overlap
5	171.9	–	38.0	2.47, overlap	49.6	1.48, overlap
6	33.5	2.56, overlap;	36.2	1.70, m;	29.7	1.37, m;
		2.42, overlap		1.55, overlap		1.32, m
7	33.0	2.18, m;	66.7	3.93, br. s ($W_{\frac{1}{2}}$ = 12.3)	32.5	1.71, m;
		1.12, m				0.92, m
8	38.9	1.57, m	40.5	1.72, overlap	36.6	1.44, m
9	46.6	1.44, m	44.0	1.51, overlap	55.4	0.72, m
10	44.6	–	40.2	–	37.7	–
11	24.6	2.02, m;	21.3	1.95, m;	22.2	1.78, m;
		1.67, overlap		1.52, overlap		1.43, overlap
12	29.2	2.69, m;	32.5	1.79, m;	38.0	1.82, m;
		2.58, m		1.28, m		1.05, overlap
13	52.7	–	48.2	–	44.1	–
14	84.7	–	47.3	1.72, overlap	52.1	0.98, overlap
15	20.8	1.70, overlap;	22.0	2.09, overlap;	24.3	1.58, m;
		1.41, overlap		1.60, m		1.25, m
16	40.2	1.97, overlap;	36.5	2.44, overlap;	30.6	1.96, m;
		1.66, overlap		2.08, overlap		1.42, overlap
17	223.0	–	223.4	–	82.4	3.55, t (*J*_17, 16ab_ = 6.0)
18	19.1	1.23, s	13.9	0.91, s	11.6	0.74, s
19	20.2	1.39, s	12.1	1.01, s	12.6	1.11, s
20	15.8	1.86, s	16.1	1.71, s	14.9	0.96, d (*J*_20, 2_ = 6.8)

#### 2-methylandrosta-7α-hydroxy-1-ene-3,17-dione (8)

White solid; m. p.: 250–256°C; UV λ_max_: 230 nm (CH_3_OH, log ε 6.32) ${\left[\alpha \right]}_{\text{D}}^{25}$ = + 35.1 (*c* 0.0077, CH_3_OH), IR (CHCl_3_): υ_max_ (cm^−1^), 3417 (OH); 1736 (C = O); HREI-MS: *m/z* 316.2042 [M^+^] (calcd. 316.2038, C_20_H_28_O_3_); EI-MS *m/z* (%): 316.2 [M^+^] (88), 270.2 (50), 159.1 (26.6), 136.1 (59.1), 123.1 (65.4); ^1^H-NMR (CD_3_OD, 400 MHz): Table 3; ^13^C-NMR (CD_3_OD, 100 MHz): Table 3.

#### 2α -methyl-5α-androstane-17β-hydroxy-3-one (9)

White solid; m. p.: 151–153°C; ${\left[\alpha \right]}_{\text{D}}^{25}$ = +16.2 (*c* 0.00065, CH_3_OH), IR (CHCl_3_): υ_max_ (cm^−1^), 3437 (OH); HREI-MS: *m/z* 304.2403 [M^+^] (calcd. 304.2402, C_20_H_32_O_2_); EI-MS *m/z* (%): 304.3 [M^+^] (63.7), 245.2 (84), 138.1 (18), 95.1 (24), 91.1 (100); ^1^H-NMR (CD_3_OD, 400 MHz): Table 3; ^13^C-NMR (CD_3_OD, 100 MHz): Table 3.

### Experimental protocol for cytotoxicity

The cytotoxicity of samples was measured against HeLa (human cervical carcinoma ATCC CCl-2), PC-3 (human prostate cancer ATCC CRL-1435), NCl-H460 (human lung carcinoma ATCC HTB-177), HCT116 (human colon cancer ATCC CCl-247), and 3T3 (control fibroblast normal ATCC CRL-1658) cell lines by using standard MTT assay. Cell lines were grown in DMEM F12 media, supplemented with 10% FBS under a 5% CO_2_ atmosphere at 37°C in an incubator.

The colorimetric assay MTT [3-(4, 5-dimethyl thiazol-2yl)-2, 5-diphenyl tetrazolium bromide] was used for the evaluation of cell metabolic activity. In this assay, the yellow MTT is reduced to purple formazon in the mitochondria of the living cells (Gerlier and Thomasset, 1986). The more the living cells, the more the color and thus more absorbance is observed by colorimeter (Fesahat et al., 2015). We have used this method to analyze the effect of our transformed products on cellular cytotoxicity against cancer cells. Around 10,000 cells of each cell line (HeLa, PC3, H460, HCT116 cancers, and 3T3 normal) were seeded in 96-well plates. After reaching 80–90% confluency, they were treated with various concentrations of compounds (25, 50, 75, 100, and 200 μM) for 24 h. All compounds were dissolved in sterile DMSO to make a 200 mM stock solution. The solution was filtered, and serial dilutions were made in growth medium. 600 μL of each dilution was used to treat 3 wells (triplicate of each) of 96 well plate i.e., 200 μL each. Each experiment was performed in three biological replicates. MTT was added, and incubated for 4 h. After removal of MTT, the purple formazon crystals were dissolved in DMSO, and reading was observed at 540 and 570 nm for normal and cancer cells, respectively. The Multiskan reader gives λ_max_ at 540 nm in DMSO treated 3T3 fibroblast cells (that are more confluent in morphology) whereas in case of cancer cells, 570 nm gives λ_max_ (Bonmati-Carrion et al., 2013; Danihelová et al., 2013). A broad range of epithelial carcinoma cell lines were used in this experiment where cisplatin was a common drug of choice. It targets DNA, interferes with cell division during mitosis and induces apoptosis (Eastman, 1999). IC_50_ values were calculated to determine the minimum concentration required to kill 50% of the cells.

$$\begin{array}{lll} \%\;Inhibition & = & 100-\frac{\left(At-Ab\right)}{\left(Ac-Ab\right)}\;\times 100\\ \%\;Cell\;survival & = & \frac{\left(At-Ab\right)}{\left(Ac-Ab\right)}\;\times 100 \end{array}$$

Whereas,

*At* = Absorbance value of test compound

*Ab* = Absorbance value of blank

*Ac* = Absorbance value of control

## Results and discussion

### Structure elucidation

**Figure 1:**
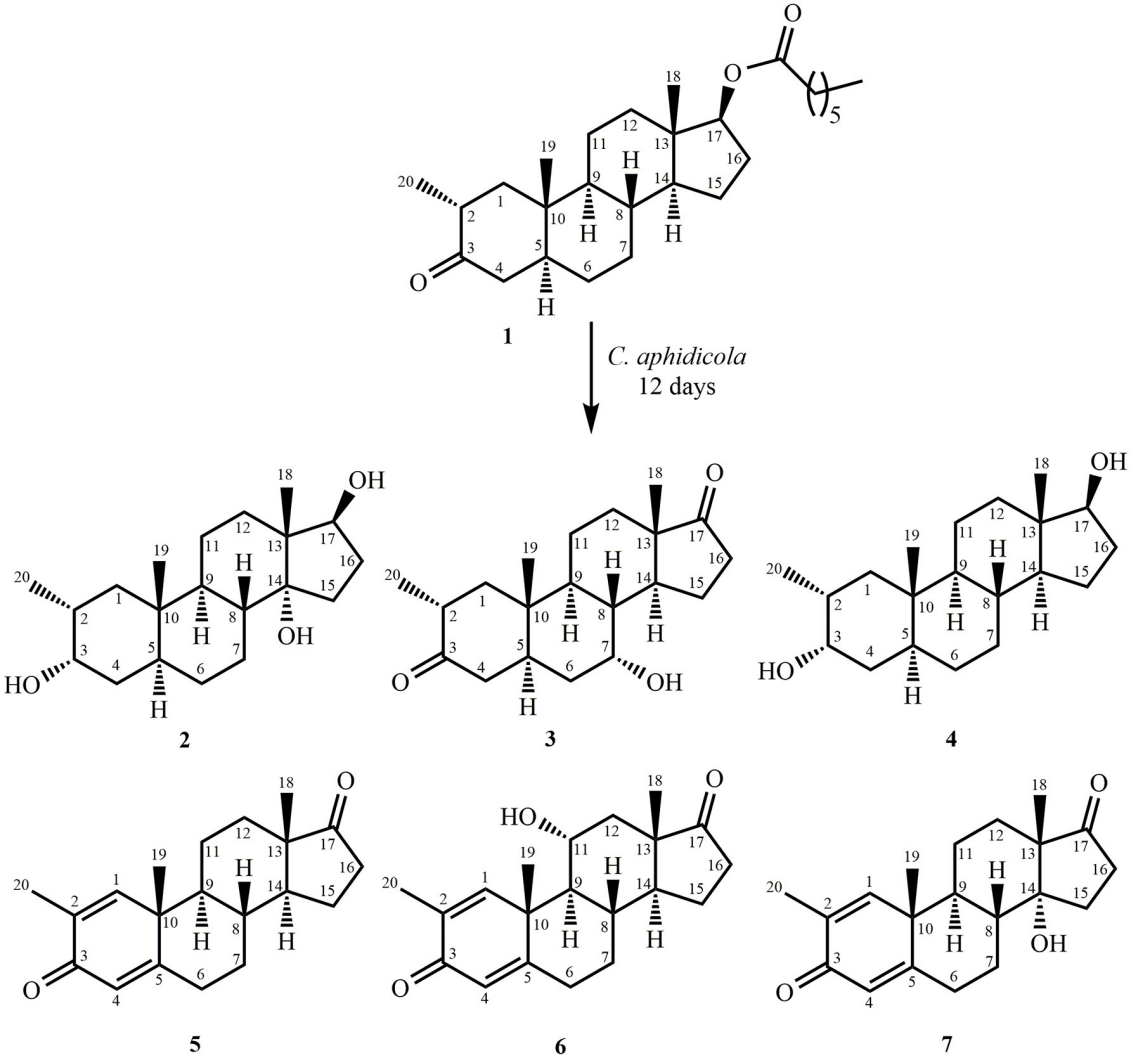
Biotransformation of drostanolone enanthate (**1**) with *Cephalosporium aphidicola*.

**Figure 2:**
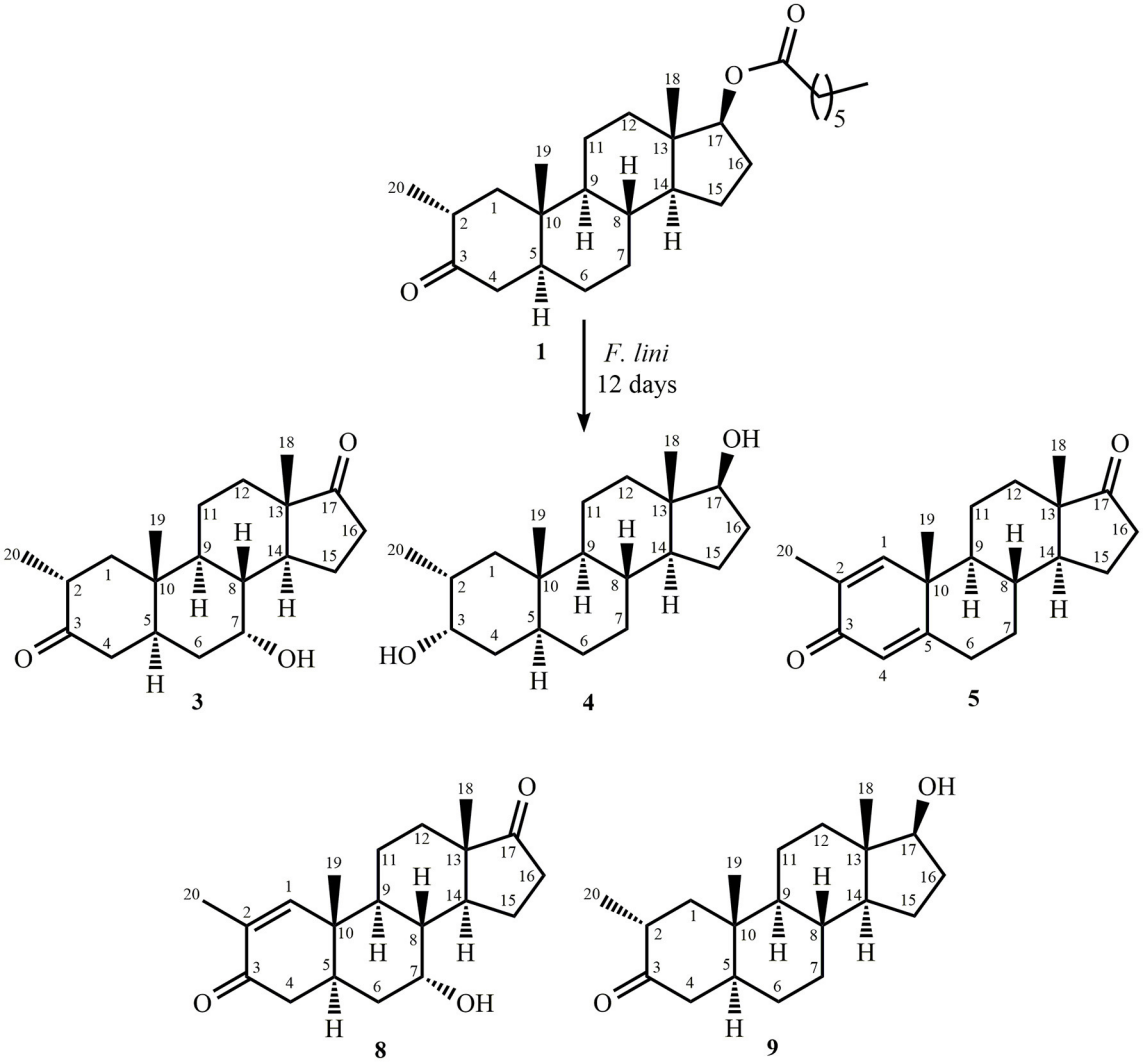
Biotransformation of drostanolone enanthate (**1**) with *Fusarium lini*.

Fermentation of drostanolone enanthate (**1**) (C_27_H_44_O_3_ [M^+^] at *m/z* 416.4), (Data sheet 1) with *C. aphidicola* and *F. lini* is being reported here. Incubation of **1** with *C. aphidicola* afforded six metabolites **2–7** (Figure 1), while with *F. lini* yielded five metabolites **3–5**, and **8**–**9** (Figure 2). Detail descriptions of new metabolites **2**, **3**, **6**, **7**, and **8** are narrated below.

**Figure 3:**
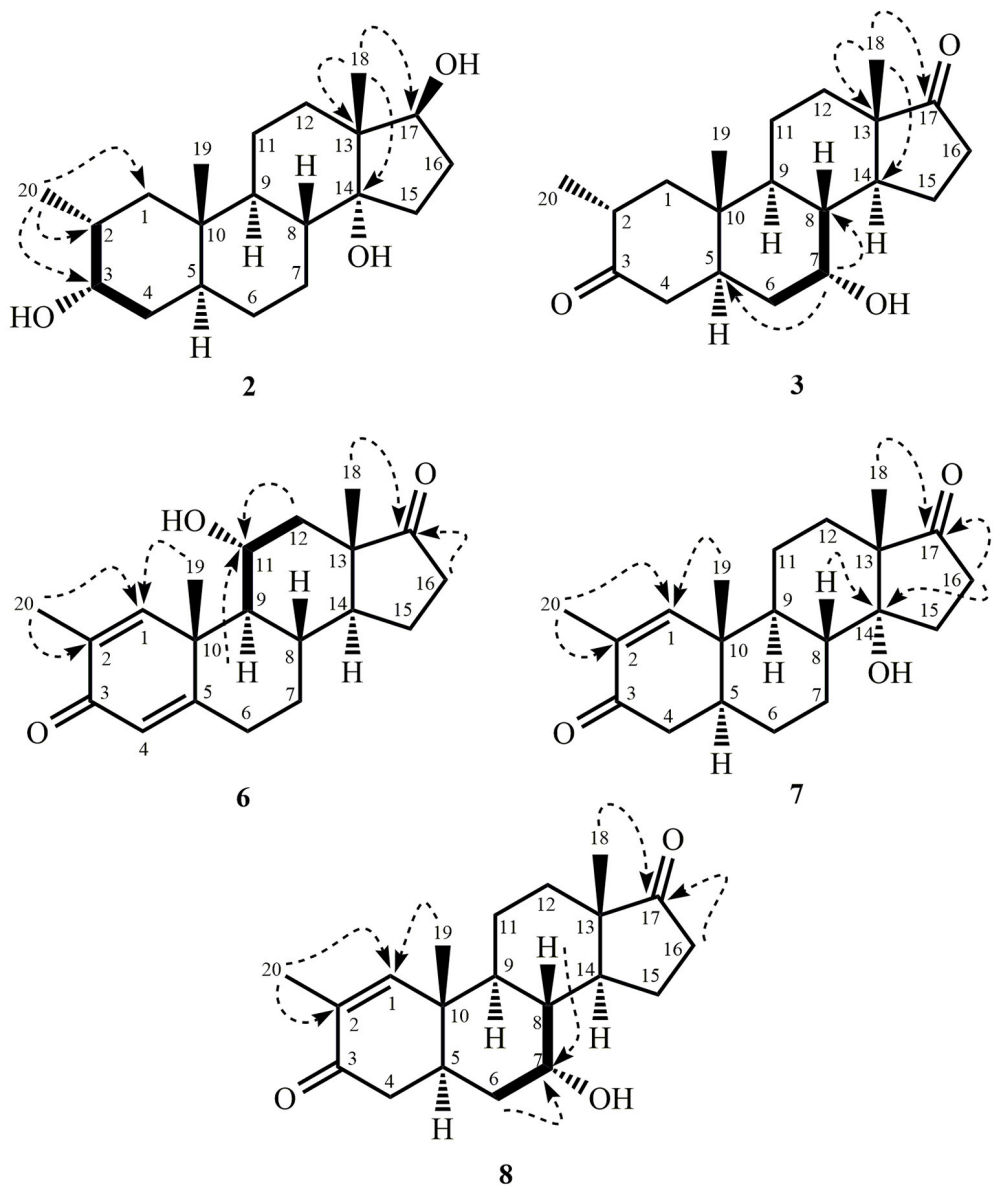
Key HMBC (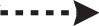), and COSY (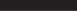) correlations in new metabolites.

**Figure 4:**
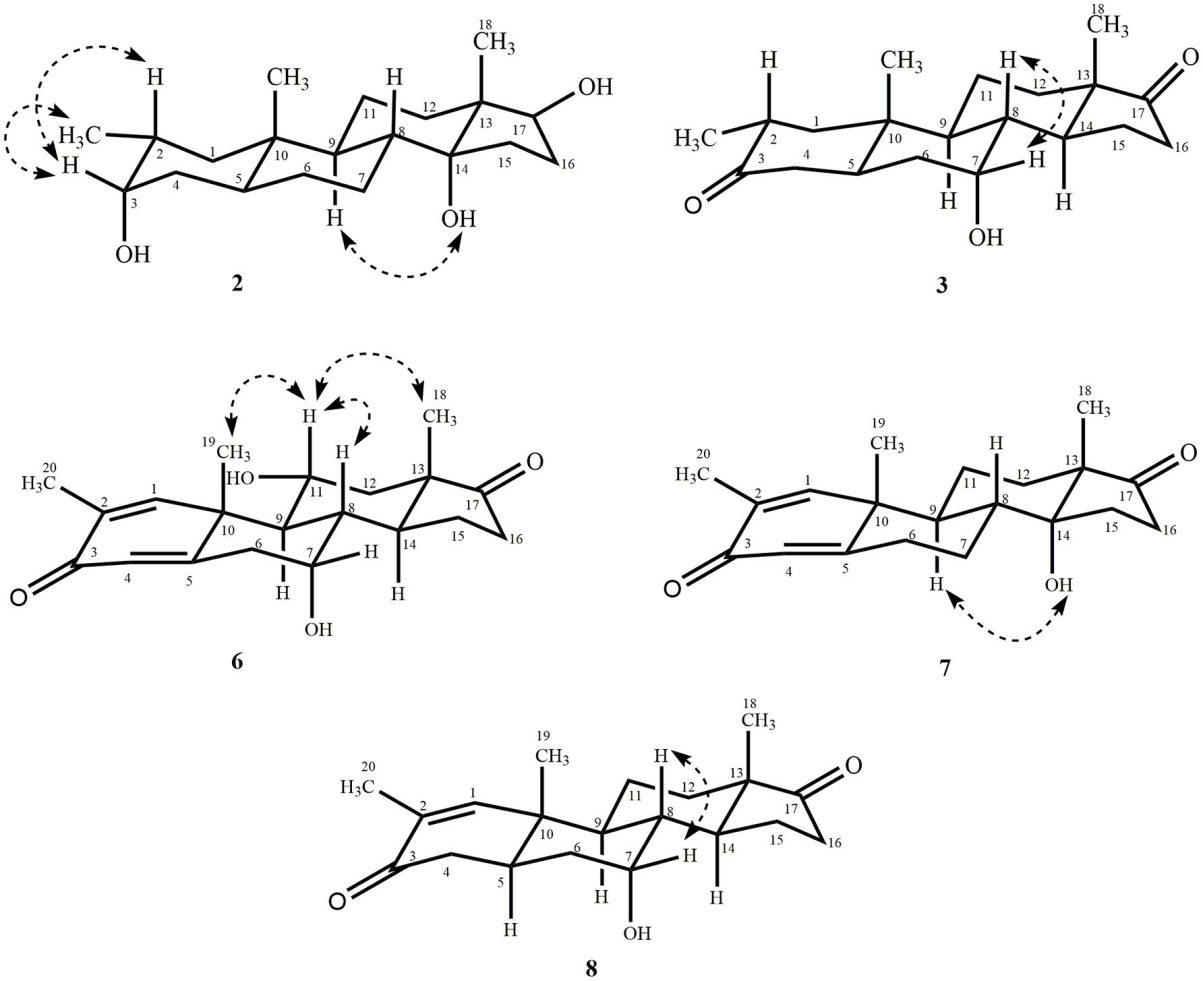
Key NOESY correlations in new metabolites.

**Figure 5:**
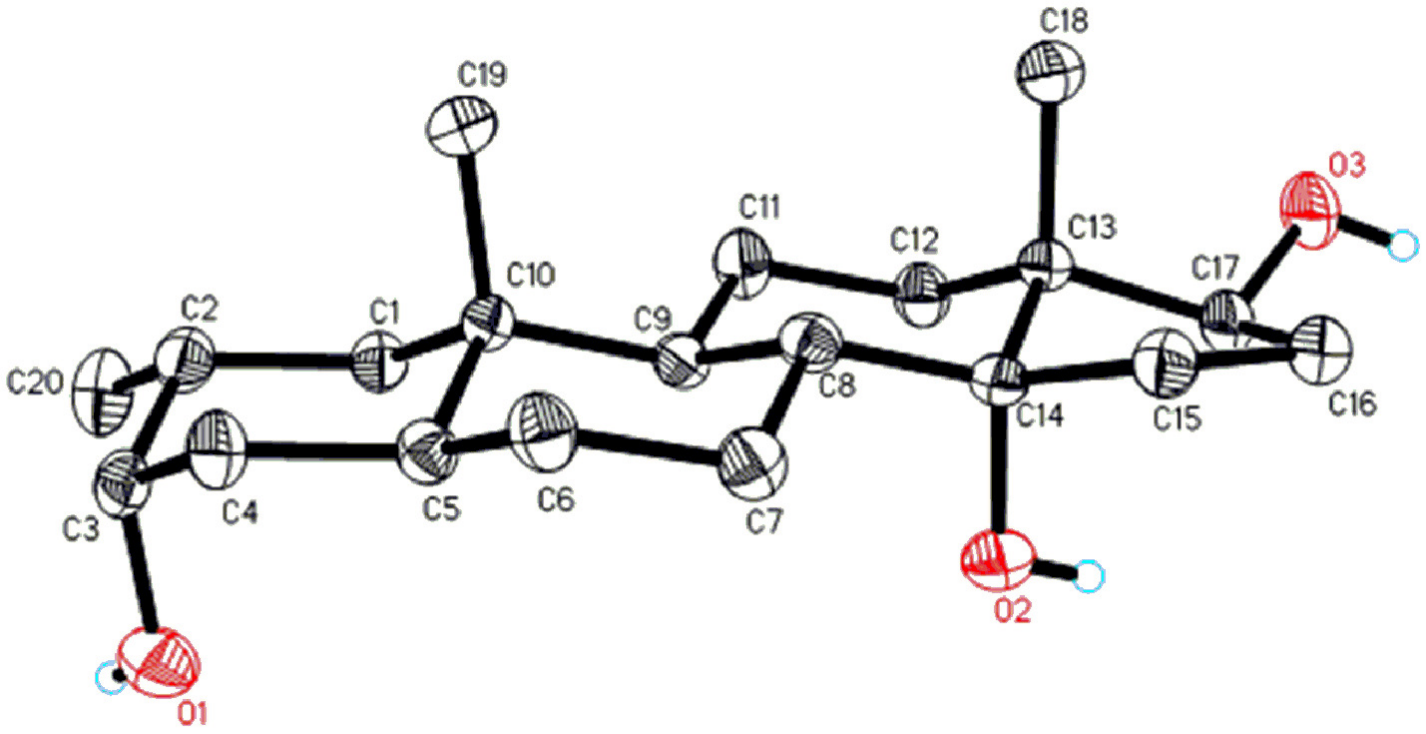
Computer-generated ORTEP drawing of final X-ray model of compound **2**. Color codes: carbon, black; hydrogen, white; oxygen, red.

Metabolite **2** was isolated as white crystals. The molecular formula C_20_H_30_O_3_ was based on HREI-MS which showed the [M^+^] at *m/z* 322.2517 (calcd. 322.2508, C_20_H_34_O_3_), suggesting the addition of three hydrogen, and an oxygen atom, reduction of C-3 ketonic carbonyl, and hydrolytic cleavage of heptanoate side chain in substrate **1**. The IR absorbance at 3,531, and 3,423 cm^−1^ were due to presence of hydroxyl groups. The ^1^H-NMR spectrum showed an additional methine proton signal at δ 3.69 (Table 1) (Data sheet 2). The signals for heptanoate side chain were also found missing in ^1^H-NMR spectrum of compound **2**. The ^13^C-NMR spectrum showed signals for a new methine carbon at δ 71.4, and a new quaternary carbon at δ 84.8. Carbon signals for heptanoate chain and ketonic carbonyl were also found missing in the ^13^C-NMR spectrum. This suggested reduction of C-3 carbonyl, hydrolytic loss of heptanoate chain, and addition of an OH at C-14 (Table 1). The OH group was placed at C-3 on the basis of HMBC correlations of H_3_-20 (δ 0.91, d) with C-3 (δ 71.4) (Figure 3). This resulted from the reduction of C-3 carbonyl. Second OH group was placed at C-14, based on the HMBC correlation of H_3_-18 (δ 0.81, s) with C-14 (δ 84.8). The third OH was at C-17, resulted from hydrolytic loss of heptanoate moiety. The O-H group at C-3 was deduced to be α-oriented based on the NOESY correlations of H-3 (δ 3.69, br. s) with β-oriented H-2 (δ 1.64, overlap), and α-oriented H_3_-20 (δ 0.91, d) (Figure 4). The OH-14 was deduced to be α-oriented, based on NOESY correlations of H-9 (δ 1.32, m) with OH-14 (δ 2.66, s) (acetone-*d*_6_). Single-crystal X-ray diffraction analysis further supported the structure of metabolite **2**, comprised of three rings in chair conformation (A, B, and C), and one in envelop conformation (D). Three OH groups at C-3, C-14, and C-17 were assigned, α-, α-, and β-orientation (Figure 5). Single-crystal diffraction data of metabolite **2** was submitted to Cambridge Crystallographic Data Collection (CCDC 1500706). The structure of metabolite **2** was thus deduced as 2α-methyl-3α,14α,17β-trihydroxy-5α-androstane.

Metabolite **3** was obtained as a white solid. The HREI-MS displayed the [M^+^] at *m/z* 318.2192 (calcd. 318.2195, C_20_H_30_O_3_). This represented the loss of heptanoate side chain, and addition of an oxygen atom in substrate **1**. IR spectrum showed absorbances for OH (3,436 cm^−1^) and ketonic carbonyls (1,711 cm^−1^). In the ^1^H-NMR spectrum, signals of heptanoate chain and C-17 methine proton were found missing, whereas a new downfield methine proton signal was observed at δ 3.92 (Table 1) (Data sheet 3). This suggested hydroxylation of steroidal skeleton and oxidative cleavage of heptanoate moiety. The ^13^C-NMR spectrum also supported the above inferences. Carbon signals for heptanoate moiety were found missing, whereas a new ketonic carbonyl and an oxy-methine signals appeared in ^13^C-NMR spectrum (Table 1). This indicated oxidative cleavage of heptanoate chain and hydroxylation in substrate **1**. The newly appeared methine proton at δ 3.92 (H-7) showed HMBC correlations with C-5, and C-8, suggesting an OH at C-7 (Figure 3). The HMBC correlations of H_3_-18 and H_2_-16 with a carbon at δ 223.8 suggested a ketonic carbon at C-17. OH at C-7 was further supported by COSY correlations of H-7 with H_2_-6 and H-8. H-7 (δ 3.92, s) showed NOESY correlations with *axially* oriented H-8 (δ 1.70, overlap), which suggested an α-orientation of OH at C-7 (Figure 4). Thus, the structure of metabolite **3** was deduced as 2α-methyl-7α-hydroxy-5α-androstan-3,17-dione.

Metabolite **6**, a white solid, displayed the [M^+^] in the HREI-MS at *m/z* 314.1897 (calcd. 314.1882, C_20_H_26_O_3_), due to the loss of heptanoate side chain, addition of an oxygen atom, and loss of five hydrogen atoms in substrate **1**. IR spectrum showed absorbances for OH (3,436 cm^−1^), ketone (1,736 cm^−1^), and enone carbonyl (1,661, 1,621 cm^−1^). The ^1^H-NMR signals for heptanoate protons were found missing, whereas new olefinic (δ 7.69, s; 6.05, s) and an oxymethine (δ 4.05, td) protons were appeared (Table 2) (Data sheet 6). Carbon signals for heptanoate chain were also found missing, whereas a new ketonic carbonyl carbon (δ 223.0), an oxy-methine carbon (δ 68.2), and four olefinic carbons (δ 158.0, 131.3, 124.3, 171.7) appeared in the ^13^C-NMR spectrum (Table 2). This indicated hydroxylation of steroidal skeleton, along with the oxidative hydrolysis of heptanoate ester moiety, and formation of double bonds in substrate **1**. One C = C was placed between C-1/C-2, based on the HMBC correlations of H_3_-19 and H_3_-20 with newly formed olefinic carbon at δ 158.0 (C-1), while another C = C was placed between C-4/C-5, based on the HMBC correlations of H_3_-19 with another newly formed olefinic carbon at δ 171.7 (C-5) and H-1 with olefinic carbon at δ 124.3 (C-4) (Figure 3). Position of OH-11 was deduced through the HMBC correlations of H-12, and H-9 with newly formed methine carbon at δ 68.2 (C-11). OH at C-11 was further supported by COSY correlations of H-11 with H_2_-12 and H-9. Newly formed ketonic carbonyl carbon was placed at C-17, based on the HMBC correlations of H_3_-18 and H_2_-16 with newly formed carbon at δ 221.4 (C-17). NOESY correlations of H-11 (δ 4.05, td) with *axially* oriented H-8 (δ 1.93, m), H_3_-18 (δ 0.96, s), and H_3_-19 (δ 1.33, overlap) suggested an OH group at alpha (Figure 4). Thus, on the basis of above observations, the structure of compound **6** was deduced as 2-methylandrosta-11α-hydroxy-1,4-diene-3,17-dione.

Metabolite **7**, a white solid, displayed the [M^+^] in the HREI-MS at *m/z* 314.1861 (calcd. 314.1882, C_20_H_26_O_3_), due to the loss of heptanoate side chain, addition of an oxygen atom, and loss of five hydrogen atoms in substrate **1**. IR spectrum showed absorbances for OH (3,468 cm^−1^), ketone (1,727 cm^−1^), and enone carbonyl (1,666, 1,628 cm^−1^). The ^1^H-NMR signals for heptanoate protons were found missing, whereas new olefinic protons (δ 7.05, s; 6.05, s) appeared (Table 3) (Data sheet 7). Carbon signals for heptanoate chain were also found missing, whereas a new ketonic carbonyl carbon (δ 223.0), an oxy-methine carbon (δ 84.7), and four olefinic carbons (δ 153.8, 134.6, 123.7, 171.9) appeared in the ^13^C-NMR spectrum (Table 3). This suggested hydroxylation of steroidal skeleton, along with the oxidative hydrolysis of heptanoate ester moiety, and formation of double bonds in ring A. One C = C was placed between C-1/C-2, based on the HMBC correlations of H_3_-19, H_3_-20, and H-4 with newly formed olefinic carbon at δ 153.8 (C-1), while another C = C was placed between C-4/C-5, based on the HMBC correlations of H_3_-19 and H_2_-6 with another newly formed olefinic carbon at δ 171.9 (C-5) and H_2_-6 with olefinic carbon at δ 123.7 (C-4) (Figure 3). The OH group was placed at C-14, based on the HMBC correlations of H_2_-16, and H-9 with newly formed methine carbon at δ 84.7 (C-14). Newly formed ketonic carbonyl was placed at C-17, based on the HMBC correlations of H_3_-18 and H_2_-16 with newly formed carbon at δ 223.0 (C-17). The OH-14 was deduced to be α-oriented, based on the NOESY correlations of H-9 (δ 1.35, m) with OH-14 (δ 2.72, s) (acetone-*d*_6_) (Figure 4). Thus, on the basis of above observations, the structure of compound **7** was deduced as. 2-methylandrosta-14α-hydroxy-1,4-diene-3,17-dione.

Metabolite **8**, a white solid, displayed the [M^+^] in the HREI-MS at *m/z* 316.2042 (calcd. 316.2038, C_20_H_28_O_3_), due to the loss of heptanoate side chain, addition of an oxygen atom, and loss of three hydrogen atoms in substrate **1**. IR spectrum showed absorbances for OH (3,417 cm^−1^), enone (1,653 cm^−1^), and carbonyl (1,736 cm^−1^). The ^1^H-NMR signals for heptanoate protons were found missing, whereas new olefinic (δ 7.02, s) and an oxymethine protons (δ 3.93, s) appeared in the spectrum of metabolite **8** (Table 3) (Data sheet 8). Carbon signals for heptanoate chain were also found missing, whereas a new ketonic carbonyl carbon (δ 223.4), an oxy-methine carbon (δ 66.7), and two olefinic carbons (δ 155.6, 134.3) appeared in the ^13^C-NMR spectrum (Table 3). This suggested hydroxylation of steroidal skeleton, along with the hydrolysis of heptanoate ester moiety and subsequent oxidation at C-17. A new C = C was placed between C-1/C-2, based on the HMBC correlations of H_3_-19 with newly formed olefinic carbon at δ 155.6 (C-1), and H_3_-20 with olefinic carbon at δ 134.3 (C-2) (Figure 3). The OH group was placed at C-7, based on the HMBC correlations of H-8 and H_2_-6 with newly formed methine carbon at δ 66.7 (C-7). OH at C-7 was further supported by COSY correlations of H-7 with H_2_-6 and H-8. Newly formed ketonic carbonyl carbon was deduced to be at C-17, as inferred from the HMBC correlations of H_3_-18 and H_2_-16 with newly formed carbon at δ 223.4 (C-17). NOESY correlations of H-7 (δ 3.93, s) with *axially* oriented H-8 (δ 1.72, overlap) (Figure 4). Thus, on the basis of above discussion, the structure of compound **8** was deduced as 2-methyl-5α-androsta-1-ene-3,17-dione.

Metabolites **4**, **5**, and **9** were identified as known metabolites, i.e., 2α-methyl-3α,17β-dihydroxy-5α-androstane (**4**) (Data sheet 4), 2-methylandrosta-1,4-diene-3,17-dione (**5**) (Data sheet 5), and 2-methyl-17β-hydroxy-5α-androstane-3-one (**9**) (Data sheet 9), by comparing their spectral data with the previously reported data. Metabolite **4** was previously reported by Fragkaki et al. through the metabolism in human body (Fragkaki et al., 2009). Whereas metabolite **5** was previously reported by Numazawa et al. through chemical modification of 2-methyl-4-androstene-3,17-dione (Numazawa et al., 2004). Similarly, metabolite **9** (drostanolone) was also obtained through the biotransformation of compound **1**. Drostanolone was used as a starting material for the synthesis of drostanolone propionate, and drostanolone enanthate (**1**), and other derivatives.

**Figure 6:**
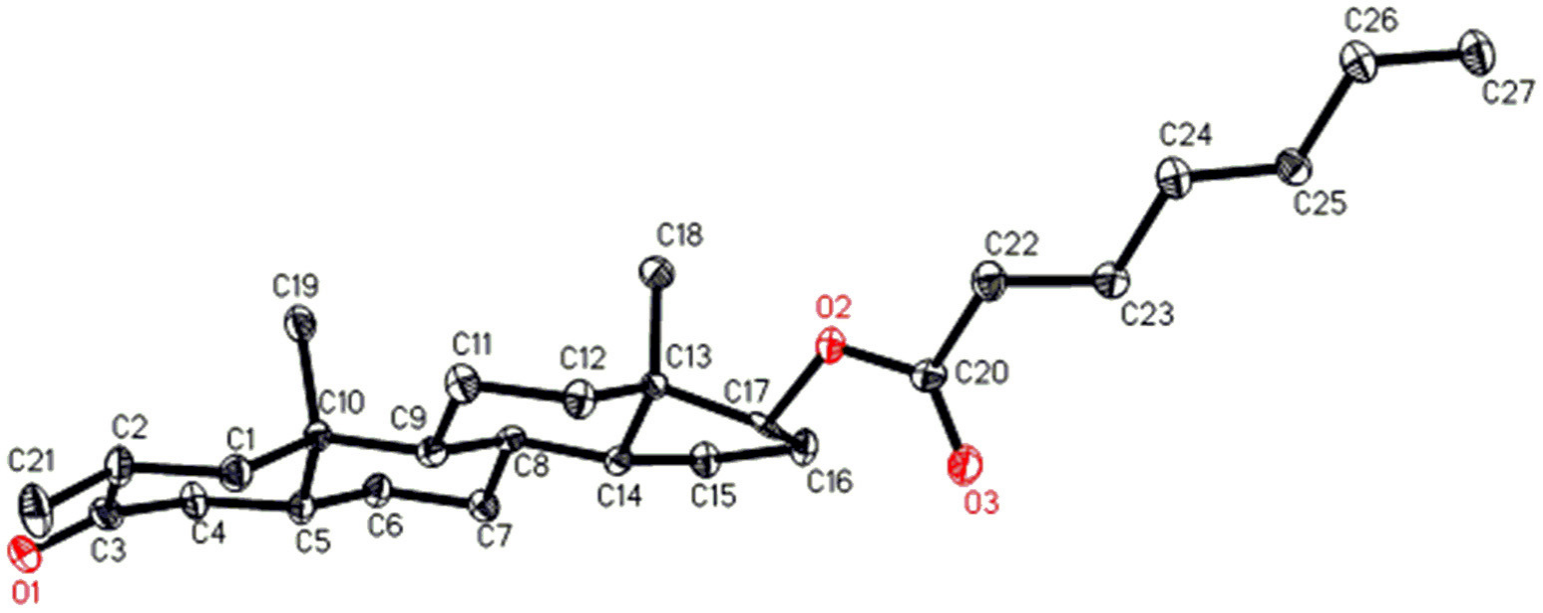
Computer-generated ORTEP drawing of final X-ray model of compound **1**. Color codes: carbon, black; oxygen, red.

**Figure 7:**
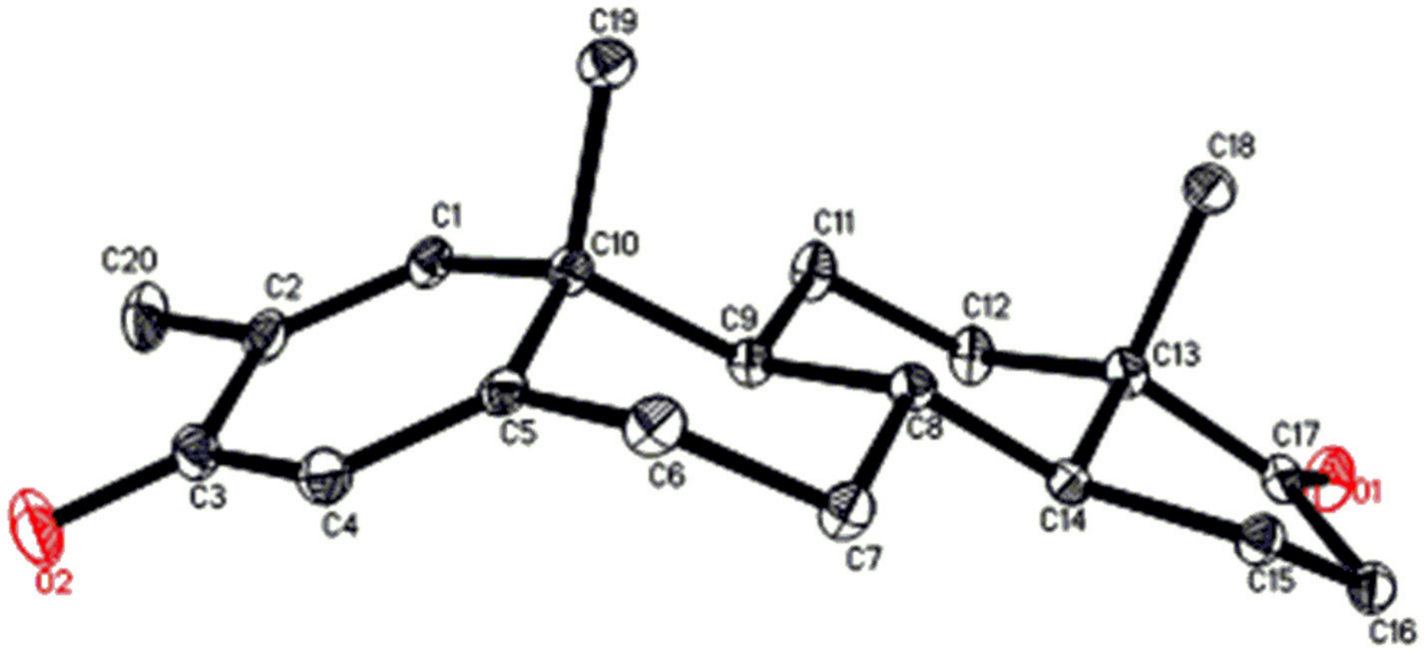
Computer-generated ORTEP drawing of final X-ray model of compound **5**. Color codes: carbon, black; oxygen, red.

Single-crystal X-ray diffraction analyses were also carried out on compounds **1**, and **5**. Compound **1** was comprised of four rings i.e., A, B, C, and D with chair, chair, chair, and envelope conformations. A methyl was present at C-2 with *equatorial* orientation. Heptanoate ester chain was present at C-17 of ring D (Figure 6). Whereas, metabolite **5** contains rings A, B, C, and D in planer, chair, chair, and envelop conformations. Heptanoate side chain was absent in metabolite **5** (Figure 7). Single-crystal diffraction data of metabolites **1**, and **5** were submitted to Cambridge Crystallographic Data Collection (CCDC 1500705, and CCDC 1500707, respectively). Single-crystal X-ray diffraction analysis data of compounds **1**, **2**, and **5** is presented in Table 4.

**Table 4:** Single-crystal X-ray diffraction analysis of compounds **1, 2**, and **5**.

**Crystal Parameters**	**1**	**2**	**5**
Empirical formula	C_27_H_44_O_3_	C_20_H_30_O_3_	C_20_H_26_O_2_
Formula weight	416.62	322.25	298.19
Wavelength	1.54178 Å	1.54178 A	1.54178 Å
Crystal system	Monoclinic	Monoclinic	Monoclinic
Space group	P2_1_	P2_1_	P2_1_
Unit cell dimensions	**a** = 8.8865(6), Å α = 90°	**a** = 7.075(3) Å α = 90°	**a** = 9.7946(16) Å α = 90°
	**b** = 6.2961(4) Å β = 90.806(2)°	**b** = 11.934(10) Å β = 98.21(3)	**b** = 12.217(2) Å β = 92.175(14)°
	**c** = 21.8704(13) Å γ = 90°	**c** = 10.539(6) Å γ = 90°	**c** = 13.629(3) Å γ = 90°
Volume	1223.53(13) Å^3^	880.8(9) Å^3^	1629.7(6) Å^3^
Z	2 mg/m^3^	3 mg/m^3^	2 mg/m^3^
Calculated density	1.131 mg/m^3^	1.216 mg/m^3^	1.216 mg/m^3^
Absorption coefficient	0.551 mm^−1^	0.620 mm^−1^	0.595 mm^−1^
F(000)	460	356	648
Crystal size	0.34 × 0.14 × 0.13 mm	0.32 × 0.13 × 0.11 mm	0.22 × 0.17 × 0.10 mm
θ Range for data collection	4.04 to 42.11°	4.24 to 49.22°	3.24 to 59.05°
Reflections collected/unique	5,163/1,679 [R(int) = 0.0562]	5,713/1,719 [R(int) = 0.0454]	1,6507/2,465 [R(int) = 0.0333]
Goodness-of-fit on F^2^	1.101	1.049	1.051
Final R indices [I>2δ (I)]	R_1_ = 0.0438, wR_2_ = 0.1060	R_1_ = 0.0396, wR_2_ = 0.0987	R_1_ = 0.0268, wR_2_ = 0.0706
R indices (all data)	R_1_ = 0.0475, wR_2_ = 0.1087	R_1_ = 0.0434, wR_2_ = 0.1013	R_1_ = 0.0288, wR_2_ = 0.0721
Largest diff. peak and hole	0.190 and −0.270 e.A^−3^	0.125 and −0.151 e.A^−3^	0.139 and −0.170 e.A^−3^

### Cytotoxicity against HeLa, PC3, H640, and HCT116 cancer, and 3T3 normal cell lines

Natural products, e.g., flavonoids, steroids, terpenes, etc. can serve as an excellent source for the production of many drugs, as they are reported for many biological activities such as anti-cancer, anti-inflammatory, anti-leishmanial, anti-bacterial activities, etc. Therefore, it is necessary to derivatize them, to study structure-activity relationship which might be helpful for the synthesis of safe and effective drugs. At present, many pharmaceutical products are efficiently synthesized *via* microbial biotransformation due to presence of a range of enzymes in them. Based on reported anti-cancer activity of drostanolone (**9**), drostanolone propionate, and drostanolone heptanoate (**1**), compounds **1**–**9** were evaluated for cytotoxicity against HeLa, PC-3, H460, and HCT116 cancer cell lines. Interestingly, all compounds showed a moderate to potent anti-cancer activity. The anti-cancer activity of compounds **1**–**9** were inferred from the IC_50_ values against HeLa (human cervical carcinoma), PC-3 (human prostate cancer), H460 (human lung cancer), and HCT116 (human colon cancer) (Table 5). Based on these results, the cytotoxicity of these compounds was also evaluated against 3T3 (mouse fibroblast) normal cell line, where all compounds, except **8** (IC_50_ = 74.6 ± 3.7 μM), and **9** (IC_50_ = 62.1 ± 1.2 μM), were found to be non-cytotoxic.

**Table 5:** Cytotoxicity of compounds **1–9** against HeLa, PC3, H460, and HCT116 cancer, and 3T3 normal cell lines.

**Compounds**	**HeLa cell line (Cervical cancer) IC_50_ ± SD [μM]**	**PC3 cell line (Prostate cancer) IC_50_ ± SD [μM]**	**H460 cell line (Lung cancer) IC_50_ ± SD [μM]**	**HCT116 cell line (Colon cancer) IC_50_ ± SD [μM]**	**3T3 cell line (Mouse fibroblast) IC_50_ ± SD [μM]**
1	54.7 ± 1.6	96.2 ± 3.0	5.0 ± 1.2	3.1 ± 3.2	>150
2	64.3 ± 3.0	84.6 ± 6.4	44.4 ± 2.0	39.4 ± 2.0	>150
3	58.0 ± 1.0	68.1 ± 1.2	16.7 ± 2.6	42.8 ± 1.2	>150
4	49.5 ± 2.2	60.4 ± 0.9	12.4 ± 2.3	80.9 ± 1.6	>150
5	39.8 ± 1.5	84.0 ± 3.1	31.9 ± 1. 8	30.4 ± 1.6	>150
6	40.7 ± 0.9	58.4 ± 1.6	33.2 ± 1.0	45.9 ± 4.2	>150
7	43.9 ± 2.4	59.1 ± 2.6	38.5 ± 2.8	46.6 ± 3.0	>150
8	19.6 ± 1.4	51.8 ± 3.4	26.4 ± 0.9	55.0 ± 1.9	74.6 ± 3.7
9	25.1 ± 1.6	57.8 ± 3.2	31.8 ± 1.2	2.8 ± 0.2	62.1 ± 1.2
Standard drug, cisplatin	40.1 ± 2.0	76.5 ± 1.2	22.2 ± 2.1	11.2 ± 3.03	42.7 ± 0.8

### Structure-activity relationship

Variations in the structures of metabolites effected their anti-cancer activity. Metabolite **2** with the absence of ester moiety, and two αO-H groups at C-3, and C-14, and a βO-H group at C-17 showed a lower cytotoxicity against HeLa, H460, and HCT116 cancer cell lines, but its anti-cancer activity was higher against PC-3 cancer cell line, as compared to compound **1**. Metabolite **3** with the loss of ester group and two keto groups at C-3, and C-17, and αO-H group at C-7 also showed a lower activity against HeLa, H460, and HCT116 cancer cell lines, but increased activity against PC-3 cancer cell line in comparison to substrate **1**. Reduction of C-3 carbonyl to O-H and hydrolytic cleavage of ester moiety in compound **4** increased its activity against both HeLa and PC-3 cancer cell lines to some extent, but its anti-cancer activity against H460, and HCT116 cancer cell lines decreased as compared to substrate **1**. Presence of C = C between C-1/C-2 and C-4/C-5, along with C = O at C-3, and C-17 made metabolite **5** a potent anti-cancer agent against HeLa cancer cell line, as compared to substrate **1** and the standard drug (cisplatin). Moreover, its anti-cancer activity against PC-3 cancer cell line was also increased as compared to substrate **1**. Interestingly, its anti-cancer activity against H460, and HCT116 cancer cell lines was lower in comparison to compound **1**. Presence of C = C between C-1/C-2 and C-4/C-5, C = O at C-3, and C-17, along with αO-H group at C-11 in metabolite **6** increased its anti-cancer activity against HeLa, and PC-3 cancer cell lines in comparison to substrate **1** and showed similar anti-cancer potential against HeLa cancer cell line as standard drug (cisplatin). However, its anti-cancer activity against H460, and HCT116 cancer cell lines decreased in comparison to compound **1**. Presence of C = C between C-1/C-2 and C-4/C-5, C = O at C-3, and C-17, along with αO-H group at C-14 in metabolite **7** again increased its anti-cancer activity against HeLa, and PC-3 cancer cell lines, and decreased its anti-cancer activity against H460, and HCT116 cell lines in comparison to substrate **1**. Presence of C = C at C-1/C-2, C = O at C-3, and C-17, and αOH at C-7 made metabolite **8** the most potent anti-cancer against HeLa cancer cell line in comparison to substrate **1** and the standard drug (cisplatin). It also showed a good activity against PC-3 cancer cell line but decreased anti-cancer activity against H460, and HCT116 cancer cell lines as compared to substrate **1**. Metabolite **9** with only hydrolysis at C-17 showed increased anti-cancer activity against HeLa, PC-3, and HCT116 cancer cell lines but decreased cytotoxicity against H460 cancer cell line in comparison to substrate **1** and the standard drug (cisplatin). On the basis of above discussion, it can be concluded that increased anti-cancer activity of compounds **5**–**8** against HeLa, and PC-3 cancer cell lines was probably due to the presence of C = C at C-1/C-2, and carbonyl ketone at C-3, and C-17 in comparison to substrate **1**.

## Conclusion

In conclusion, the microbial transformation of anabolic-androgenic steroid drostanolone heptanoate (**1**) with *C. aphidicola* and *F. lini* led to the synthesis of eight metabolites, including five new metabolites **2**, **3**, **6**, **7**, and **8**. Hydroxylation, oxidative cleavage of ester moiety, reduction, and dehydrogenation were the main reactions observed during the transformation. Compounds **1**–**9** exhibited anti-cancer potential against HeLa (human cervical carcinoma), PC-3 (human prostate carcinoma), H460 (human lung cancer), and HCT116 (human colon cancer) cancer cell lines between moderate to potent range. Interestingly, substrate **1** (IC_50_ = 3.1 ± 3.2 μM) and metabolite **9** (IC_50_ = 2.8 ± 0.2 μM) were found to be more active against HCT116 cancer cell line than the standard drug, cisplatin (IC_50_ = 11.2 ± 3.0 μM). Compounds **1** (IC_50_ = 5.0 ± 1.2 μM), **3** (IC_50_ = 16.7 ± 2.6 μM), and **4** (IC_50_ = 12.4 ± 2.3 μM) were found to be more active against H460 cancer cell line than the standard drug, cisplatin (IC_50_ = 22.2 ± 2.1 μM). Metabolites **5** (IC_50_ = 39.8 ± 1.5 μM), **8** (IC_50_ = 19.6 ± 1.4 μM), and **9** (IC_50_ = 25.1 ± 1.6 μM) were also found more active against HeLa cancer cell line than the standard drug, cisplatin (IC_50_ = 40.1 ± 2.0 μM). Metabolites **3** (IC_50_ = 68.0 ± 1.2 μM), **4** (IC_50_ = 60.4 ± 0.9 μM), **6** (IC_50_ = 58.4 ± 1.6 μM), **7** (IC_50_ = 59.1 ± 2.6 μM), **8** (IC_50_ = 51.8 ± 3.4 μM), and **9** (IC_50_ = 57.8 ± 3.2 μM) were also found more active against PC-3 cancer cell line than the standard drug, cisplatin (IC_50_ = 76.5 ± 1.2 μM). Except compounds **8** and **9**, all compounds were found to be non-cytotoxic to normal 3T3 cell line. These results indicated specific cytotoxicity of this class of compounds against cancer cell lines, as compared to normal cell line. Thus, the results of presented study will be helpful towards the drug discovery against cervical, prostate, lung, and colon cancers.

## Author contributions

The concept of presented research was developed and designed by MIC. MIC was also the project supervisor. All the data were analyzed and interpreted by MIC, A-t-W, and HC. MS, SY, MA, and NF performed the experiments. MIC, A-t-W, and MS wrote the manuscript. All the authors revised the manuscript. The final version of manuscript was also approved by MIC before submission.

### Conflict of interest statement

The authors declare that the research was conducted in the absence of any commercial or financial relationships that could be construed as a potential conflict of interest.
