# Supplementary material for: Bio-Catalytic Structural Transformation of Anti-cancer Steroid, Drostanolone Enanthate with Cephalosporium aphidicola and Fusarium lini, and Cytotoxic Potential Evaluation of Its Metabolites against Certain Cancer Cell Lines
Source: Front Pharmacol. 2017 Dec 20;8:900. doi: 10.3389/fphar.2017.00900 (PMC5742531; doi:10.3389/fphar.2017.00900)
Supplement: Supplementary file 1 [file DataSheet1.PDF]

File: DRO-01 Date Run: 02-24-2015 (Time Run: 16:13:04)
Sample: MAHWISH SIDDIQUI /DR. M. IQBAL
Instrument: JEOL JMS-600H Run By: HEJ (ICCBS]
Inlet: Direct Probe Ionization mode: EI+

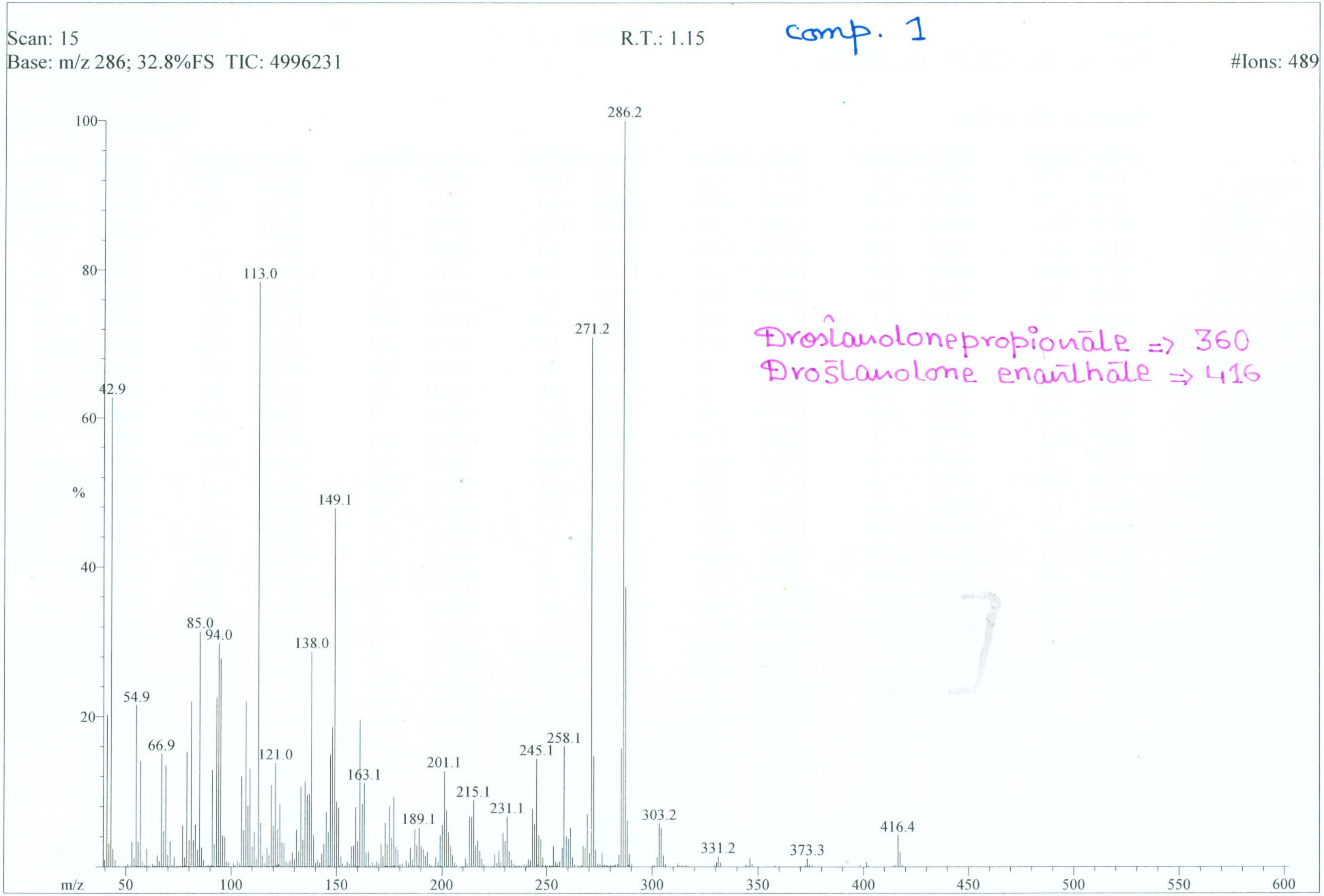

JEOL HX 110 MASS SPECTROMETER (EI-HR)

STUDENT NAME	MAHWISH	SAMPLE CODE	DATED
SUPERVISOR NAME	Dr. IQBAL	DRO-01	23-11-17

comp - 1

Mass	Theoretical Mass	Delta [ppm]	Delta [mmu]	RDB	Composition
416.3311	416.3290	4.9	2.1	6.0	$C_{27} H_{44} O_{3}$
401.3045	401.3056	-2.7	-1.1	6.5	$C_{26} H_{41} O_{3}$
286.2255	286.2297	-14.6	-4.2	6.0	$C_{20} H_{30} O_{1}$
271.2051	271.2062	-4.0	-1.1	6.5	$C_{19} H_{27} O_{1}$

AVANCE AV-400 MHz
Lab # 115

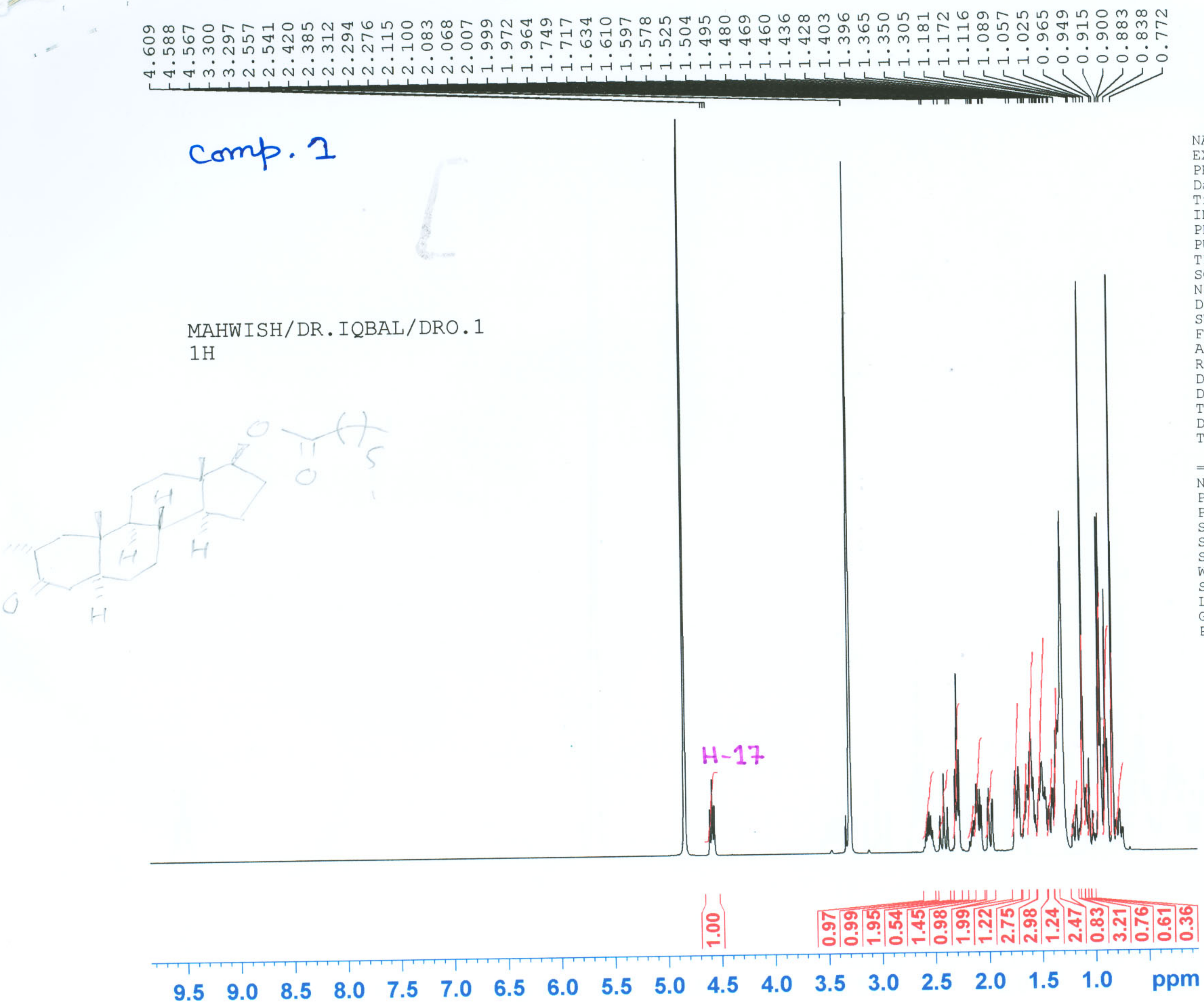

NAME	feb25-15
EXPNO	11
PROCNO	1
Date_	20150225
Time	13.16
INSTRUM	spect
PROBHD	5 mm SEI 1H-13
PULPROG	zg30
TD	32768
SOLVENT	MeOD
NS	128
DS	0
SWH	8012.820 Hz
FIDRES	0.244532 Hz
AQ	2.0447731 sec
RG	161.3
DW	62.400 usec
DE	6.50 usec
TE	300.0 K
D1	1.50000000 sec
TD0	1

======== CHANNEL f1 ========

NUC1	1H
P1	10.80 usec
PL1	3.00 dB
SFO1	400.0332002 MHz
SI	16384
SF	400.0300087 MHz
WDW	EM
SSB	0
LB	0.30 Hz
GB	0
PC	1.00

Mahwish / Dr. Iqbal / Dros-01
BB

comp. 1

AVANCE AV-III HD
400 MHz
LAB #109A

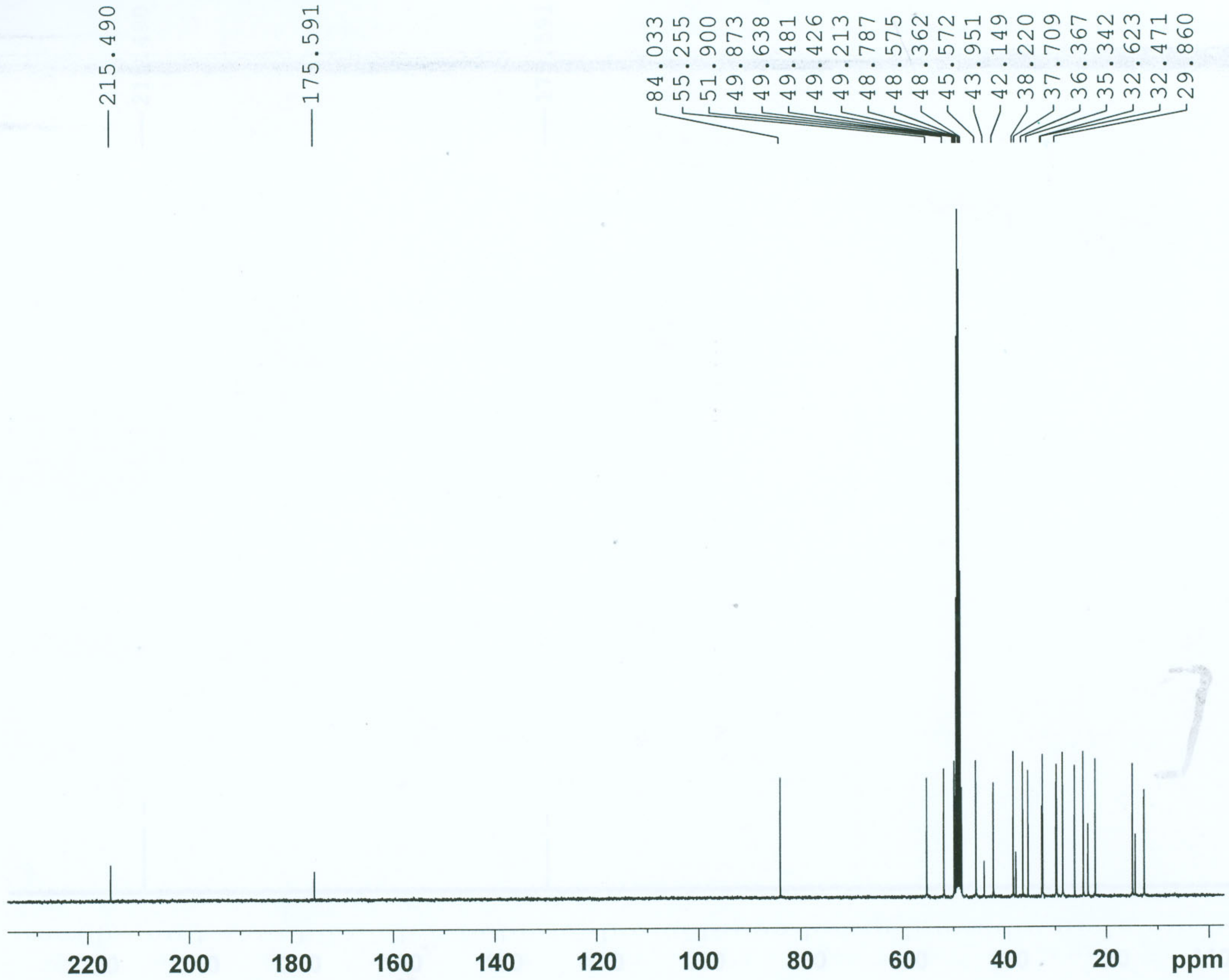


```
Current Data Parameters
NAME              apr08-15
EXPNO                    6
PROCNO                   1

F2 - Acquisition Parameters
Date_             20150409
Time                 10.59
INSTRUM              spect
PROBHD    5 mm SEI 1H/D-
PULPROG               zgpg
TD                   32768
SOLVENT               MeOD
NS                    6755
DS                       4
SWH              24038.461 Hz
FIDRES            0.733596 Hz
AQ               0.6815744 sec
RG                  202.75
DW                  20.800 usec
DE                    6.50 usec
TE                   299.0 K
D1              1.50000000 sec
D11             0.03000000 sec
TD0                     20

======== CHANNEL f1 ========
SFO1           100.6746353 MHz
NUC1                   13C
P1                   11.00 usec
PLW1          150.00000000 W

======== CHANNEL f2 ========
SFO2           400.3316013 MHz
NUC2                    1H
CPDPRG[2           waltz16
PCPD2                90.00 usec
PLW2           20.00000000 W
PLW12           0.08984600 W
PLW13           0.07277500 W

F2 - Processing parameters
SI                   32768
SF             100.6629176 MHz
WDW                     EM
SSB      0
LB                    1.00 Hz
GB       0
PC                    1.40
```

Mahwish / Dr. Iqbal / Dros-01
Dept90

Comp. 1

AVANCE AV-III HD
400 MHz
LAB #109A

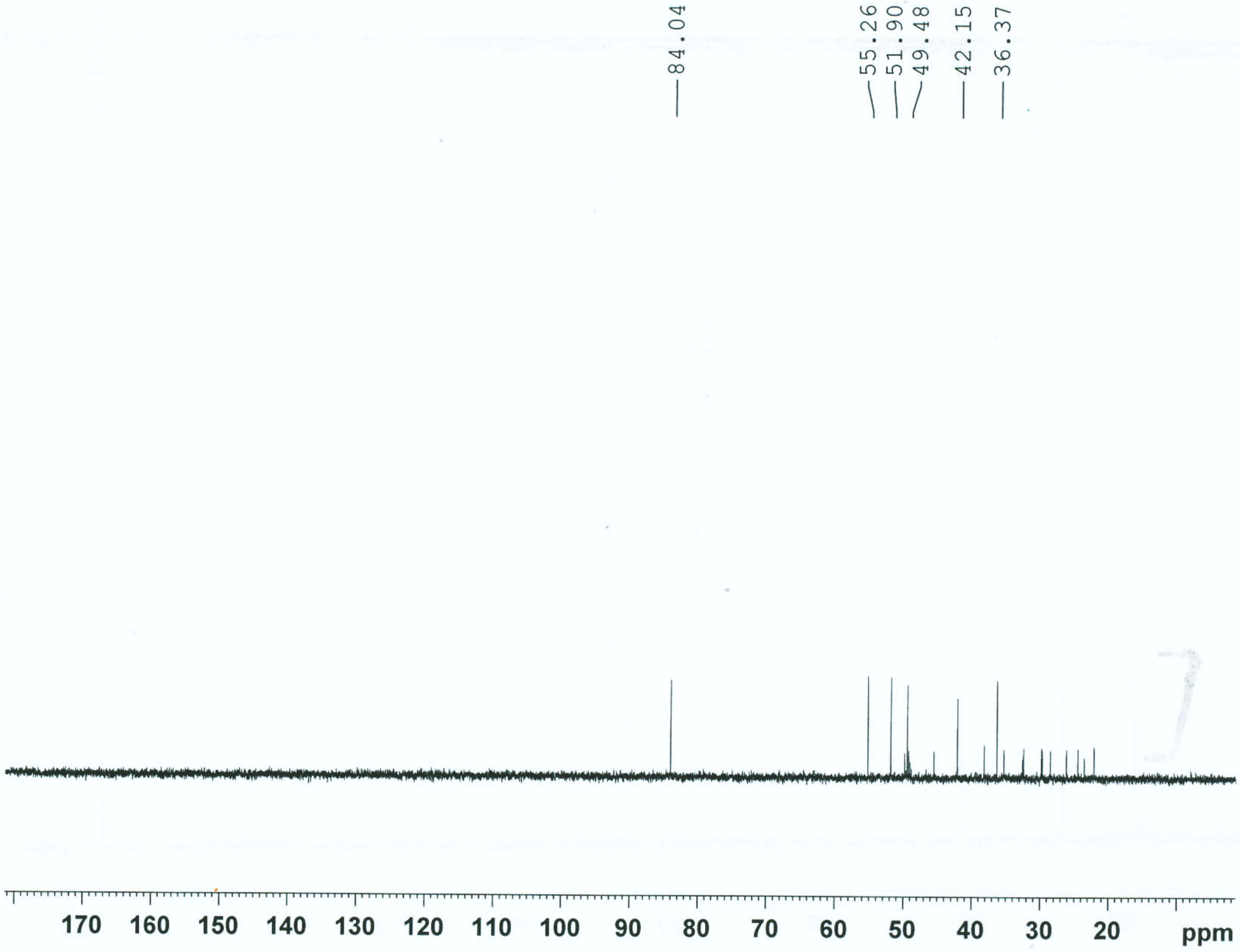

Current Data Parameters
NAME apr08-15
EXPNO 8
PROCNO 1

F2 - Acquisition Parameters
Date_ 20150409
Time 15.53
INSTRUM spect
PROBHD 5 mm SEI 1H/D-
PULPROG deptsp90
TD 32768
SOLVENT MeOD
NS 228
DS 4
SWH 18115.941 Hz
FIDRES 0.552855 Hz
AQ 0.9043968 sec
RG 202.75
DW 27.600 usec
DE 6.50 usec
TE 300.6 K
CNST2 142.0000000
D1 1.50000000 sec
D2 0.00352113 sec
D12 0.00002000 sec
TD0 4

======== CHANNEL f1 ========
SFO1 100.6721187 MHz
NUC1 13C
P1 11.00 usec
P13 2000.00 usec
PLW0 0 W
PLW1 150.00000000 W
SPNAM[5] Crp60comp.4
SPOAL5 0.500
SPOFFS5 0 Hz
SPW5 27.73100090 W

======== CHANNEL f2 ========
SFO2 400.3316013 MHz
NUC2 1H
CPDPRG[2 waltz16
P3 6.80 usec
P4 13.60 usec
PCPD2 90.00 usec
PLW2 20.00000000 W
PLW12 0.11417000 W

F2 - Processing parameters
SI 32768
SF 100.6629176 MHz
WDW EM
SSB 0
LB 1.00 Hz
GB 0
PC 1.40

Mahwish / Dr. Iqbal / Dros-01
Dept135

AVANCE AV-III HD
400 MHz
LAB #109A

Comp. 1

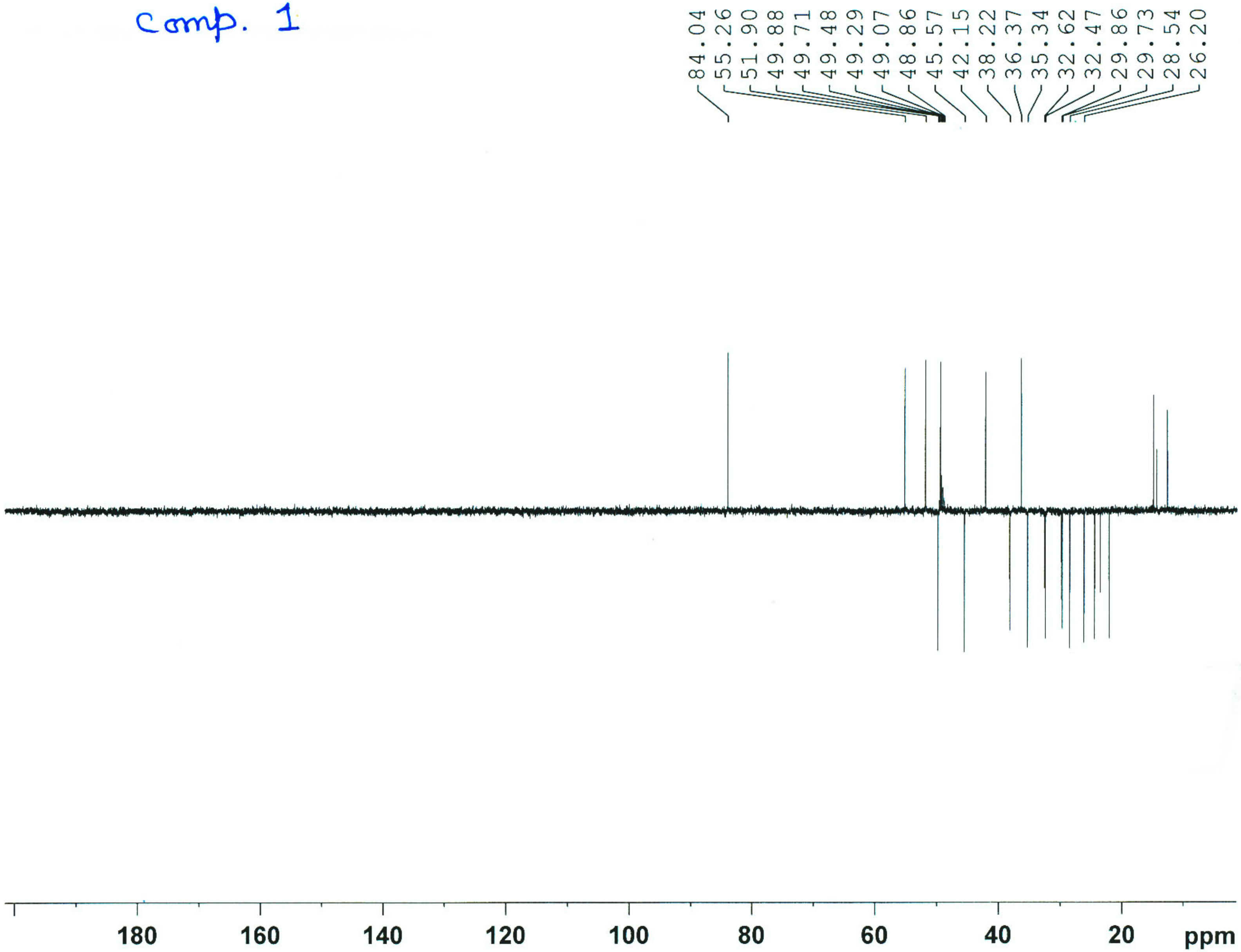

Current Data Parameters

NAME	apr08-15	
EXPNO	7	
PROCNO	1	

F2 - Acquisition Parameters

Date_	20150409	
Time	15.40	
INSTRUM	spect	
PROBHD	5 mm SEI 1H/D-	
PULPROG	deptsp135	
TD	32768	
SOLVENT	MeOD	
NS	1133	
DS	4	
SWH	20161.291	Hz
FIDRES	0.615274	Hz
AQ	0.8126464	sec
RG	202.75	
DW	24.800	usec
DE	6.50	usec
TE	300.8	K
CNST2	145.0000000	
D1	1.50000000	sec
D2	0.00344828	sec
D12	0.00002000	sec
TD0	9	

======== CHANNEL f1 ========

SFO1	100.6731253	MHz
NUC1	13C	
P1	11.00	usec
P13	2000.00	usec
PLW0	0 W	
PLW1	150.00000000	W
SPNAM[5]	Crp60comp.4	
SPOAL5	0.500	
SPOFFS5	0 Hz	
SPW5	27.73100090	W

======== CHANNEL f2 ========

SFO2	400.3316013	MHz
NUC2	1H	
CPDPRG[2	waltz16	
P3	6.80	usec
P4	13.60	usec
PCPD2	90.00	usec
PLW2	20.00000000	W
PLW12	0.11417000	W

F2 - Processing parameters

SI	32768	
SF	100.6629176	MHz
WDW	EM	
SSB	0	
LB	1.00	Hz
GB	0	
PC	1.40	

Mahwish / Dr. Iqbal / Dros-01
HSQC

Comp. I

AVANCE AV-III HD
400 MHz
LAB #109A

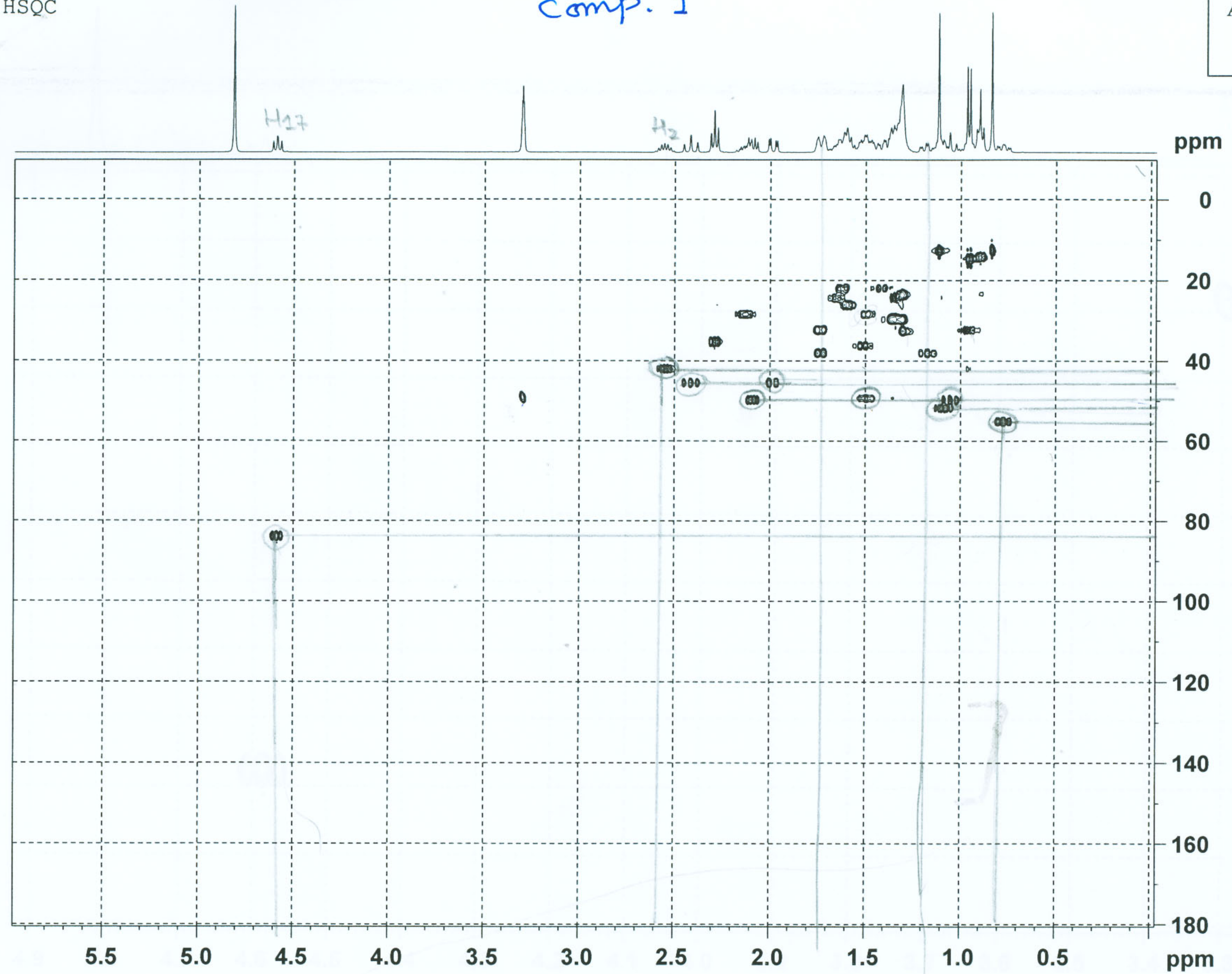

Current Data Parameters

NAME	apr08-15
EXPNO	4
PROCNO	1

F2 - Acquisition Parameters

Date_	20150409
Time	0.42
INSTRUM	spect
PROBHD	5 mm SEI 1H/D-
PULPROG	hsqcetgpsi
TD	2048
SOLVENT	MeOD
NS	64
DS	16
SWH	2400.768 Hz
FIDRES	1.172250 Hz
AQ	0.4265301 sec
RG	202.75
DW	208.267 usec
DE	6.50 usec
TE	299.5 K
CNST2	145.0000000
D0	0.00000300 sec
D1	1.50000000 sec
D4	0.00172414 sec
D11	0.03000000 sec
D16	0.00020000 sec
D24	0.00089000 sec
IN0	0.00002610 sec
ZGOPTNS	

======== CHANNEL f1 ========

SFO1	400.3312010 MHz
NUC1	1H
P1	6.60 usec
P2	13.20 usec
P28	1000.00 usec
PLW1	20.00000000 W

======== CHANNEL f2 ========

SFO2	100.6715147 MHz
NUC2	13C
CPDPRG[2	garp
P3	11.00 usec
P4	22.00 usec
PCPD2	84.00 usec
PLW2	150.00000000 W
PLW12	2.57229996 W

====== GRADIENT CHANNEL =====

GPNAM[1]	SMSQ10.100
GPNAM[2]	SMSQ10.100
GPZ1	80.00 %
GPZ2	20.10 %
P16	1000.00 usec

F1 - Acquisition parameters

TD	128
SFO1	100.6715 MHz
FIDRES	149.664749 Hz
SW	190.293 ppm
FnMODE	Echo-Antiecho

F2 - Processing parameters

SI	1024
SF	400.3300116 MHz
WDW	QSINE
SSB	2
LB	0 Hz
GB	0
PC	1.40

F1 - Processing parameters

SI	1024
MC2	echo-antiecho
SF	100.6629176 MHz
WDW	QSINE
SSB	2
LB	0 Hz
GB	0

Mahwish / Dr. Iqbal / Dros-01
HMBC

comp. 1

AVANCE AV-III HD
400 MHz
LAB #109A

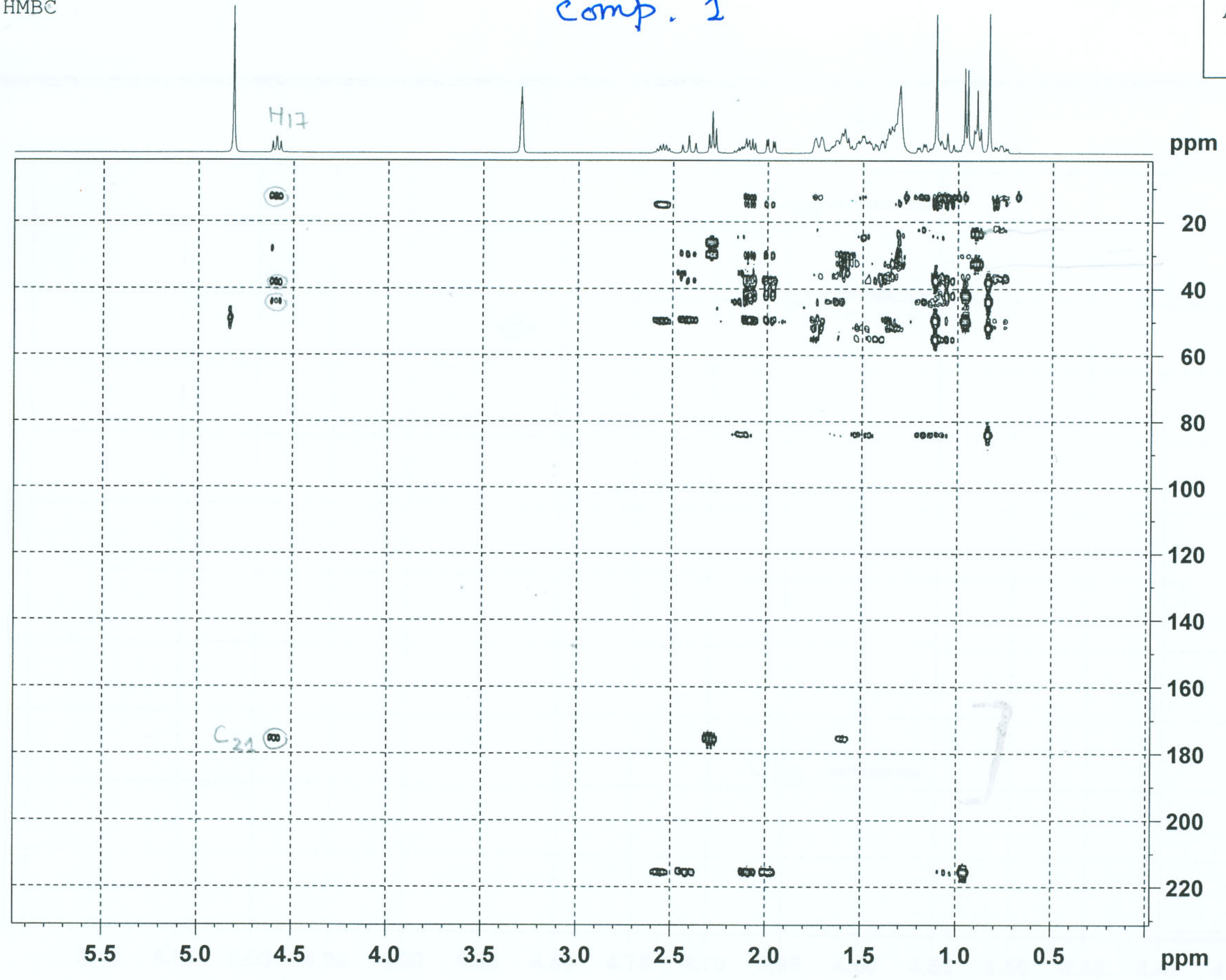


```
Current Data Parameters
NAME              apr08-15
EXPNO                    5
PROCNO                   1

F2 - Acquisition Parameters
Date_             20150409
Time                  5.09
INSTRUM              spect
PROBHD     5 mm SEI 1H/D-
PULPROG       hmbcgplpndqf
TD                    2048
SOLVENT               MeOD
NS                      64
DS                      16
SWH               2400.768 Hz
FIDRES            1.172250 Hz
AQ               0.4265301 sec
RG                  202.75
DW                 208.267 usec
DE                    6.50 usec
TE                   299.2 K
CNST2          145.0000000
CNST13          10.0000000
D0              0.00000300 sec
D1              2.00000000 sec
D2              0.00344828 sec
D6              0.05000000 sec
D16             0.00020000 sec
IN0             0.00002160 sec

======== CHANNEL f1 ========
SFO1           400.3312010 MHz
NUC1                    1H
P1                    6.70 usec
P2                   13.40 usec
PLW1           20.00000000 W

======== CHANNEL f2 ========
SFO2           100.6746353 MHz
NUC2                   13C
P3                   11.00 usec
PLW2          150.00000000 W

====== GRADIENT CHANNEL =====
GPNAM[1]         SMSQ10.100
GPNAM[2]         SMSQ10.100
GPNAM[3]         SMSQ10.100
GPZ1                 50.00 %
GPZ2                 30.00 %
GPZ3                 40.10 %
P16                1000.00 usec

F1 - Acquisition parameters
TD                     128
SFO1              100.6746 MHz
FIDRES          180.844910 Hz
SW                 229.930 ppm
FnMODE                  QF

F2 - Processing parameters
SI                    2048
SF             400.3300116 MHz
WDW                   SINE
SSB      0
LB       0 Hz
GB       0
PC                    1.40

F1 - Processing parameters
SI                    1024
MC2                     QF
SF             100.6629176 MHz
WDW                   SINE
SSB      0
LB       0 Hz
GB       0
```

Mahwish / Dr. Iqbal / Dros-01
COSY

comp. 1

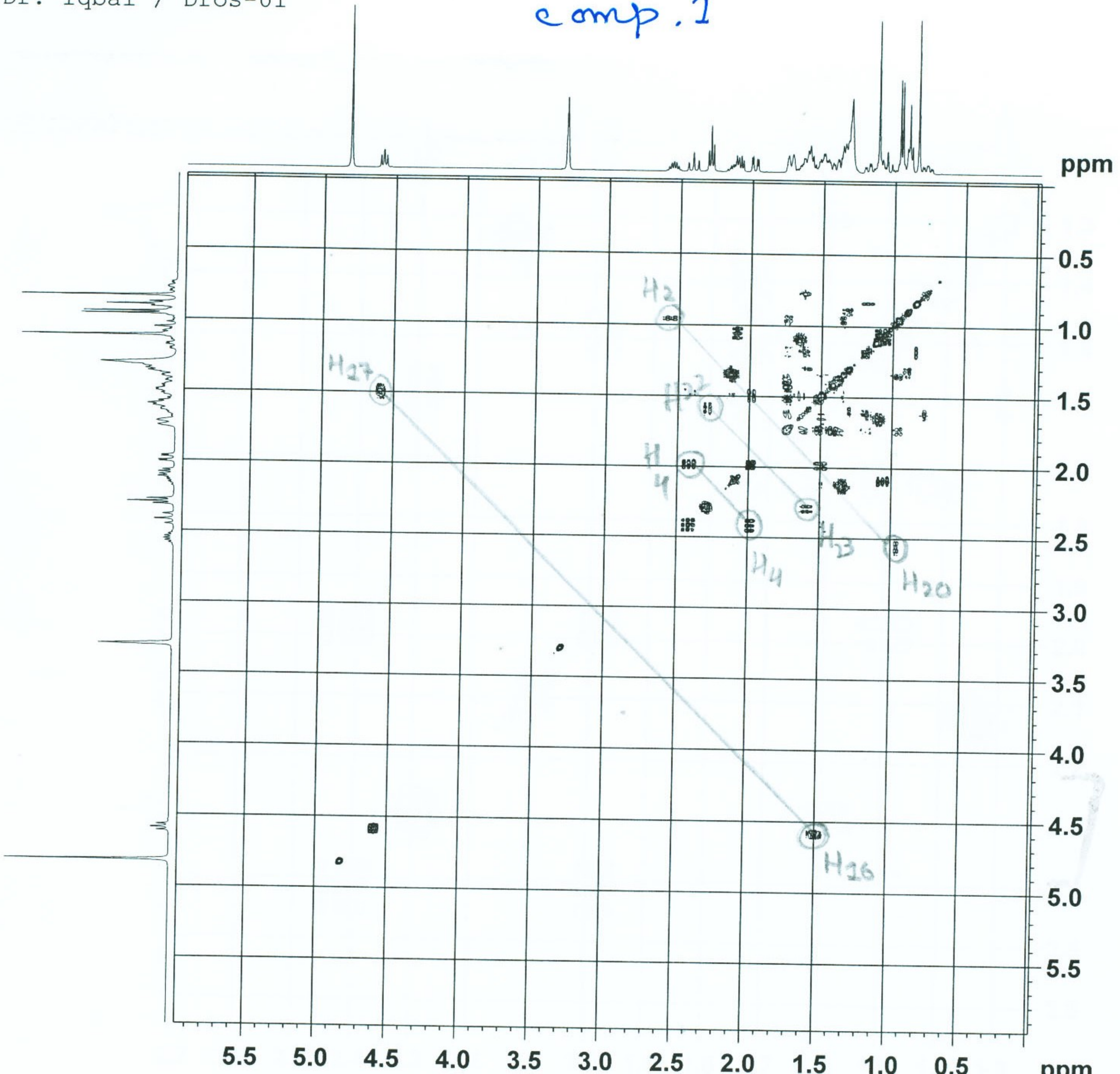

AVANCE AV-III HD
400 MHz
LAB #109A

Current Data Parameters
NAME apr08-15
EXPNO 2
PROCNO 1

F2 - Acquisition Parameters
Date_ 20150408
Time 16.07
INSTRUM spect
PROBHD 5 mm SEI 1H/D-
PULPROG cosygpqf
TD 2048
SOLVENT MeOD
NS 16
DS 2
SWH 2400.768 Hz
FIDRES 1.172250 Hz
AQ 0.4265301 sec
RG 132.58
DW 208.267 usec
DE 6.50 usec
TE 300.4 K
D0 0.00000300 sec
D1 1.50000000 sec
D13 0.00000400 sec
D16 0.00020000 sec
IN0 0.00041660 sec

======== CHANNEL f1 ========
SFO1 400.3312010 MHz
NUC1 1H
P0 6.70 usec
P1 6.70 usec
PLW1 20.00000000 W

====== GRADIENT CHANNEL =====
GPNAM[1] SMSQ10.100
GPZ1 10.00 %
P16 1000.00 usec

F1 - Acquisition parameters
TD 256
SFO1 400.3312 MHz
FIDRES 9.376500 Hz
SW 5.996 ppm
FnMODE QF

F2 - Processing parameters
SI 2048
SF 400.3300116 MHz
WDW QSINE
SSB 0
LB 0 Hz
GB 0
PC 1.40

F1 - Processing parameters
SI 1024
MC2 QF
SF 400.3300116 MHz
WDW QSINE
SSB 0
LB 0 Hz
GB 0

Mahwish / Dr. Iqbal / Dros-01
NOESY

Comp. 1

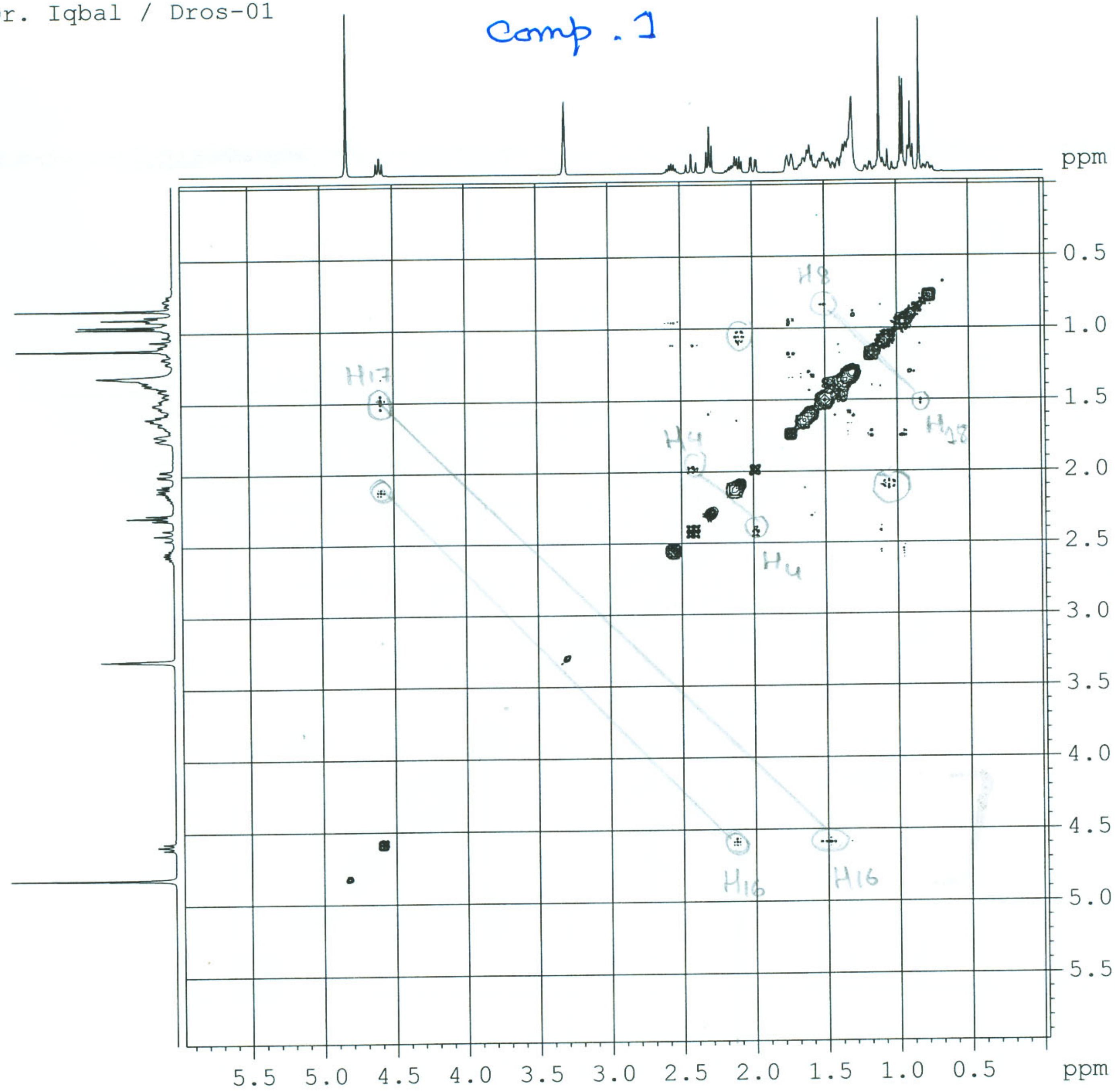

AVANCE AV-III HD
400 MHz
LAB #109A

Current Data Parameters
NAME apr08-15
EXPNO 3
PROCNO 1

F2 - Acquisition Parameters
Date_ 20150408
Time 18.24
INSTRUM spect
PROBHD 5 mm SEI 1H/D-
PULPROG noesygpph
TD 2048
SOLVENT MeOD
NS 32
DS 4
SWH 2400.768 Hz
FIDRES 1.172250 Hz
AQ 0.4265301 sec
RG 116.04
DW 208.267 usec
DE 6.50 usec
TE 299.8 K
D0 0.00019977 sec
D1 2.00000000 sec
D8 0.30000001 sec
D16 0.00020000 sec
IN0 0.00041660 sec

======== CHANNEL f1 ========
SFO1 400.3312010 MHz
NUC1 1H
P1 6.70 usec
P2 13.40 usec
PLW1 20.00000000 W

====== GRADIENT CHANNEL =====
GPNAM[1] SMSQ10.100
GPZ1 40.00 %
P16 1000.00 usec

F1 - Acquisition parameters
TD 256
SFO1 400.3312 MHz
FIDRES 9.376500 Hz
SW 5.996 ppm
FnMODE States-TPPI

F2 - Processing parameters
SI 1024
SF 400.3300116 MHz
WDW QSINE
SSB 2
LB 0 Hz
GB 0
PC 1.40

F1 - Processing parameters
SI 1024
MC2 States-TPPI
SF 400.3300116 MHz
WDW QSINE
SSB 2
LB 0 Hz
GB 0
