# Supplementary material for: Bio-Catalytic Structural Transformation of Anti-cancer Steroid, Drostanolone Enanthate with Cephalosporium aphidicola and Fusarium lini, and Cytotoxic Potential Evaluation of Its Metabolites against Certain Cancer Cell Lines
Source: Front Pharmacol. 2017 Dec 20;8:900. doi: 10.3389/fphar.2017.00900 (PMC5742531; doi:10.3389/fphar.2017.00900)

File: DR-7'
Sample: MAHWISH / DR. IQBAL
Instrument: JEOL MS 600H-1

Date Run: 03-07-2016 (Time Run: 09:29:04)

comp. 2

Ionization mode: EI+

Scan: 11
Base: m/z 289; 91.4%FS TIC: 19320244

R.T.: .88

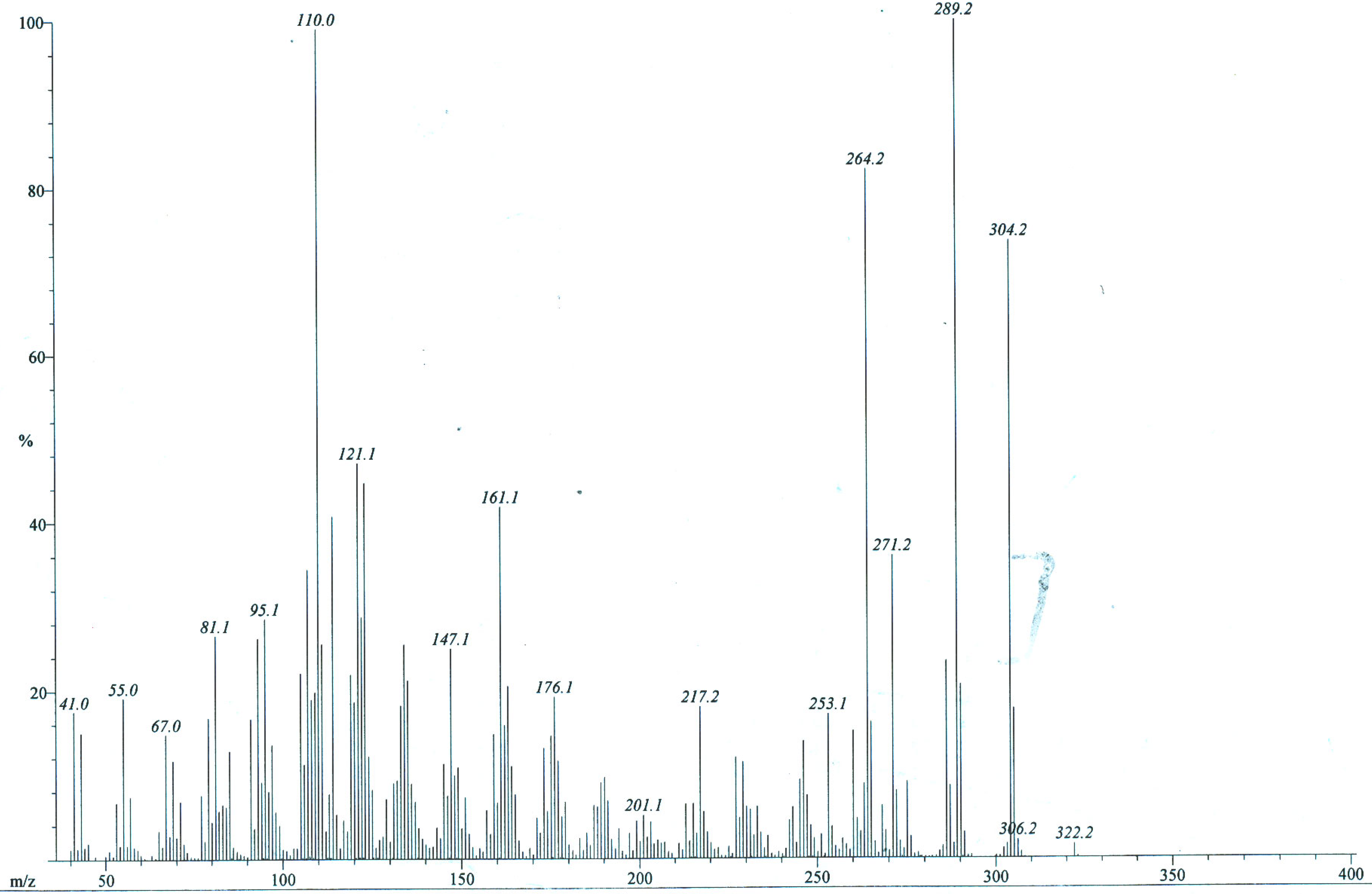

comp. 2

Mass	Relative Intensity	Theoretical Mass	Delta [ppm]	Delta [mmu]	RDB	Composition
		257.1330	25.5	6.6	12.5	$C_{20}H_{17}$
258.1504	1.8	258.1467	14.3	3.7	3.0	$C_{13}H_{22}O_{5}$
		258.1409	37.1	9.6	12.0	$C_{20}H_{18}$
259.1628	1.3	259.1698	-26.9	-7.0	6.5	$C_{17}H_{23}O_{2}$
		259.1545	32.0	8.3	2.5	$C_{13}H_{23}O_{5}$
260.1771	11.1	260.1776	-2.1	-0.6	6.0	$C_{17}H_{24}O_{2}$
261.1828	3.5	261.1855	-10.2	-2.7	5.5	$C_{17}H_{25}O_{2}$
262.1656	2.8	262.1722	-25.2	-6.6	10.0	$C_{20}H_{22}$
		262.1569	33.0	8.7	6.0	$C_{16}H_{22}O_{3}$
263.1776	7.7	263.1800	-9.0	-2.4	9.5	$C_{20}H_{23}$
		263.1858	-31.3	-8.2	0.5	$C_{13}H_{27}O_{5}$
264.1896	58.0	264.1878	6.9	1.8	9.0	$C_{20}H_{24}$
		264.1937	-15.3	-4.0	0.0	$C_{13}H_{28}O_{5}$
265.1971	10.2	265.1956	5.4	1.4	8.5	$C_{20}H_{25}$
266.2000	1.6	266.2035	-13.0	-3.4	8.0	$C_{20}H_{26}$
267.1727	1.0	267.1749	-8.3	-2.2	8.5	$C_{19}H_{23}O_{1}$
267.1952	1.0	267.1960	-2.9	-0.8	3.5	$C_{16}H_{27}O_{3}$
268.2133	6.2	268.2191	-21.7	-5.8	7.0	$C_{20}H_{28}$
		268.2038	35.1	9.4	3.0	$C_{16}H_{28}O_{3}$
269.1919	4.3	269.1905	5.1	1.4	7.5	$C_{19}H_{25}O_{1}$
270.1979	1.5	270.1984	-1.6	-0.4	7.0	$C_{19}H_{26}O_{1}$
271.2038	41.6	271.2062	-8.7	-2.4	6.5	$C_{19}H_{27}O_{1}$
272.2085	9.1	272.2140	-20.1	-5.5	6.0	$C_{19}H_{28}O_{1}$
		272.1988	35.9	9.8	2.0	$C_{15}H_{28}O_{4}$
273.2146	1.9	273.2218	-26.5	-7.2	5.5	$C_{19}H_{29}O_{1}$
		273.2066	29.3	8.0	1.5	$C_{15}H_{29}O_{4}$
274.2266	1.3	274.2297	-11.0	-3.0	5.0	$C_{19}H_{30}O_{1}$
275.2005	7.0	275.2011	-2.1	-0.6	5.5	$C_{18}H_{27}O_{2}$
276.2048	2.4	276.2089	-14.8	-4.1	5.0	$C_{18}H_{28}O_{2}$
277.2115	0.8	277.2168	-18.8	-5.2	4.5	$C_{18}H_{29}O_{2}$
		277.2015	36.2	10.0	0.5	$C_{14}H_{29}O_{5}$
282.1933	0.6	282.1984	-18.0	-5.1	8.0	$C_{20}H_{26}O_{1}$
		282.1831	36.1	10.2	4.0	$C_{16}H_{26}O_{4}$
284.2134	2.0	284.2140	-2.0	-0.6	7.0	$C_{20}H_{28}O_{1}$
285.2011	2.1	285.2066	-19.3	-5.5	2.5	$C_{16}H_{29}O_{4}$
286.2282	25.3	286.2297	-5.0	-1.4	6.0	$C_{20}H_{30}O_{1}$
287.2235	8.2	287.2222	4.5	1.3	1.5	$C_{16}H_{31}O_{4}$
288.2135	1.9	288.2089	15.9	4.6	6.0	$C_{19}H_{28}O_{2}$
289.2145	77.3	289.2168	-7.8	-2.2	5.5	$C_{19}H_{29}O_{2}$
290.2183	15.9	290.2246	-21.6	-6.3	5.0	$C_{19}H_{30}O_{2}$
		290.2093	30.9	9.0	1.0	$C_{15}H_{30}O_{5}$
291.2228	1.9	291.2171	19.6	5.7	0.5	$C_{15}H_{31}O_{5}$
		291.2324	-32.8	-9.6	4.5	$C_{19}H_{31}O_{2}$
		291.2113	39.7	11.6	9.5	$C_{22}H_{27}$
298.1953	0.8	298.1933	6.9	2.1	8.0	$C_{20}H_{26}O_{2}$
300.2079	1.9	300.2089	-3.4	-1.0	7.0	$C_{20}H_{28}O_{2}$
301.2054	0.8	301.2015	13.0	3.9	2.5	$C_{16}H_{29}O_{5}$
		301.1956	32.5	9.8	11.5	$C_{23}H_{25}$
		301.2168	-37.6	-11.3	6.5	$C_{20}H_{29}O_{2}$
302.2253	2.9	302.2246	2.5	0.7	6.0	$C_{20}H_{30}O_{2}$
303.2270	1.9	303.2324	-17.7	-5.4	5.5	$C_{20}H_{31}O_{2}$
		303.2171	32.6	9.9	1.5	$C_{16}H_{31}O_{5}$
304.2407	46.5	304.2402	1.6	0.5	5.0	$C_{20}H_{32}O_{2}$
305.2444	10.2	305.2481	-12.1	-3.7	4.5	$C_{20}H_{33}O_{2}$
		305.2328	37.9	11.6	0.5	$C_{16}H_{33}O_{5}$
306.2459	1.4	306.2406	17.1	5.2	0.0	$C_{16}H_{34}O_{5}$
		306.2559	-32.7	-10.0	4.0	$C_{20}H_{34}O_{2}$
		306.2348	36.3	11.1	9.0	$C_{23}H_{30}$
307.2323	0.5	307.2273	16.2	5.0	4.5	$C_{19}H_{31}O_{3}$
		307.2426	-33.4	-10.3	8.5	$C_{23}H_{31}$
316.2061	0.9	316.2038	7.2	2.3	7.0	$C_{20}H_{28}O_{3}$
318.2224	2.7	318.2195	9.0	2.9	6.0	$C_{20}H_{30}O_{3}$
		318.2348	-38.9	-12.4	10.0	$C_{24}H_{30}$
319.2233	0.7	319.2273	-12.6	-4.0	5.5	$C_{20}H_{31}O_{3}$
		319.2121	35.2	11.2	1.5	$C_{16}H_{31}O_{6}$
320.2381	2.6	320.2351	9.3	3.0	5.0	$C_{20}H_{32}O_{3}$
		320.2504	-38.4	-12.3	9.0	$C_{24}H_{32}$
321.2407	0.6	321.2430	-7.2	-2.3	4.5	$C_{20}H_{33}O_{3}$
322.2517	0.8	322.2508	2.8	0.9	4.0	$C_{20}H_{34}O_{3}$

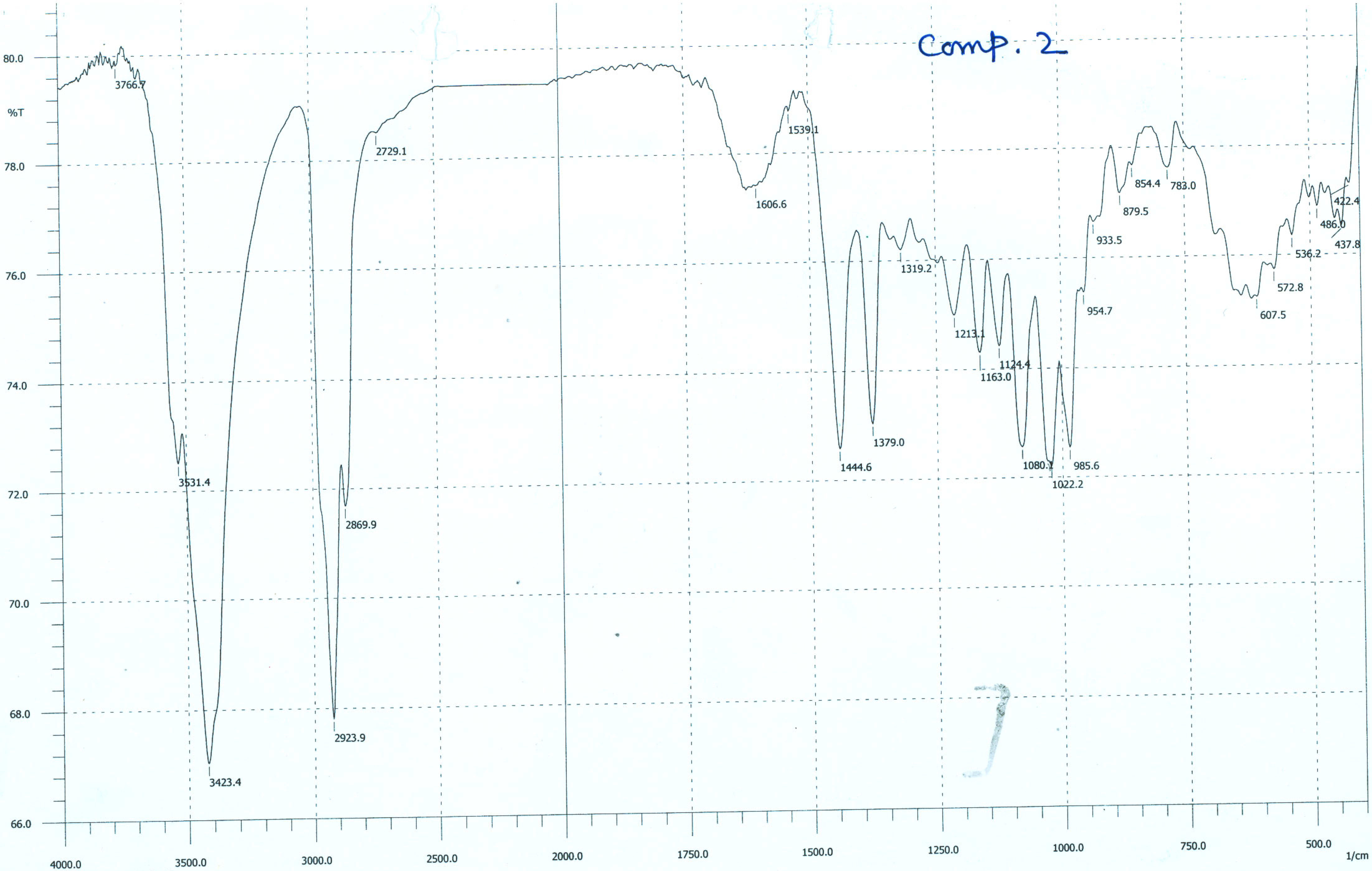

DR-7.IRS: dr-7/Mahwish

Date:	10/12/2015	Time:	11:33:16	NScans:	5
Type:	HYPER IR	User:	Zubair Ahmed	Detector:	standard
Abscissa:	1/cm	Ordinate:	%T	Apodization:	Happ
Min:	401.17	Max:	3998.16	Range:	1/cm
Ndp:	1866	Data Interval:	1.92868	Resolution:	4.0
Gain:	auto	Aperture:	auto	Mirror Speed:	2.8(low)

Comp. 2

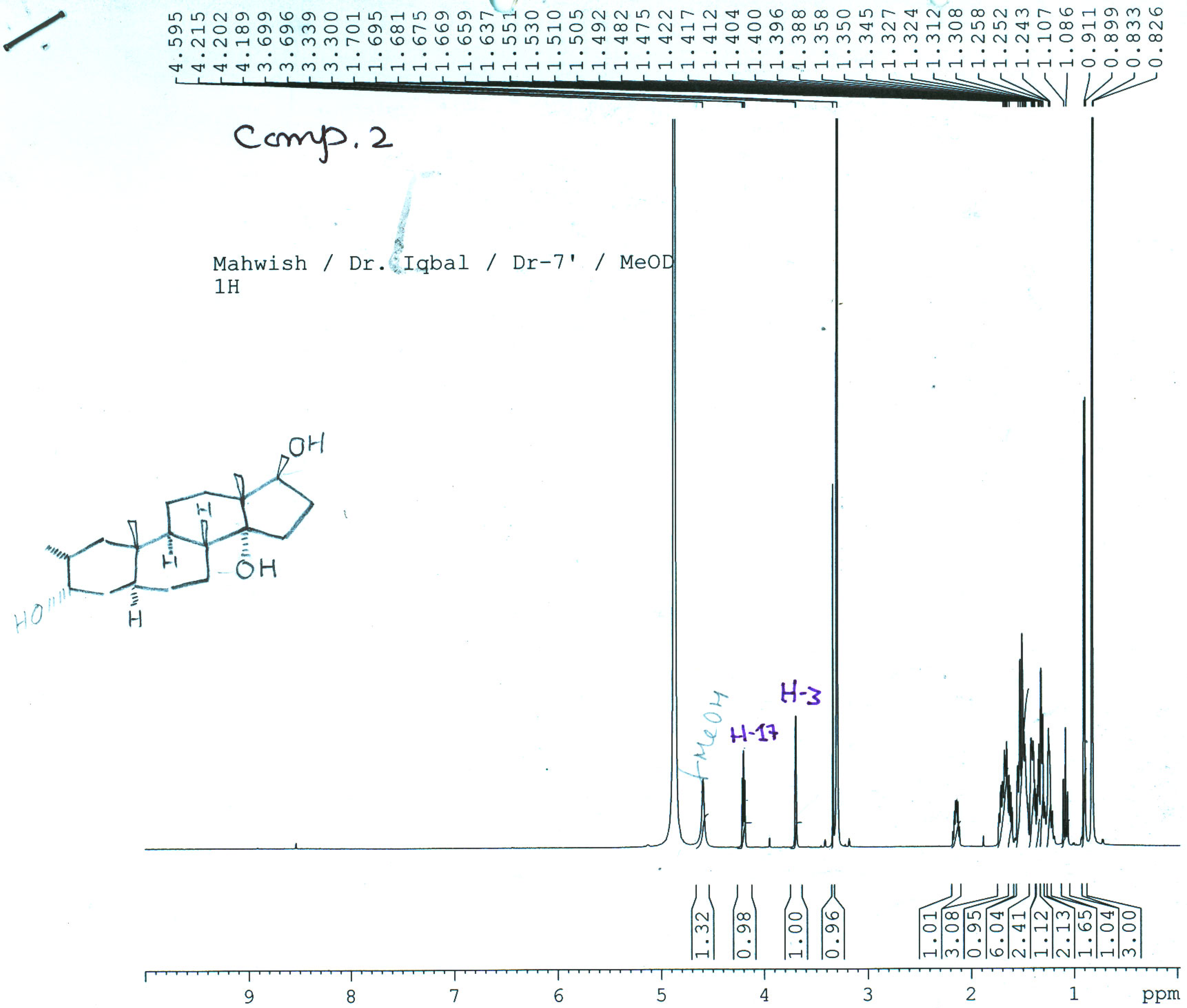

NAME	sep30-15
EXPNO	10
PROCNO	1
Date_	20150930
Time	16.03
INSTRUM	spect
PROBHD	5 mm CPTCI 1H-
PULPROG	zg30
TD	32768
SOLVENT	MeOD
NS	32
DS	0
SWH	12019.230 Hz
FIDRES	0.366798 Hz
AQ	1.3632404 sec
RG	9
DW	41.600 usec
DE	6.50 usec
TE	298.0 K
D1	2.00000000 sec
TD0	1

======== CHANNEL f1 ========

NUC1	1H
P1	8.00 usec
PL1	3.31 dB
PL1W	6.79873323 W
SFO1	600.0348002 MHz
SI	32768
SF	600.0300173 MHz
WDW	EM
SSB	0
LB	0.50 Hz
GB	0
PC	1.40

Mahwish / Dr. Iqbal / Dr-7 / MeOD
BB

Comp. 2

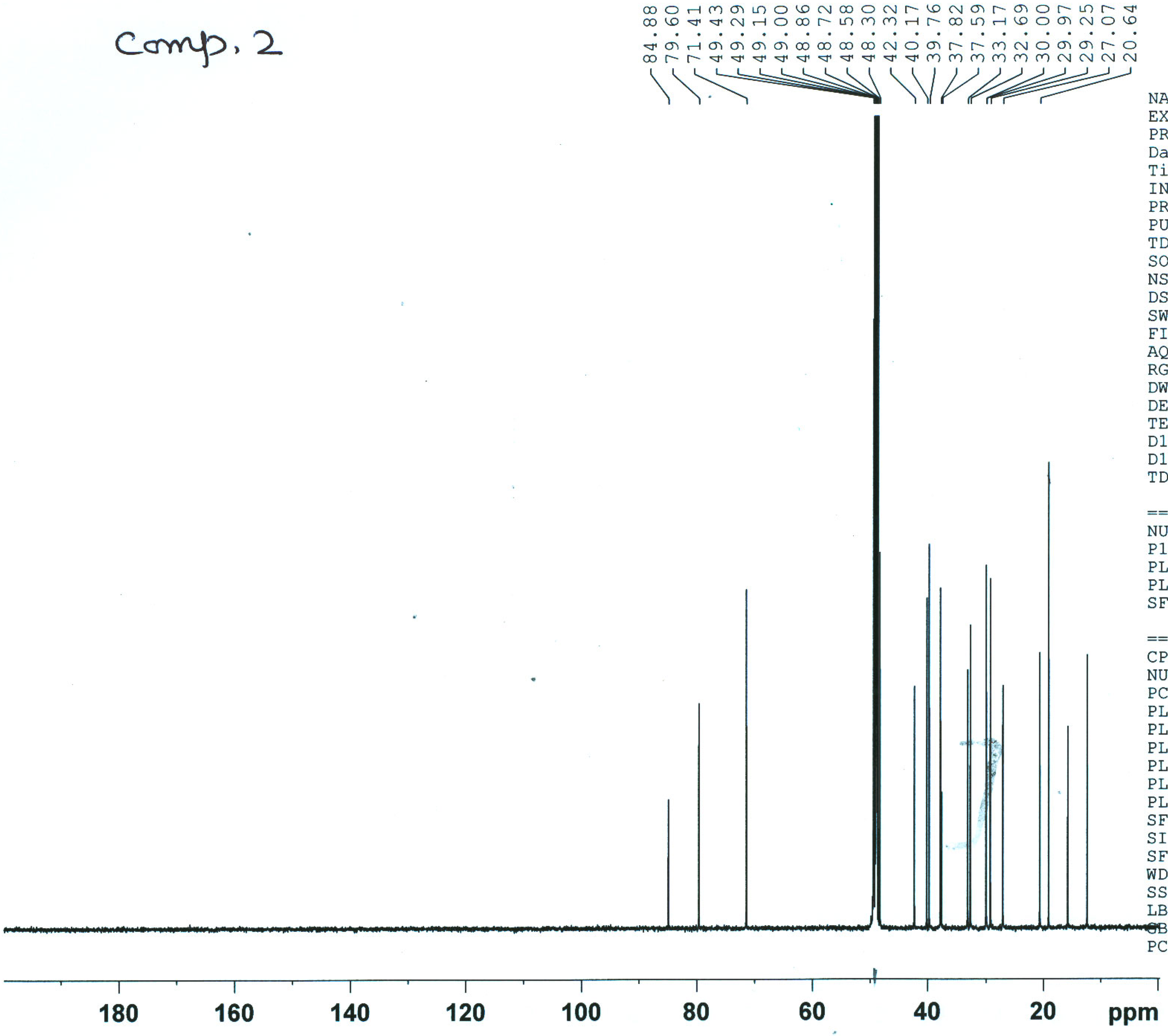

NAME	sep30-15
EXPNO	7
PROCNO	1
Date_	20151001
Time	7.46
INSTRUM	spect
PROBHD	5 mm CPTCI 1H-
PULPROG	zgpg
TD	32768
SOLVENT	MeOD
NS	2172
DS	4
SWH	35971.223 Hz
FIDRES	1.097755 Hz
AQ	0.4555391 sec
RG	32768
DW	13.900 usec
DE	6.50 usec
TE	289.4 K
D1	2.00000000 sec
D11	0.03000000 sec
TD0	14
======== CHANNEL f1 ========	
NUC1	13C
P1	12.70 usec
PL1	-1.81 dB
PL1W	81.92915344 W
SFO1	150.8950149 MHz
======== CHANNEL f2 ========	
CPDPRG2	waltz16
NUC2	1H
PCPD2	80.00 usec
PL2	3.31 dB
PL12	23.31 dB
PL13	22.50 dB
PL2W	6.79873323 W
PL12W	0.06798734 W
PL13W	0.08192718 W
SFO2	600.0336002 MHz
SI	16384
SF	150.8774513 MHz
WDW	EM
SSB	0
LB	1.00 Hz
GB	0
PC	1.00

Mahwish / Dr. Iqbal / Dr-7' / MeOD
deptsp 90

Comp. 2

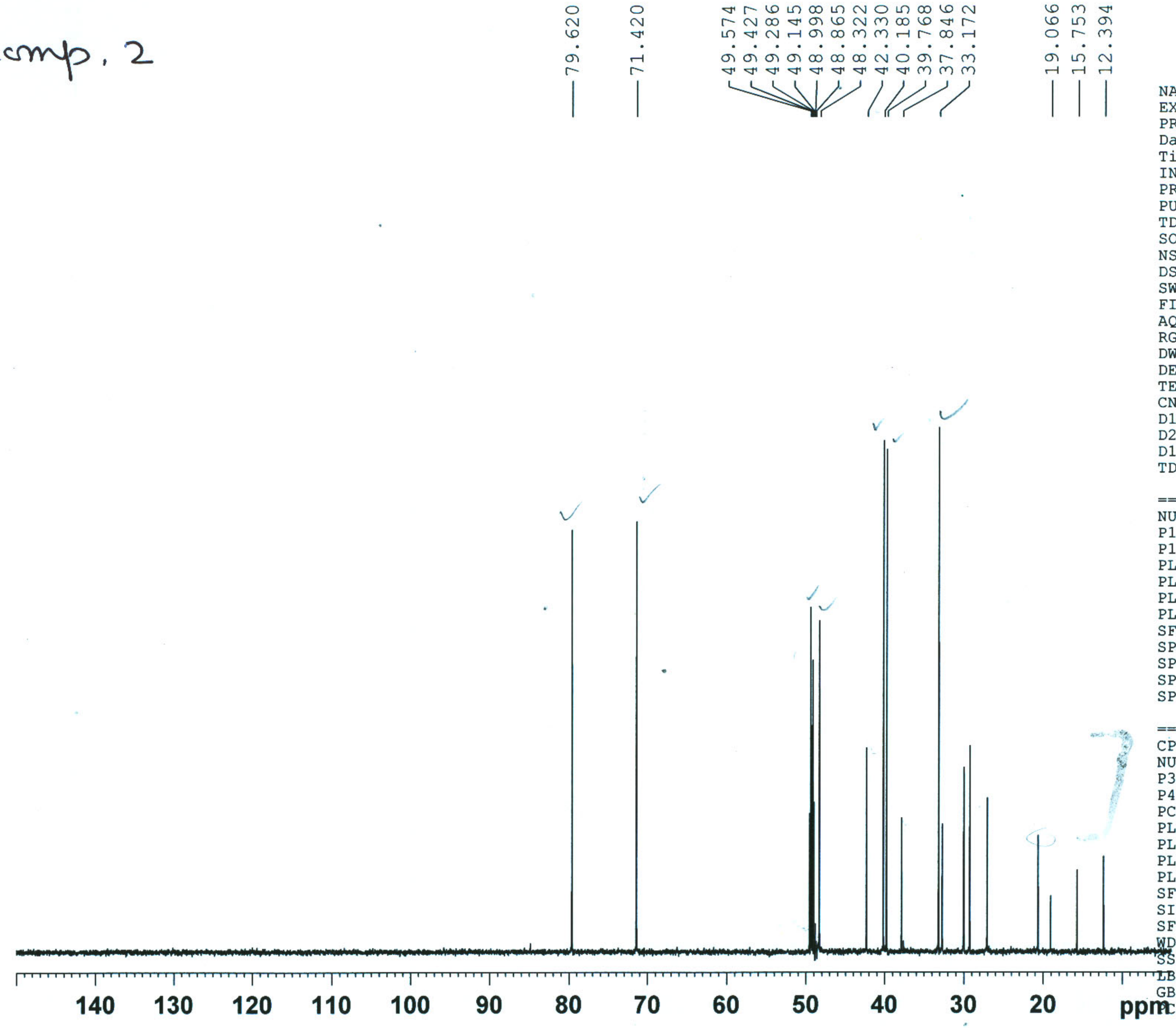

NAME	sep30-15	
EXPNO	9	
PROCNO	1	
Date_	20151001	
Time	10.22	
INSTRUM	spect	
PROBHD	5 mm CPTCI 1H-	
PULPROG	deptsp90	
TD	32768	
SOLVENT	MeOD	
NS	2138	
DS	2	
SWH	30303.031	Hz
FIDRES	0.924775	Hz
AQ	0.5407385	sec
RG	32768	
DW	16.500	usec
DE	6.50	usec
TE	298.0	K
CNST2	145.0000000	
D1	1.50000000	sec
D2	0.00344828	sec
D12	0.00002000	sec
TD0	3	
======== CHANNEL f1 ========		
NUC1	13C	
P1	12.70	usec
P12	2000.00	usec
PL0	120.00	dB
PL1	-1.81	dB
PL0W	0.00000000	W
PL1W	81.92915344	W
SFO1	150.8927518	MHz
SP2	4.19	dB
SPNAM2	Crp60comp.4	
SPOAL2	0.500	
SPOFFS2	0.00	Hz
======== CHANNEL f2 ========		
CPDPRG2	waltz16	
NUC2	1H	
P3	8.00	usec
P4	16.00	usec
PCPD2	80.00	usec
PL2	3.31	dB
PL12	23.31	dB
PL2W	6.79873323	W
PL12W	0.06798734	W
SFO2	600.0330002	MHz
SI	16384	
SF	150.8774513	MHz
WDW	EM	
SSB	0	
LB	1.00	Hz
GB	0	
PC	1.40	

Mahwish / Dr. Iqbal / Dr-7' / MeOD
dept135

comp. 2

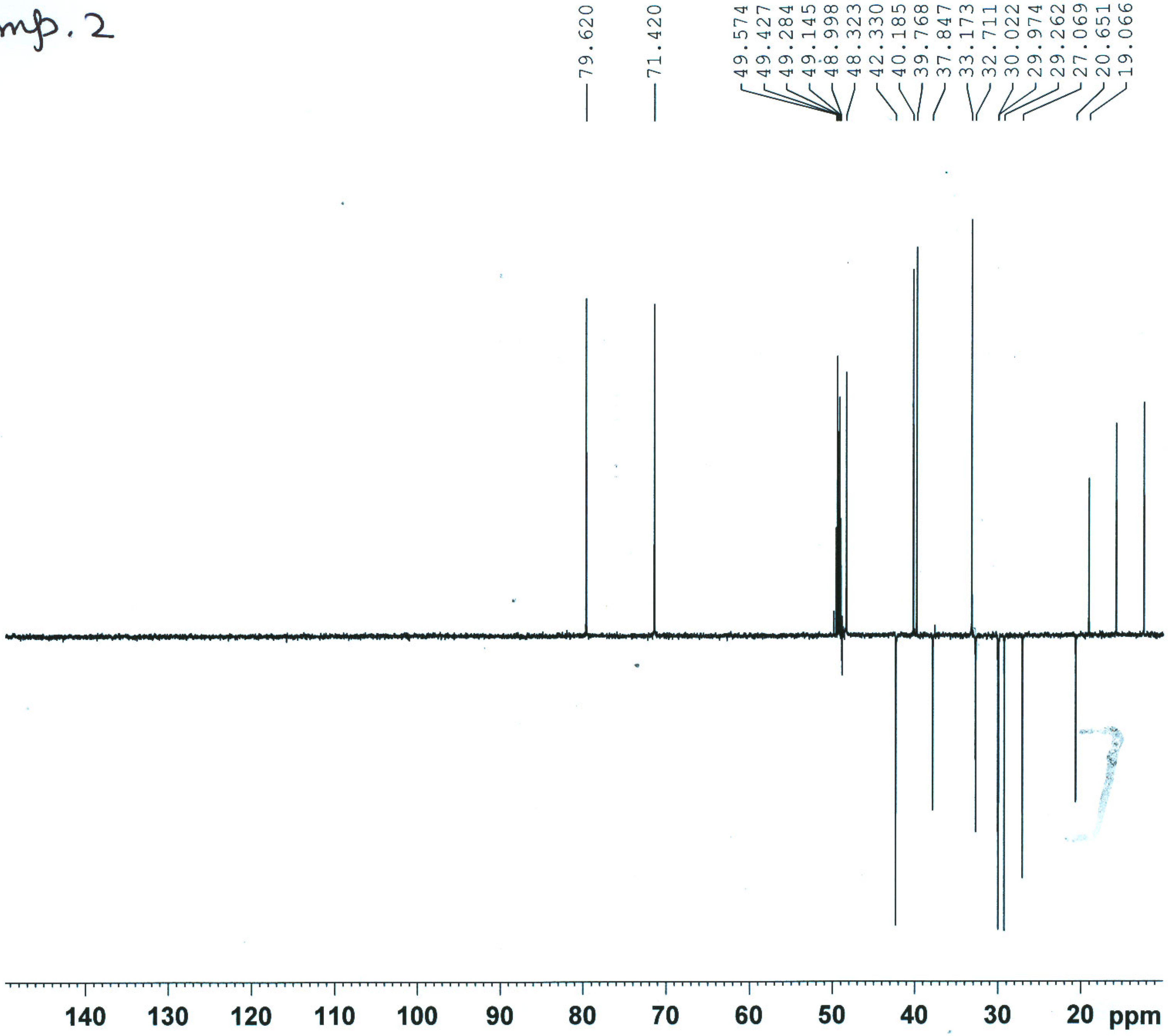

NAME	sep30-15	
EXPNO	8	
PROCNO	1	
Date_	20151001	
Time	9.01	
INSTRUM	spect	
PROBHD	5 mm CPTCI 1H-	
PULPROG	deptsp135	
TD	32768	
SOLVENT	MeOD	
NS	2051	
DS	2	
SWH	30303.031	Hz
FIDRES	0.924775	Hz
AQ	0.5407385	sec
RG	32768	
DW	16.500	usec
DE	6.50	usec
TE	298.0	K
CNST2	145.0000000	
D1	1.50000000	sec
D2	0.00344828	sec
D12	0.00002000	sec
TD0	8	
======== CHANNEL f1 ========		
NUC1	13C	
P1	12.70	usec
P12	2000.00	usec
PL0	120.00	dB
PL1	-1.81	dB
PL0W	0.00000000	W
PL1W	81.92915344	W
SFO1	150.8927518	MHz
SP2	4.19	dB
SPNAM2	Crp60comp.4	
SPOAL2	0.500	
SPOFFS2	0.00	Hz
======== CHANNEL f2 ========		
CPDPRG2	waltz16	
NUC2	1H	
P3	8.00	usec
P4	16.00	usec
PCPD2	80.00	usec
PL2	3.31	dB
PL12	23.31	dB
PL2W	6.79873323	W
PL12W	0.06798734	W
SFO2	600.0330002	MHz
SI	16384	
SF	150.8774513	MHz
WDW	EM	
SSB	0	
LB	1.00	Hz
GB	0	
PC	1.40	

Mahwish /Dr. Iqbal / Dr-7' / MeOD
HSQC

Comp. 2

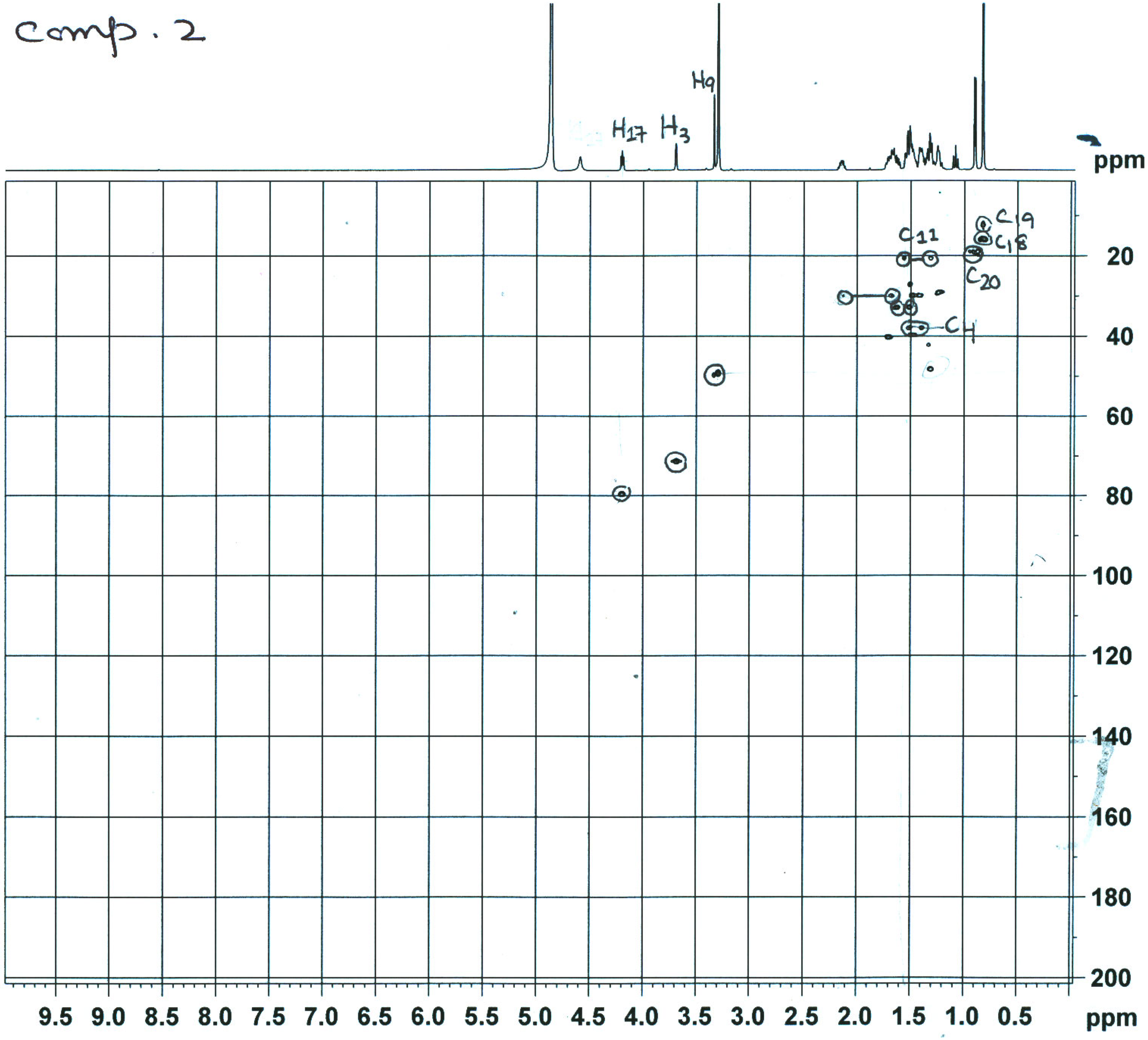

NAME	sep30-15	
EXPNO	5	
PROCNO	1	
Date_	20150930	
Time	16.08	
INSTRUM	spect	
PROBHD	5 mm CPTCI 1H-	
PULPROG	hsqcetgpsi	
TD	1024	
SOLVENT	MeOD	
NS	32	
DS	8	
SWH	6009.615	Hz
FIDRES	5.868765	Hz
AQ	0.0853300	sec
RG	32768	
DW	83.200	usec
DE	6.50	usec
TE	298.0	K
CNST2	145.0000000	
D0	0.00000300	sec
D1	2.00000000	sec
D4	0.00172414	sec
D11	0.03000000	sec
D13	0.00000400	sec
D16	0.00020000	sec
D24	0.00110000	sec
IN0	0.00001655	sec
ZGOPTNS		
======== CHANNEL f1 ========		
NUC1	1H	
P1	8.00	usec
P2	16.00	usec
P28	1000.00	usec
PL1	3.31	dB
PL1W	6.79873323	W
SFO1	600.0330002	MHz
======== CHANNEL f2 ========		
CPDPRG2	garp	
NUC2	13C	
P3	11.50	usec
P4	23.00	usec
PCPD2	55.00	usec
PL2	-1.81	dB
PL12	11.70	dB
PL2W	81.92915344	W
PL12W	3.65122390	W
SFO2	150.8927518	MHz
====== GRADIENT CHANNEL =====		
GPNAM1	SINE.100	
GPNAM2	SINE.100	
GPZ1	80.00	%
GPZ2	20.10	%
P16	1000.00	usec
ND0	2	

Mahwish / Dr. Iqbal / Dr-7' / MeOD
HMBC

comp. 2

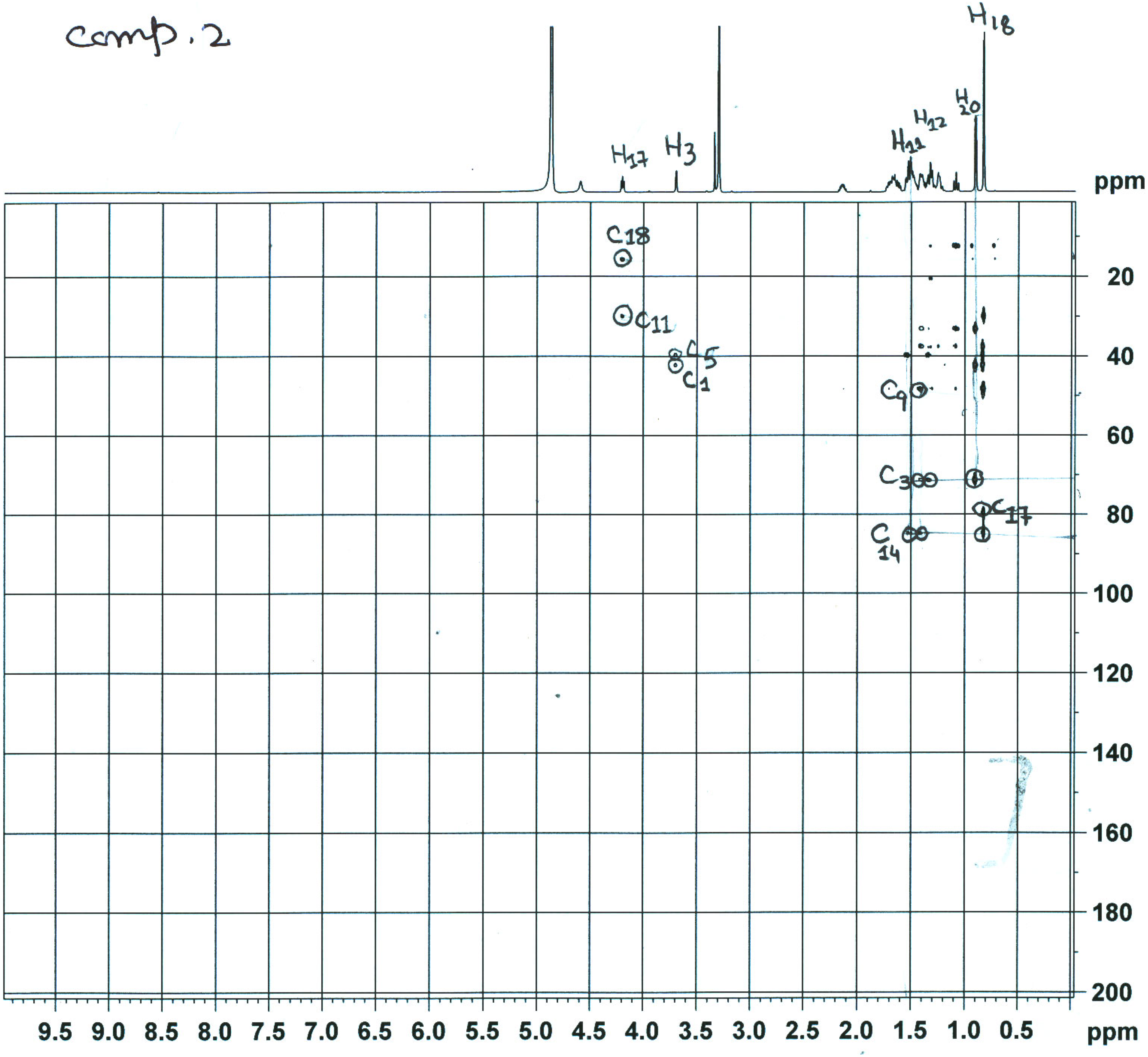

NAME	sep30-15
EXPNO	6
PROCNO	1
Date_	20150930
Time	20.57
INSTRUM	spect
PROBHD	5 mm CPTCI 1H-
PULPROG	hmbcgplpndqf
TD	2048
SOLVENT	MeOD
NS	64
DS	16
SWH	6009.615 Hz
FIDRES	2.934382 Hz
AQ	0.1705268 sec
RG	41285.1
DW	83.200 usec
DE	6.50 usec
TE	289.4 K
CNST2	145.0000000
CNST13	13.0000000
D0	0.00000300 sec
D1	2.00000000 sec
D2	0.00344828 sec
D6	0.03846154 sec
D16	0.00015000 sec
IN0	0.00001440 sec

======== CHANNEL f1 ========

NUC1	1H
P1	8.00 usec
P2	16.00 usec
PL1	3.31 dB
PL1W	6.79873323 W
SFO1	600.0330002 MHz

======== CHANNEL f2 ========

NUC2	13C
P3	11.50 usec
PL2	-1.81 dB
PL2W	81.92915344 W
SFO2	150.8950149 MHz

====== GRADIENT CHANNEL =====

GPNAM1	SINE.100
GPNAM2	SINE.100
GPNAM3	SINE.100
GPZ1	50.00 %
GPZ2	30.00 %
GPZ3	40.10 %
P16	2000.00 usec
ND0	2
TD	256
SFO1	150.895 MHz
FIDRES	135.569733 Hz
SW	230.000 ppm
FnMODE	QF
SI	2048

MAHWISH/DR.IQBAL/DR-7'/CD3OD
ICCBS/U.O.K
COSY

comp. 2

AVANCE AV-500
LAB NO:118

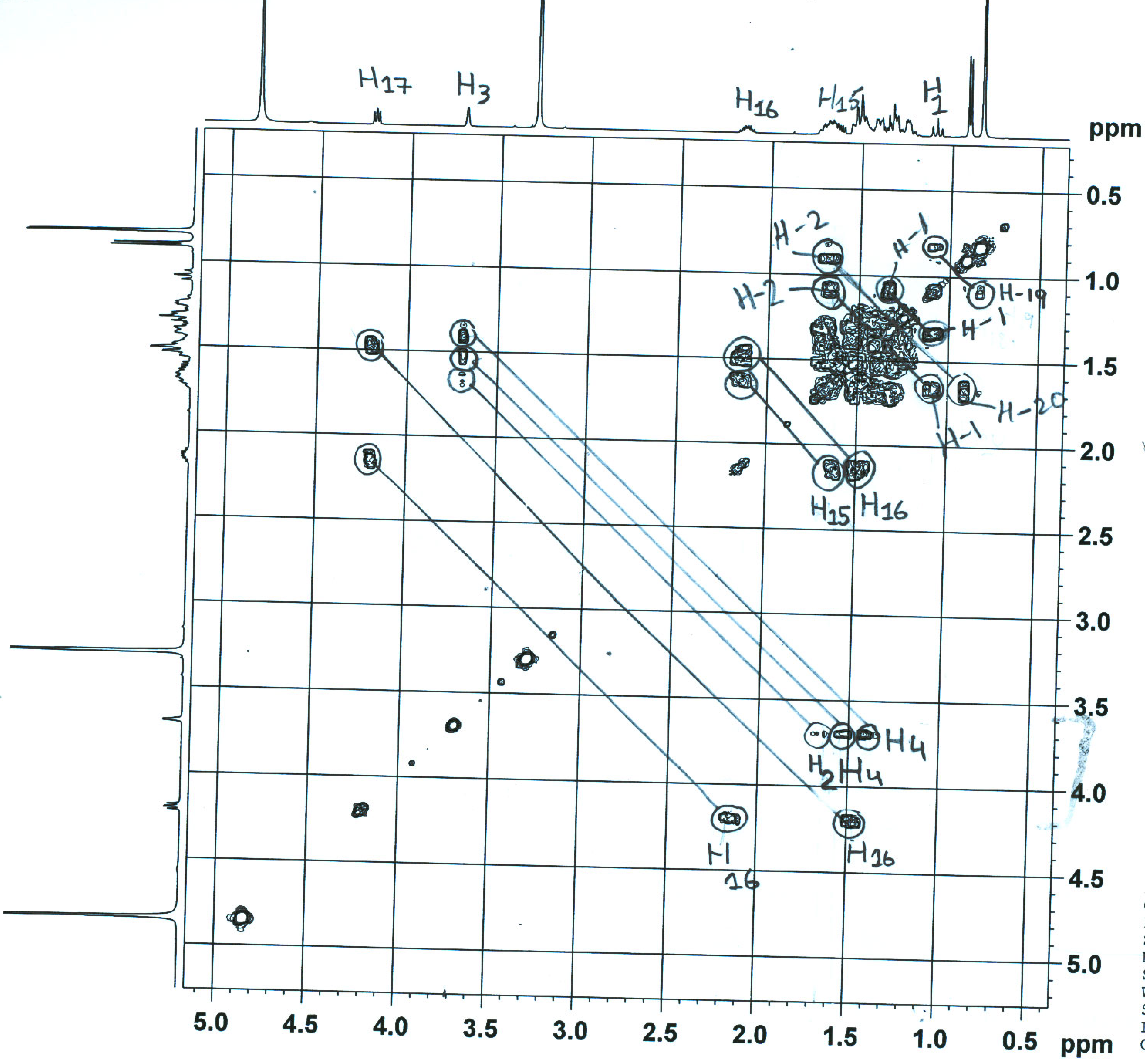

NAME	sep22-15
EXPNO	2
PROCNO	1
Date_	20150922
Time	10.27
INSTRUM	spect
PROBHD	5 mm PABBI 1H/
PULPROG	cosygpqf
TD	2048
SOLVENT	MeOD
NS	8
DS	8
SWH	4699.248 Hz
FIDRES	2.294555 Hz
AQ	0.2180636 sec
RG	812.7
DW	106.400 usec
DE	6.50 usec
TE	298.6 K
D0	0.00000300 sec
D1	1.50000000 sec
D13	0.00000400 sec
D16	0.00020000 sec
IN0	0.00021280 sec

======== CHANNEL f1 ========

NUC1	1H
P0	8.03 usec
P1	8.03 usec
PL1	3.00 dB
SFO1	500.2323511 MHz

====== GRADIENT CHANNEL =====

GPNAM1	SINE.100
GPZ1	10.00 %
P16	1000.00 usec
ND0	1
TD	256
SFO1	500.2324 MHz
FIDRES	18.356438 Hz
SW	9.394 ppm
FnMODE	QF
SI	1024
SF	500.2300119 MHz
WDW	QSINE
SSB	0
LB	0.00 Hz
GB	0
PC	1.00
SI	1024
MC2	QF
SF	500.2300119 MHz
WDW	QSINE
SSB	0
LB	0.00 Hz
GB	0

MAHWISH/DR.IQBAL/DR-7'/CD3OD
ICCBS/U.O.K
NOESY

Comp. 2

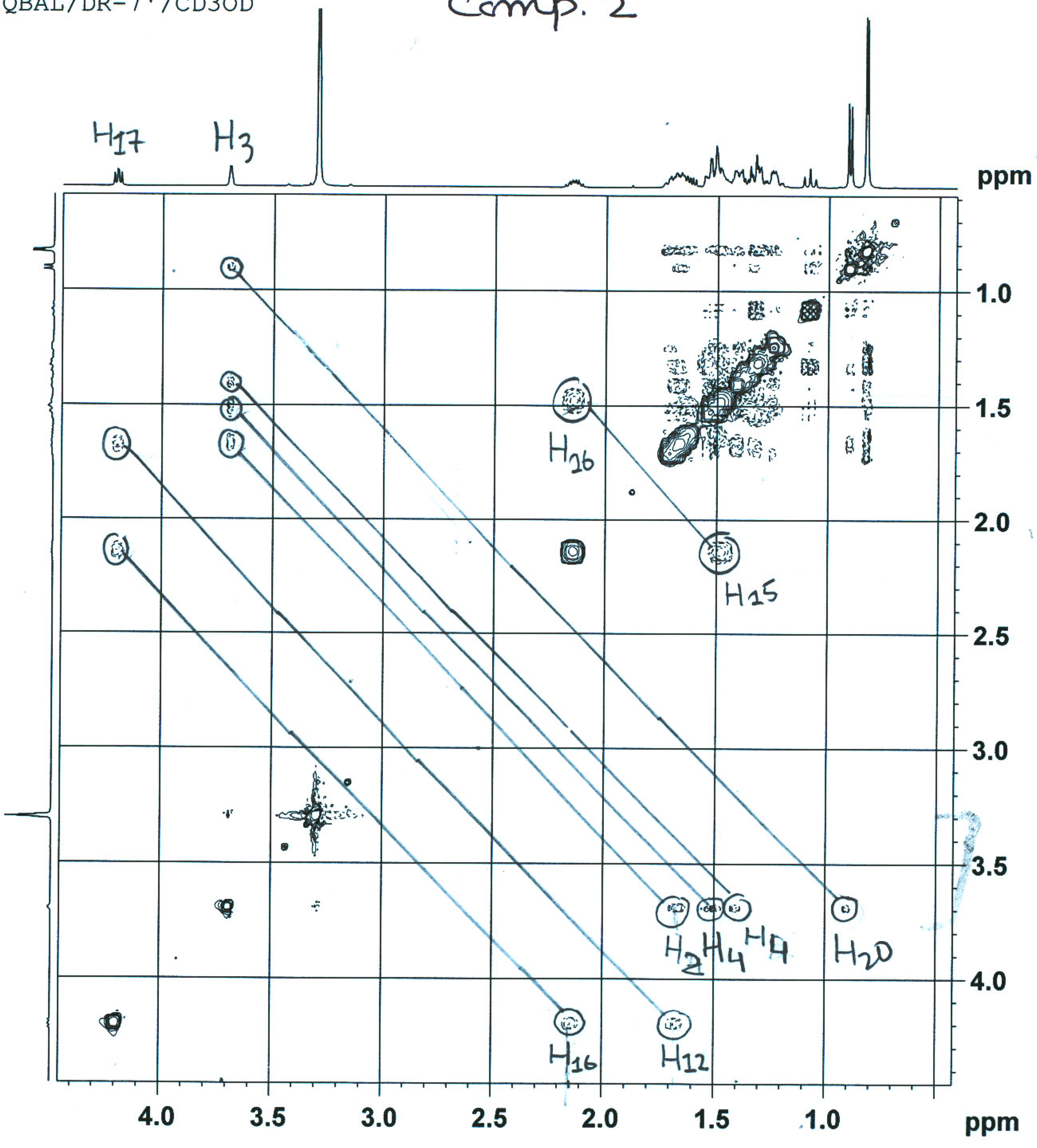

Supplement: Supplementary file 2 [file DataSheet2.PDF]
