# Supplementary material for: Bio-Catalytic Structural Transformation of Anti-cancer Steroid, Drostanolone Enanthate with Cephalosporium aphidicola and Fusarium lini, and Cytotoxic Potential Evaluation of Its Metabolites against Certain Cancer Cell Lines
Source: Front Pharmacol. 2017 Dec 20;8:900. doi: 10.3389/fphar.2017.00900 (PMC5742531; doi:10.3389/fphar.2017.00900)
Supplement: Supplementary file 3 [file DataSheet3.PDF]

File: FL-D-5
Sample: MAHWISH /DR. IQBAL
Instrument: JEOL MS 600H-1

Date Run: 01-14-2016 (Time Run: 15:37:48)

Ionization mode: EI+

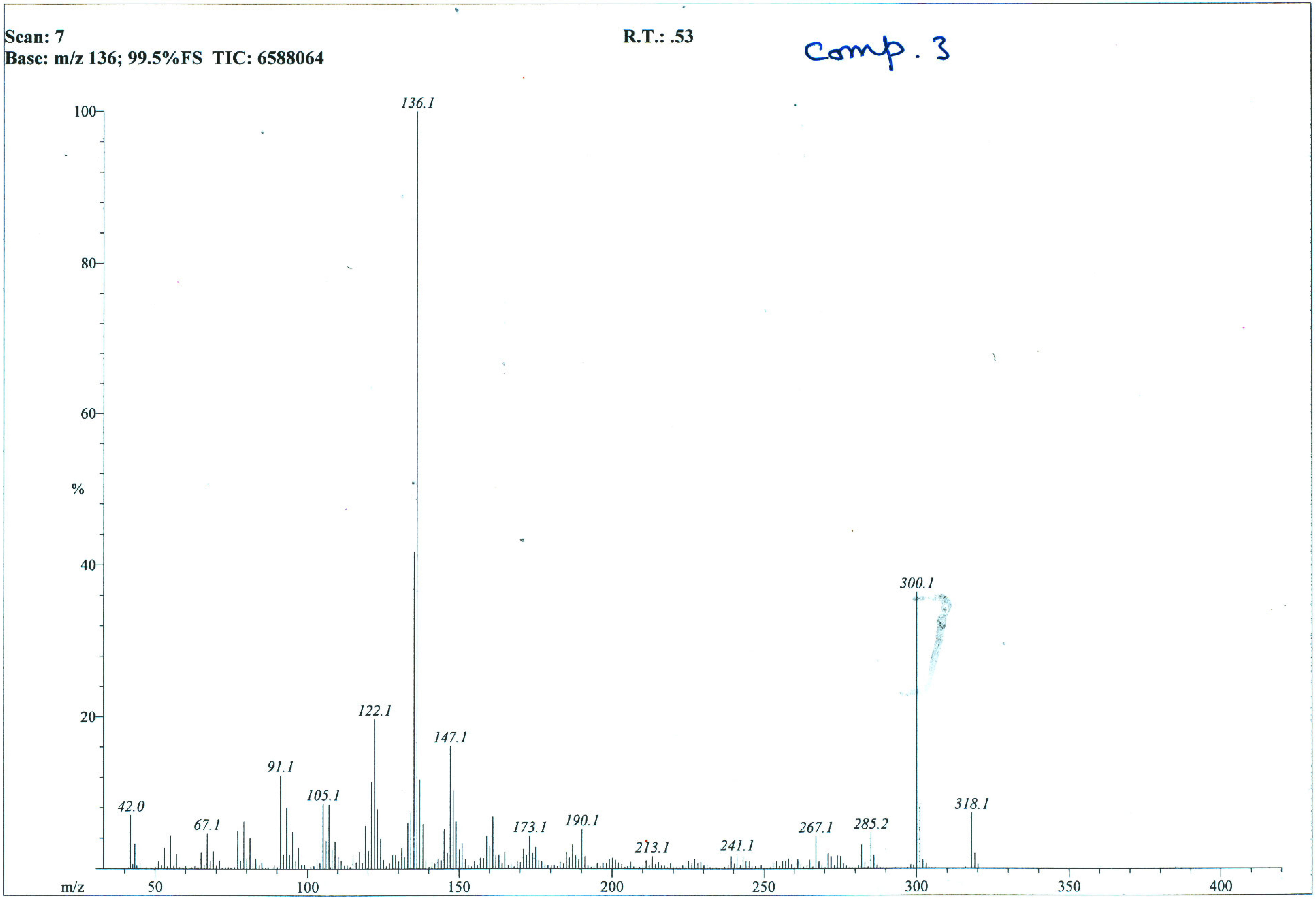

Comp. 3

Mass	Relative Intensity	Theoretical Mass	Delta [ppm]	Delta [mmu]	RDB	Composition
176.1213	1.1	176.1201	6.5	1.2	5.0	$C_{12} H_{16} O_1$
183.1160	1.0	183.1174	-7.3	-1.3	7.5	$C_{14} H_{15}$
185.0961	2.2	185.0966	-2.8	-0.5	7.5	$C_{13} H_{13} O_1$
186.1042	2.0	186.1045	-1.2	-0.2	7.0	$C_{13} H_{14} O_1$
187.1118	3.0	187.1123	-2.6	-0.5	6.5	$C_{13} H_{15} O_1$
188.1178	1.9	188.1201	-12.2	-2.3	6.0	$C_{13} H_{16} O_1$
189.1266	1.2	189.1279	-7.1	-1.3	5.5	$C_{13} H_{17} O_1$
190.1351	3.2	190.1358	-3.7	-0.7	5.0	$C_{13} H_{18} O_1$
191.1420	1.3	191.1436	-8.2	-1.6	4.5	$C_{13} H_{19} O_1$
199.1119	1.5	199.1123	-2.2	-0.4	7.5	$C_{14} H_{15} O_1$
200.1189	1.7	200.1201	-6.1	-1.2	7.0	$C_{14} H_{16} O_1$
201.1267	1.2	201.1279	-6.3	-1.3	6.5	$C_{14} H_{17} O_1$
213.1264	1.6	213.1279	-7.3	-1.6	7.5	$C_{15} H_{17} O_1$
225.1286	1.0	225.1279	2.9	0.7	8.5	$C_{16} H_{17} O_1$
239.1451	1.4	239.1436	6.3	1.5	8.5	$C_{17} H_{19} O_1$
239.1760	1.1	239.1800	-16.6	-4.0	7.5	$C_{18} H_{23}$
241.1588	2.2	241.1592	-1.9	-0.5	7.5	$C_{17} H_{21} O_1$
243.1735	1.4	243.1749	-5.6	-1.3	6.5	$C_{17} H_{23} O_1$
257.1879	1.0	257.1905	-10.4	-2.7	6.5	$C_{18} H_{25} O_1$
265.1945	1.1	265.1956	-4.1	-1.1	8.5	$C_{20} H_{25}$
267.1752	4.4	267.1749	1.1	0.3	8.5	$C_{19} H_{23} O_1$
268.1777	1.0	268.1827	-18.6	-5.0	8.0	$C_{19} H_{24} O_1$
271.2063	1.9	271.2062	0.4	0.1	6.5	$C_{19} H_{27} O_1$
272.2132	1.5	272.2140	-3.1	-0.9	6.0	$C_{19} H_{28} O_1$
274.1939	1.1	274.1933	2.1	0.6	6.0	$C_{18} H_{26} O_2$
275.2002	1.1	275.2011	-3.5	-1.0	5.5	$C_{18} H_{27} O_2$
282.1984	3.4	282.1984	-0.0	-0.0	8.0	$C_{20} H_{26} O_1$
285.1867	2.9	285.1855	4.3	1.2	7.5	$C_{19} H_{25} O_2$
300.2073	22.6	300.2089	-5.3	-1.6	7.0	$C_{20} H_{28} O_2$
301.2111	4.9	301.2168	-18.7	-5.6	6.5	$C_{20} H_{29} O_2$
318.2192	3.4	318.2195	-0.9	-0.3	6.0	$C_{20} H_{30} O_3$

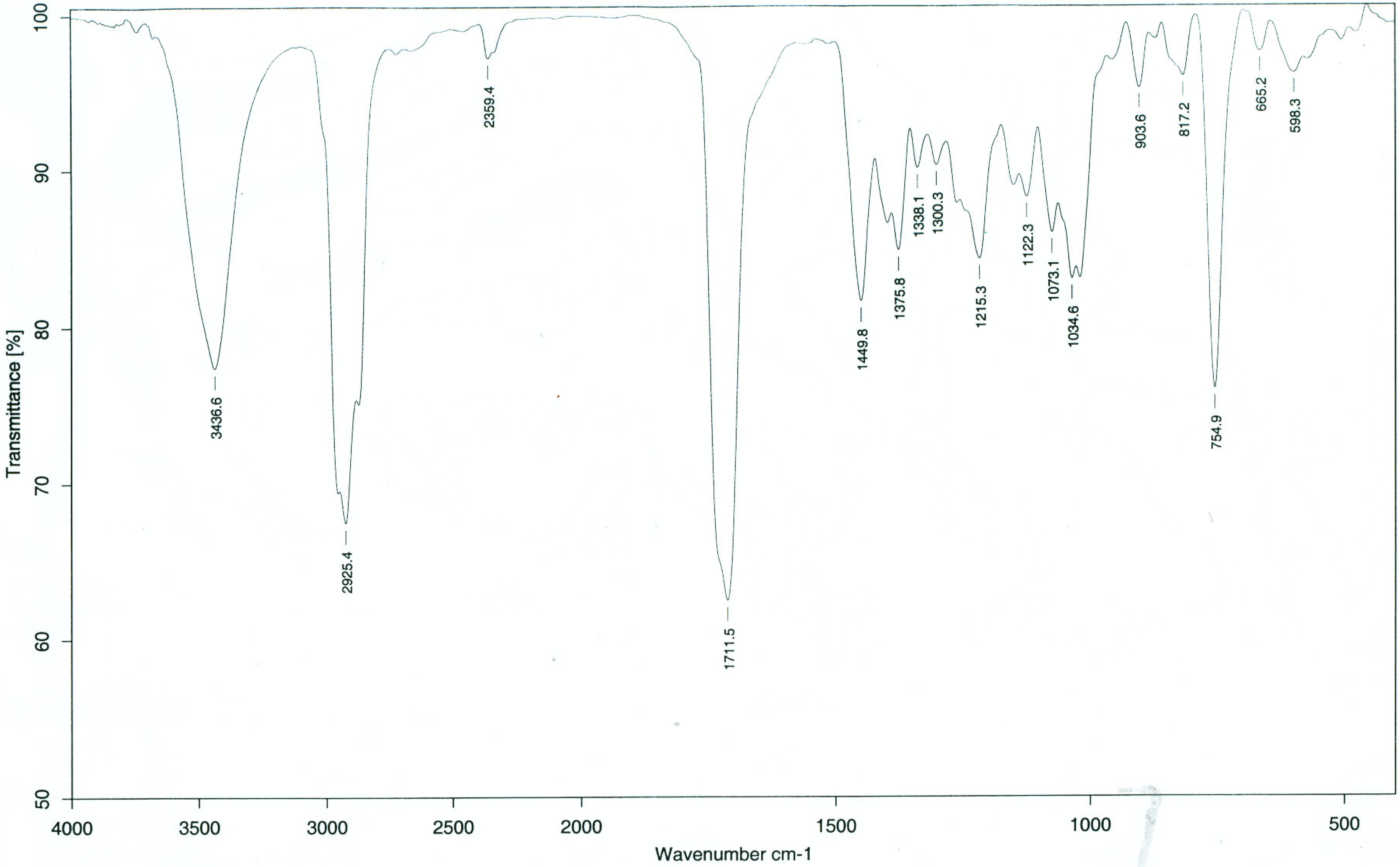

Sample : H-D-5/Mahwish. dR M .Iqbal Choudhary

Spectrum : H-D-5.1 (in D:\IRSTUDENT)

Measured : 23/05/2017 on VECTOR22

Technic : Liquid

Resolution : 4 cm-1 (10 scans)

Analyst : MA/ZA/JS

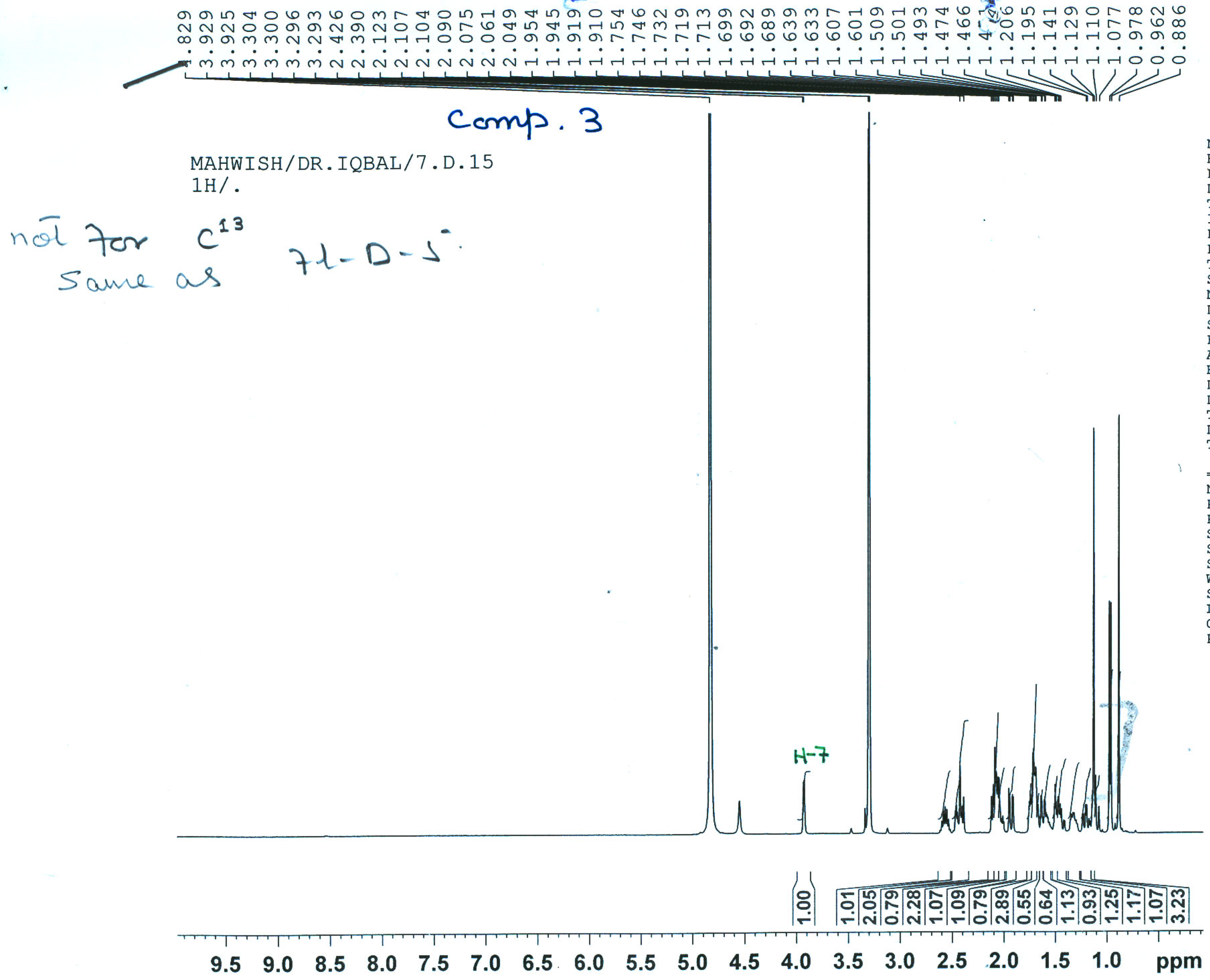

AVANCE AV-400 MHz
Lab # 115

NAME	june06-16
EXPNO	10
PROCNO	1
Date_	20160606
Time	14.57
INSTRUM	spect
PROBHD	5 mm SEI 1H-13
PULPROG	zg30
TD	65536
SOLVENT	MeOD
NS	128
DS	0
SWH	8012.820 Hz
FIDRES	0.122266 Hz
AQ	4.0894966 sec
RG	256
DW	62.400 usec
DE	6.50 usec
TE	300.0 K
D1	2.00000000 sec
TD0	1

======== CHANNEL f1 ========

NUC1	1H
P1	10.80 usec
PL1	3.00 dB
SFO1	400.0332002 MHz
SI	32768
SF	400.0300087 MHz
WDW	EM
SSB	0
LB	0.30 Hz
GB	0
PC	0.20

MAHWISH / DR.IQBAL / FL-D-5
BB

Comp. 3

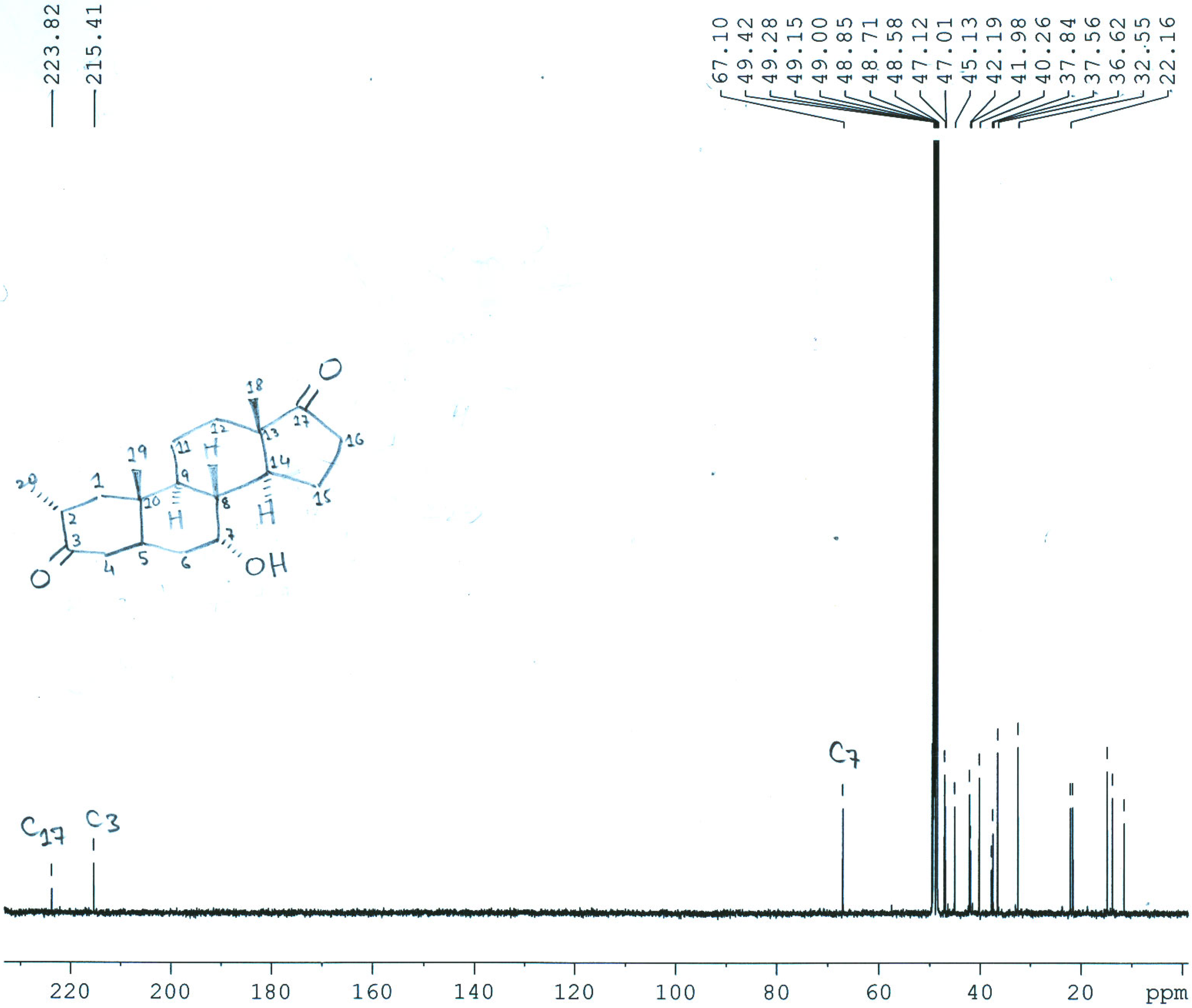

AVANCE AV-600
CRYO PROBE
LAB NO: 108

NAME	june17-15
EXPNO	6
PROCNO	1
Date_	20150618
Time	6.00
INSTRUM	spect
PROBHD	5 mm CPTCI 1H-
PULPROG	zgpg
TD	32768
SOLVENT	MeOD
NS	7405
DS	2
SWH	35971.223 H
FIDRES	1.097755 H
AQ	0.4555391 se
RG	32768
DW	13.900 us
DE	6.50 us
TE	298.0 K
D1	1.50000000 se
D11	0.03000000 se
TD0	10

======== CHANNEL f1 =====

NUC1	13C
P1	15.40 us
PL1	1.00 d
PL1W	83.60149384 W
SFO1	150.9453107 M

======== CHANNEL f2 =====

CPDPRG2	waltz16
NUC2	1H
PCPD2	65.00 us
PL2	3.30 d
PL12	22.06 d
PL13	27.00 d
PL2W	9.16420078 W
PL12W	0.12192553 W
PL13W	0.03909260 W
SFO2	600.2336014 M
SI	16384
SF	150.9277402 M
WDW	EM
SSB	0
LB	1.00 H
GB	0
PC	1.00

MAHWISH / DR.IQBAL / 7L-D-5
DEPT90

comp. 3

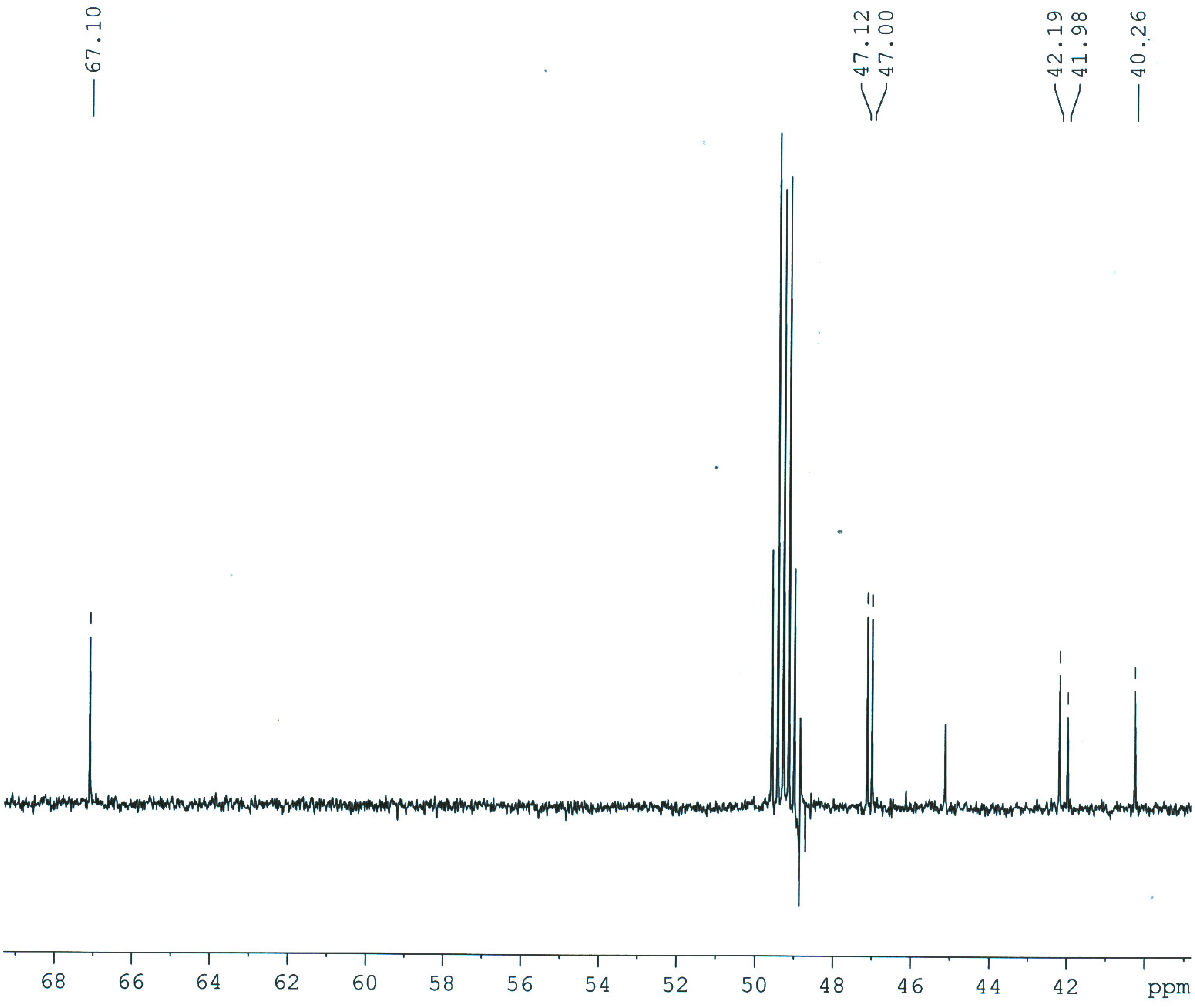

AVANCE AV-600
CRYO PROBE
LAB NO: 108

NAME	june17-15	
EXPNO	8	
PROCNO	1	
Date_	20150618	
Time	12.14	
INSTRUM	spect	
PROBHD	5 mm CPTCI 1H-	
PULPROG	deptsp90	
TD	32768	
SOLVENT	MeOD	
NS	1788	
DS	2	
SWH	30303.031	Hz
FIDRES	0.924775	Hz
AQ	0.5407385	sec
RG	32768	
DW	16.500	usec
DE	6.50	usec
TE	298.0	K
CNST2	145.0000000	
D1	1.50000000	sec
D2	0.00344828	sec
D12	0.00002000	sec
TD0	4	

======== CHANNEL f1 ========

NUC1	13C	
P1	15.40	usec
P12	2000.00	usec
PL0	120.00	dB
PL1	1.00	dB
PL0W	0.00000000	W
PL1W	83.60149384	W
SFO1	150.9430468	MHz
SP2	5.40	dB
SPNAM2	Crp60comp.4	
SPOAL2	0.500	
SPOFFS2	0.00	Hz

======== CHANNEL f2 ========

CPDPRG2	waltz16	
NUC2	1H	
P3	7.50	usec
P4	15.00	usec
PCPD2	65.00	usec
PL2	3.30	dB
PL12	22.06	dB
PL2W	9.16420078	W
PL12W	0.12192553	W
SFO2	600.2324009	MHz
SI	16384	
SF	150.9277402	MHz
WDW	EM	
SSB	0	
LB	1.00	Hz
GB	0	
PC	1.00	

Comp. 3

MAHWISH / DR.IQBAL / 7L-D-5
DEPT135

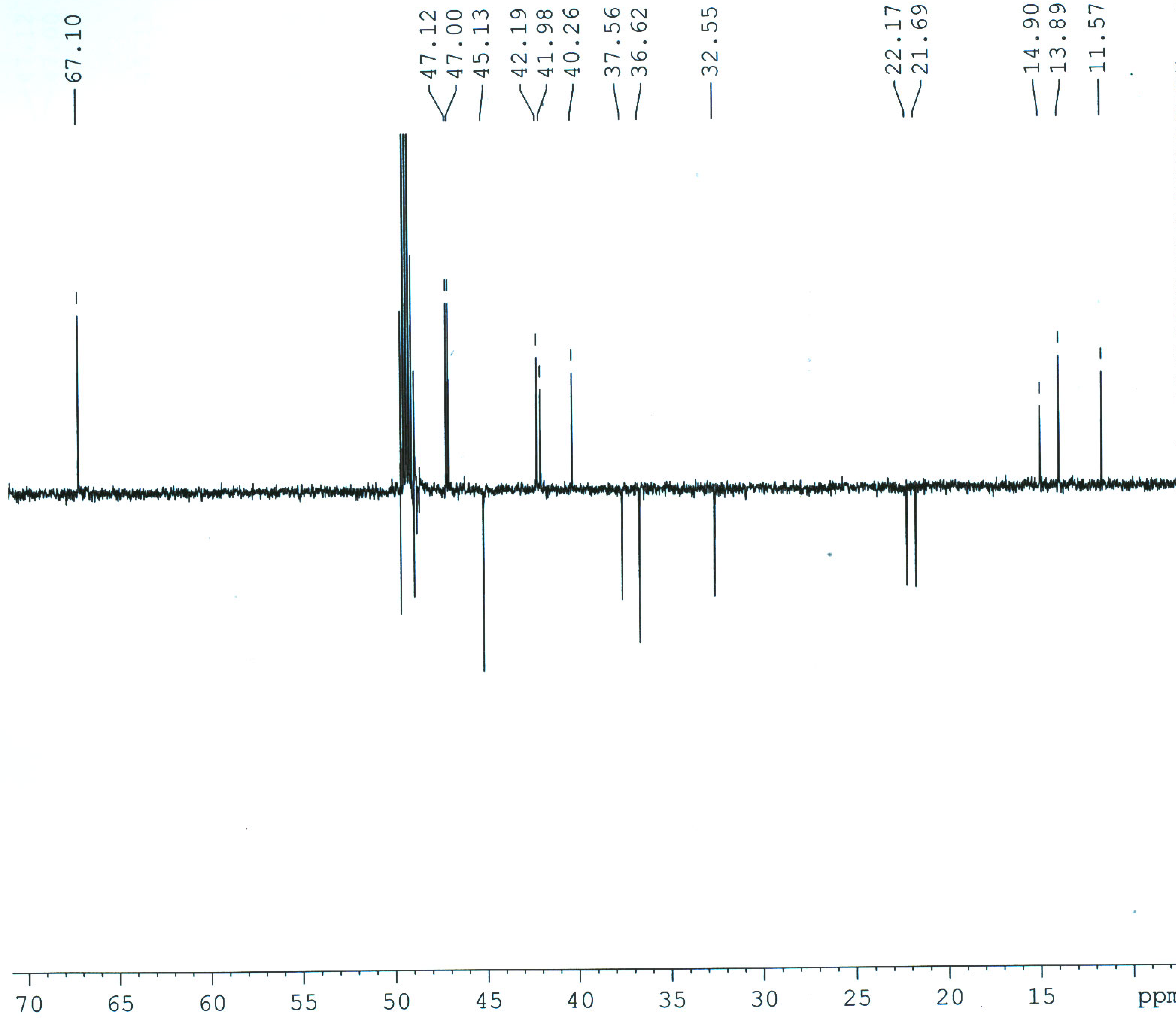

BRUKER

NAME	june17-15
EXPNO	7
PROCNO	1
Date_	20150618
Time	10.11
INSTRUM	spect
PROBHD	5 mm CPTCI 1H-
PULPROG	deptsp135
TD	32768
SOLVENT	MeOD
NS	3611
DS	2
SWH	30303.031 Hz
FIDRES	0.924775 Hz
AQ	0.5407385 sec
RG	32768
DW	16.500 usec
DE	6.50 usec
TE	298.0 K
CNST2	145.0000000
D1	1.50000000 sec
D2	0.00344828 sec
D12	0.00002000 sec
TD0	6
======== CHANNEL f1 ========	
NUC1	13C
P1	15.40 usec
P12	2000.00 usec
PL0	120.00 dB
PL1	1.00 dB
PL0W	0.00000000 W
PL1W	83.60149384 W
SFO1	150.9430468 MHz
SP2	5.40 dB
SPNAM2	Crp60comp.4
SPOAL2	0.500
SPOFFS2	0.00 Hz
======== CHANNEL f2 ========	
CPDPRG2	waltz16
NUC2	1H
P3	7.50 usec
P4	15.00 usec
PCPD2	65.00 usec
PL2	3.30 dB
PL12	22.06 dB
PL2W	9.16420078 W
PL12W	0.12192553 W
SFO2	600.2324009 MHz
SI	16384
SF	150.9277402 MHz
WDW	EM
SSB	0
LB	1.00 Hz
GB	0
PC	1.00

MAHWISH / DR.IQBAL / 7L-D-5
HSQC

Comp. 3

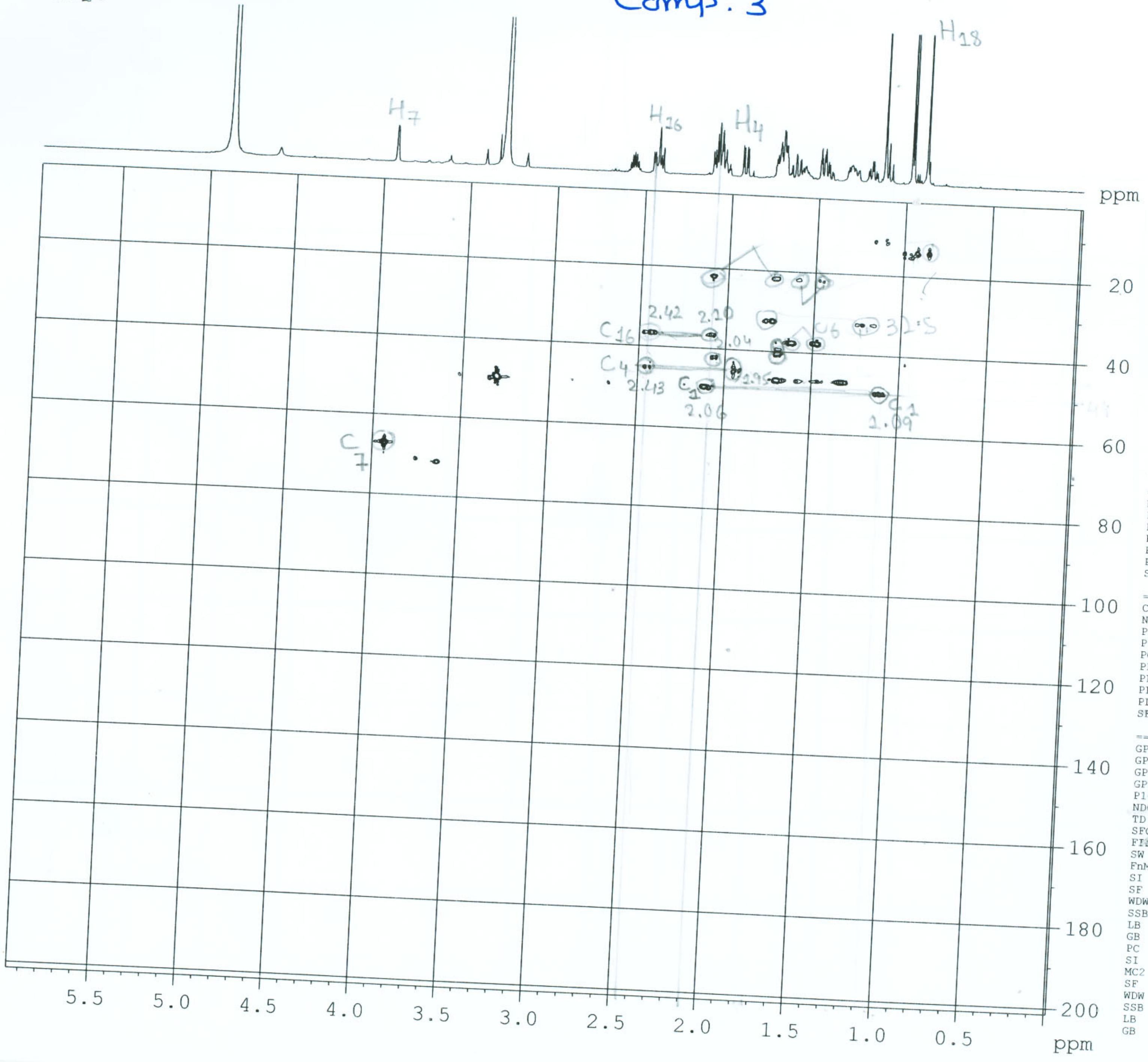

AVANCE AV-600
CRYO PROBE
LAB NO: 108

NAME	june17-15
EXPNO	4
PROCNO	1
Date_	20150617
Time	19.39
INSTRUM	spect
PROBHD	5 mm CPTCI 1H-
PULPROG	hsqcetgpsi
TD	1024
SOLVENT	MeOD
NS	32
DS	8
SWH	3591.954 Hz
FIDRES	3.507768 Hz
AQ	0.1427300 sec
RG	41285.1
DW	139.200 usec
DE	6.50 usec
TE	298.0 K
CNST2	145.0000000
D0	0.00000300 sec
D1	1.50000000 sec
D4	0.00172414 sec
D11	0.03000000 sec
D13	0.00000400 sec
D16	0.00015000 sec
D24	0.00110000 sec
IN0	0.00001655 sec
ZGOPTNS	

======== CHANNEL f1 ========

NUC1	1H
P1	7.20 usec
P2	14.40 usec
P28	0.50 usec
PL1	3.30 dB
PL1W	9.16420078 W
SFO1	600.2318007 MHz

======== CHANNEL f2 ========

CPDPRG2	garp
NUC2	13C
P3	15.40 usec
P4	30.80 usec
PCPD2	61.00 usec
PL2	1.00 dB
PL12	13.00 dB
PL2W	83.60149384 W
PL12W	5.27489758 W
SFO2	150.9430468 MHz

===== GRADIENT CHANNEL =====

GPNAM1	SINE.100
GPNAM2	SINE.100
GPZ1	80.00 %
GPZ2	20.10 %
P16	2000.00 usec
ND0	2
TD	256
SFO1	150.943 MHz
FIDRES	117.924255 Hz
SW	200.000 ppm
FnMODE	Echo-Antiecho
SI	1024
SF	600.2300154 MHz
WDW	QSINE
SSB	2
LB	0.00 Hz
GB	0
PC	4.00
SI	1024
MC2	echo-antiecho
SF	150.9277402 MHz
WDW	QSINE
SSB	2
LB	0.00 Hz
GB	0

MAHWISH / DR.IQBAL / 7L-D-5
HMBC

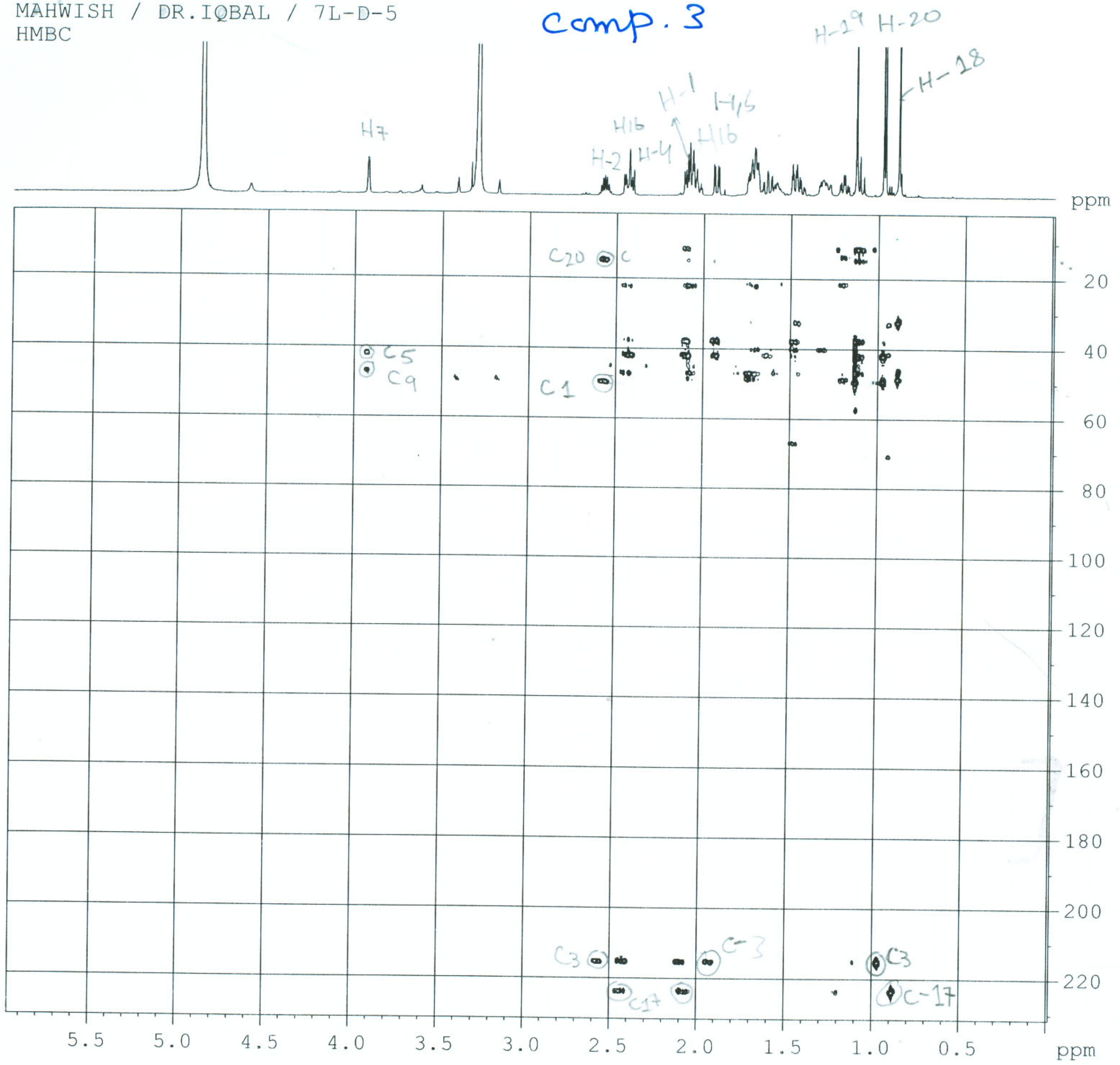

AVANCE AV-600
CRYO PROBE
LAB NO: 108

NAME	june17-15
EXPNO	5
PROCNO	1
Date_	20150617
Time	23.28
INSTRUM	spect
PROBHD	5 mm CPTCI 1H-
PULPROG	hmbcgplpndqf
TD	4096
SOLVENT	MeOD
NS	32
DS	16
SWH	3591.954 Hz
FIDRES	0.876942 Hz
AQ	0.5703524 sec
RG	46341
DW	139.200 usec
DE	6.50 usec
TE	298.0 K
CNST2	145.0000000
CNST13	13.0000000
D0	0.00000300 sec
D1	2.00000000 sec
D2	0.00344828 sec
D6	0.03846154 sec
D16	0.00015000 sec
IN0	0.00001440 sec

======== CHANNEL f1 ========

NUC1	1H
P1	7.20 usec
P2	14.40 usec
PL1	3.30 dB
PL1W	9.16420078 W
SFO1	600.2318007 MHz

======== CHANNEL f2 ========

NUC2	13C
P3	15.40 usec
PL2	1.00 dB
PL2W	83.60149384 W
SFO2	150.9453107 MHz

====== GRADIENT CHANNEL =====

GPNAM1	SINE.100
GPNAM2	SINE.100
GPNAM3	SINE.100
GPZ1	50.00 %
GPZ2	30.00 %
GPZ3	40.10 %
P16	2000.00 usec
ND0	2
TD	256
SFO1	150.9453 MHz
FIDRES	135.614929 Hz
SW	230.000 ppm
FnMODE	QF
SI	1024
SF	600.2300154 MHz
WDW	SINE
SSB	0
LB	0.00 Hz
GB	0
PC	1.40
SI	1024
MC2	QF
SF	150.9277402 MHz
WDW	SINE
SSB	0
LB	0.00 Hz
GB	0

MAHWISH / DR.IQBAL / .7L-D-5
ICCBS/U.O.K
COSY

Comp. 3

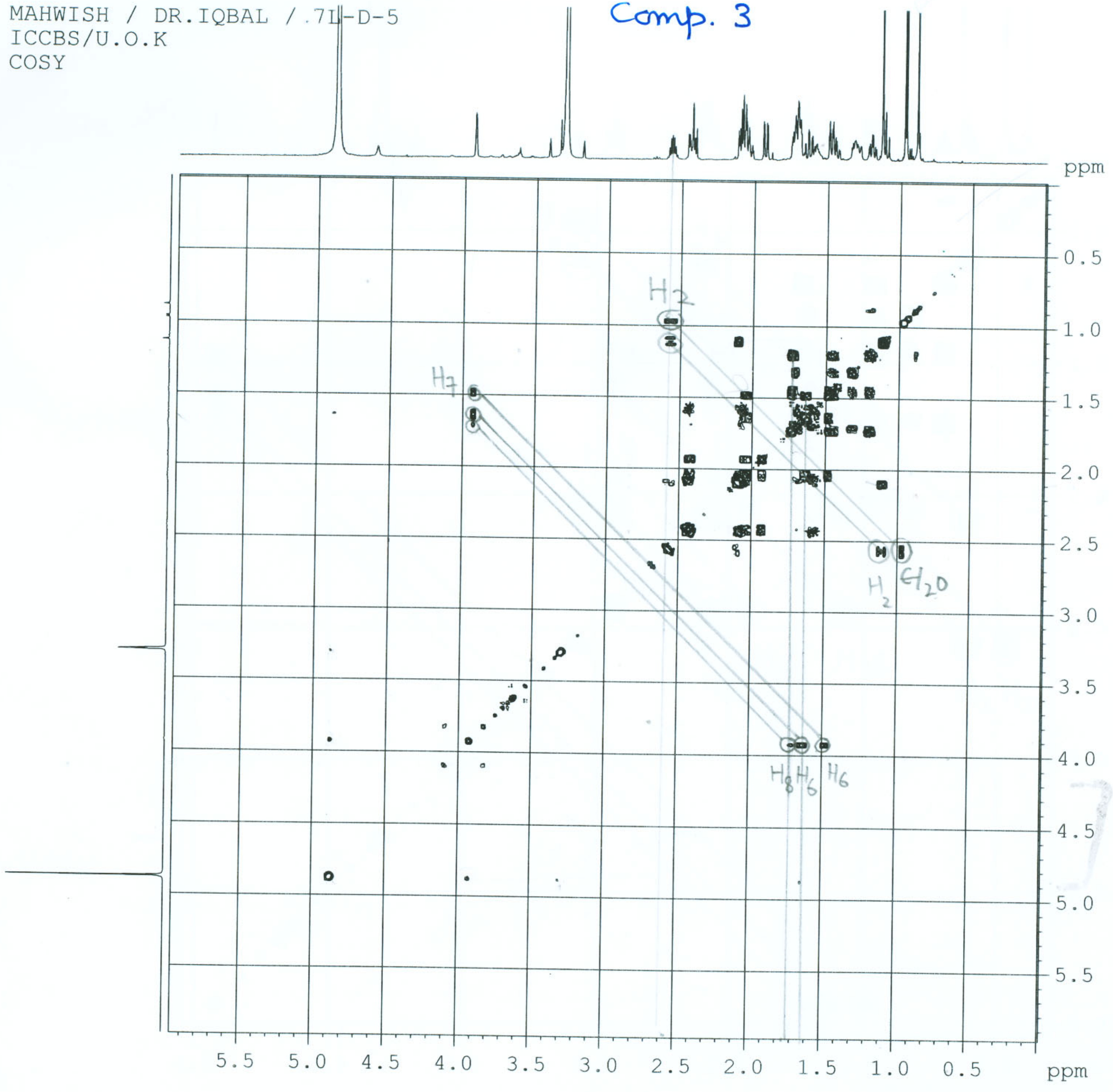

AVANCE AV-600
CRYO PROBE
LAB NO: 108

NAME	june17-15	
EXPNO	2	
PROCNO	1	
Date_	20150617	
Time	14.10	
INSTRUM	spect	
PROBHD	5 mm CPTCI 1H-	
PULPROG	cosydfqf	
TD	2048	
SOLVENT	MeOD	
NS	16	
DS	4	
SWH	3591.954	Hz
FIDRES	1.753884	Hz
AQ	0.2852708	sec
RG	35.9	
DW	139.200	usec
DE	6.50	usec
TE	298.0	K
D0	0.00000300	sec
D1	1.50000000	sec
D13	0.00000400	sec
D20	0.00000200	sec
IN0	0.00027840	sec

======== CHANNEL f1 ========

NUC1	1H	
P1	7.20	usec
PL1	3.30	dB
PL1W	9.16420078	W
SFO1	600.2318007	MHz
ND0	1	
TD	256	
SFO1	600.2318	MHz
FIDRES	14.031071	Hz
SW	5.984	ppm
FnMODE	QF	
SI	1024	
SF	600.2300154	MHz
WDW	QSINE	
SSB	0	
LB	0.00	Hz
GB	0	
PC	1.40	
SI	1024	
MC2	QF	
SF	600.2300154	MHz
WDW	QSINE	
SSB	0	
LB	0.00	Hz
GB	0	

Comp. 3

MAHWISH / DR.IQBAL / 7L-D-5
ICCBS/U.O.K
NOESY

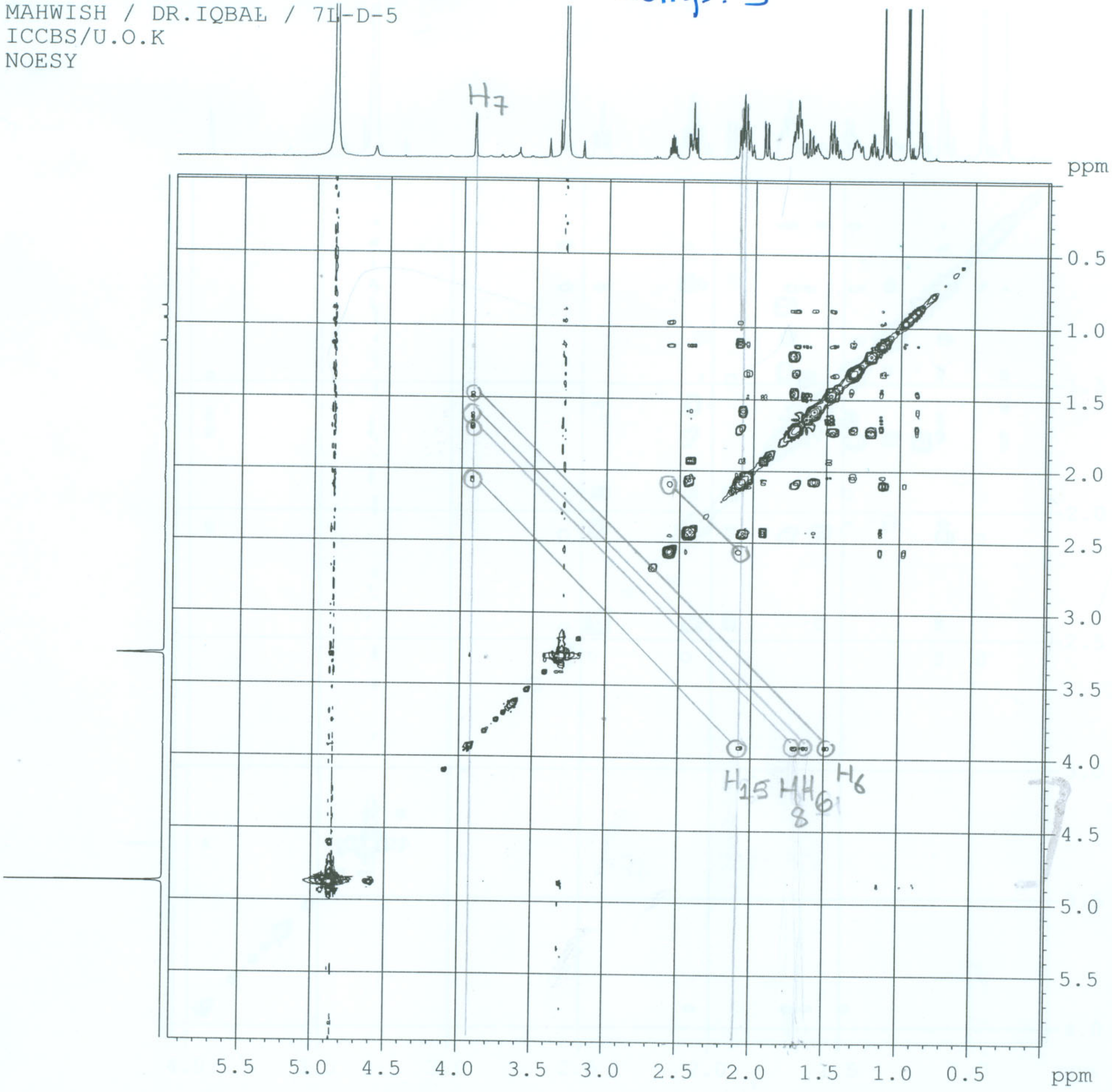

AVANCE AV-600
CRYO PROBE
LAB NO: 108

NAME	june17-15
EXPNO	3
PROCNO	1
Date_	20150617
Time	16.15
INSTRUM	spect
PROBHD	5 mm CPTCI 1H-
PULPROG	noesygpph
TD	1024
SOLVENT	MeOD
NS	16
DS	4
SWH	3591.954 Hz
FIDRES	3.507768 Hz
AQ	0.1427300 sec
RG	90.5
DW	139.200 usec
DE	6.50 usec
TE	298.0 K
D0	0.00012978 sec
D1	2.00000000 sec
D8	0.80000001 sec
D16	0.00015000 sec
IN0	0.00027840 sec

======== CHANNEL f1 ========

NUC1	1H
P1	7.40 usec
P2	14.80 usec
PL1	3.30 dB
PL1W	9.16420078 W
SFO1	600.2318007 MHz

====== GRADIENT CHANNEL =====

GPNAM1	SINE.100
GPNAM2	SINE.100
GPZ1	40.00 %
GPZ2	-40.00 %
P16	2000.00 usec
ND0	1
TD	256
SFO1	600.2318 MHz
FIDRES	14.031071 Hz
SW	5.984 ppm
FnMODE	States-TPPI
SI	1024
SF	600.2300154 MHz
WDW	QSINE
SSB	2
LB	0.00 Hz
GB	0
PC	1.40
SI	1024
MC2	States-TPPI
SF	600.2300154 MHz
WDW	QSINE
SSB	2
LB	0.00 Hz
GB	0
