# Supplementary material for: Bio-Catalytic Structural Transformation of Anti-cancer Steroid, Drostanolone Enanthate with Cephalosporium aphidicola and Fusarium lini, and Cytotoxic Potential Evaluation of Its Metabolites against Certain Cancer Cell Lines
Source: Front Pharmacol. 2017 Dec 20;8:900. doi: 10.3389/fphar.2017.00900 (PMC5742531; doi:10.3389/fphar.2017.00900)
Supplement: Supplementary file 4 [file DataSheet4.PDF]

File: AP-Dr-4
Sample: MAHWISH /DR. IQBAL
Instrument: JEOL MS 600H-1

Date Run: 06-26-2015 (Time Run: 11:56:59)

Ionization mode: EI+

Scan: 13
Base: m/z 121; 46.4%FS TIC: 15604035

R.T.: 1.07

Comp. 4

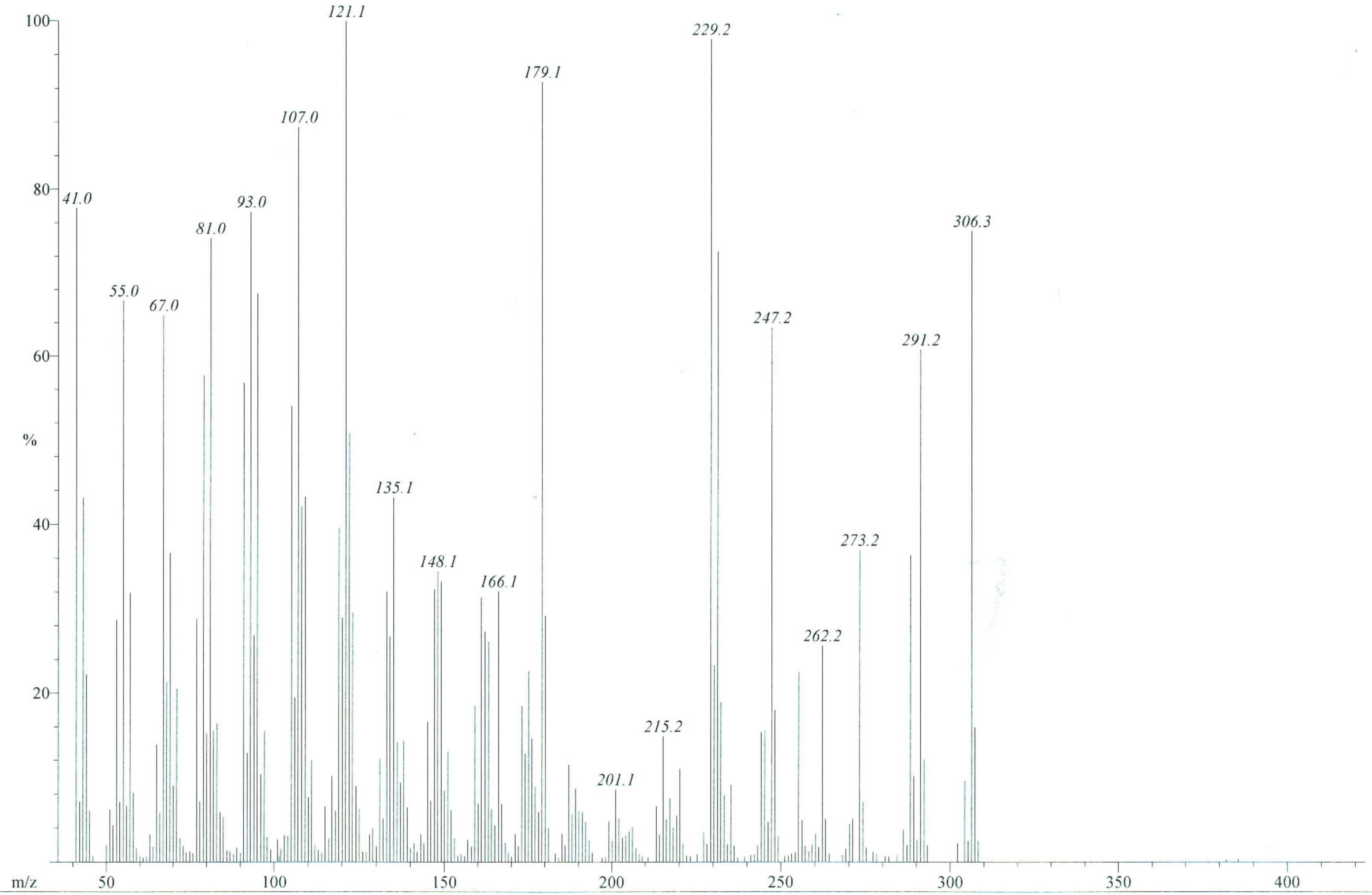

Comp - 4

Mass	Relative Intensity	Theoretical Mass	Delta [ppm]	Delta [mmu]	RDB	Composition
229.1938	36.7	229.1956	-7.9	-1.8	5.5	$C_{17} H_{25}$
230.1983	8.6					
231.2093	26.2	231.2113	-8.5	-2.0	4.5	$C_{17} H_{27}$
232.1841	3.1	232.1827	6.0	1.4	5.0	$C_{16} H_{24} O_1$
232.2123	4.8					
233.1908	3.6	233.1905	1.1	0.2	4.5	$C_{16} H_{25} O_1$
234.1999	1.2	234.1984	6.6	1.5	4.0	$C_{16} H_{26} O_1$
235.1677	3.9	235.1698	-8.7	-2.1	4.5	$C_{15} H_{23} O_2$
244.2221	7.2	244.2191	12.1	3.0	5.0	$C_{18} H_{28}$
245.1920	4.2	245.1905	5.8	1.4	5.5	$C_{17} H_{25} O_1$
245.2249	3.7	245.2269	-8.1	-2.0	4.5	$C_{18} H_{29}$
246.1963	1.2	246.1984	-8.5	-2.1	5.0	$C_{17} H_{26} O_1$
246.2279	1.1					
247.2058	19.5	247.2062	-1.5	-0.4	4.5	$C_{17} H_{27} O_1$
248.2105	4.8	248.2140	-14.3	-3.5	4.0	$C_{17} H_{28} O_1$
255.2112	8.0	255.2113	-0.2	-0.1	6.5	$C_{19} H_{27}$
256.2110	1.8					
260.2156	2.4	260.2140	6.0	1.6	5.0	$C_{18} H_{28} O_1$
262.2272	7.4	262.2297	-9.2	-2.4	4.0	$C_{18} H_{30} O_1$
263.2332	1.3	263.2375	-16.3	-4.3	3.5	$C_{18} H_{31} O_1$
270.2373	1.7	270.2348	9.5	2.6	6.0	$C_{20} H_{30}$
271.2052	2.2	271.2062	-3.5	-1.0	6.5	$C_{19} H_{27} O_1$
273.2221	16.9	273.2218	1.1	0.3	5.5	$C_{19} H_{29} O_1$
274.2260	4.1	274.2297	-13.3	-3.7	5.0	$C_{19} H_{30} O_1$
286.2308	1.4	286.2297	3.9	1.1	6.0	$C_{20} H_{30} O_1$
288.2463	11.1	288.2453	3.6	1.0	5.0	$C_{20} H_{32} O_1$
289.2477	3.1	289.2531	-19.0	-5.5	4.5	$C_{20} H_{33} O_1$
291.2322	19.6	291.2324	-0.7	-0.2	4.5	$C_{19} H_{31} O_2$
292.2347	3.7	292.2402	-18.9	-5.5	4.0	$C_{19} H_{32} O_2$
302.2267	1.1	302.2246	7.2	2.2	6.0	$C_{20} H_{30} O_2$
304.2349	5.8	304.2402	-17.4	-5.3	5.0	$C_{20} H_{32} O_2$
305.2413	1.3					
306.2553	24.2	306.2559	-1.9	-0.6	4.0	$C_{20} H_{34} O_2$
307.2587	6.1	307.2637	-16.2	-5.0	3.5	$C_{20} H_{35} O_2$

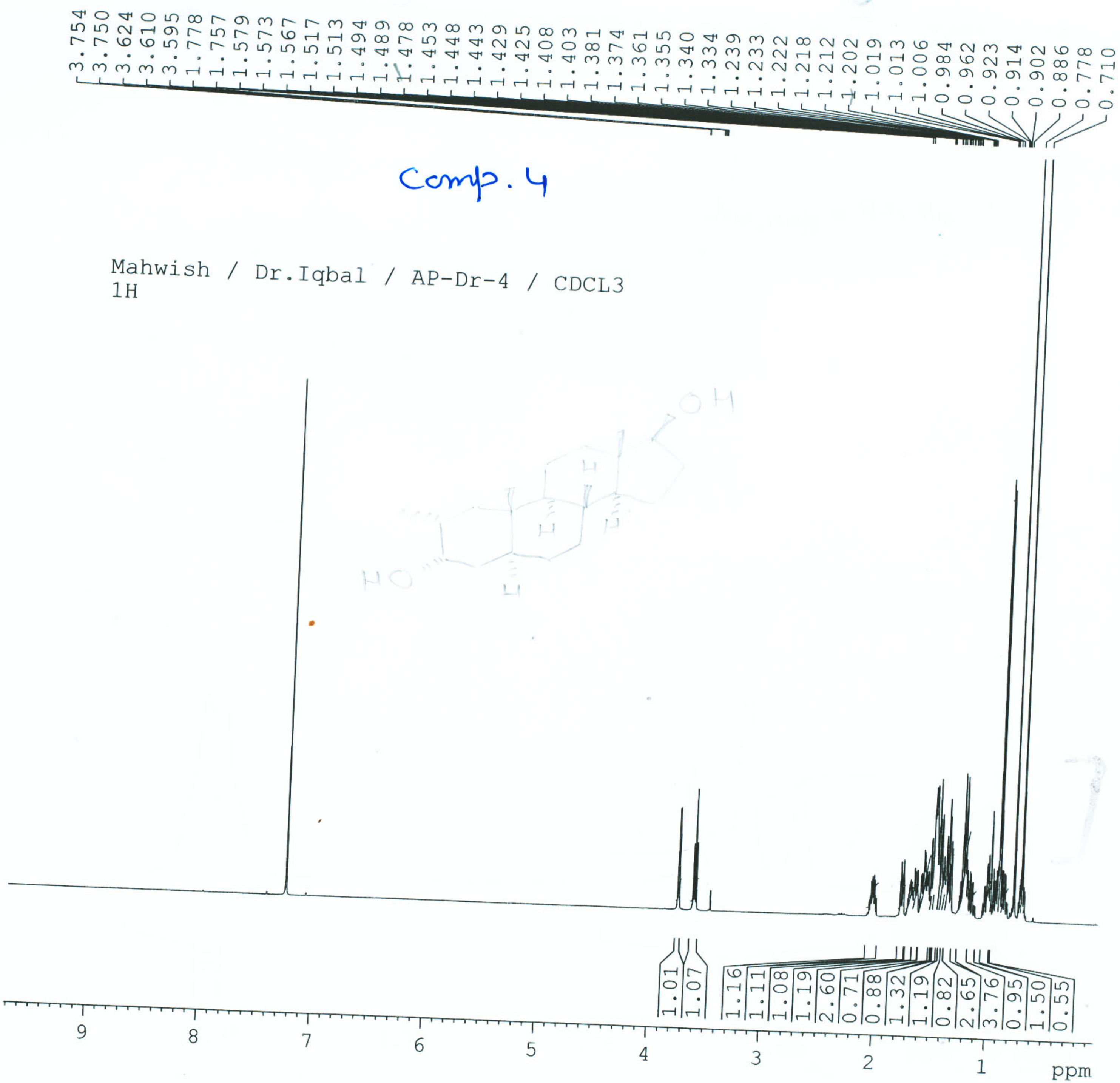

AVANCE AV[illegible]
CRYO[illegible]
LAB[illegible]

NAME	june26-15
EXPNO	6
PROCNO	1
Date_	20150626
Time	10.30
INSTRUM	spect
PROBHD	5 mm CPTCI 1H-
PULPROG	zg30
TD	32768
SOLVENT	CDCl3
NS	32
DS	0
SWH	12019.230 Hz
FIDRES	0.366798 Hz
AQ	1.3632404 sec
RG	9
DW	41.600 usec
DE	6.50 usec
TE	298.1 K
D1	2.00000000 sec
TD0	1

======== CHANNEL f1 ========

NUC1	1H
P1	8.00 usec
PL1	3.31 dB
PL1W	6.79873323 W
SFO1	600.0348002 MHz
SI	32768
SF	600.0300256 MHz
WDW	EM
SSB	0
LB	0.30 Hz
GB	0
PC	1.40

Comp. 4

AVANCE AV[illegible]
CRYOPR[illegible]
LAB NO: [illegible]

Mahwish / Dr.Iqbal / AP-Dr-4 / CDCL3
BB

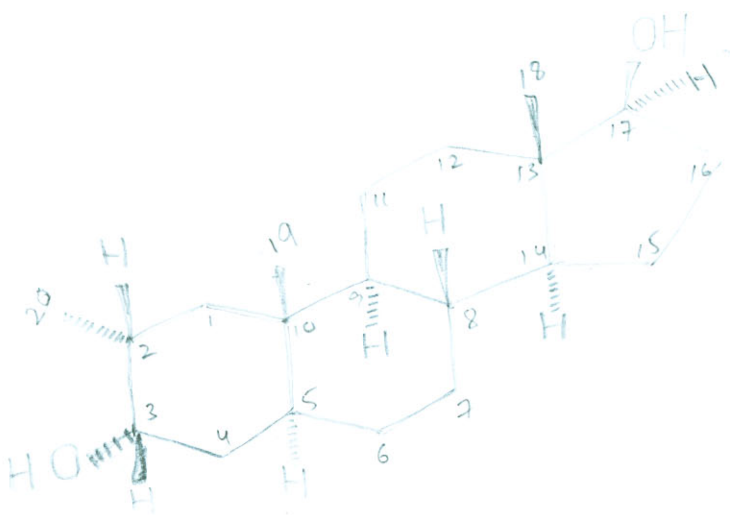

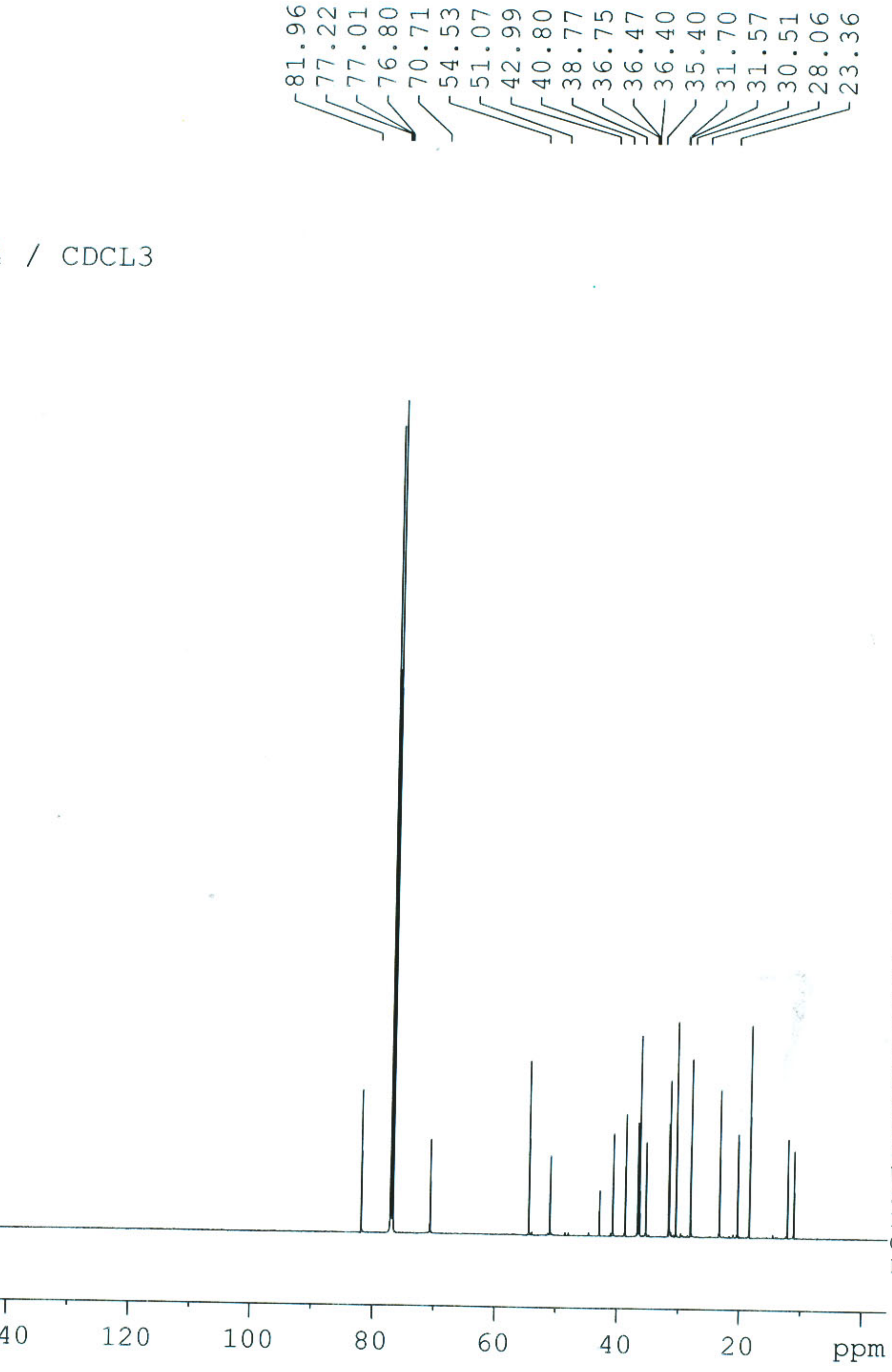

NAME	june26-15
EXPNO	11
PROCNO	1
Date_	20150627
Time	3.40
INSTRUM	spect
PROBHD	5 mm CPTCI 1H-
PULPROG	zgpg
TD	32768
SOLVENT	CDCl3
NS	10195
DS	4
SWH	35971.223 Hz
FIDRES	1.097755 Hz
AQ	0.4555391 sec
RG	32768
DW	13.900 usec
DE	6.50 usec
TE	298.0 K
D1	2.00000000 sec
D11	0.03000000 sec
TD0	12

======== CHANNEL f1 ========

NUC1	13C
P1	12.70 usec
PL1	-1.81 dB
PL1W	81.92915344 W
SFO1	150.8950149 MHz

======== CHANNEL f2 ========

CPDPRG2	waltz16
NUC2	1H
PCPD2	80.00 usec
PL2	3.31 dB
PL12	23.31 dB
PL13	22.50 dB
PL2W	6.79873323 W
PL12W	0.06798734 W
PL13W	0.08192718 W
SFO2	600.0336002 MHz
SI	16384
SF	150.8776659 MHz
WDW	EM
SSB	0
LB	1.00 Hz
GB	0
PC	1.00

Comp. 4

AVANCE AV-600-LC
CRYOPROBE
LAB NO: 108

Mahwish / Dr.Iqbal / AP-Dr-4 / CDCL3
deptsp 90

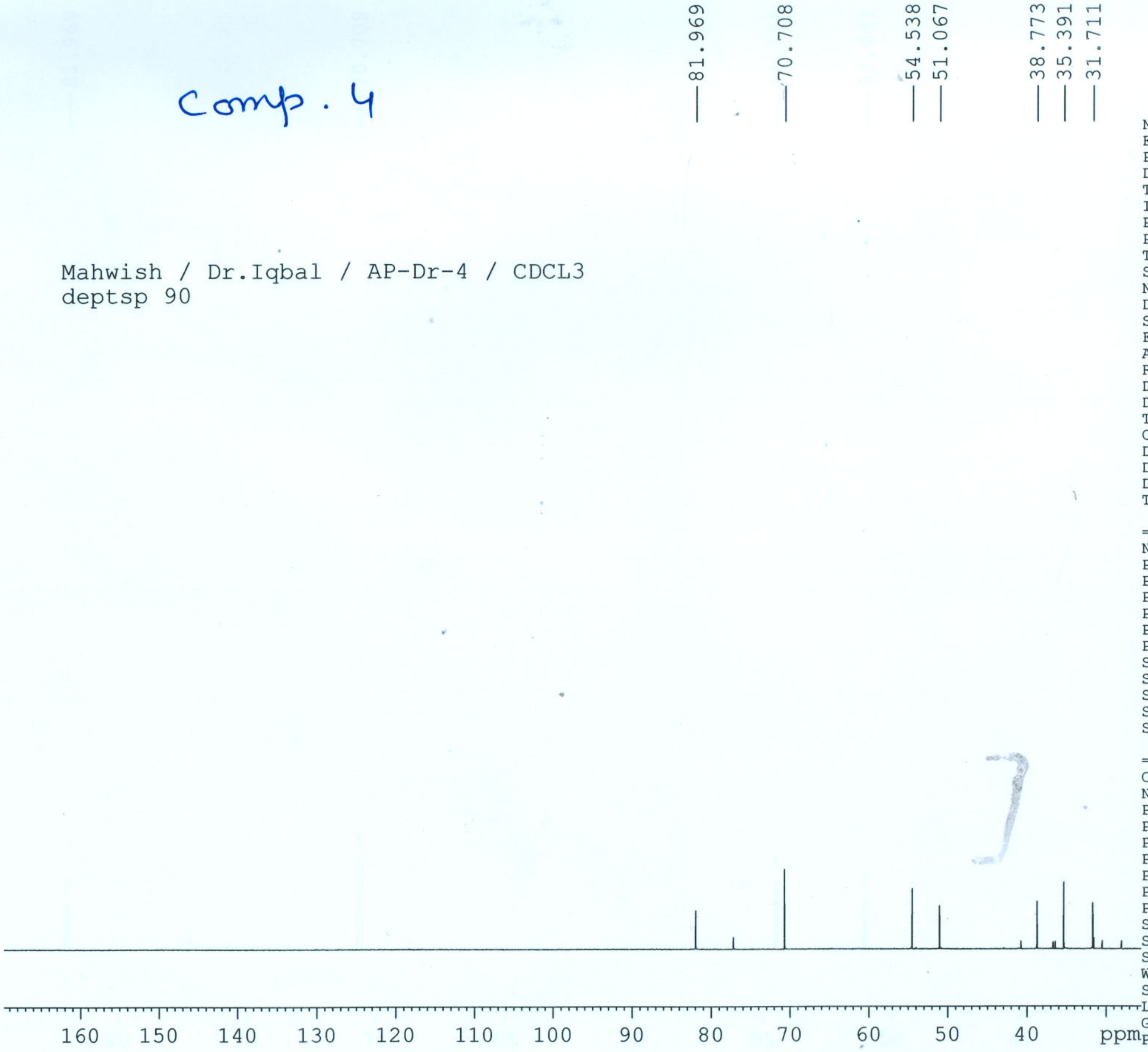

NAME	june26-15
EXPNO	13
PROCNO	1
Date_	20150627
Time	11.45
INSTRUM	spect
PROBHD	5 mm CPTCI 1H-
PULPROG	deptsp90
TD	32768
SOLVENT	CDCl3
NS	1286
DS	2
SWH	30303.031 Hz
FIDRES	0.924775 Hz
AQ	0.5407385 sec
RG	32768
DW	16.500 usec
DE	6.50 usec
TE	298.1 K
CNST2	145.0000000
D1	1.50000000 sec
D2	0.00344828 sec
D12	0.00002000 sec
TD0	3

======== CHANNEL f1 ========

NUC1	13C
P1	12.70 usec
P12	2000.00 usec
PL0	120.00 dB
PL1	-1.81 dB
PL0W	0.00000000 W
PL1W	81.92915344 W
SFO1	150.8927518 MHz
SP2	4.19 dB
SPNAM2	Crp60comp.4
SPOAL2	0.500
SPOFFS2	0.00 Hz

======== CHANNEL f2 ========

CPDPRG2	waltz16
NUC2	1H
P3	8.00 usec
P4	16.00 usec
PCPD2	80.00 usec
PL2	3.31 dB
PL12	23.31 dB
PL2W	6.79873323 W
PL12W	0.06798734 W
SFO2	600.0330002 MHz
SI	16384
SF	150.8776659 MHz
WDW	EM
SSB	0
LB	1.00 Hz
GB	0
PC	1.40

Comp. 4

AVANCE AV 600-LC
CRYOPROBE
LAB NO: 108

Mahwish / Dr.Iqbal / AP-Dr-4 / CDCL3
dept135

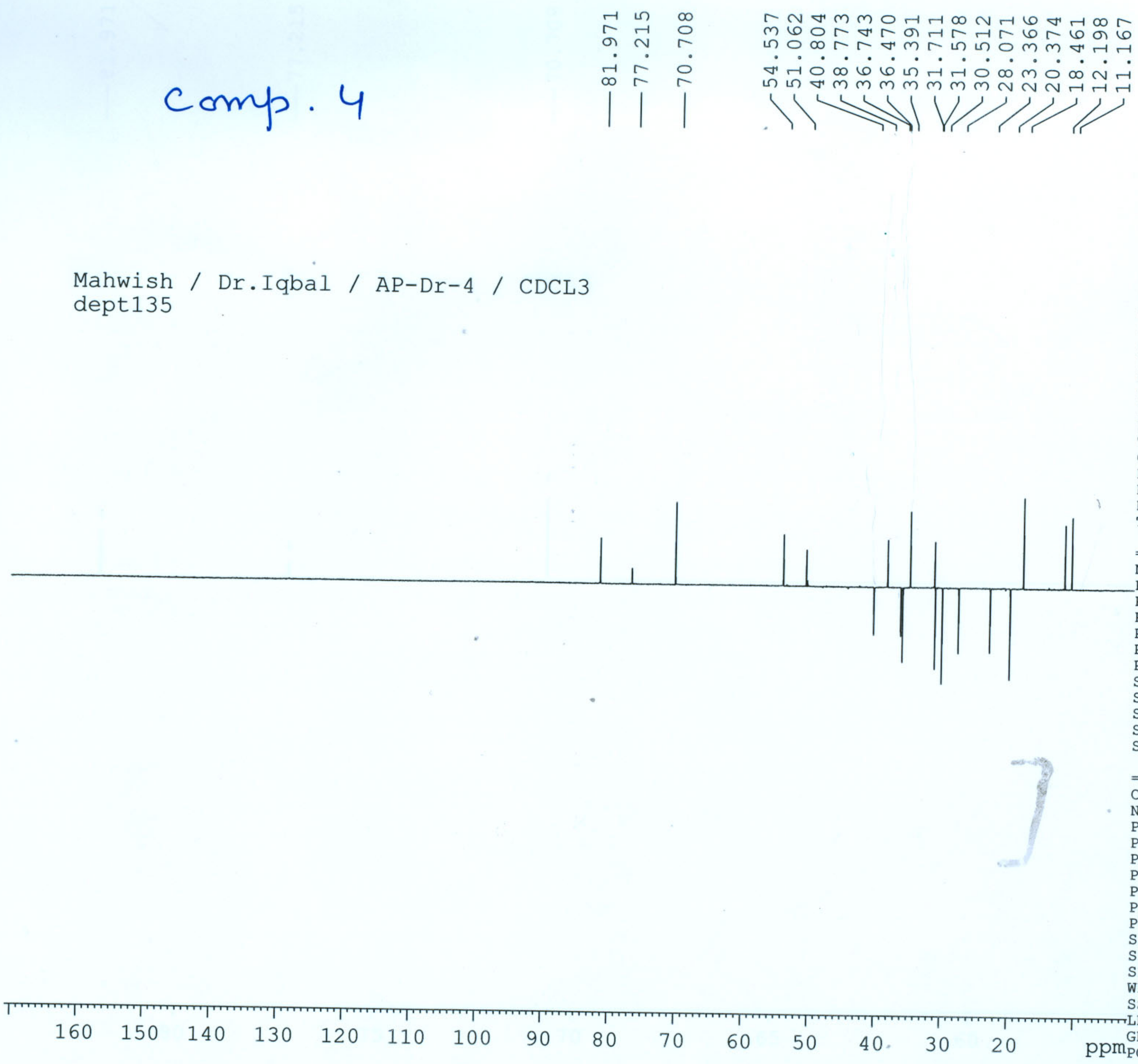

NAME	june26-15
EXPNO	12
PROCNO	1
Date_	20150627
Time	10.41
INSTRUM	spect
PROBHD	5 mm CPTCI 1H-
PULPROG	deptsp135
TD	32768
SOLVENT	CDCl3
NS	1837
DS	2
SWH	30303.031 Hz
FIDRES	0.924775 Hz
AQ	0.5407385 sec
RG	32768
DW	16.500 usec
DE	6.50 usec
TE	298.0 K
CNST2	145.0000000
D1	1.50000000 sec
D2	0.00344828 sec
D12	0.00002000 sec
TD0	6
======== CHANNEL f1 ========	
NUC1	13C
P1	12.70 usec
P12	2000.00 usec
PL0	120.00 dB
PL1	-1.81 dB
PL0W	0.00000000 W
PL1W	81.92915344 W
SFO1	150.8927518 MHz
SP2	4.19 dB
SPNAM2	Crp60comp.4
SPOAL2	0.500
SPOFFS2	0.00 Hz
======== CHANNEL f2 ========	
CPDPRG2	waltz16
NUC2	1H
P3	8.00 usec
P4	16.00 usec
PCPD2	80.00 usec
PL2	3.31 dB
PL12	23.31 dB
PL2W	6.79873323 W
PL12W	0.06798734 W
SFO2	600.0330002 MHz
SI	16384
SF	150.8776659 MHz
WDW	EM
SSB	0
LB	1.00 Hz
GB	0
PC	1.40

HSQC

Comp. 4

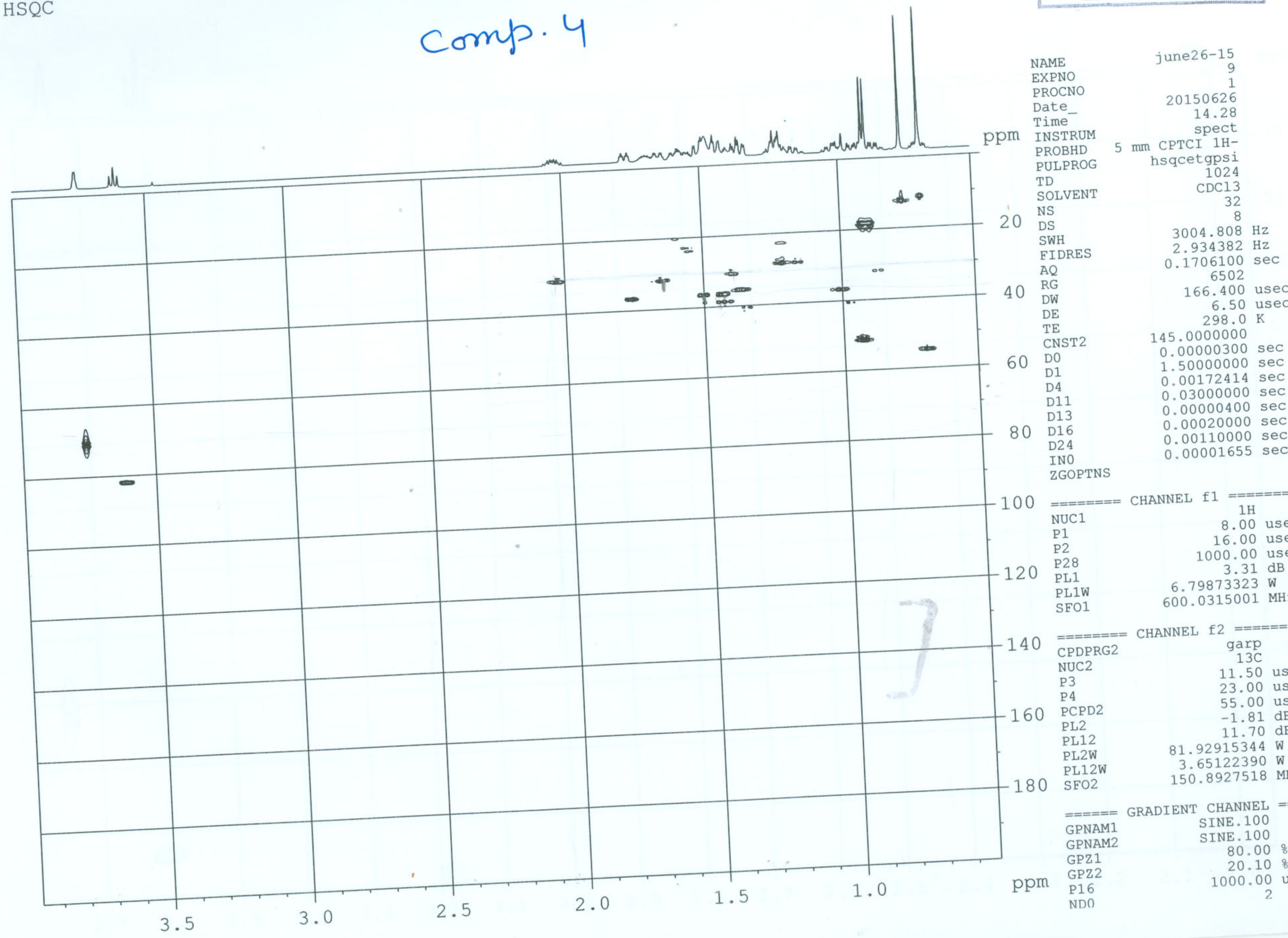

NAME	june26-15
EXPNO	9
PROCNO	1
Date_	20150626
Time	14.28
INSTRUM	spect
PROBHD	5 mm CPTCI 1H-
PULPROG	hsqcetgpsi
TD	1024
SOLVENT	CDCl3
NS	32
DS	8
SWH	3004.808 Hz
FIDRES	2.934382 Hz
AQ	0.1706100 sec
RG	6502
DW	166.400 usec
DE	6.50 usec
TE	298.0 K
CNST2	145.0000000
D0	0.00000300 sec
D1	1.50000000 sec
D4	0.00172414 sec
D11	0.03000000 sec
D13	0.00000400 sec
D16	0.00020000 sec
D24	0.00110000 sec
IN0	0.00001655 sec
ZGOPTNS	

======== CHANNEL f1 ========

NUC1	1H
P1	8.00 use
P2	16.00 use
P28	1000.00 use
PL1	3.31 dB
PL1W	6.79873323 W
SFO1	600.0315001 MH

======== CHANNEL f2 ======

CPDPRG2	garp
NUC2	13C
P3	11.50 us
P4	23.00 us
PCPD2	55.00 us
PL2	-1.81 dB
PL12	11.70 dB
PL2W	81.92915344 W
PL12W	3.65122390 W
SFO2	150.8927518 MH

====== GRADIENT CHANNEL =

GPNAM1	SINE.100
GPNAM2	SINE.100
GPZ1	80.00 %
GPZ2	20.10 %
P16	1000.00 u
ND0	2

Mahwish / Dr.Iqbal / AP-Dr-4 / CDCL3
HMBC

comp. 4

AVANCE AV 600-[illegible]
CRYOPROBE
LAB NO: 108

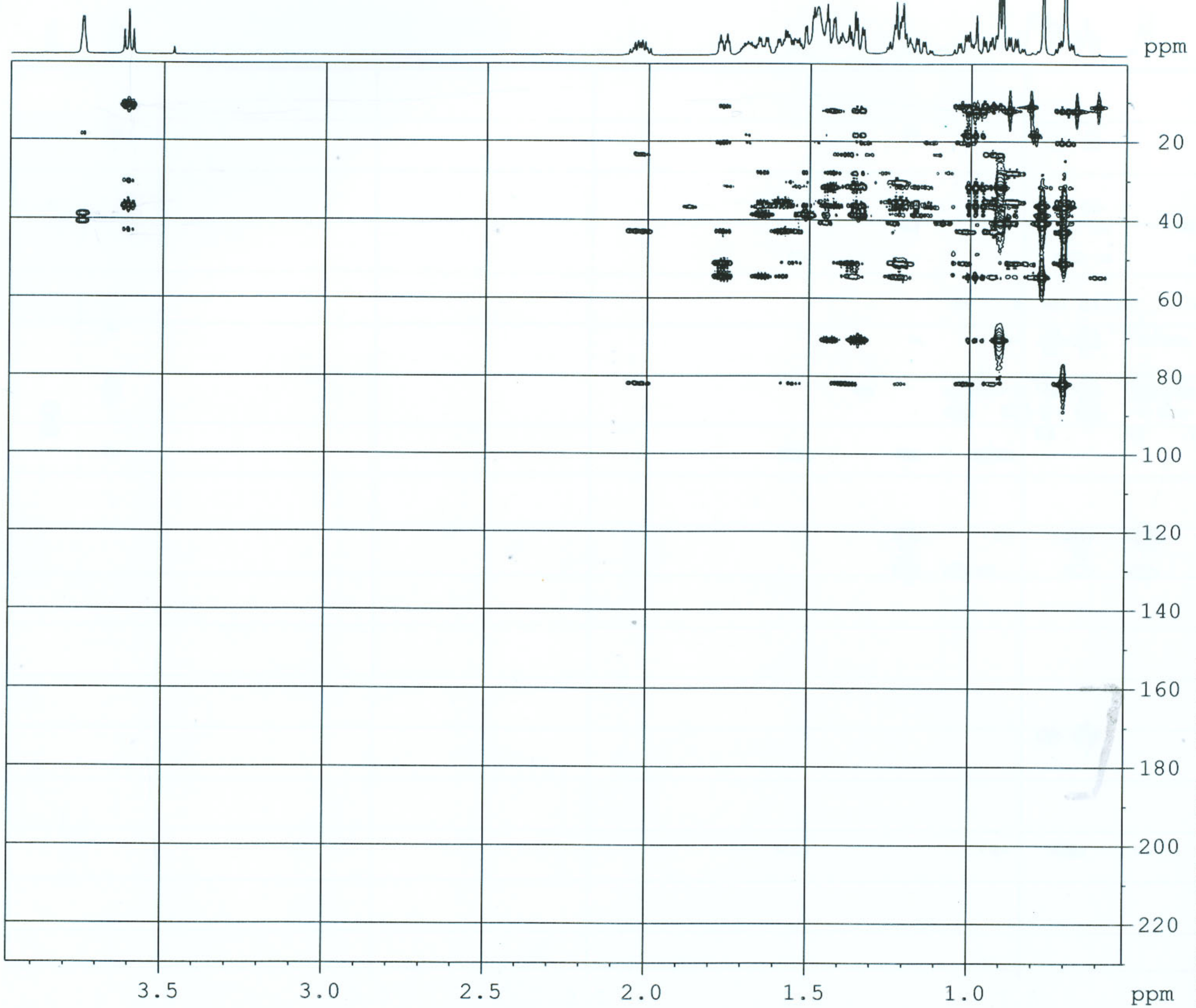

NAME	june26-15	
EXPNO	10	
PROCNO	1	
Date_	20150626	
Time	18.21	
INSTRUM	spect	
PROBHD	5 mm CPTCI 1H-	
PULPROG	hmbcgplpndqf	
TD	2048	
SOLVENT	CDCl3	
NS	64	
DS	16	
SWH	3004.808	Hz
FIDRES	1.467191	Hz
AQ	0.3410036	sec
RG	41285.1	
DW	166.400	usec
DE	6.50	usec
TE	298.0	K
CNST2	145.0000000	
CNST13	13.0000000	
D0	0.00000300	sec
D1	1.50000000	sec
D2	0.00344828	sec
D6	0.03846154	sec
D16	0.00015000	sec
IN0	0.00001440	sec

======== CHANNEL f1 ========

NUC1	1H	
P1	8.00	usec
P2	16.00	usec
PL1	3.31	dB
PL1W	6.79873323	W
SFO1	600.0315001	MHz

======== CHANNEL f2 ========

NUC2	13C	
P3	11.50	usec
PL2	-1.81	dB
PL2W	81.92915344	W
SFO2	150.8950149	MHz

====== GRADIENT CHANNEL =====

GPNAM1	SINE.100	
GPNAM2	SINE.100	
GPNAM3	SINE.100	
GPZ1	50.00	%
GPZ2	30.00	%
GPZ3	40.10	%
P16	2000.00	usec
ND0	2	
TD	256	
SFO1	150.895	MHz
FIDRES	135.569733	Hz
SW	230.000	ppm
FnMODE	QF	
SI	2048	

Mahwish / Dr.Iqbal / AP-Dr-4 / CDCL3
cosy

comp. 4

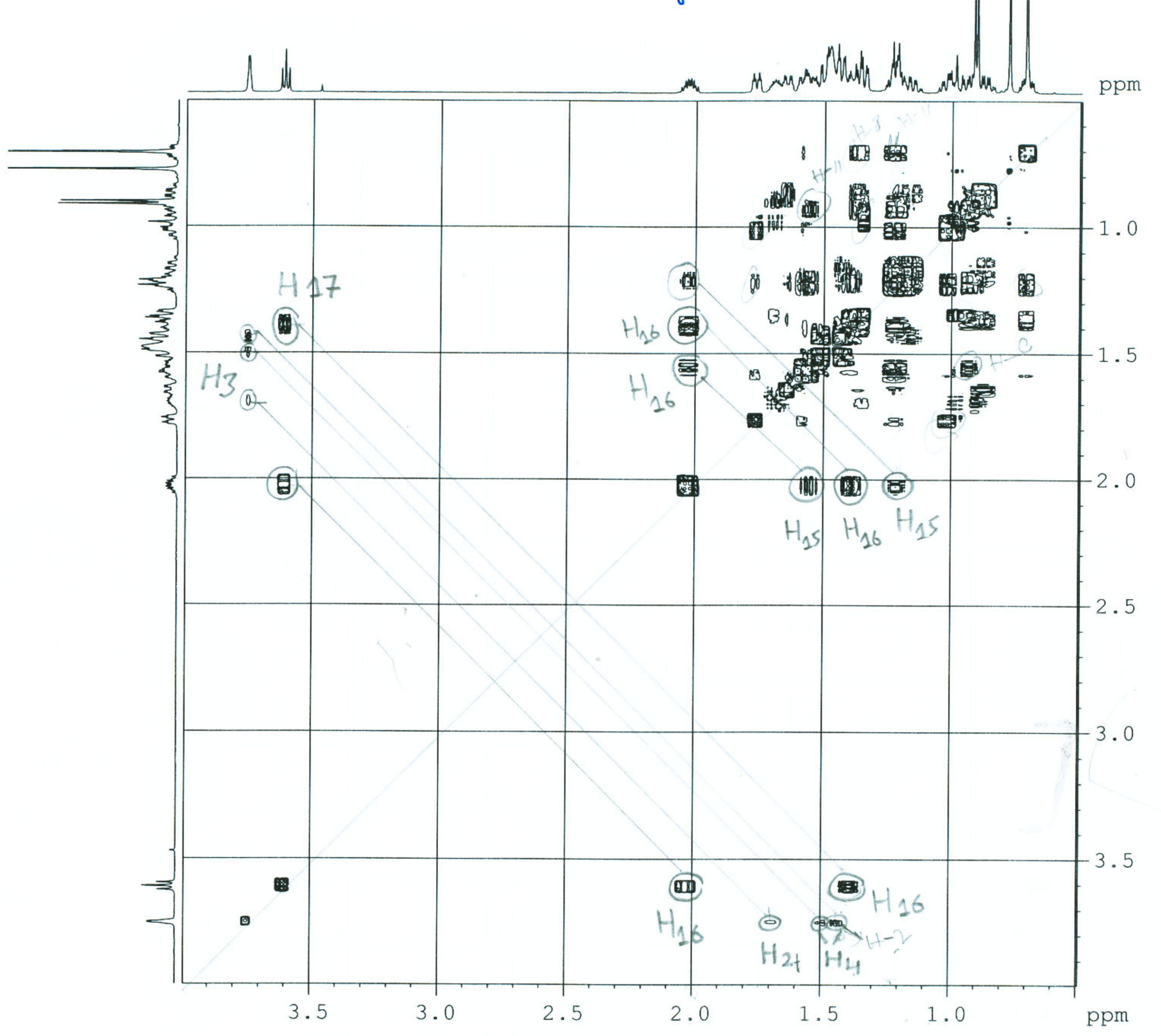

NAME	june26-15	
EXPNO	7	
PROCNO	1	
Date_	20150626	
Time	10.44	
INSTRUM	spect	
PROBHD	5 mm CPTCI 1H-	
PULPROG	cosydfqf	
TD	2048	
SOLVENT	CDCl3	
NS	8	
DS	4	
SWH	3004.808	Hz
FIDRES	1.467191	Hz
AQ	0.3410036	sec
RG	22.6	
DW	166.400	usec
DE	6.50	usec
TE	298.0	K
D0	0.00000300	sec
D1	2.00000000	sec
D13	0.00000400	sec
D20	0.00000200	sec
IN0	0.00033280	sec

======== CHANNEL f1 ========

NUC1	1H	
P1	8.00	usec
PL1	3.31	dB
PL1W	6.79873323	W
SFO1	600.0315001	MHz
ND0	1	
TD	128	
SFO1	600.0315	MHz
FIDRES	23.475063	Hz
SW	5.008	ppm
FnMODE	QF	
SI	1024	
SF	600.0300256	MHz
WDW	QSINE	
SSB	0	
LB	0.00	Hz
GB	0	
PC	4.00	
SI	1024	
MC2	QF	
SF	600.0300256	MHz
WDW	QSINE	
SSB	0	
LB	0.00	Hz
GB	0	

Mahwish / Dr.Iqbal / AP-Dr-4 / CDCL3
NOESY

comp. 4

AVANCE AV-600-LC
CRYO[illegible]
LA[illegible] 108

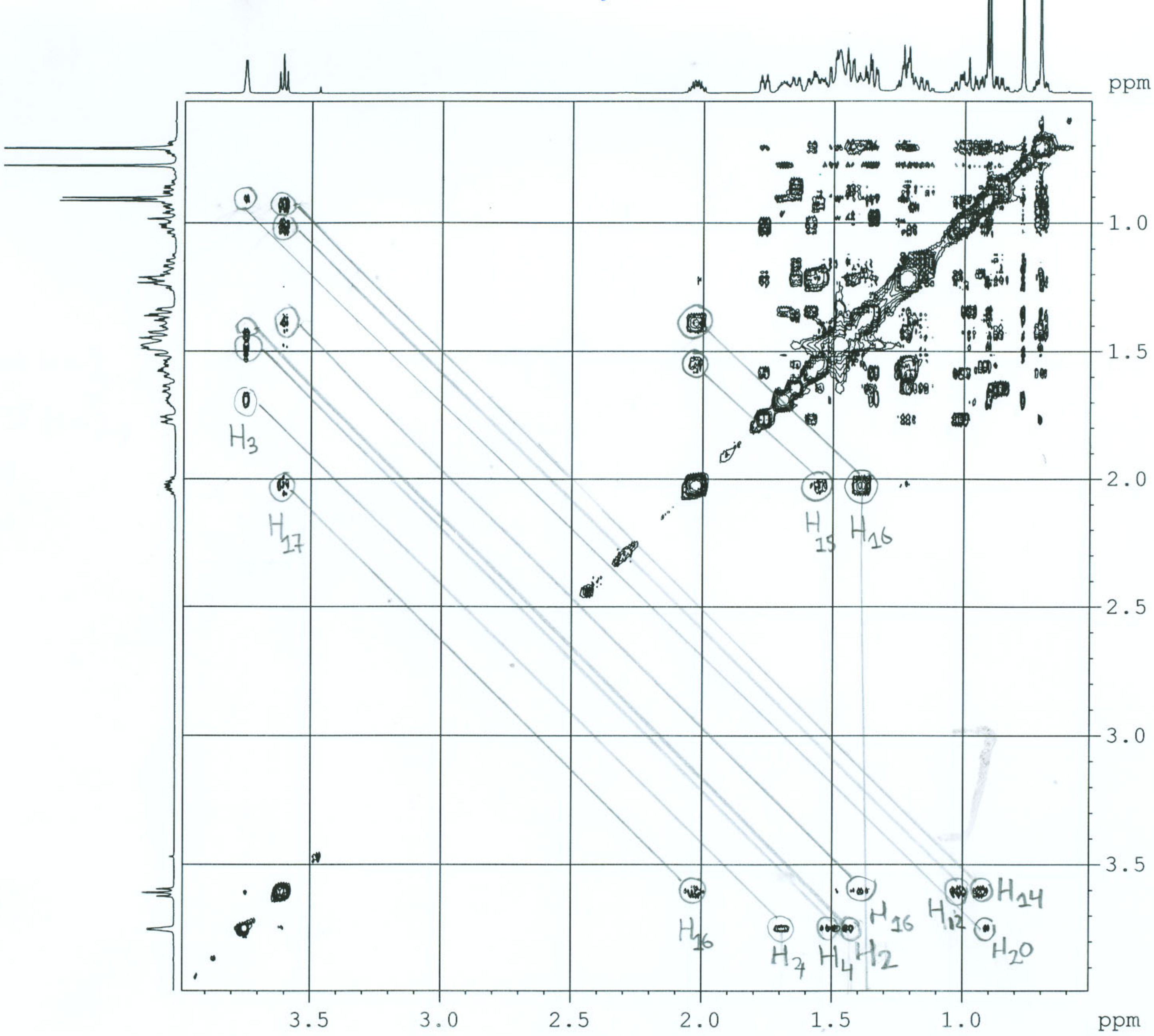

NAME	june26-15	
EXPNO	8	
PROCNO	1	
Date_	20150626	
Time	11.25	
INSTRUM	spect	
PROBHD	5 mm CPTCI 1H-	
PULPROG	noesygpph	
TD	2048	
SOLVENT	CDCl3	
NS	16	
DS	4	
SWH	3004.808	Hz
FIDRES	1.467191	Hz
AQ	0.3410036	sec
RG	25.4	
DW	166.400	usec
DE	6.50	usec
TE	298.0	K
D0	0.00015621	sec
D1	1.50000000	sec
D8	0.80000001	sec
D16	0.00020000	sec
IN0	0.00033280	sec
======== CHANNEL f1 ========		
NUC1	1H	
P1	8.00	usec
P2	16.00	usec
PL1	3.31	dB
PL1W	6.79873323	W
SFO1	600.0315001	MHz
====== GRADIENT CHANNEL =====		
GPNAM1	SINE.100	
GPNAM2	SINE.100	
GPZ1	40.00	%
GPZ2	-40.00	%
P16	1000.00	usec
ND0	1	
TD	256	
SFO1	600.0315	MHz
FIDRES	11.737532	Hz
SW	5.008	ppm
FnMODE	States-TPPI	
SI	1024	
SF	600.0300256	MHz
WDW	SINE	
SSB	2	
LB	0.00	Hz
GB	0	
PC	4.00	
SI	512	
MC2	States-TPPI	
SF	600.0300256	MHz
WDW	SINE	
SSB	2	
LB	0.00	Hz
GB	0	
