# Supplementary material for: Bio-Catalytic Structural Transformation of Anti-cancer Steroid, Drostanolone Enanthate with Cephalosporium aphidicola and Fusarium lini, and Cytotoxic Potential Evaluation of Its Metabolites against Certain Cancer Cell Lines
Source: Front Pharmacol. 2017 Dec 20;8:900. doi: 10.3389/fphar.2017.00900 (PMC5742531; doi:10.3389/fphar.2017.00900)
Supplement: Supplementary file 5 [file DataSheet5.PDF]

File: DR-17-9 Date Run: 12-04-2015 (Time Run: 11:52:50)
Sample: MAHWISH / DR. M. IQBAL
Instrument: JEOL MS 600H-1

Ionization mode: EI+

Comp. 5

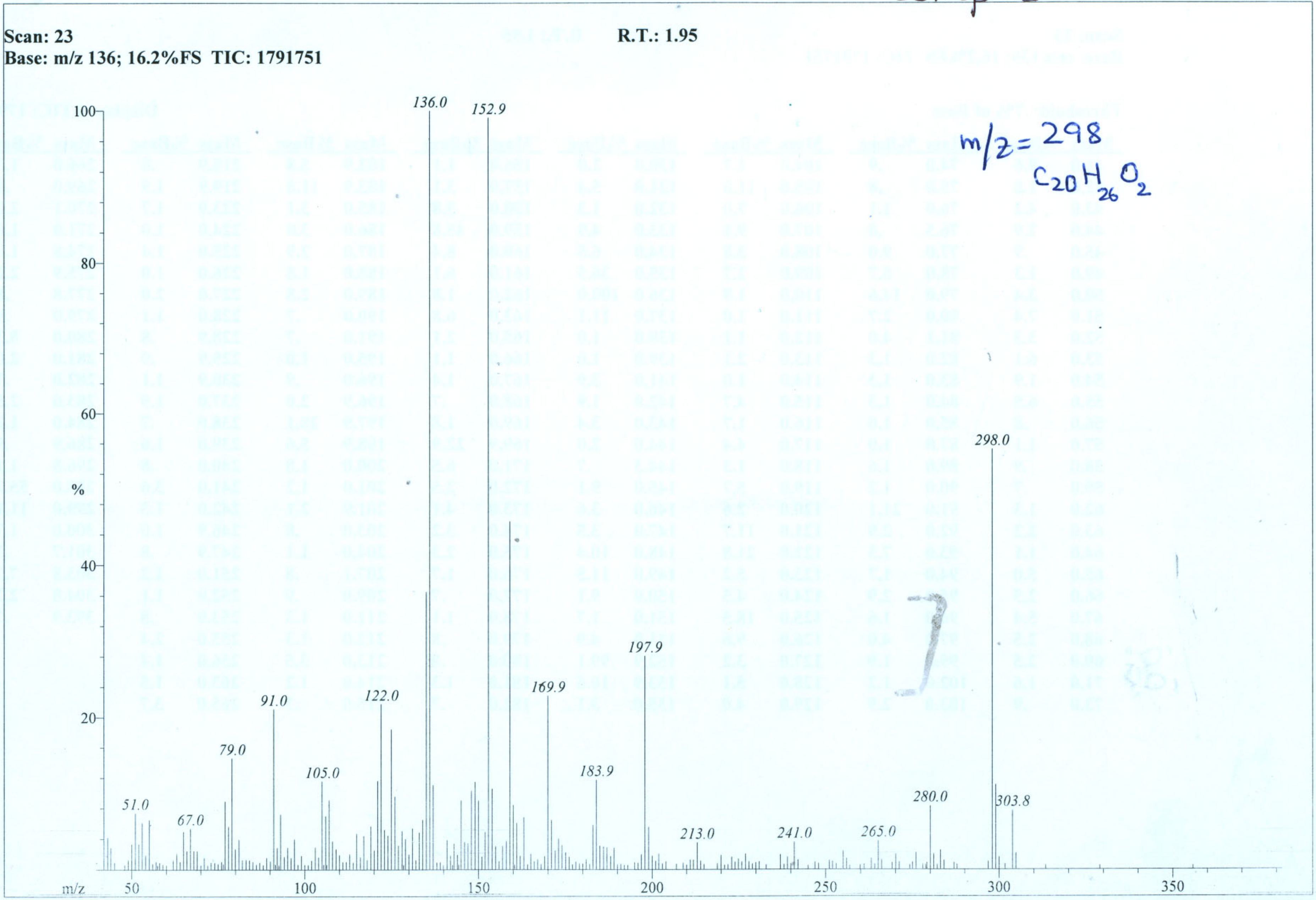

comp - 5

| Mass | Relative Intensity | Theoretical Mass | Delta [ppm] | Delta [mmu] | RDB | Composition |
|---|---|---|---|---|---|---|
| 149.0239 | 7.1 | 149.0239 | 0.1 | 0.0 | 6.5 | $C_8 H_5 O_3$ |
| 149.0961 | 15.2 | 149.0966 | -3.9 | -0.6 | 4.5 | $C_{10} H_{13} O_1$ |
| 149.1327 | 1.4 | 149.1330 | -2.1 | -0.3 | 3.5 | $C_{11} H_{17}$ |
| 150.1043 | 11.0 | 150.1045 | -1.0 | -0.1 | 4.0 | $C_{10} H_{14} O_1$ |
| 151.1081 | 1.3 | 151.1123 | -27.4 | -4.1 | 3.5 | $C_{10} H_{15} O_1$ |
| 155.0865 | 1.3 | 155.0861 | 2.7 | 0.4 | 7.5 | $C_{12} H_{11}$ |
| 157.1019 | 2.0 | 157.1017 | 1.1 | 0.2 | 6.5 | $C_{12} H_{13}$ |
| 158.1088 | 3.4 | 158.1096 | -4.9 | -0.8 | 6.0 | $C_{12} H_{14}$ |
| 159.0818 | 2.7 | 159.0810 | 5.3 | 0.8 | 6.5 | $C_{11} H_{11} O_1$ |
| 159.1173 | 58.4 | 159.1174 | -0.4 | -0.1 | 5.5 | $C_{12} H_{15}$ |
| 160.0879 | 1.7 | 160.0888 | -5.9 | -0.9 | 6.0 | $C_{11} H_{12} O_1$ |
| 160.1203 | 7.3 | 160.1252 | -30.5 | -4.9 | 5.0 | $C_{12} H_{16}$ |
| 161.0959 | 5.2 | 161.0966 | -4.6 | -0.7 | 5.5 | $C_{11} H_{13} O_1$ |
| 161.1284 | 1.3 | 161.1330 | -28.8 | -4.6 | 4.5 | $C_{12} H_{17}$ |
| 162.1025 | 1.5 | 162.1045 | -12.3 | -2.0 | 5.0 | $C_{11} H_{14} O_1$ |
| 163.1115 | 5.9 | 163.1123 | -5.1 | -0.8 | 4.5 | $C_{11} H_{15} O_1$ |
| 167.0327 | 2.3 | 167.0344 | -10.5 | -1.8 | 5.5 | $C_8 H_7 O_4$ |
| 169.1006 | 1.0 | 169.1017 | -6.6 | -1.1 | 7.5 | $C_{13} H_{13}$ |
| 171.0802 | 1.5 | 171.0810 | -4.8 | -0.8 | 7.5 | $C_{12} H_{11} O_1$ |
| 171.1160 | 2.3 | 171.1174 | -7.8 | -1.3 | 6.5 | $C_{13} H_{15}$ |
| 172.0878 | 1.2 | 172.0888 | -6.1 | -1.0 | 7.0 | $C_{12} H_{12} O_1$ |
| 173.0957 | 2.9 | 173.0966 | -5.6 | -1.0 | 6.5 | $C_{12} H_{13} O_1$ |
| 173.1316 | 1.0 | 173.1330 | -8.4 | -1.5 | 5.5 | $C_{13} H_{17}$ |
| 174.1031 | 2.1 | 174.1045 | -8.0 | -1.4 | 6.0 | $C_{12} H_{14} O_1$ |
| 175.1106 | 2.0 | 175.1123 | -9.8 | -1.7 | 5.5 | $C_{12} H_{15} O_1$ |
| 179.0860 | 1.3 | 179.0861 | -0.3 | -0.0 | 9.5 | $C_{14} H_{11}$ |
| | | 179.0919 | -33.1 | -5.9 | 0.5 | $C_7 H_{15} O_5$ |
| 181.1006 | 1.9 | 181.1017 | -6.0 | -1.1 | 8.5 | $C_{14} H_{13}$ |
| 182.1080 | 1.2 | 182.1096 | -8.7 | -1.6 | 8.0 | $C_{14} H_{14}$ |
| 183.0814 | 1.4 | 183.0810 | 2.2 | 0.4 | 8.5 | $C_{13} H_{11} O_1$ |
| 183.1158 | 2.5 | 183.1174 | -8.8 | -1.6 | 7.5 | $C_{14} H_{15}$ |
| 184.1244 | 1.2 | 184.1252 | -4.1 | -0.8 | 7.0 | $C_{14} H_{16}$ |
| 185.0968 | 4.7 | 185.0966 | 0.8 | 0.2 | 7.5 | $C_{13} H_{13} O_1$ |
| 185.1319 | 2.0 | 185.1330 | -6.0 | -1.1 | 6.5 | $C_{14} H_{17}$ |
| 186.1036 | 2.8 | 186.1045 | -4.8 | -0.9 | 7.0 | $C_{13} H_{14} O_1$ |
| 187.1126 | 4.8 | 187.1123 | 1.4 | 0.3 | 6.5 | $C_{13} H_{15} O_1$ |
| 188.1190 | 1.8 | 188.1201 | -5.7 | -1.1 | 6.0 | $C_{13} H_{16} O_1$ |
| 189.1282 | 5.4 | 189.1279 | 1.3 | 0.3 | 5.5 | $C_{13} H_{17} O_1$ |
| 197.0961 | 1.2 | 197.0966 | -2.8 | -0.5 | 8.5 | $C_{14} H_{13} O_1$ |
| 199.1112 | 1.6 | 199.1123 | -5.6 | -1.1 | 7.5 | $C_{14} H_{15} O_1$ |
| 200.1192 | 1.3 | 200.1201 | -4.7 | -0.9 | 7.0 | $C_{14} H_{16} O_1$ |
| 201.1281 | 1.0 | 201.1279 | 1.0 | 0.2 | 6.5 | $C_{14} H_{17} O_1$ |
| 213.1280 | 1.7 | 213.1279 | 0.1 | 0.0 | 7.5 | $C_{15} H_{17} O_1$ |
| 227.1461 | 1.3 | 227.1436 | 11.0 | 2.5 | 7.5 | $C_{16} H_{19} O_1$ |
| 241.1595 | 2.6 | 241.1592 | 1.0 | 0.3 | 7.5 | $C_{17} H_{21} O_1$ |
| 255.1766 | 1.8 | 255.1749 | 6.8 | 1.7 | 7.5 | $C_{18} H_{23} O_1$ |
| 263.1800 | 1.4 | 263.1800 | 0.1 | 0.0 | 9.5 | $C_{20} H_{23}$ |
| | | 263.1858 | -22.3 | -5.9 | 0.5 | $C_{13} H_{27} O_5$ |
| 265.1605 | 3.5 | 265.1592 | 4.7 | 1.3 | 9.5 | $C_{19} H_{21} O_1$ |
| 270.1974 | 1.4 | 270.1984 | -3.5 | -0.9 | 7.0 | $C_{19} H_{26} O_1$ |
| 279.1644 | 1.1 | 279.1596 | 17.1 | 4.8 | 5.5 | $C_{16} H_{23} O_4$ |
| | | 279.1749 | -37.5 | -10.5 | 9.5 | $C_{20} H_{23} O_1$ |
| 280.1810 | 8.8 | 280.1827 | -6.1 | -1.7 | 9.0 | $C_{20} H_{24} O_1$ |
| 281.1855 | 2.1 | 281.1905 | -17.8 | -5.0 | 8.5 | $C_{20} H_{25} O_1$ |
| | | 281.1753 | 36.4 | 10.2 | 4.5 | $C_{16} H_{25} O_4$ |
| 283.1704 | 2.1 | 283.1698 | 2.1 | 0.6 | 8.5 | $C_{19} H_{23} O_2$ |
| 298.1944 | 53.1 | 298.1933 | 3.6 | 1.1 | 8.0 | $C_{20} H_{26} O_2$ |
| 299.1978 | 10.9 | 299.2011 | -11.0 | -3.3 | 7.5 | $C_{20} H_{27} O_2$ |
| 300.2016 | 1.4 | 300.2089 | -24.5 | -7.4 | 7.0 | $C_{20} H_{28} O_2$ |
| | | 300.1937 | 26.3 | 7.9 | 3.0 | $C_{16} H_{28} O_5$ |

MAHWISH/DR.IQBAL/DR-17-9/CD3OD

AVANCE AV-500
LAB NO:118

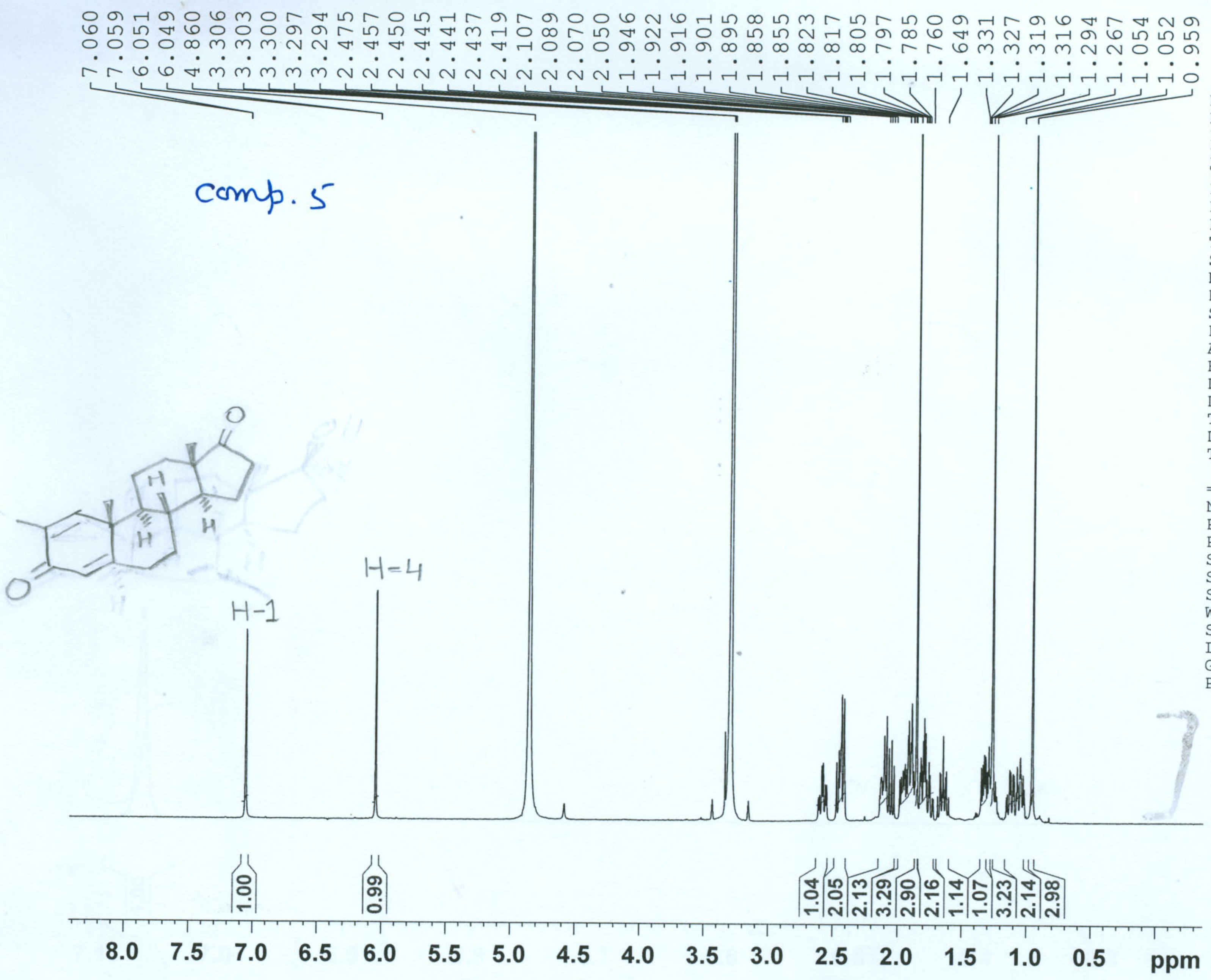


| | |
|---|---|
| NAME | dec03-15 |
| EXPNO | 4 |
| PROCNO | 1 |
| Date_ | 20151203 |
| Time | 15.19 |
| INSTRUM | spect |
| PROBHD | 5 mm PABBI 1H/ |
| PULPROG | zg30 |
| TD | 65536 |
| SOLVENT | MeOD |
| NS | 128 |
| DS | 0 |
| SWH | 10000.000 Hz |
| FIDRES | 0.152588 Hz |
| AQ | 3.2769001 sec |
| RG | 362 |
| DW | 50.000 usec |
| DE | 6.50 usec |
| TE | 297.0 K |
| D1 | 1.50000000 sec |
| TD0 | 1 |

======== CHANNEL f1 ========

| | |
|---|---|
| NUC1 | 1H |
| P1 | 8.03 usec |
| PL1 | 3.00 dB |
| SFO1 | 500.2342520 MHz |
| SI | 32768 |
| SF | 500.2300119 MHz |
| WDW | EM |
| SSB | 0 |
| LB | 0.30 Hz |
| GB | 0 |
| PC | 1.00 |

MAHWISH/DR.IQBAL/Dr-17-9/CD3OD
BB

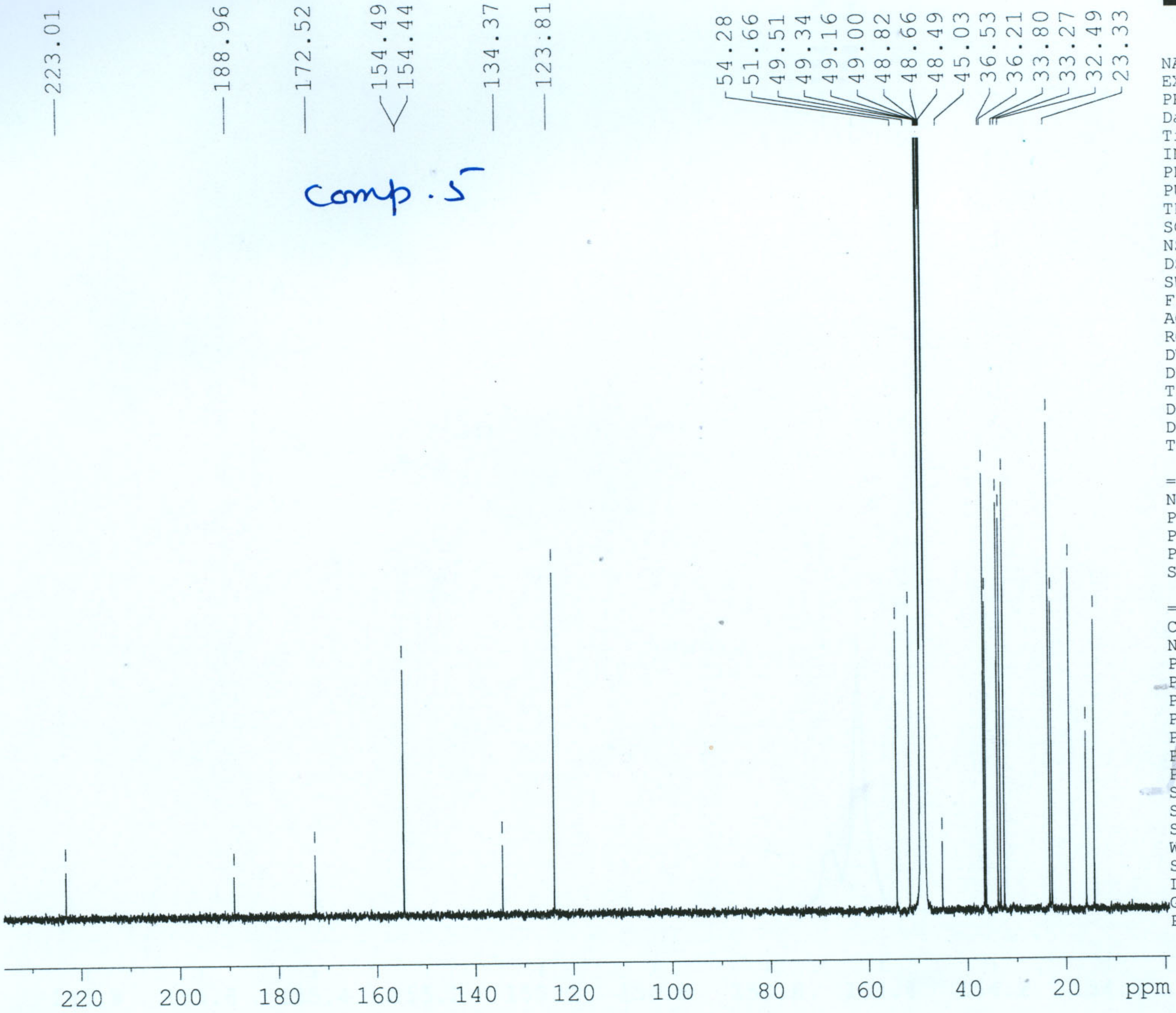


BRUKER

| | |
|---|---|
| NAME | Dec07-15 |
| EXPNO | 9 |
| PROCNO | 1 |
| Date_ | 20151208 |
| Time | 1.42 |
| INSTRUM | spect |
| PROBHD | 5 mm CPDUL 13C |
| PULPROG | zgpg |
| TD | 32768 |
| SOLVENT | MeOD |
| NS | 10240 |
| DS | 2 |
| SWH | 30303.031 Hz |
| FIDRES | 0.924775 Hz |
| AQ | 0.5407385 sec |
| RG | 32768 |
| DW | 16.500 usec |
| DE | 6.50 usec |
| TE | 298.0 K |
| D1 | 1.50000000 sec |
| D11 | 0.03000000 sec |
| TD0 | 10 |

======== CHANNEL f1 ========

| | |
|---|---|
| NUC1 | 13C |
| P1 | 10.30 usec |
| PL1 | 5.00 dB |
| PL1W | 21.04969788 W |
| SFO1 | 125.8226727 MHz |

======== CHANNEL f2 ========

| | |
|---|---|
| CPDPRG2 | waltz16 |
| NUC2 | 1H |
| PCPD2 | 80.00 usec |
| PL2 | -0.50 dB |
| PL12 | 13.11 dB |
| PL13 | 24.00 dB |
| PL2W | 14.09191513 W |
| PL12W | 0.61371964 W |
| PL13W | 0.05000000 W |
| SFO2 | 500.3335023 MHz |
| SI | 16384 |
| SF | 125.8079013 MHz |
| WDW | EM |
| SSB | 0 |
| LB | 1.50 Hz |
| GB | 0 |
| PC | 1.00 |

MAHWISH/DR.IQBAL/Dr-17-9/CD3OD
Dept-HSQC

Comp. 5

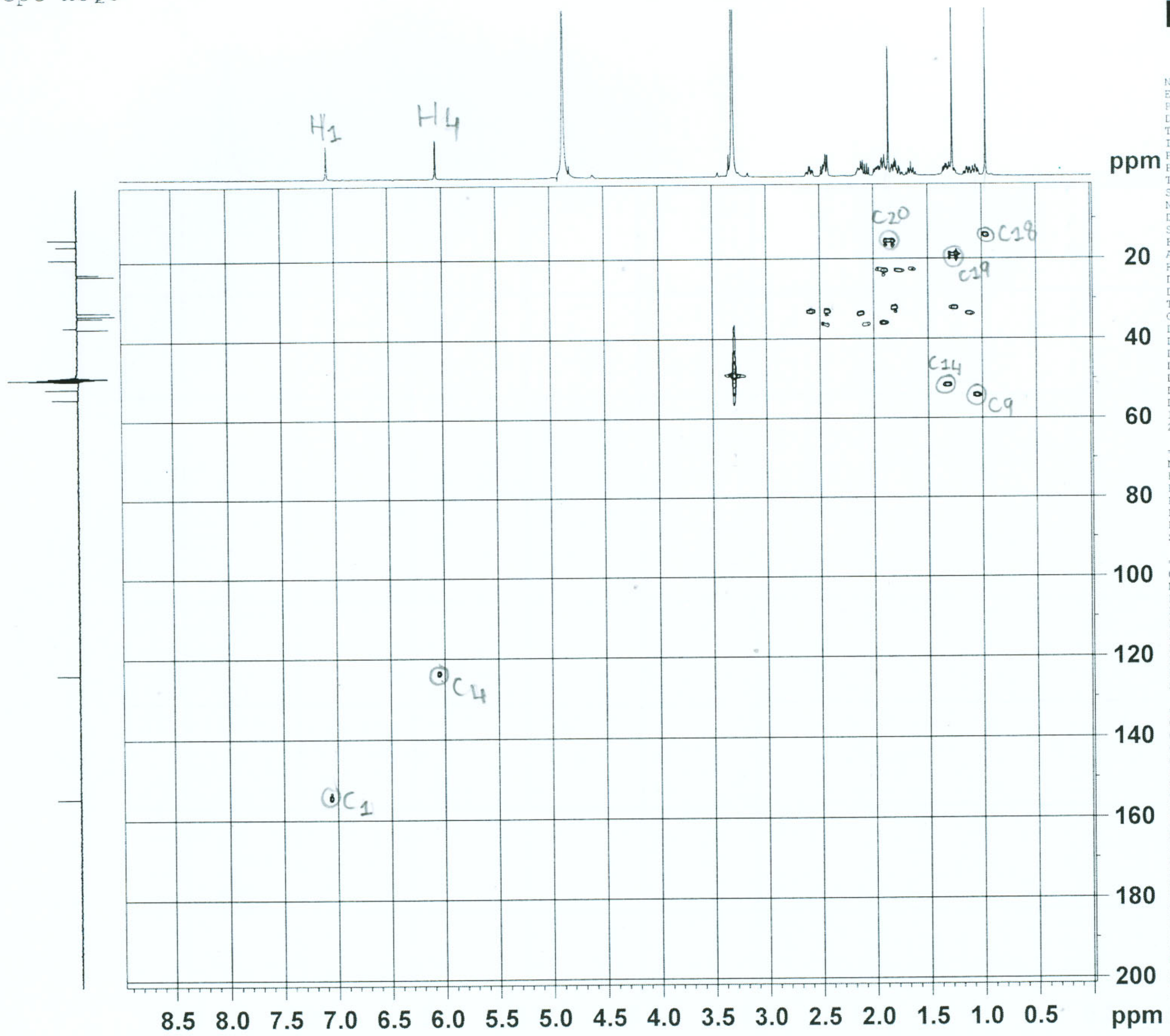


BRUKER

| | |
|---|---|
| NAME | Dec07-15 |
| EXPNO | 7 |
| PROCNO | 1 |
| Date_ | 20151207 |
| Time | 16.57 |
| INSTRUM | spect |
| PROBHD | 5 mm CPDUL 13C |
| PULPROG | hsqcedetgp |
| TD | 1024 |
| SOLVENT | MeOD |
| NS | 16 |
| DS | 16 |
| SWH | 4496.403 Hz |
| FIDRES | 4.391018 Hz |
| AQ | 0.1140300 sec |
| RG | 41285.1 |
| DW | 111.200 usec |
| DE | 6.50 usec |
| TE | 298.0 K |
| CNST2 | 145.0000000 |
| D0 | 0.00000300 sec |
| D1 | 2.00000000 sec |
| D4 | 0.00172414 sec |
| D11 | 0.03000000 sec |
| D13 | 0.00000400 sec |
| D16 | 0.00015000 sec |
| D21 | 0.00345000 sec |
| IN0 | 0.00001985 sec |
| ZGOPTNS | |

======== CHANNEL f1 ========

| | |
|---|---|
| NUC1 | 1H |
| P1 | 16.50 usec |
| P2 | 33.00 usec |
| P28 | 0.50 usec |
| PL1 | -0.50 dB |
| PL1W | 14.09191513 W |
| SFO1 | 500.3322515 MHz |

======== CHANNEL f2 ========

| | |
|---|---|
| CPDPRG2 | garp |
| NUC2 | 13C |
| P3 | 10.50 usec |
| P4 | 21.00 usec |
| PCPD2 | 65.00 usec |
| PL2 | 5.00 dB |
| PL12 | 20.83 dB |
| PL2W | 21.04969788 W |
| PL12W | 0.54985207 W |
| SFO2 | 125.8206598 MHz |

====== GRADIENT CHANNEL =====

| | |
|---|---|
| GPNAM1 | SINE.100 |
| GPNAM2 | SINE.100 |
| GPZ1 | 80.00 % |
| GPZ2 | 20.10 % |
| P16 | 1000.00 usec |
| ND0 | 2 |
| TD | 256 |
| SFO1 | 125.8207 MHz |
| FIDRES | 98.297394 Hz |
| SW | 200.000 ppm |
| FnMODE | Echo-Antiecho |
| SI | 1024 |
| SF | 500.3300143 MHz |
| WDW | QSINE |
| SSB | 2 |
| LB | 0.00 Hz |
| GB | 0 |
| PC | 1.00 |
| SI | 1024 |
| MC2 | echo-antiecho |
| SF | 125.8079013 MHz |
| WDW | QSINE |
| SSB | 2 |
| LB | 0.00 Hz |
| GB | 0 |

MAHWISH/DR.IQBAL/Dr-17-9/CD3OD
HMBC

Comp. 5

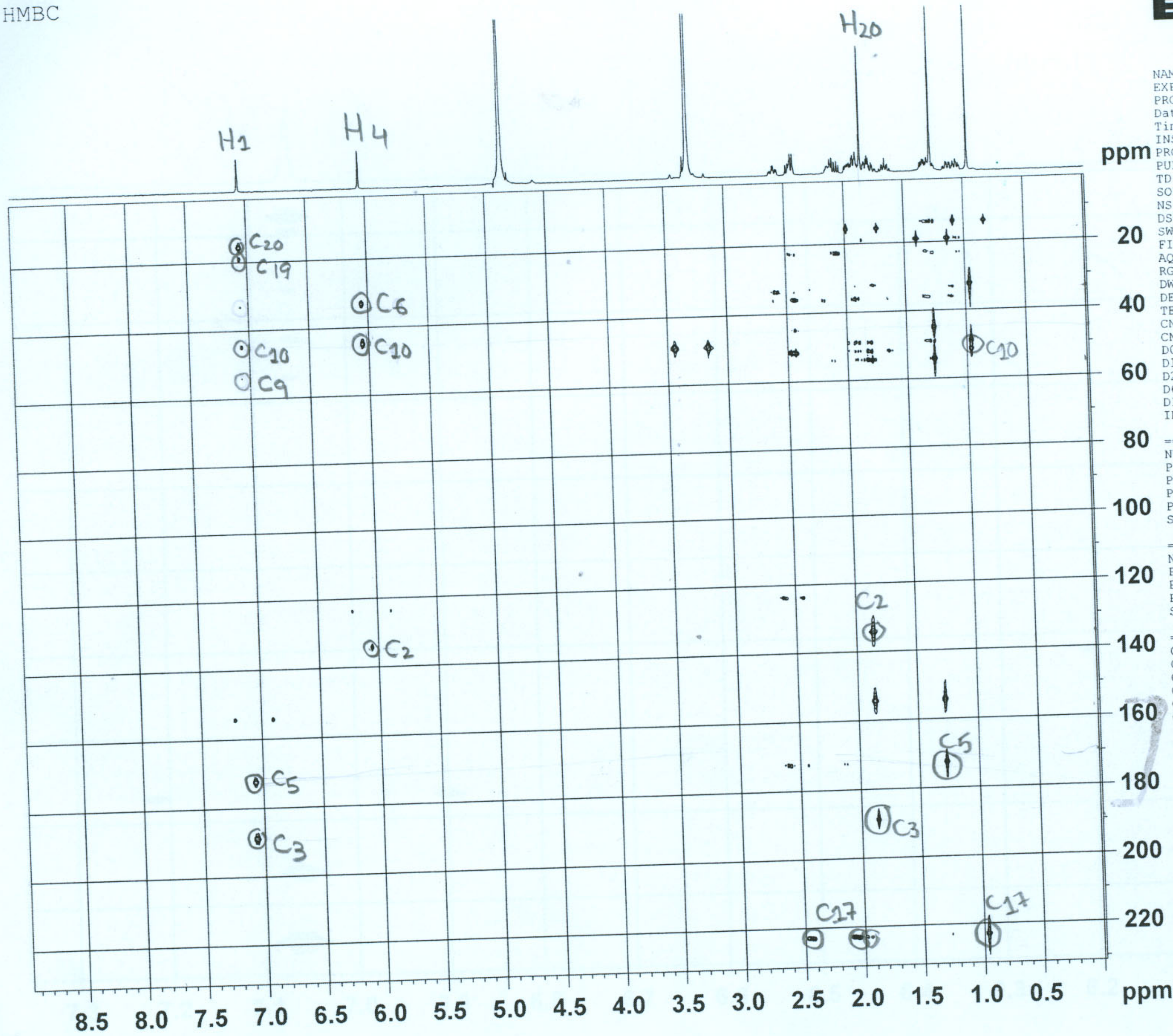


| | |
|---|---|
| NAME | Dec07-15 |
| EXPNO | 8 |
| PROCNO | 1 |
| Date_ | 20151207 |
| Time | 19.24 |
| INSTRUM | spect |
| PROBHD | 5 mm CPDUL 13C |
| PULPROG | hmbcgplpndqf |
| TD | 4096 |
| SOLVENT | MeOD |
| NS | 32 |
| DS | 4 |
| SWH | 4496.403 Hz |
| FIDRES | 1.097755 Hz |
| AQ | 0.4556364 sec |
| RG | 36780.8 |
| DW | 111.200 usec |
| DE | 6.50 usec |
| TE | 298.0 K |
| CNST2 | 145.0000000 |
| CNST13 | 13.0000000 |
| D0 | 0.00000300 sec |
| D1 | 2.00000000 sec |
| D2 | 0.00344828 sec |
| D6 | 0.03846154 sec |
| D16 | 0.00015000 sec |
| IN0 | 0.00001730 sec |

======== CHANNEL f1 ========

| | |
|---|---|
| NUC1 | 1H |
| P1 | 16.50 usec |
| P2 | 33.00 usec |
| PL1 | -0.50 dB |
| PL1W | 14.09191513 W |
| SFO1 | 500.3322515 MHz |

======== CHANNEL f2 ========

| | |
|---|---|
| NUC2 | 13C |
| P3 | 10.50 usec |
| PL2 | 5.00 dB |
| PL2W | 21.04969788 W |
| SFO2 | 125.8225469 MHz |

====== GRADIENT CHANNEL =====

| | |
|---|---|
| GPNAM1 | SINE.100 |
| GPNAM2 | SINE.100 |
| GPNAM3 | SINE.100 |
| GPZ1 | 50.00 % |
| GPZ2 | 30.00 % |
| GPZ3 | 40.10 % |
| P16 | 2000.00 usec |
| ND0 | 2 |
| TD | 256 |
| SFO1 | 125.8225 MHz |
| FIDRES | 113.043694 Hz |
| SW | 230.000 ppm |
| FnMODE | QF |
| SI | 2048 |
| SF | 500.3300143 MHz |
| WDW | QSINE |
| SSB | 2 |
| LB | 0.00 Hz |
| GB | 0 |
| PC | 4.00 |
| SI | 1024 |
| MC2 | QF |
| SF | 125.8079013 MHz |
| WDW | QSINE |
| SSB | 2 |
| LB | 0.00 Hz |
| GB | 0 |

Comp. 5

MAHWISH/DR.IQBAL/Dr-17-9/CD3OD
COSY

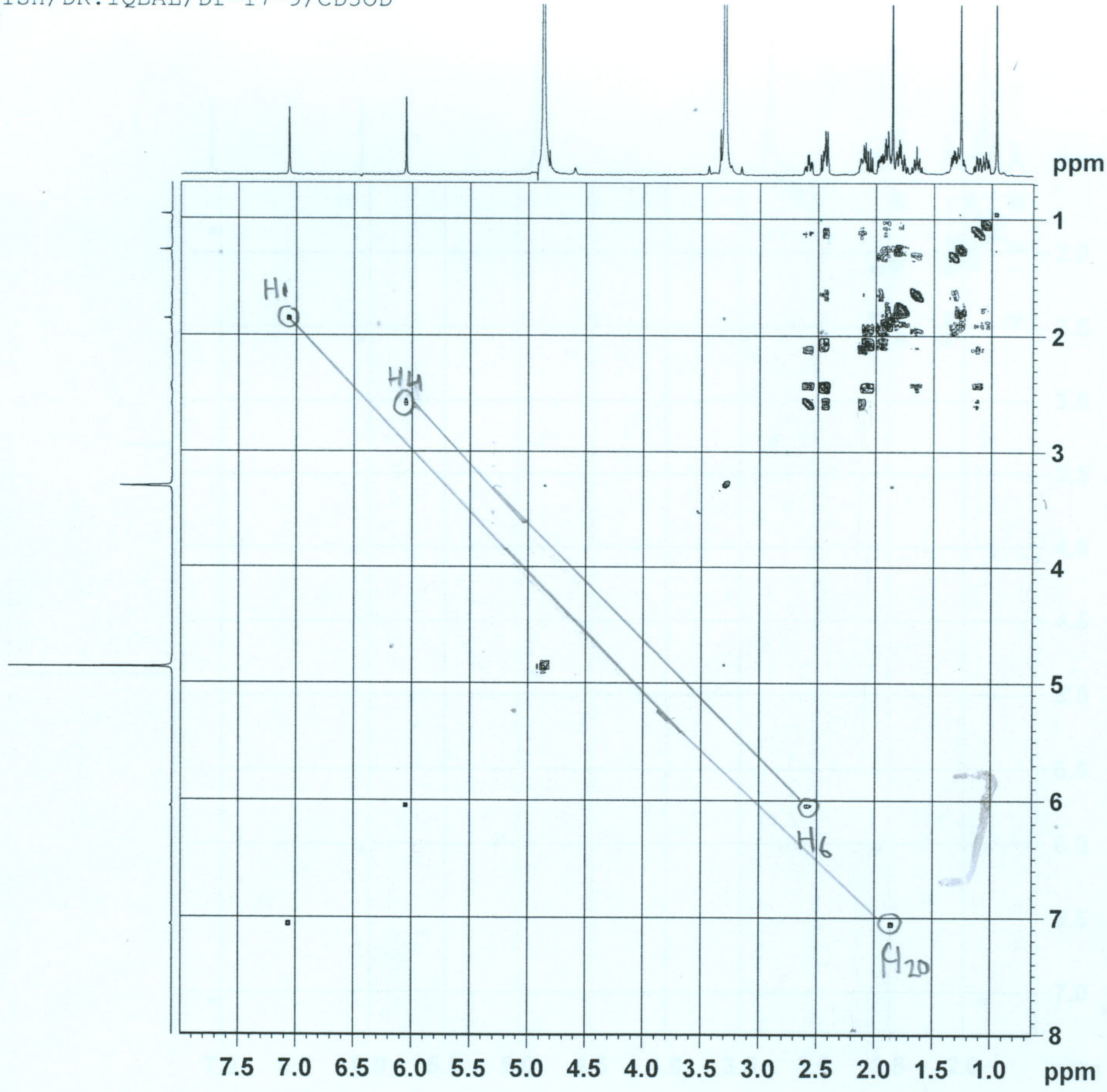


| | |
|---|---|
| NAME | Dec07-15 |
| EXPNO | 5 |
| PROCNO | 1 |
| Date_ | 20151207 |
| Time | 12.27 |
| INSTRUM | spect |
| PROBHD | 5 mm CPDUL 13C |
| PULPROG | cosydfqf |
| TD | 2048 |
| SOLVENT | MeOD |
| NS | 8 |
| DS | 2 |
| SWH | 4496.403 Hz |
| FIDRES | 2.195509 Hz |
| AQ | 0.2278988 sec |
| RG | 128 |
| DW | 111.200 usec |
| DE | 6.50 usec |
| TE | 298.0 K |
| D0 | 0.00000300 sec |
| D1 | 1.50000000 sec |
| D13 | 0.00000400 sec |
| D20 | 0.00000400 sec |
| IN0 | 0.00022240 sec |

======== CHANNEL f1 ========

| | |
|---|---|
| NUC1 | 1H |
| P1 | 16.50 usec |
| PL1 | -0.50 dB |
| PL1W | 14.09191513 W |
| SFO1 | 500.3322515 MHz |
| ND0 | 1 |
| TD | 256 |
| SFO1 | 500.3323 MHz |
| FIDRES | 17.564074 Hz |
| SW | 8.987 ppm |
| FnMODE | QF |
| SI | 2048 |
| SF | 500.3300143 MHz |
| WDW | QSINE |
| SSB | 0 |
| LB | 0.00 Hz |
| GB | 0 |
| PC | 4.00 |
| SI | 2048 |
| MC2 | QF |
| SF | 500.3300143 MHz |
| WDW | QSINE |
| SSB | 0 |
| LB | 0.00 Hz |
| GB | 0 |

comp. 5

MAHWISH/DR.IQBAL/Dr-17-9/CD3OD
NOESY

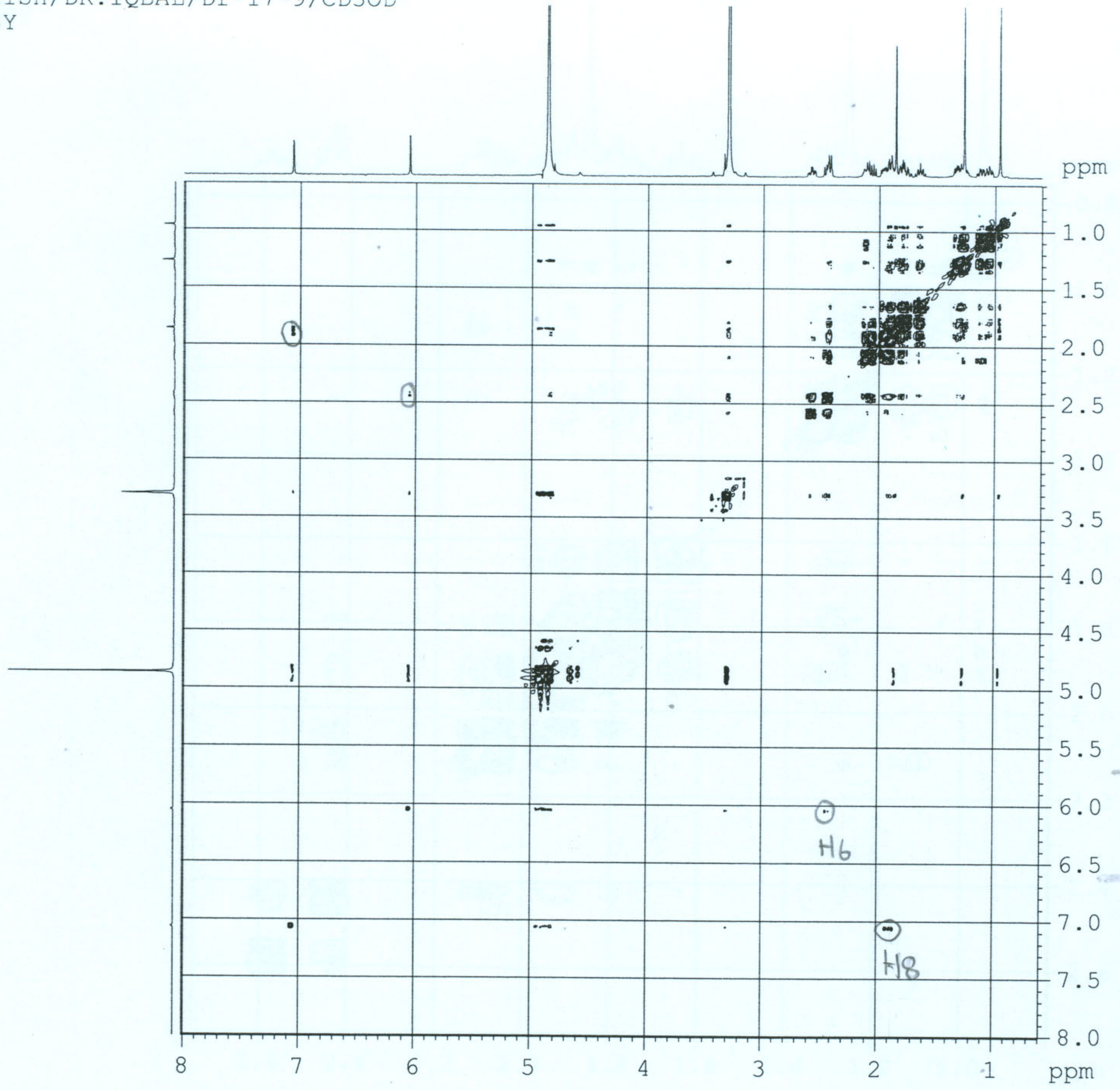


BRUKER

| | | |
|---|---|---|
| NAME | Dec07-15 | |
| EXPNO | 6 | |
| PROCNO | 1 | |
| Date_ | 20151207 | |
| Time | 13.28 | |
| INSTRUM | spect | |
| PROBHD | 5 mm CPDUL 13C | |
| PULPROG | noesygpph | |
| TD | 2048 | |
| SOLVENT | MeOD | |
| NS | 16 | |
| DS | 16 | |
| SWH | 4496.403 | Hz |
| FIDRES | 2.195509 | Hz |
| AQ | 0.2278988 | sec |
| RG | 57 | |
| DW | 111.200 | usec |
| DE | 6.50 | usec |
| TE | 298.0 | K |
| D0 | 0.00009019 | sec |
| D1 | 2.00000000 | sec |
| D8 | 0.80000001 | sec |
| D16 | 0.00020000 | sec |
| IN0 | 0.00022240 | sec |

======== CHANNEL f1 ========

| | | |
|---|---|---|
| NUC1 | 1H | |
| P1 | 16.50 | usec |
| P2 | 33.00 | usec |
| PL1 | -0.50 | dB |
| PL1W | 14.09191513 | W |
| SFO1 | 500.3322515 | MHz |

====== GRADIENT CHANNEL =====

| | | |
|---|---|---|
| GPNAM1 | SINE.100 | |
| GPZ1 | 40.00 | % |
| P16 | 1000.00 | usec |
| ND0 | 1 | |
| TD | 256 | |
| SFO1 | 500.3323 | MHz |
| FIDRES | 17.564074 | Hz |
| SW | 8.987 | ppm |
| FnMODE | States-TPPI | |
| SI | 1024 | |
| SF | 500.3300143 | MHz |
| WDW | QSINE | |
| SSB | 2 | |
| LB | 0.00 | Hz |
| GB | 0 | |
| PC | 4.00 | |
| SI | 1024 | |
| MC2 | States-TPPI | |
| SF | 500.3300143 | MHz |
| WDW | QSINE | |
| SSB | 2 | |
| LB | 0.00 | Hz |
| GB | 0 | |
