# Supplementary material for: Bio-Catalytic Structural Transformation of Anti-cancer Steroid, Drostanolone Enanthate with Cephalosporium aphidicola and Fusarium lini, and Cytotoxic Potential Evaluation of Its Metabolites against Certain Cancer Cell Lines
Source: Front Pharmacol. 2017 Dec 20;8:900. doi: 10.3389/fphar.2017.00900 (PMC5742531; doi:10.3389/fphar.2017.00900)

File: MK-12
Sample: MAHWISH /DR. IQBAL
Instrument: JEOL MS 600H-1

Date Run: 02-23-2017 (Time Run: 12:40:01)

Ionization mode: EI+

compound 6

Scan: 16
Base: m/z 136; 67.3%FS TIC: 4925788

R.T.: 1.33

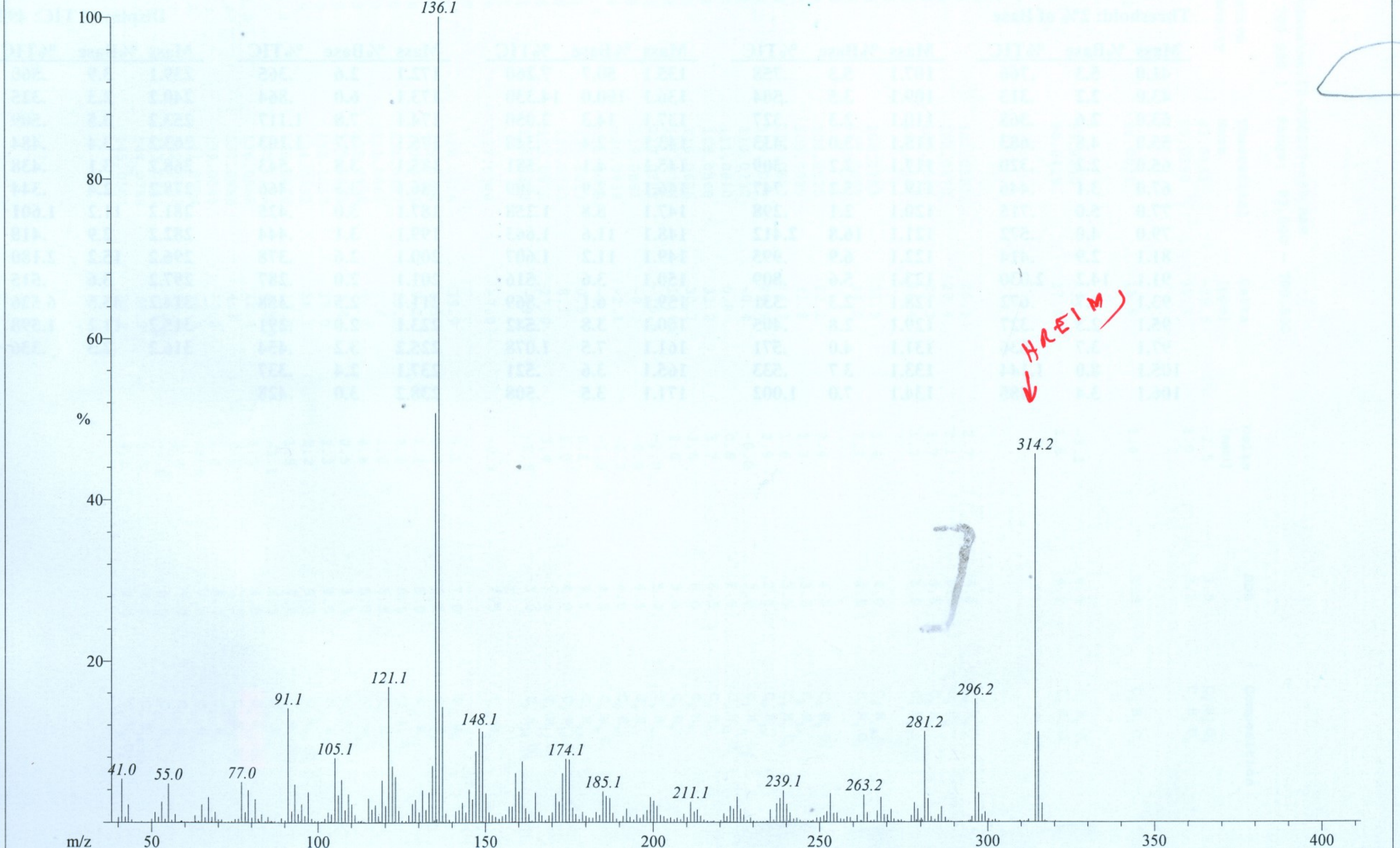

HREI Compound 6

Mass	Relative Intensity	Theoretical Mass	Delta [ppm]	Delta [mmu]	RDB	Composition
199.1110	2.1	199.1123	-6.4	-1.3	7.5	$C_{14} H_{15} O_{1}$
200.1172	2.4	200.1201	-14.6	-2.9	7.0	$C_{14} H_{16} O_{1}$
201.1283	1.1	201.1279	1.8	0.4	6.5	$C_{14} H_{17} O_{1}$
202.0746	1.5	202.0783	-18.3	-3.7	12.0	$C_{16} H_{10}$
203.0825	1.1	203.0861	-17.7	-3.6	11.5	$C_{16} H_{11}$
205.1084	1.1	205.1076	3.8	0.8	1.5	$C_{9} H_{17} O_{5}$
		205.1017	32.4	6.6	10.5	$C_{16} H_{13}$
206.1129	1.2	206.1154	-12.3	-2.5	1.0	$C_{9} H_{18} O_{5}$
		206.1096	16.1	3.3	10.0	$C_{16} H_{14}$
207.1139	1.5	207.1174	-16.9	-3.5	9.5	$C_{16} H_{15}$
209.0941	2.4	209.0966	-12.2	-2.5	9.5	$C_{15} H_{13} O_{1}$
210.0962	1.4	210.0892	33.5	7.0	5.0	$C_{11} H_{14} O_{4}$
		210.1045	-39.1	-8.2	9.0	$C_{15} H_{14} O_{1}$
211.1092	2.1	211.1123	-14.6	-3.1	8.5	$C_{15} H_{15} O_{1}$
213.1262	2.0	213.1279	-8.0	-1.7	7.5	$C_{15} H_{17} O_{1}$
221.0966	1.9	221.0966	-0.1	-0.0	10.5	$C_{16} H_{13} O_{1}$
221.1274	1.8	221.1330	-25.6	-5.7	9.5	$C_{17} H_{17}$
222.1038	1.6	222.1045	-2.8	-0.6	10.0	$C_{16} H_{14} O_{1}$
223.1119	4.2	223.1123	-2.0	-0.4	9.5	$C_{16} H_{15} O_{1}$
224.1205	2.7	224.1201	1.8	0.4	9.0	$C_{16} H_{16} O_{1}$
225.1319	3.1	225.1279	17.8	4.0	8.5	$C_{16} H_{17} O_{1}$
226.1317	1.6	226.1358	-17.8	-4.0	8.0	$C_{16} H_{18} O_{1}$
235.1162	2.2	235.1123	16.6	3.9	10.5	$C_{17} H_{15} O_{1}$
236.1162	1.1	236.1201	-16.7	-3.9	10.0	$C_{17} H_{16} O_{1}$
237.1384	2.4					
238.1357	3.6	238.1358	-0.2	-0.0	9.0	$C_{17} H_{18} O_{1}$
239.1430	3.9	239.1436	-2.3	-0.5	8.5	$C_{17} H_{19} O_{1}$
240.1515	1.9	240.1514	0.4	0.1	8.0	$C_{17} H_{20} O_{1}$
248.1207	1.1	248.1201	2.3	0.6	11.0	$C_{18} H_{16} O_{1}$
249.1359	1.1	249.1279	31.8	7.9	10.5	$C_{18} H_{17} O_{1}$
251.1366	1.2	251.1436	-27.9	-7.0	9.5	$C_{18} H_{19} O_{1}$
		251.1283	32.9	8.3	5.5	$C_{14} H_{19} O_{4}$
252.1513	1.1	252.1514	-0.4	-0.1	9.0	$C_{18} H_{20} O_{1}$
253.1606	2.7	253.1592	5.5	1.4	8.5	$C_{18} H_{21} O_{1}$
263.1370	4.7	263.1436	-25.0	-6.6	10.5	$C_{19} H_{19} O_{1}$
		263.1283	32.9	8.7	6.5	$C_{15} H_{19} O_{4}$
264.1373	1.0	264.1362	4.3	1.1	6.0	$C_{15} H_{20} O_{4}$
265.1665	1.0	265.1592	27.4	7.3	9.5	$C_{19} H_{21} O_{1}$
268.1510	1.8	268.1463	17.3	4.7	9.0	$C_{18} H_{20} O_{2}$
278.1568	3.0	278.1518	17.8	4.9	6.0	$C_{16} H_{22} O_{4}$
		278.1671	-37.1	-10.3	10.0	$C_{20} H_{22} O_{1}$
279.1591	1.7	279.1596	-2.0	-0.6	5.5	$C_{16} H_{23} O_{4}$
280.1632	1.4	280.1675	-15.4	-4.3	5.0	$C_{16} H_{24} O_{4}$
281.1489	6.6	281.1542	-18.6	-5.2	9.5	$C_{19} H_{21} O_{2}$
		281.1389	35.7	10.0	5.5	$C_{15} H_{21} O_{5}$
282.1511	1.7	282.1467	15.5	4.4	5.0	$C_{15} H_{22} O_{5}$
		282.1409	36.3	10.2	14.0	$C_{22} H_{18}$
		282.1620	-38.6	-10.9	9.0	$C_{19} H_{22} O_{2}$
296.1757	8.7	296.1776	-6.4	-1.9	9.0	$C_{20} H_{24} O_{2}$
297.1910	2.5	297.1855	18.6	5.5	8.5	$C_{20} H_{25} O_{2}$
298.1921	2.4	298.1933	-4.0	-1.2	8.0	$C_{20} H_{26} O_{2}$
314.1897	6.7	314.1882	4.7	1.5	8.0	$C_{20} H_{26} O_{3}$
315.2064	1.7	315.2113	-15.5	-4.9	11.5	$C_{24} H_{27}$
		315.1960	32.9	10.4	7.5	$C_{20} H_{27} O_{3}$
		315.2171	-34.1	-10.8	2.5	$C_{17} H_{31} O_{5}$

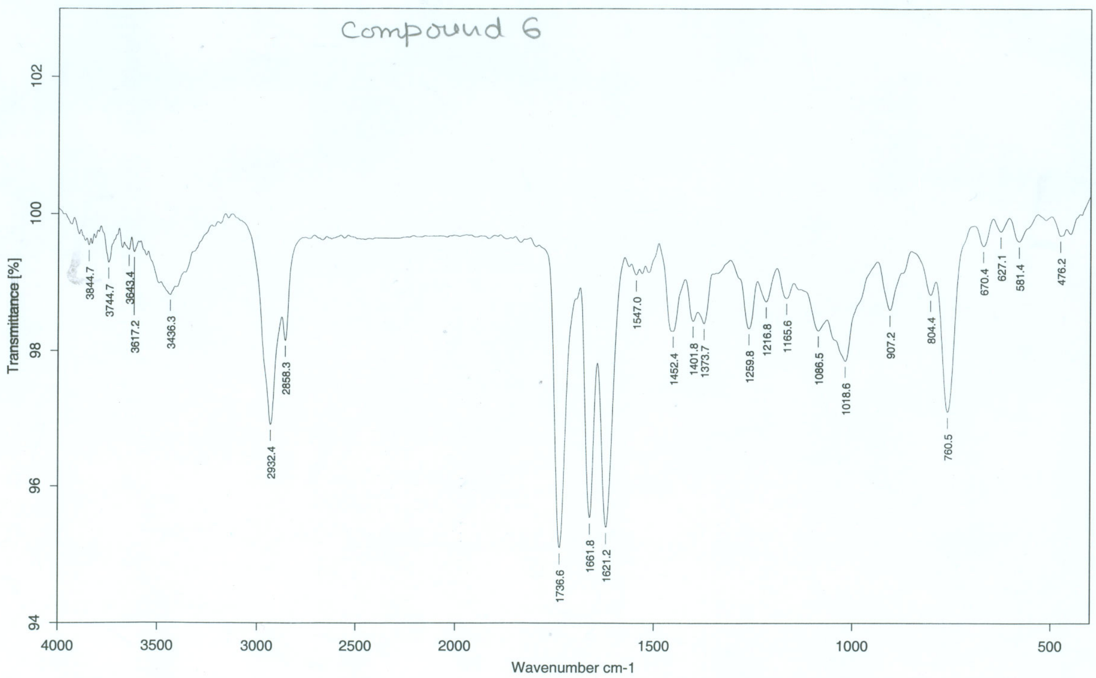

Sample : MK-12/Mahwish	Spectrum : MK-12.0 (in D:\IRSTUDENT)
Measured : 24/02/2017 on VECTOR22	Technic : Solid
Resolution : 4 cm-1 (10 scans)	Analyst : M. Asif

mahwish/dr.iqbal/mk.12/cd3od
1H

compound 6

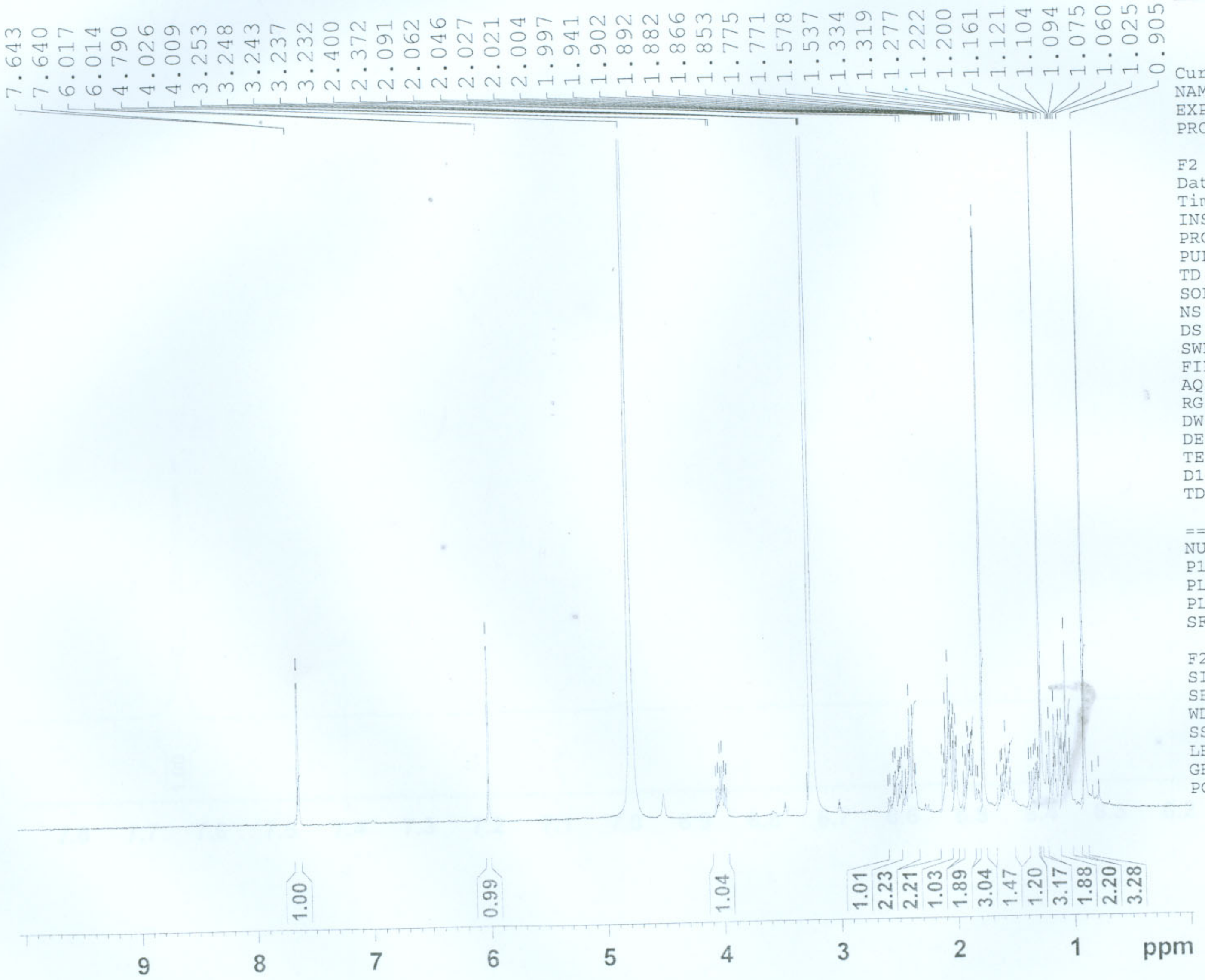

BRUKER

Current Data Parameters
NAME Dec06-16
EXPNO 15
PROCNO 1

F2 - Acquisition Parameters
Date_ 20161122
Time 14.58
INSTRUM Spect
PROBHD 5 mm BBO BB-1H
PULPROG zg30
TD 32768
SOLVENT MeOD
NS 64
DS 0
SWH 6188.119 Hz
FIDRES 0.188846 Hz
AQ 2.6476543 sec
RG 203
DW 80.800 usec
DE 6.50 usec
TE 300.0 K
D1 2.00000000 sec
TD0 1

======== CHANNEL f1 ========
NUC1 1H
P1 12.50 usec
PL1 0 dB
PL1W 13.16228485 W
SFO1 300.1324010 MHz

F2 - Processing parameters
SI 32768
SF 300.1300247 MHz
WDW EM
SSB 0
LB 0.30 Hz
GB 0
PC 1.00

Mahwish / DR.Iqbal / MK-12 / CD3OD
BB

Compound 6

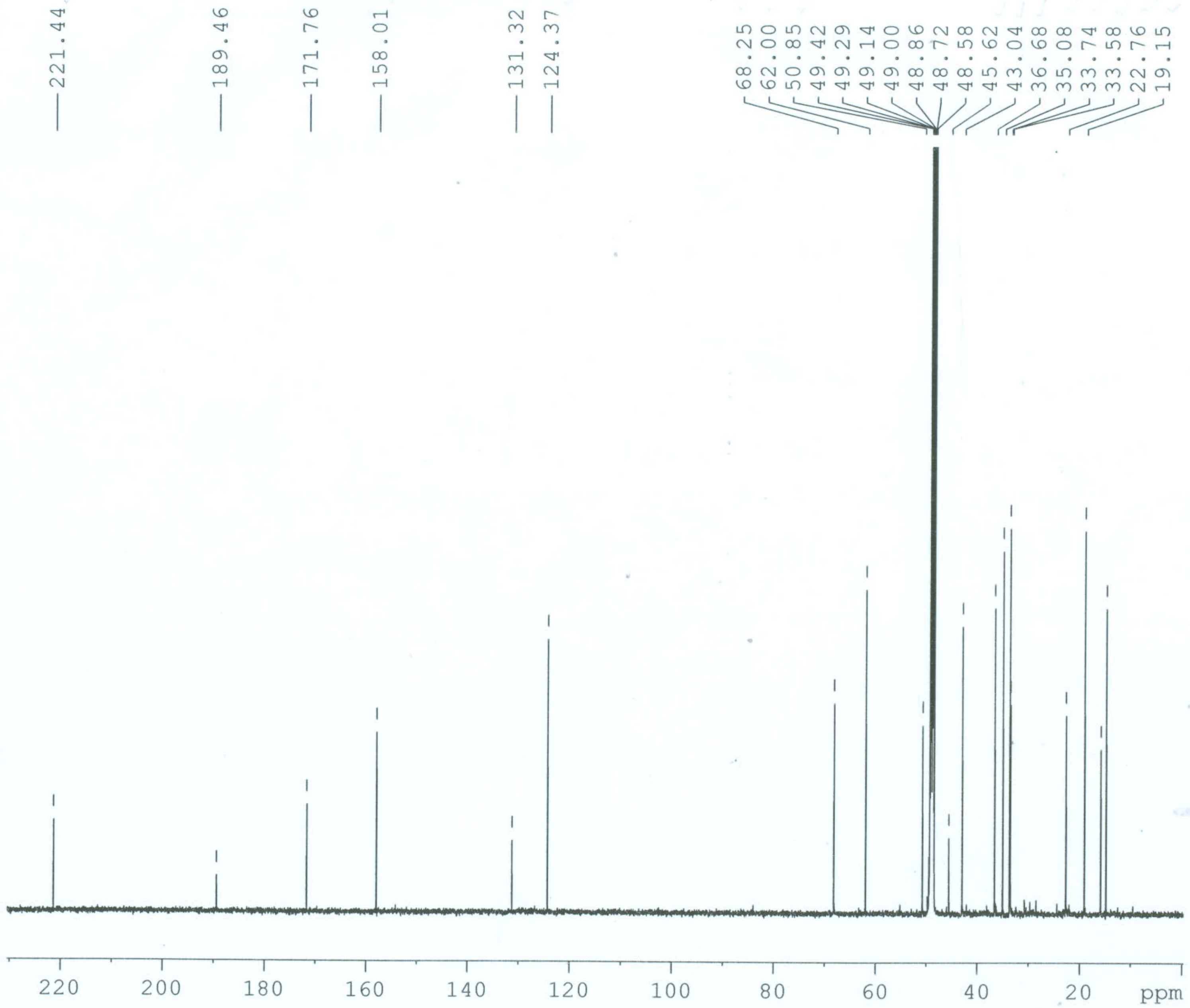

AVANCE AV-600
CRYO PROBE
LAB NO: 108

NAME	Dec06-16
EXPNO	10
PROCNO	1
Date_	20161207
Time	5.43
INSTRUM	spect
PROBHD	5 mm CPTCI 1H-
PULPROG	zgpg
TD	32768
SOLVENT	MeOD
NS	6871
DS	2
SWH	35971.223 Hz
FIDRES	1.097755 Hz
AQ	0.4555391 sec
RG	32768
DW	13.900 usec
DE	6.50 usec
TE	298.0 K
D1	1.50000000 sec
D11	0.03000000 sec
TD0	8

======== CHANNEL f1 ========

NUC1	13C
P1	15.40 usec
PL1	1.00 dB
PL1W	83.60149384 W
SFO1	150.9453107 MHz

======== CHANNEL f2 ========

CPDPRG2	waltz16
NUC2	1H
PCPD2	65.00 usec
PL2	3.30 dB
PL12	22.06 dB
PL13	27.00 dB
PL2W	9.16420078 W
PL12W	0.12192553 W
PL13W	0.03909260 W
SFO2	600.2336014 MHz
SI	16384
SF	150.9277403 MHz
WDW	EM
SSB	0
LB	1.00 Hz
GB	0
PC	1.00

Mahwish / DR.Iqbal / MK-12 / CD3OD
DEPT90

Compound 6

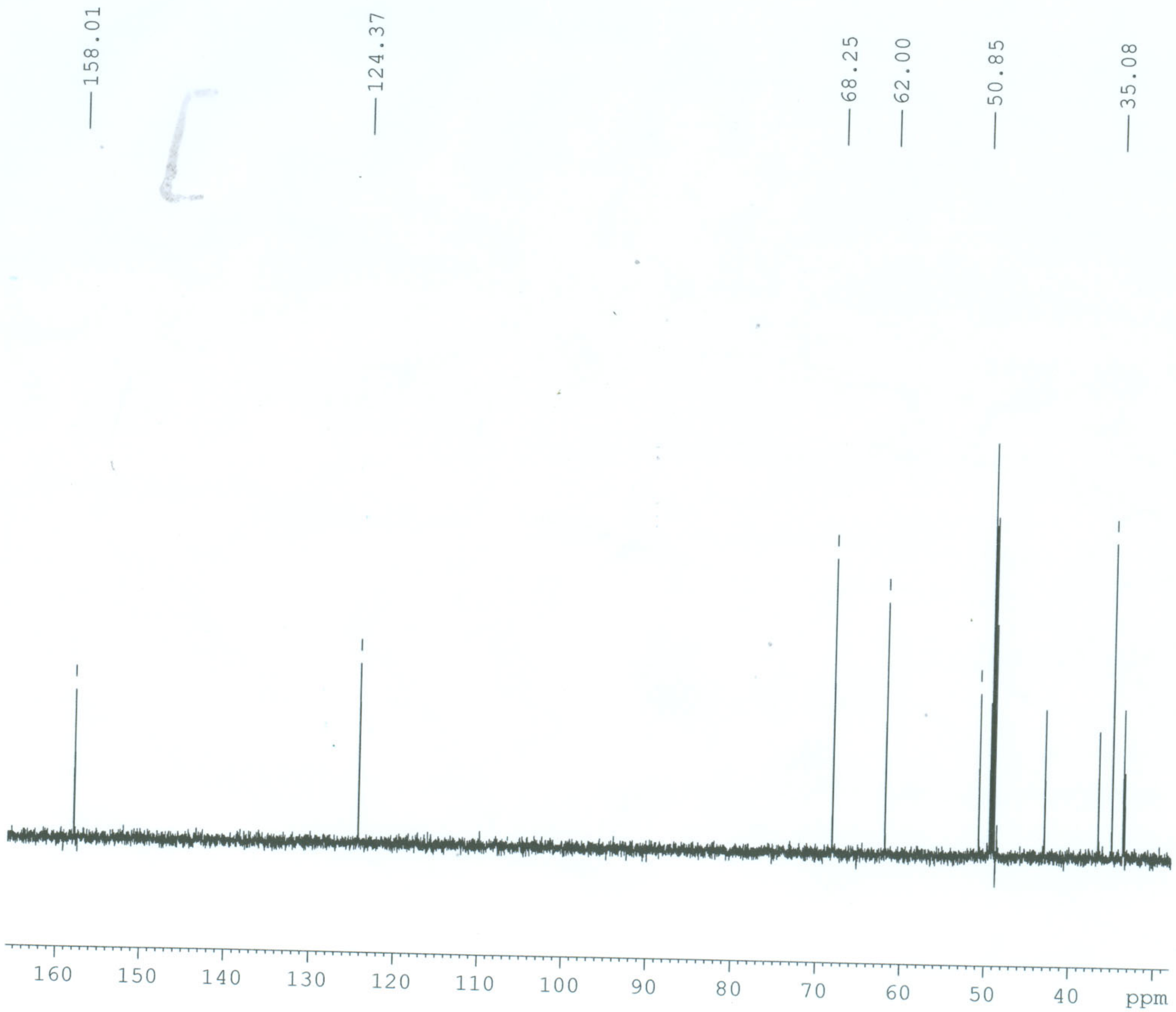

AVANCE AV-600
CRYO PROBE
LAB NO: 108

NAME	Dec06-16
EXPNO	12
PROCNO	1
Date_	20161207
Time	9.52
INSTRUM	spect
PROBHD	5 mm CPTCI 1H-
PULPROG	deptsp90
TD	32768
SOLVENT	MeOD
NS	605
DS	2
SWH	30303.031 Hz
FIDRES	0.924775 Hz
AQ	0.5407385 sec
RG	32768
DW	16.500 usec
DE	6.50 usec
TE	298.0 K
CNST2	145.0000000
D1	1.50000000 sec
D2	0.00344828 sec
D12	0.00002000 sec
TD0	2

======== CHANNEL f1 ========

NUC1	13C
P1	15.40 usec
P12	2000.00 usec
PL0	120.00 dB
PL1	1.00 dB
PL0W	0.00000000 W
PL1W	83.60149384 W
SFO1	150.9430468 MHz
SP2	5.40 dB
SPNAM2	Crp60comp.4
SPOAL2	0.500
SPOFFS2	0.00 Hz

======== CHANNEL f2 ========

CPDPRG2	waltz16
NUC2	1H
P3	7.50 usec
P4	15.00 usec
PCPD2	65.00 usec
PL2	3.30 dB
PL12	22.06 dB
PL2W	9.16420078 W
PL12W	0.12192553 W
SFO2	600.2324009 MHz
SI	16384
SF	150.9277403 MHz
WDW	EM
SSB	0
LB	1.00 Hz
GB	0
PC	1.00

Mahwish / DR.Iqbal / MK-12 / CD3OD
DEPT135

Compound 6

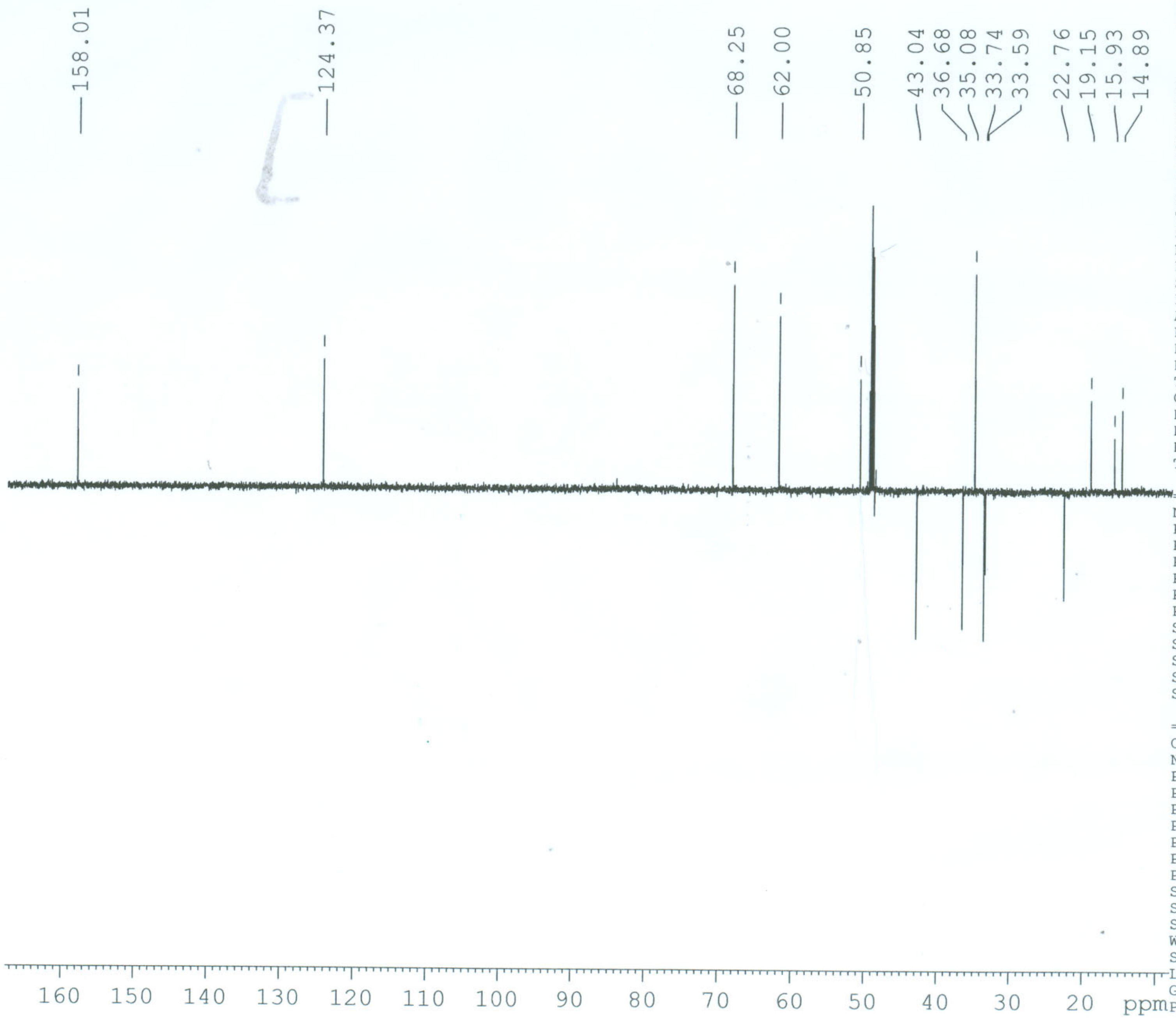

BRUKER

NAME	Dec06-16	
EXPNO	11	
PROCNO	1	
Date_	20161207	
Time	9.36	
INSTRUM	spect	
PROBHD	5 mm CPTCI 1H-	
PULPROG	deptsp135	
TD	32768	
SOLVENT	MeOD	
NS	1133	
DS	2	
SWH	30303.031	Hz
FIDRES	0.924775	Hz
AQ	0.5407385	sec
RG	32768	
DW	16.500	usec
DE	6.50	usec
TE	298.0	K
CNST2	145.0000000	
D1	1.50000000	sec
D2	0.00344828	sec
D12	0.00002000	sec
TD0	4	

======== CHANNEL f1 ========

NUC1	13C	
P1	15.40	usec
P12	2000.00	usec
PL0	120.00	dB
PL1	1.00	dB
PL0W	0.00000000	W
PL1W	83.60149384	W
SFO1	150.9430468	MHz
SP2	5.40	dB
SPNAM2	Crp60comp.4	
SPOAL2	0.500	
SPOFFS2	0.00	Hz

======== CHANNEL f2 ========

CPDPRG2	waltz16	
NUC2	1H	
P3	7.50	usec
P4	15.00	usec
PCPD2	65.00	usec
PL2	3.30	dB
PL12	22.06	dB
PL2W	9.16420078	W
PL12W	0.12192553	W
SFO2	600.2324009	MHz
SI	16384	
SF	150.9277403	MHz
WDW	EM	
SSB	0	
LB	1.00	Hz
GB	0	
PC	1.00	

compound 6

Mahwish / DR.Iqbal / MK-12 / CD3OD
HSQC

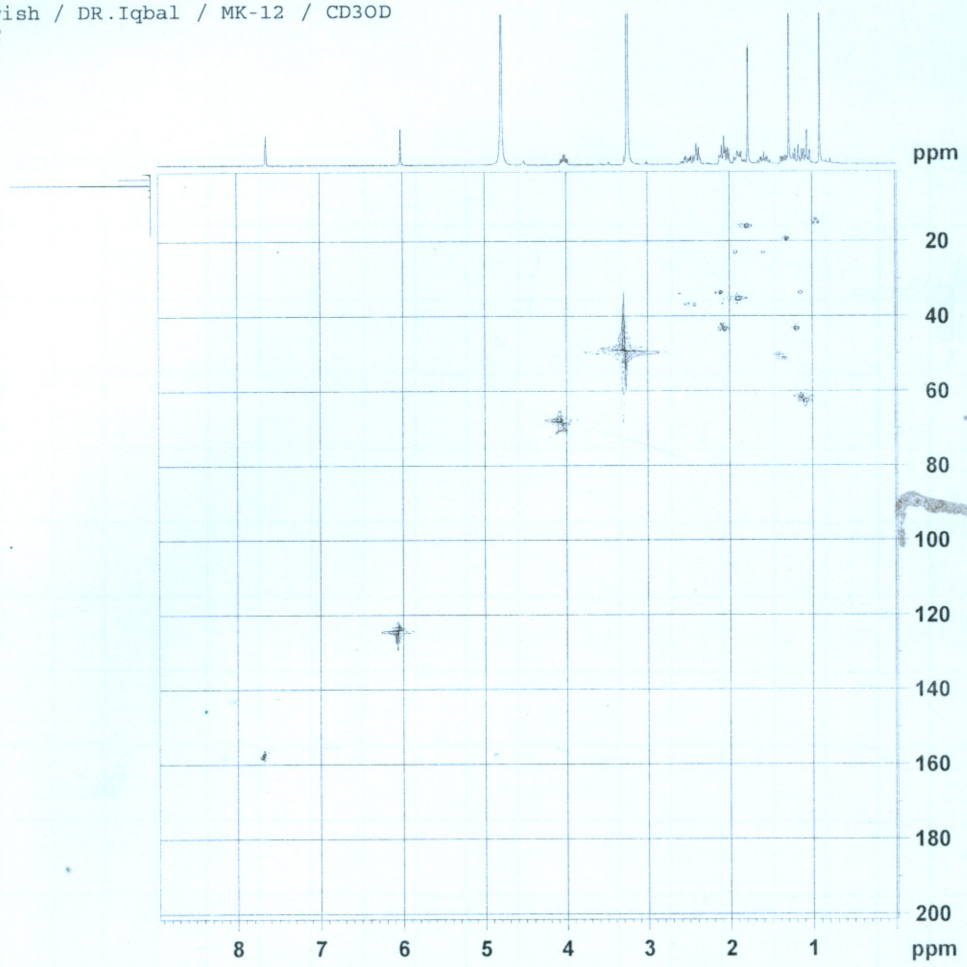

BRUKER

```
Current Data Parameters
NAME               Dec06-16
EXPNO                     8
PROCNO                    1

F2 - Acquisition Parameters
Date_              20161207
Time                   0.21
INSTRUM               spect
PROBHD   5 mm CPTCI 1H-
PULPROG          hsqcetgpsi
TD                     1024
SOLVENT                MeOD
NS                       32
DS                        8
SWH                5387.931 Hz
FIDRES             5.261652 Hz
AQ                0.0950272 sec
RG                  36780.8
DW                   92.800 usec
DE                     6.50 usec
TE                    298.0 K
CNST2           145.0000000
D0               0.00000300 sec
D1               1.50000000 sec
D4               0.00172414 sec
D11              0.03000000 sec
D13              0.00000400 sec
D16              0.00015000 sec
D24              0.00110000 sec
IN0              0.00001655 sec
ZGOPTNS

======== CHANNEL f1 ========
NUC1                     1H
P1                     7.20 usec
P2                    14.40 usec
P28                    0.50 usec
PL1                    3.30 dB
PL1W             9.16420078 W
SFO1            600.2327010 MHz

======== CHANNEL f2 ========
CPDPRG[2               garp
NUC2                    13C
P3                    15.40 usec
P4                    30.80 usec
PCPD2                 61.00 usec
PL2                    1.00 dB
PL12                  13.00 dB
PL2W            83.60149384 W
PL12W            5.27489758 W
SFO2            150.9430468 MHz

====== GRADIENT CHANNEL =====
GPNAM[1]           SINE.100
GPNAM[2]           SINE.100
GPZ1                  80.00 %
GPZ2                  20.10 %
P16                 2000.00 usec

F1 - Acquisition parameters
TD                      256
SFO1                150.943 MHz
FIDRES           235.848511 Hz
SW                  200.000 ppm
FnMODE       Echo-Antiecho

F2 - Processing parameters
SI                     1024
SF              600.2300247 MHz
WDW                   QSINE
SSB                       2
LB              0 Hz
GB              0
PC                     4.00

F1 - Processing parameters
SI                     1024
MC2          echo-antiecho
SF              150.9277403 MHz
WDW                   QSINE
SSB                       2
LB              0 Hz
GB              0
```

HMBC
Mahwish*/ Dr. Iqbal / MK-12 / CD3OD / compound 6

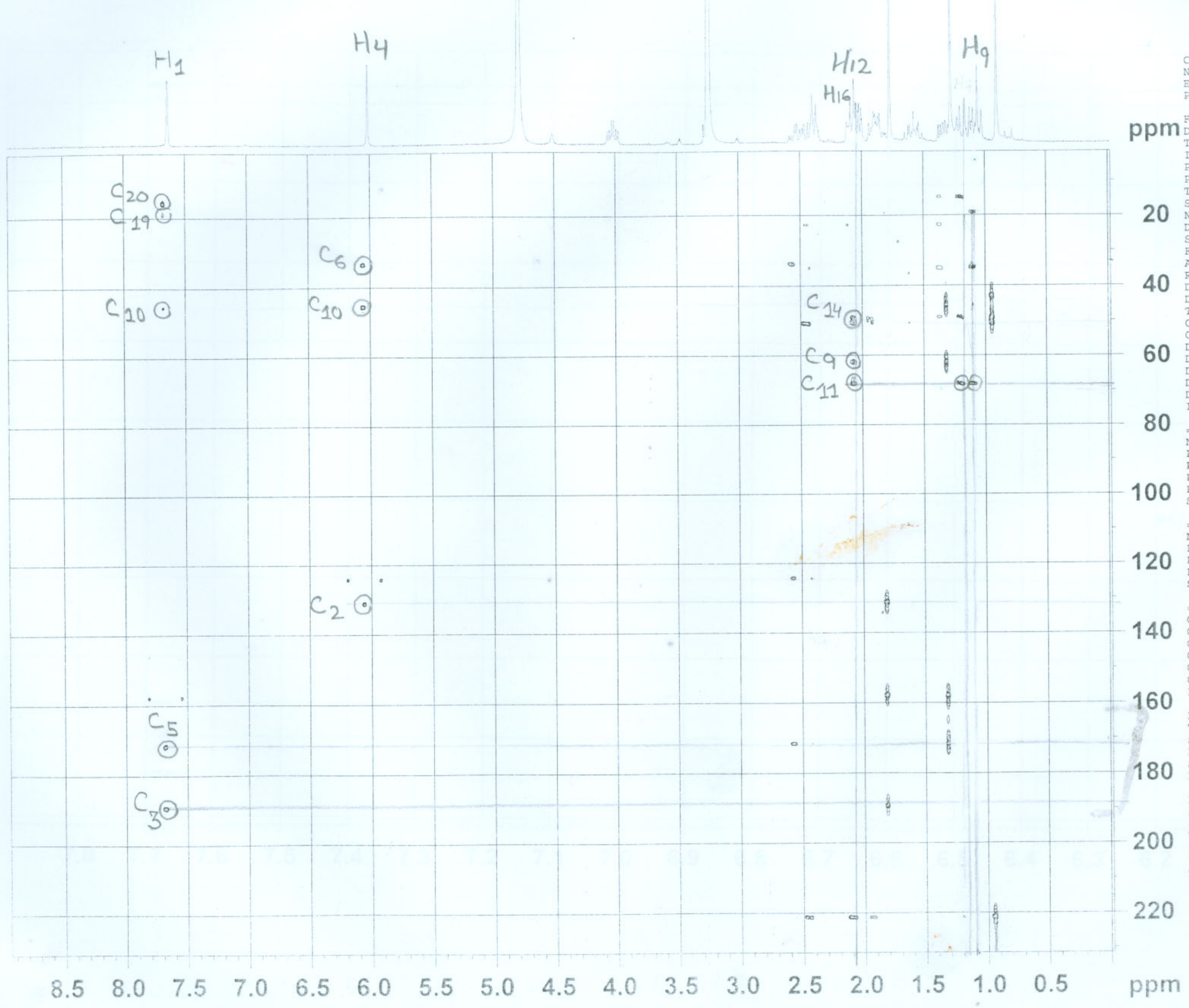

BRUKER

Current Data Parameters		
NAME	Dec06-16	
EXPNO	9	
PROCNO	1	
F2 - Acquisition Parameters		
Date_	20161207	
Time	4.03	
INSTRUM	spect	
PROBHD	5 mm CPTCI 1H-	
PULPROG	hmbcgplpndqf	
TD	2048	
SOLVENT	MeOD	
NS	32	
DS	16	
SWH	5387.931	Hz
FIDRES	2.630826	Hz
AQ	0.1900544	sec
RG	46341	
DW	92.800	usec
DE	6.50	usec
TE	298.0	K
CNST2	145.0000000	
CNST13	13.0000000	
D0	0.00000300	sec
D1	2.00000000	sec
D2	0.00344828	sec
D6	0.03846154	sec
D16	0.00015000	sec
IN0	0.00001440	sec
======== CHANNEL f1 ========		
NUC1	1H	
P1	7.20	usec
P2	14.40	usec
PL1	3.30	dB
PL1W	9.16420078	W
SFO1	600.2327010	MHz
======== CHANNEL f2 ========		
NUC2	13C	
P3	15.40	usec
PL2	1.00	dB
PL2W	83.60149384	W
SFO2	150.9453107	MHz
====== GRADIENT CHANNEL =====		
GPNAM[1]	SINE.100	
GPNAM[2]	SINE.100	
GPNAM[3]	SINE.100	
GPZ1	50.00	%
GPZ2	30.00	%
GPZ3	40.10	%
P16	2000.00	usec
F1 - Acquisition parameters		
TD	256	
SFO1	150.9453	MHz
FIDRES	271.229858	Hz
SW	230.000	ppm
FnMODE	QF	
F2 - Processing parameters		
SI	1024	
SF	600.2300247	MHz
WDW	SINE	
SSB	0	
LB	0	Hz
GB	0	
PC	1.40	
F1 - Processing parameters		
SI	1024	
MC2	QF	
SF	150.9277403	MHz
WDW	SINE	
SSB	0	
LB	0	Hz
GB	0	

compound 6

Mahwish / DR.Iqbal / MK-12 / CD3OD
COSY

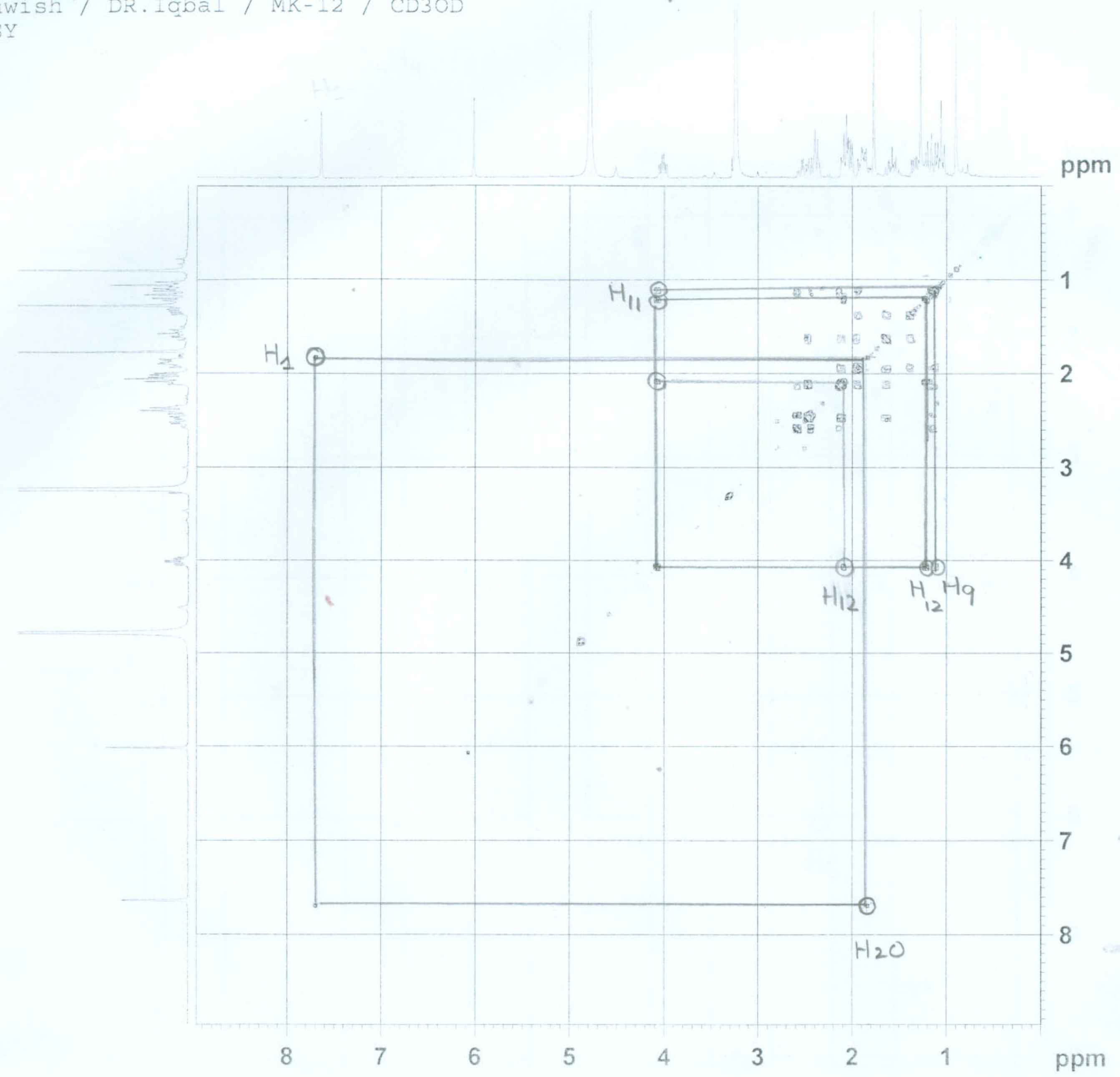

BRUKER

Current Data Parameters

NAME	Dec06-16
EXPNO	6
PROCNO	1

F2 - Acquisition Parameters

Date_	20161206
Time	20.01
INSTRUM	spect
PROBHD	5 mm CPTCI 1H-
PULPROG	cosydfqf
TD	2048
SOLVENT	MeOD
NS	8
DS	4
SWH	5387.931 Hz
FIDRES	2.630826 Hz
AQ	0.1900544 sec
RG	90.5
DW	92.800 usec
DE	6.50 usec
TE	298.0 K
D0	0.00000300 sec
D1	1.50000000 sec
D13	0.00000400 sec
D20	0.00000200 sec
IN0	0.00018560 sec

======== CHANNEL f1 ========

NUC1	1H
P1	7.20 usec
PL1	3.30 dB
PL1W	9.16420078 W
SFO1	600.2327010 MHz

F1 - Acquisition parameters

TD	256
SFO1	600.2327 MHz
FIDRES	42.093212 Hz
SW	8.976 ppm
FnMODE	QF

F2 - Processing parameters

SI	1024
SF	600.2300154 MHz
WDW	QSINE
SSB	0
LB	0 Hz
GB	0
PC	1.40

F1 - Processing parameters

SI	1024
MC2	QF
SF	600.2300154 MHz
WDW	QSINE
SSB	0
LB	0 Hz
GB	0

NOESY Compound 6

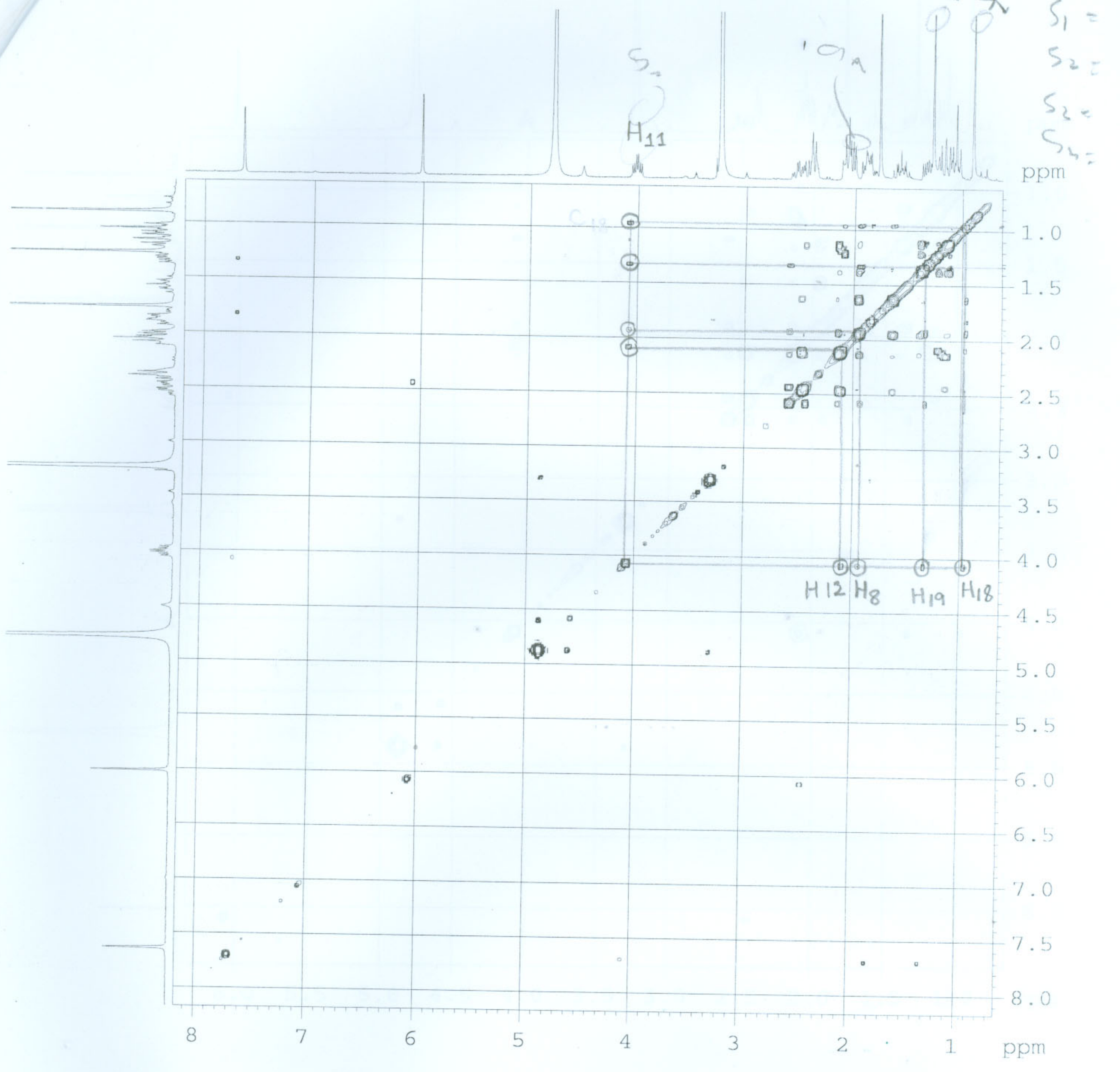

Supplement: Supplementary file 6 [file DataSheet6.PDF]
