# Supplementary material for: Bio-Catalytic Structural Transformation of Anti-cancer Steroid, Drostanolone Enanthate with Cephalosporium aphidicola and Fusarium lini, and Cytotoxic Potential Evaluation of Its Metabolites against Certain Cancer Cell Lines
Source: Front Pharmacol. 2017 Dec 20;8:900. doi: 10.3389/fphar.2017.00900 (PMC5742531; doi:10.3389/fphar.2017.00900)

2/23/2017 1:01:20 PM

File: MK-14R
Sample: MAHWISH /DR. IQBAL
Instrument: JEOL MS 600H-1

Date Run: 02-23-2017 (Time Run: 12:48:18)

Ionization mode: EI+ Compound 7

Scan: 30
Base: m/z 135; 4.1%FS TIC: 748862

R.T.: 2.57

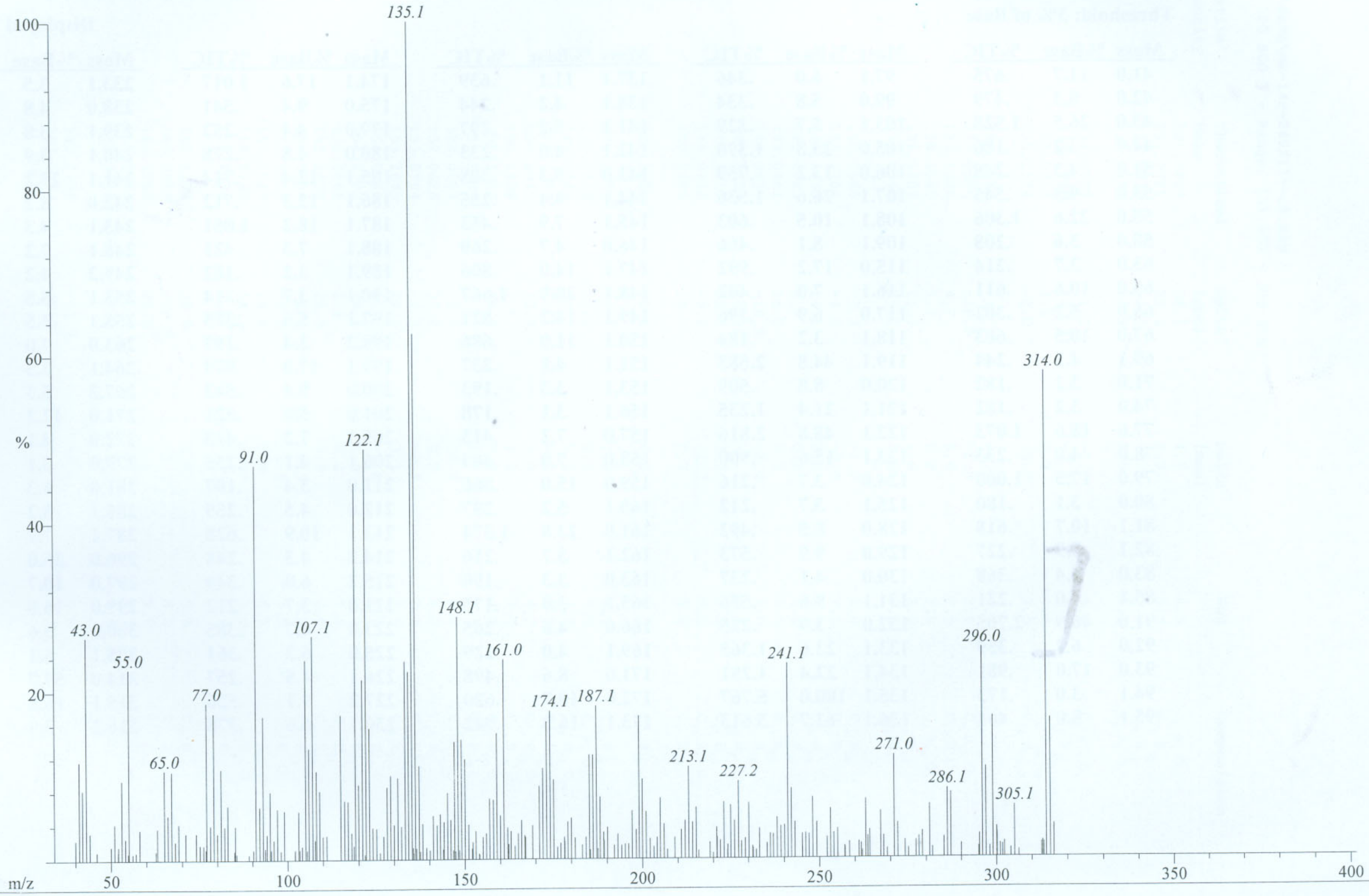

HREI Compound 7

Mass	Relative Intensity	Theoretical Mass	Delta [ppm]	Delta [mmu]	RDB	Composition
		202.0841	-27.1	-5.5	3.0	$C_9 H_{14} O_5$
203.1702	1.0	203.1647	26.9	5.5	0.5	$C_{11} H_{23} O_3$
205.1000	1.9	205.1017	-8.6	-1.8	10.5	$C_{16} H_{13}$
		205.1076	-37.2	-7.6	1.5	$C_9 H_{17} O_5$
206.1167	1.4	206.1154	6.4	1.3	1.0	$C_9 H_{18} O_5$
		206.1096	34.9	7.2	10.0	$C_{16} H_{14}$
207.1182	1.4	207.1174	4.0	0.8	9.5	$C_{16} H_{15}$
		207.1232	-24.4	-5.1	0.5	$C_9 H_{19} O_5$
209.1025	1.3	209.0966	28.1	5.9	9.5	$C_{15} H_{13} O_1$
209.1276	1.2	209.1330	-25.9	-5.4	8.5	$C_{16} H_{17}$
211.1117	1.7	211.1123	-2.8	-0.6	8.5	$C_{15} H_{15} O_1$
211.1457	1.0	211.1487	-14.2	-3.0	7.5	$C_{16} H_{19}$
213.1260	1.7	213.1279	-9.2	-2.0	7.5	$C_{15} H_{17} O_1$
213.1860	2.0	213.1855	2.7	0.6	1.5	$C_{13} H_{25} O_2$
214.1401	1.0	214.1358	20.4	4.4	7.0	$C_{15} H_{18} O_1$
219.1258	1.2	219.1232	11.5	2.5	1.5	$C_{10} H_{19} O_5$
		219.1174	38.4	8.4	10.5	$C_{17} H_{15}$
221.1263	1.1	221.1330	-30.5	-6.8	9.5	$C_{17} H_{17}$
		221.1178	38.5	8.5	5.5	$C_{13} H_{17} O_3$
223.1227	1.6					
223.1305	1.6	223.1334	-13.3	-3.0	4.5	$C_{13} H_{19} O_3$
224.1154	1.2	224.1201	-21.0	-4.7	9.0	$C_{16} H_{16} O_1$
225.1242	2.0	225.1279	-16.8	-3.8	8.5	$C_{16} H_{17} O_1$
227.1511	2.1	227.1436	33.1	7.5	7.5	$C_{16} H_{19} O_1$
235.1501	1.1	235.1487	6.1	1.4	9.5	$C_{18} H_{19}$
		235.1545	-18.9	-4.4	0.5	$C_{11} H_{23} O_5$
237.1221	1.3	237.1279	-24.7	-5.9	9.5	$C_{17} H_{17} O_1$
		237.1127	39.7	9.4	5.5	$C_{13} H_{17} O_4$
239.1402	1.7	239.1436	-14.4	-3.4	8.5	$C_{17} H_{19} O_1$
241.1571	3.6	241.1592	-8.9	-2.2	7.5	$C_{17} H_{21} O_1$
241.2029	1.1	241.1956	30.1	7.3	6.5	$C_{18} H_{25}$
242.1693	1.4	242.1671	9.2	2.2	7.0	$C_{17} H_{22} O_1$
254.1667	1.1	254.1671	-1.5	-0.4	8.0	$C_{18} H_{22} O_1$
263.1552	1.1	263.1647	-36.3	-9.6	5.5	$C_{16} H_{23} O_3$
278.1709	1.1	278.1671	13.9	3.9	10.0	$C_{20} H_{22} O_1$
281.1542	1.0	281.1542	0.2	0.1	9.5	$C_{19} H_{21} O_2$
296.1789	3.7	296.1776	4.3	1.3	9.0	$C_{20} H_{24} O_2$
298.1859	1.0	298.1933	-24.8	-7.4	8.0	$C_{20} H_{26} O_2$
		298.1780	26.4	7.9	4.0	$C_{16} H_{26} O_5$
299.1721	1.1	299.1647	24.8	7.4	8.5	$C_{19} H_{23} O_3$
		299.1800	-26.2	-7.9	12.5	$C_{23} H_{23}$
300.1727	2.0	300.1725	0.5	0.2	8.0	$C_{19} H_{24} O_3$
314.1861	3.9	314.1882	-6.6	-2.1	8.0	$C_{20} H_{26} O_3$

I. C.C.B.S., University of Karachi Analytical Laboratory - Pakistan

Compound 7

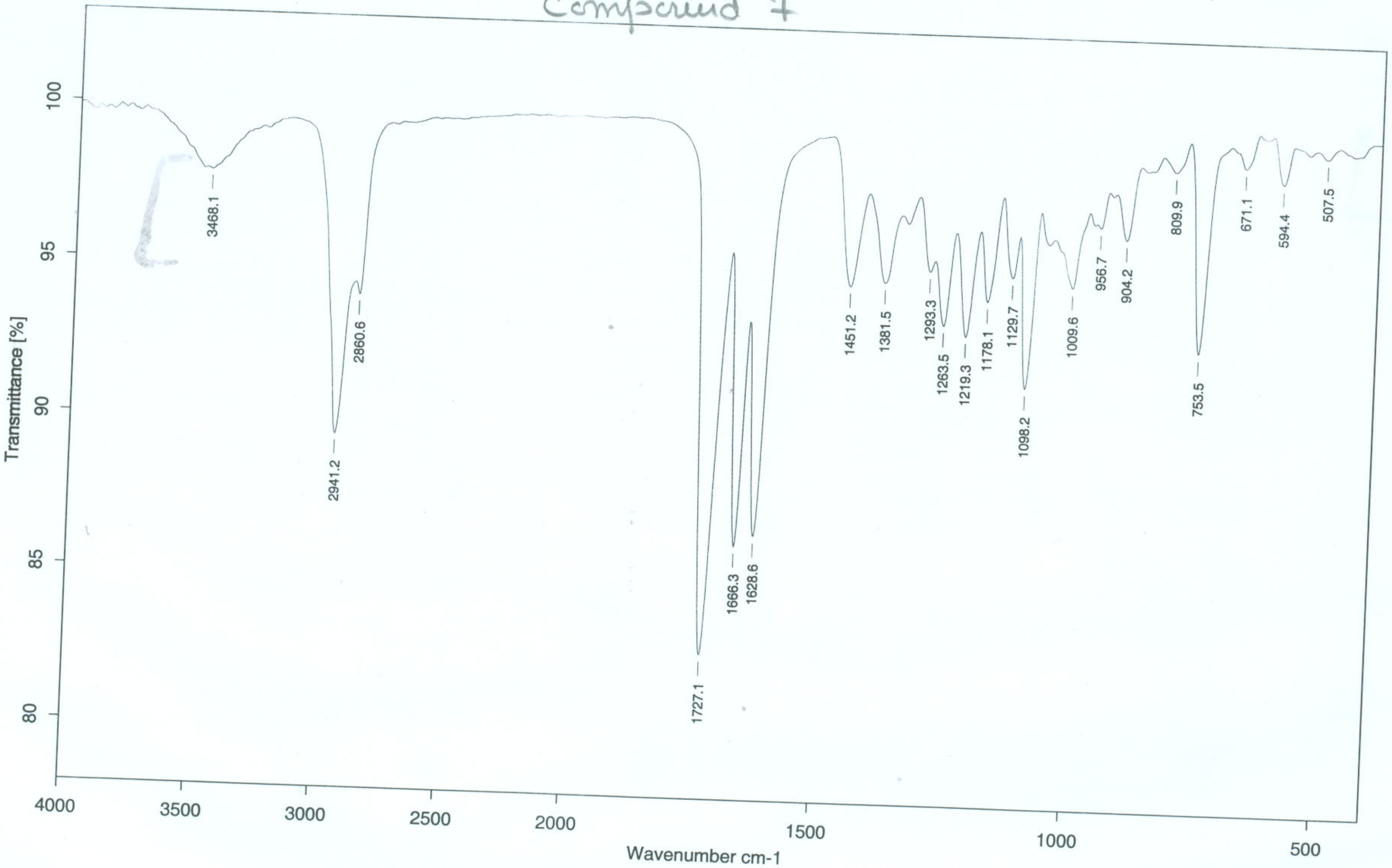

Sample : MK-14/Mahwish	
Measured : 24/02/2017 on VECTOR22	Spectrum : MK-14.0 (in D:\IRSTUDENT)
Resolution : 4 cm-1 (10 scans)	Technic : Solid
	Analyst : M. Asif

Mahwish / DR.Iqbal / MK-14R / CD3OD * compound 7

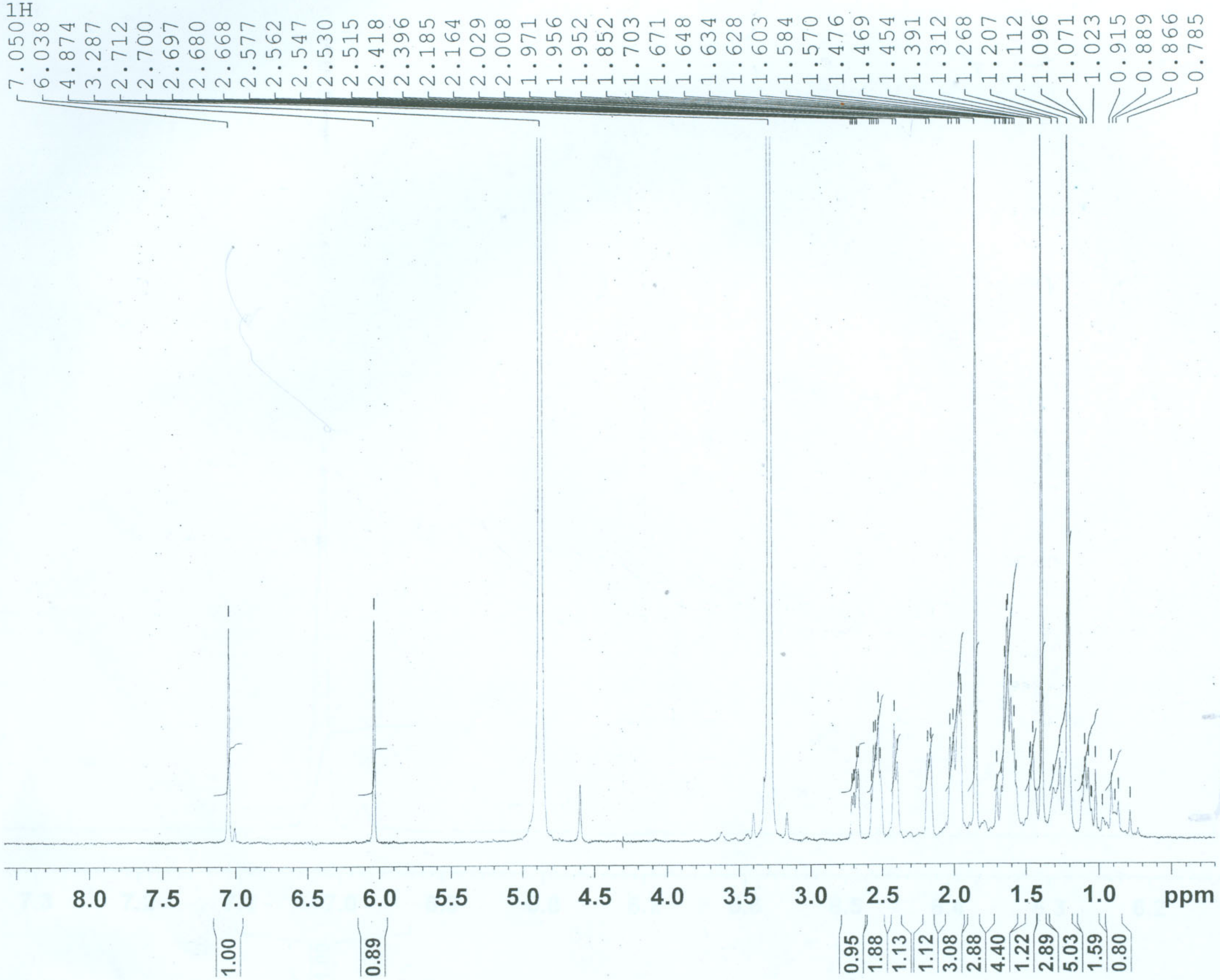

BRUKER

Current Data Parameters
NAME Dec07-16
EXPNO 1
PROCNO 1

F2 - Acquisition Parameters
Date_ 20161207
Time 20.05
INSTRUM spect
PROBHD 5 mm CPTCI 1H-
PULPROG zg30
TD 32768
SOLVENT MeOD
NS 32
DS 0
SWH 12019.230 Hz
FIDRES 0.366798 Hz
AQ 1.3631488 sec
RG 8
DW 41.600 usec
DE 6.50 usec
TE 298.0 K
D1 1.50000000 sec
TD0 1

======== CHANNEL f1 ========
NUC1 1H
P1 7.20 usec
PL1 3.30 dB
PL1W 9.16420078 W
SFO1 600.2348018 MHz

F2 - Processing parameters
SI 16384
SF 600.2300237 MHz
WDW EM
SSB 0
LB 0.30 Hz
GB 0
PC 1.00

compound 7

MEHWISH/DR.IQBAL/MK.14/CD3OD
BB

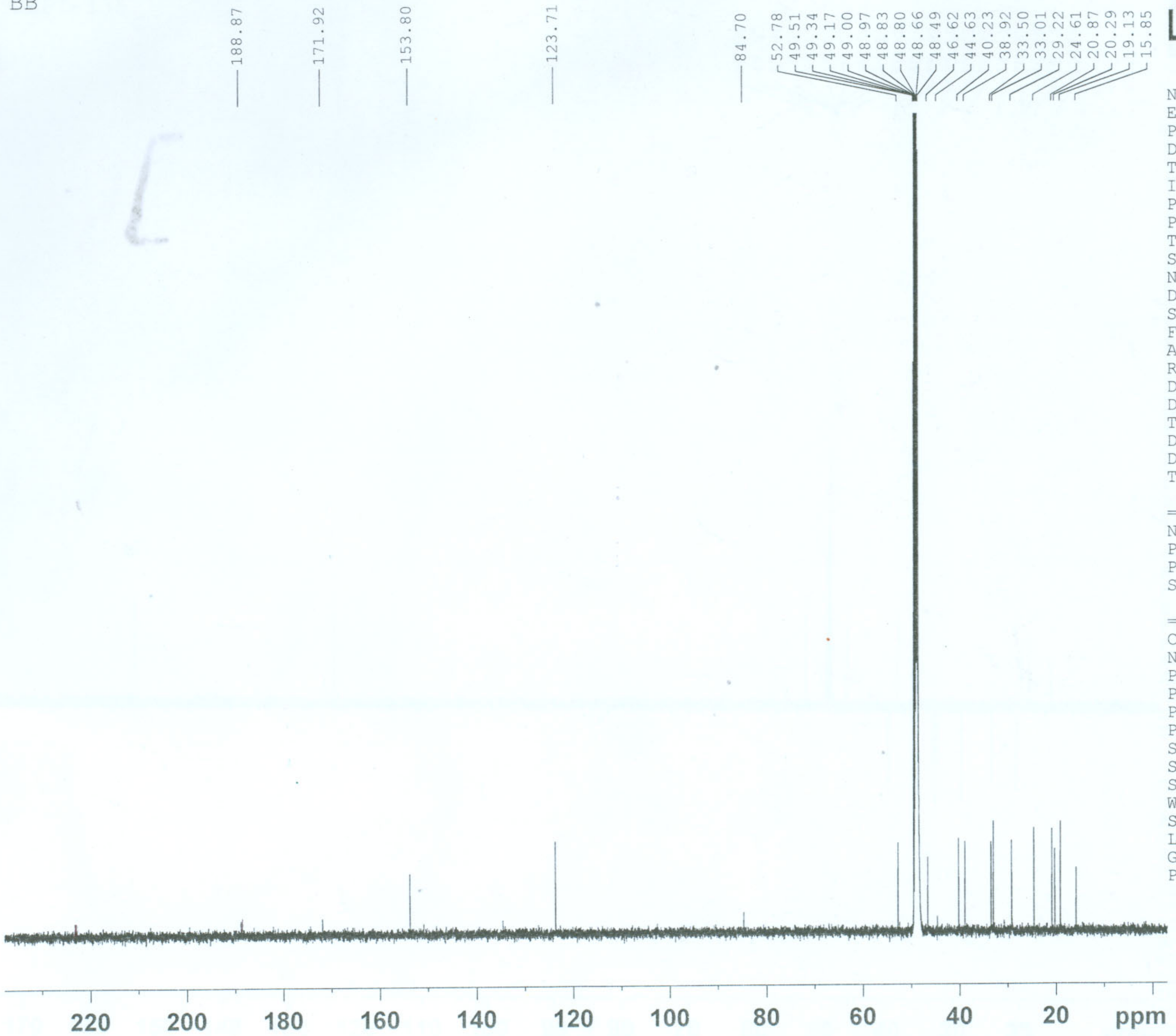

AVANCE AV-500
LAB NO:118

NAME	dec15-16	
EXPNO	6	
PROCNO	1	
Date_	20161216	
Time	12.41	
INSTRUM	spect	
PROBHD	5 mm PABBI 1H/	
PULPROG	zgpg	
TD	32768	
SOLVENT	MeOD	
NS	20480	
DS	4	
SWH	30303.031	Hz
FIDRES	0.924775	Hz
AQ	0.5407385	sec
RG	32768	
DW	16.500	usec
DE	6.50	usec
TE	296.2	K
D1	1.50000000	sec
D11	0.03000000	sec
TD0	20	

======== CHANNEL f1 ========

NUC1	13C	
P1	13.35	usec
PL1	-3.00	dB
SFO1	125.7975248	MHz

======== CHANNEL f2 ========

CPDPRG2	waltz16	
NUC2	1H	
PCPD2	80.00	usec
PL2	3.00	dB
PL12	22.74	dB
PL13	26.00	dB
SFO2	500.2330014	MHz
SI	32768	
SF	125.7827558	MHz
WDW	EM	
SSB	0	
LB	1.00	Hz
GB	0	
PC	1.40	

Mahwish / DR.Iqbal / MK-14R / CD3OD
DEPT135

compound 7

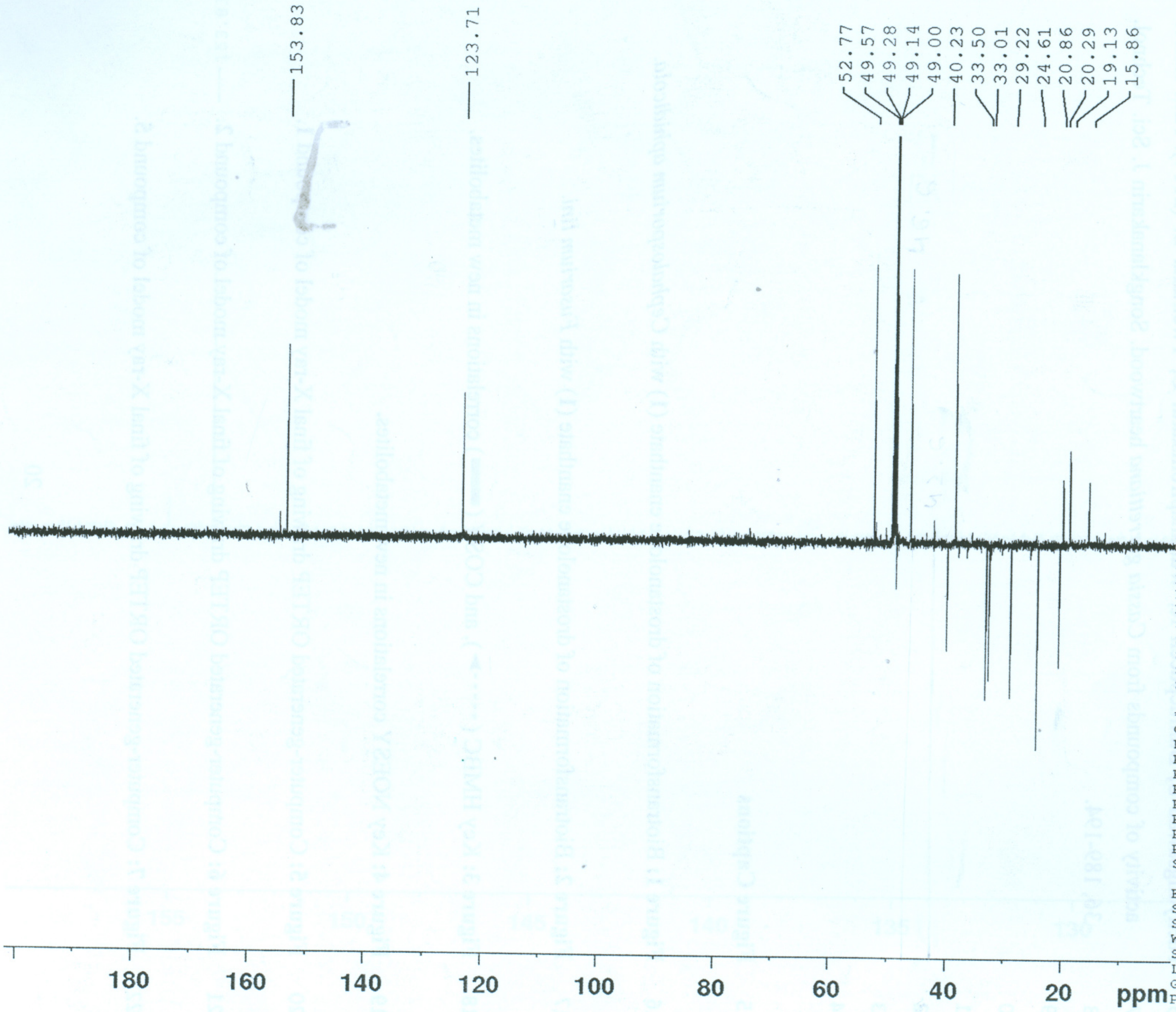

Current Data Parameters
NAME Dec07-16
EXPNO 7
PROCNO 1

F2 - Acquisition Parameters
Date_ 20161208
Time 14.25
INSTRUM spect
PROBHD 5 mm CPTCI 1H-
PULPROG deptsp135
TD 32768
SOLVENT MeOD
NS 3090
DS 2
SWH 30303.031 Hz
FIDRES 0.924775 Hz
AQ 0.5406720 sec
RG 32768
DW 16.500 usec
DE 6.50 usec
TE 298.0 K
CNST2 145.0000000
D1 1.50000000 sec
D2 0.00344828 sec
D12 0.00002000 sec
TD0 4

======== CHANNEL f1 ========
NUC1 13C
P1 15.40 usec
P12 2000.00 usec
PL0 120.00 dB
PL1 1.00 dB
PL0W 0 W
PL1W 83.60149384 W
SFO1 150.9430468 MHz
SP2 5.40 dB
SPNAM[2] Crp60comp.4
SPOAL2 0.500
SPOFFS2 0 Hz

======== CHANNEL f2 ========
CPDPRG[2 waltz16
NUC2 1H
P3 7.50 usec
P4 15.00 usec
PCPD2 65.00 usec
PL2 3.30 dB
PL12 22.06 dB
PL2W 9.16420078 W
PL12W 0.12192553 W
SFO2 600.2324009 MHz

F2 - Processing parameters
SI 16384
SF 150.9277403 MHz
WDW EM
SSB 0
LB 1.00 Hz
GB 0
PC 1.00

compound 7

MEHWISH/DR.IQBAL/MK.14/CD3OD
DEPT 90

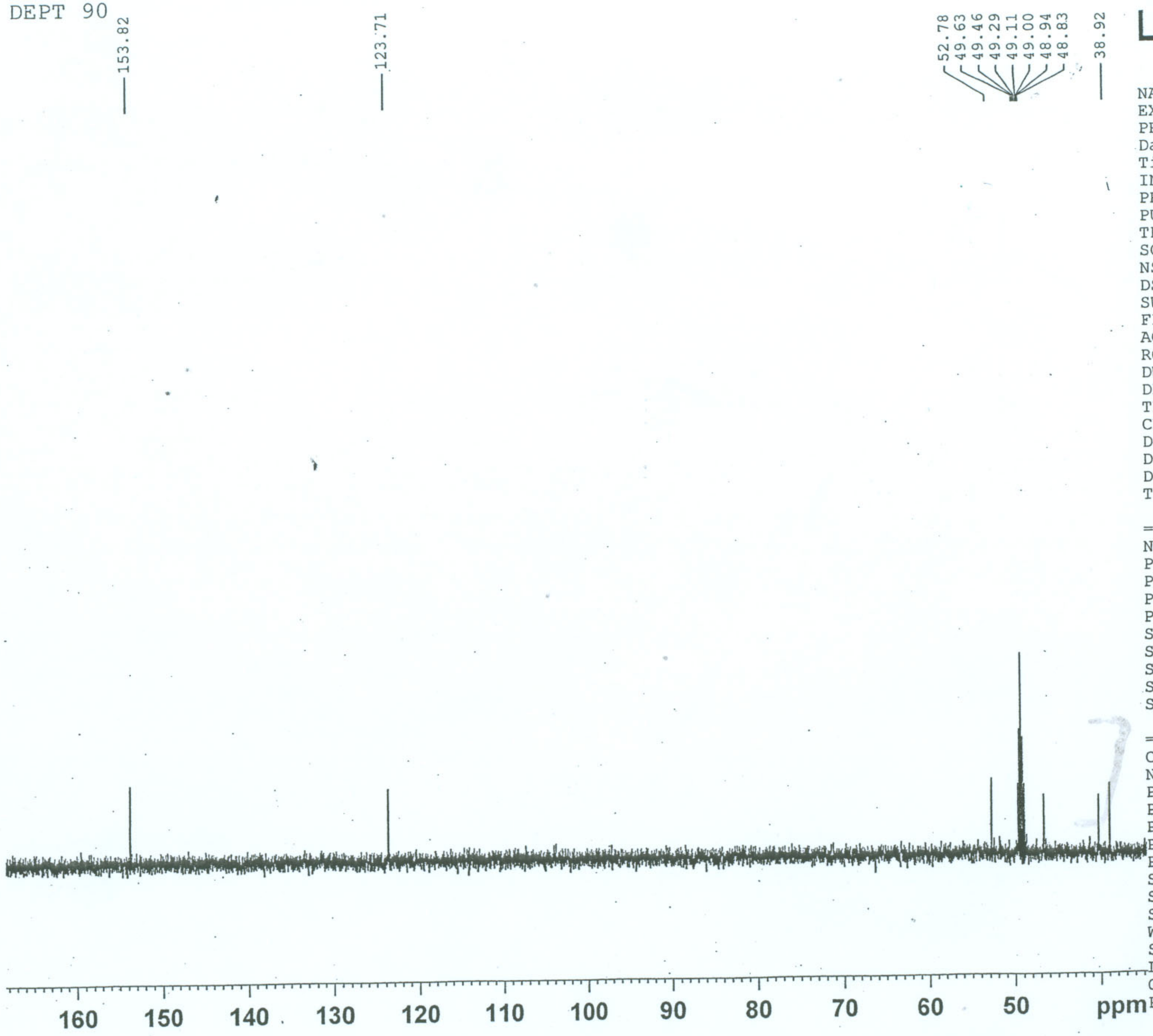

AVANCE AV-500
LAB NO:118

NAME	dec15-16
EXPNO	8
PROCNO	1
Date_	20161217
Time	10.45
INSTRUM	spect
PROBHD	5 mm PABBI 1H/
PULPROG	deptsp90
TD	32768
SOLVENT	MeOD
NS	6144
DS	4
SWH	24752.475 Hz
FIDRES	0.755386 Hz
AQ	0.6619838 sec
RG	32768
DW	20.200 usec
DE	6.50 usec
TE	296.5 K
CNST2	145.0000000
D1	1.50000000 sec
D2	0.00344828 sec
D12	0.00002000 sec
TD0	6

======== CHANNEL f1 ========

NUC1	13C
P1	13.35 usec
P12	2000.00 usec
PL0	120.00 dB
PL1	-3.00 dB
SFO1	125.7950092 MHz
SP2	2.65 dB
SPNAM2	Crp60comp.4
SPOAL2	0.500
SPOFFS2	0.00 Hz

======== CHANNEL f2 ========

CPDPRG2	waltz16
NUC2	1H
P3	8.03 usec
P4	16.06 usec
PCPD2	80.00 usec
PL2	3.00 dB
PL12	22.74 dB
SFO2	500.2330014 MHz
SI	32768
SF	125.7827558 MHz
WDW	EM
SSB	0
LB	1.00 Hz
GB	0
PC	0.80

compound 7

MEHWISH/DR.IQBAL/MK.14/CD3OD
HMBC

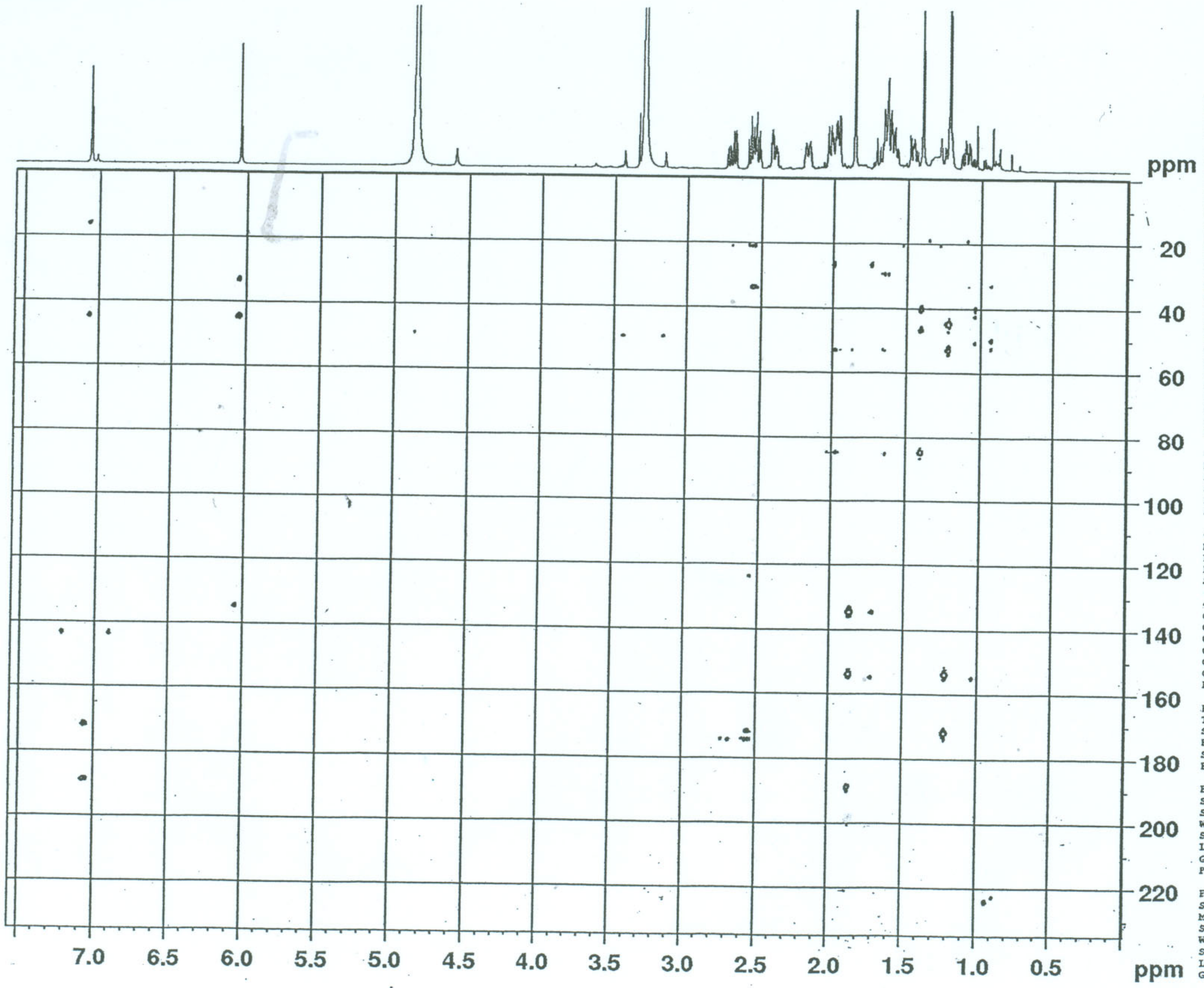

Current Data Parameters	
NAME	mehwish- MK-14
EXPNO	5
PROCNO	1
F2 - Acquisition Parameters	
Date_	20161216
Time	4.57
INSTRUM	spect
PROBHD	5 mm PABBI 1H/
PULPROG	hmbcgplpndqf
TD	2048
SOLVENT	MeOD
NS	64
DS	16
SWH	3787.879 Hz
FIDRES	1.849550 Hz
AQ	0.2703360 sec
RG	20642.5
DW	132.000 usec
DE	6.50 usec
TE	297.6 K
CNST2	145.0000000
CNST13	10.0000000
D0	0.00000300 sec
D1	2.00000000 sec
D2	0.00344828 sec
D6	0.05000000 sec
D16	0.00020000 sec
IN0	0.00001690 sec
======== CHANNEL f1 ========	
NUC1	1H
P1	8.03 usec
P2	16.06 usec
PL1	3.00 dB
SFO1	500.2319009 MHz
======== CHANNEL f2 ========	
NUC2	13C
P3	13.35 usec
PL2	-3.00 dB
SFO2	125.7974871 MHz
====== GRADIENT CHANNEL =====	
GPNAM[1]	SINE.100
GPNAM[2]	SINE.100
GPNAM[3]	SINE.100
GPZ1	50.00 %
GPZ2	30.00 %
GPZ3	40.10 %
P16	1000.00 usec
F1 - Acquisition parameters	
TD	256
SFO1	125.7975 MHz
FIDRES	230.956329 Hz
SW	235.000 ppm
FnMODE	QF
F2 - Processing parameters	
SI	1024
SF	500.2300118 MHz
WDW	SINE
SSB	0
LB	0 Hz
GB	0
PC	1.00
F1 - Processing parameters	
SI	512
MC2	QF
SF	125.7827558 MHz
WDW	SINE
SSB	0
LB	0 Hz
GB	0

MEHWISH/DR.IQBAL/MK.14/CD3OD
HSQC

compound 7

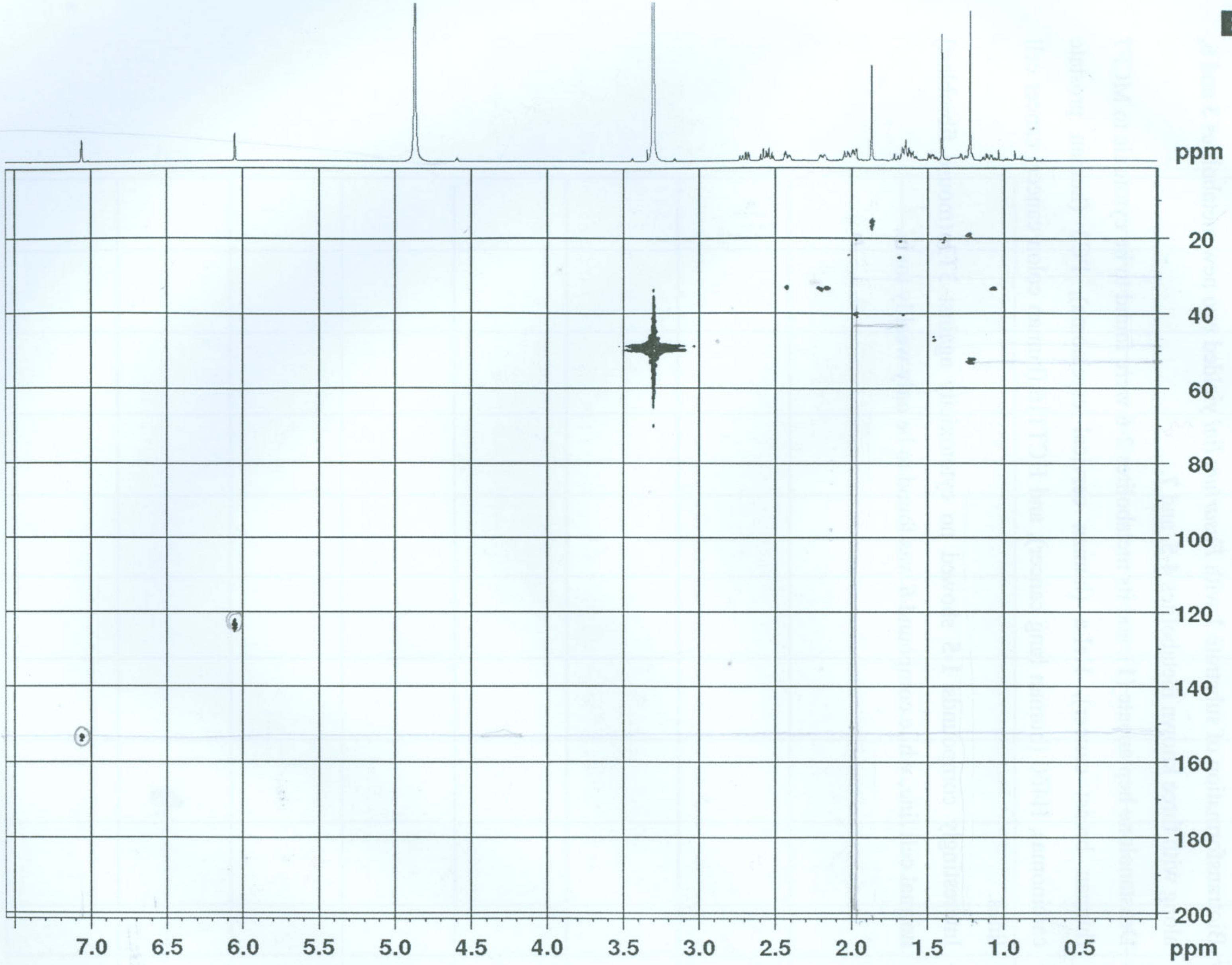

BRUKER

```
NAME      [illegible]
EXPNO                4
PROCNO               1

F2 - Acquisition Parameters
Date_         20161216
Time              1.09
INSTRUM          spect
PROBHD   5 mm PABBI 1H/
PULPROG     hsqcedetgp
TD                1024
SOLVENT           MeOD
NS                  32
DS                  16
SWH           3787.879 Hz
FIDRES        3.699100 Hz
AQ           0.1351680 sec
RG             18390.4
DW             132.000 usec
DE                6.50 usec
TE               297.4 K
CNST2      145.0000000
D0          0.00000300 sec
D1          1.50000000 sec
D4          0.00172414 sec
D11         0.03000000 sec
D13         0.00000400 sec
D16         0.00020000 sec
D21         0.00345000 sec
IN0         0.00001985 sec
ZGOPTNS

======== CHANNEL f1 ========
NUC1                1H
P1                8.03 usec
P2               16.06 usec
P28            1000.00 usec
PL1               3.00 dB
SFO1       500.2319009 MHz

======== CHANNEL f2 ========
CPDPRG[2          garp
NUC2               13C
P3               13.35 usec
P4               26.70 usec
PCPD2            70.00 usec
PL2              -3.00 dB
PL12             10.00 dB
SFO2       125.7955123 MHz

====== GRADIENT CHANNEL =====
GPNAM[1]      SINE.100
GPNAM[2]      SINE.100
GPZ1             80.00 %
GPZ2             20.10 %
P16            1000.00 usec

F1 - Acquisition parameters
TD                 256
SFO1          125.7955 MHz
FIDRES      196.555481 Hz
SW             200.000 ppm
FnMODE   Echo-Antiecho

F2 - Processing parameters
SI                1024
SF         500.2300118 MHz
WDW              QSINE
SSB                  2
LB        0 Hz
GB        0
PC                0.80

F1 - Processing parameters
SI                1024
MC2     echo-antiecho
SF         125.7827558 MHz
WDW              QSINE
SSB                  2
LB        0 Hz
GB        0
```

MEHWISH/DR.IQBAL/MK.14/CD3OD
ICCBS/U.O.K
COSY

Compound 7

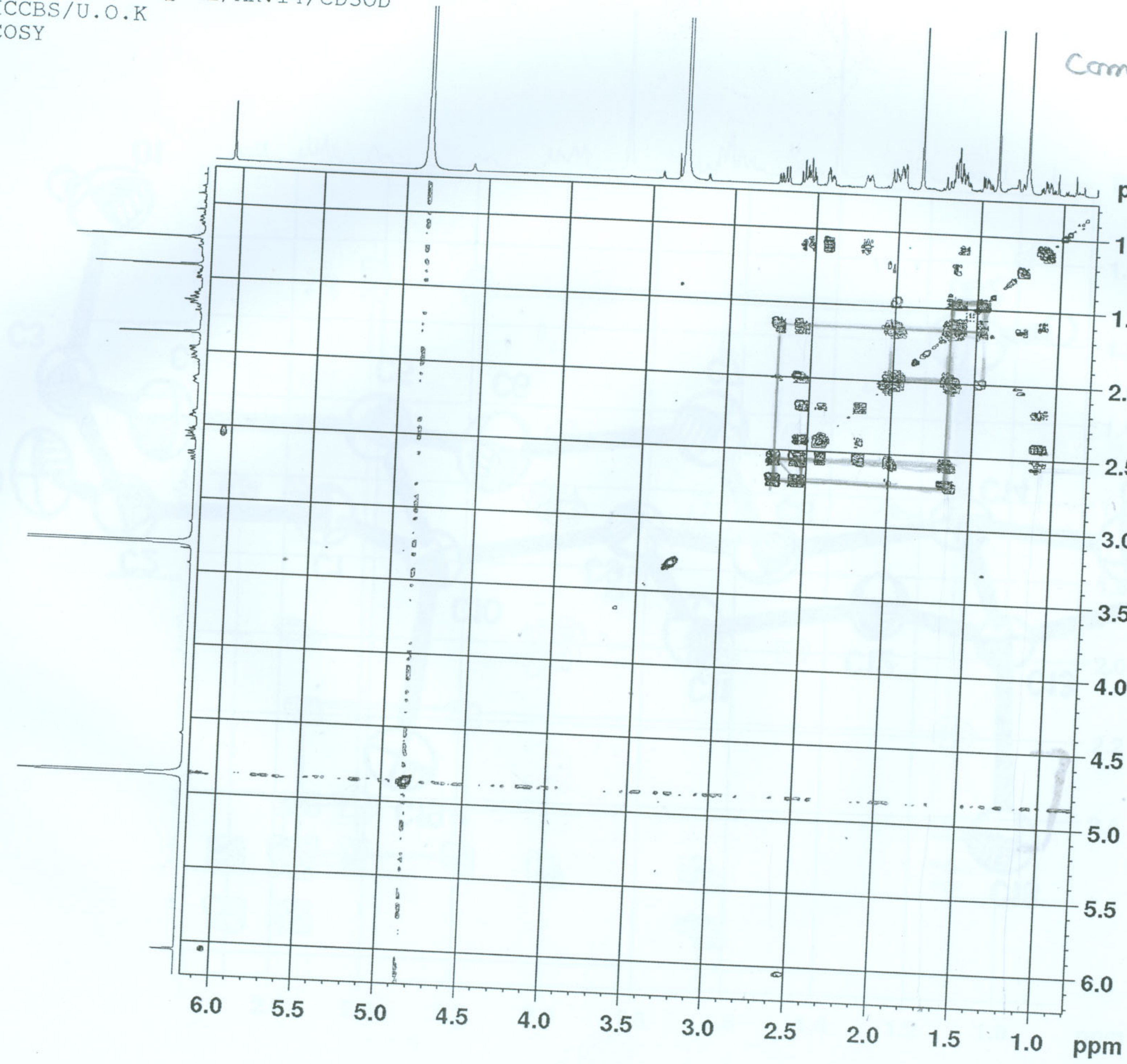

Supplement: Supplementary file 7 [file DataSheet7.PDF]
