# Supplementary material for: Bio-Catalytic Structural Transformation of Anti-cancer Steroid, Drostanolone Enanthate with Cephalosporium aphidicola and Fusarium lini, and Cytotoxic Potential Evaluation of Its Metabolites against Certain Cancer Cell Lines
Source: Front Pharmacol. 2017 Dec 20;8:900. doi: 10.3389/fphar.2017.00900 (PMC5742531; doi:10.3389/fphar.2017.00900)
Supplement: Supplementary file 8 [file DataSheet8.PDF]

File Name	:	d:\mswin\data\30-d.mss
Creation Date/Time	:	15.06.16 at 20:30:19
File Type	:	Lo-Res Mass Data (Centroid)
File Source	:	Acquired on MASPEC system [msw/A091]
File Title	:	MAHWISH / DR. M. IQBAL
Operator	:	Barkat Ali
Instrument	:	MAT312---EI

Comp. 8

SCAN GRAPH. Flagging=M/z.
Scan 18-9:18. Entries=211. 100% Int.=33762.

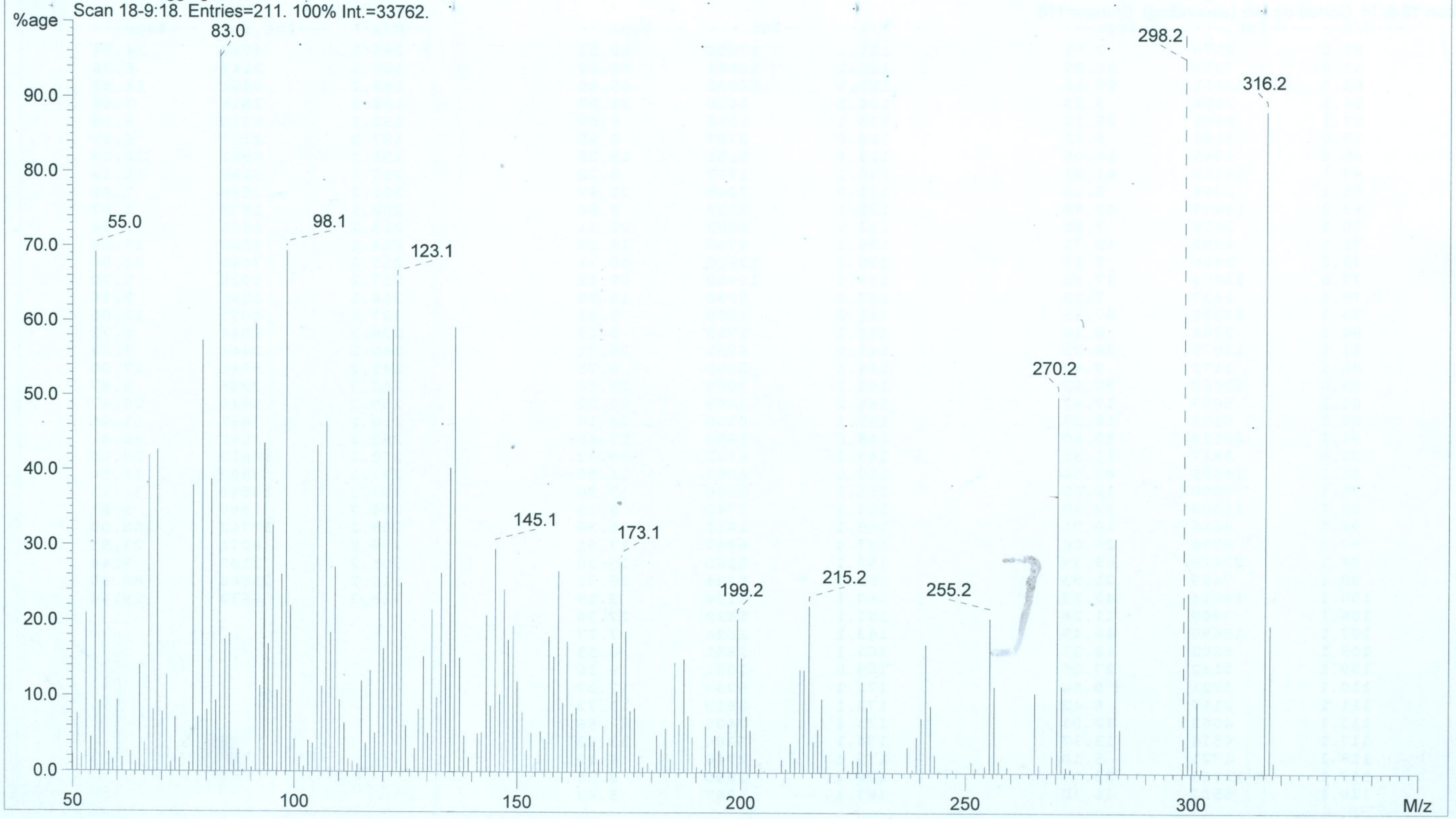

comp. 8

Mass	Relative Intensity	Theoretical Mass	Delta [ppm]	Delta [mmu]	RDB	Composition
199.0363	2.7	199.0395	-16.4	-3.3	9.5	$C_{12} H_7 O_3$
200.0082	2.5	200.0110	-13.7	-2.7	10.0	$C_{11} H_4 O_4$
202.0218	2.3	202.0266	-23.7	-4.8	9.0	$C_{11} H_6 O_4$
214.9999	3.8					
218.8363	3.6					
230.8567	4.8					
242.7990	5.0					
270.2035	7.0	270.1984	19.0	5.1	7.0	$C_{19} H_{26} O_1$
283.1675	6.5	283.1698	-8.3	-2.3	8.5	$C_{19} H_{23} O_2$
298.1921	11.5	298.1933	-3.8	-1.1	8.0	$C_{20} H_{26} O_2$
299.1939	2.6	299.2011	-24.2	-7.2	7.5	$C_{20} H_{27} O_2$
316.2042	3.7	316.2038	1.3	0.4	7.0	$C_{20} H_{28} O_3$

[illegible], University of Karachi Analytical Laboratory - Pakistan

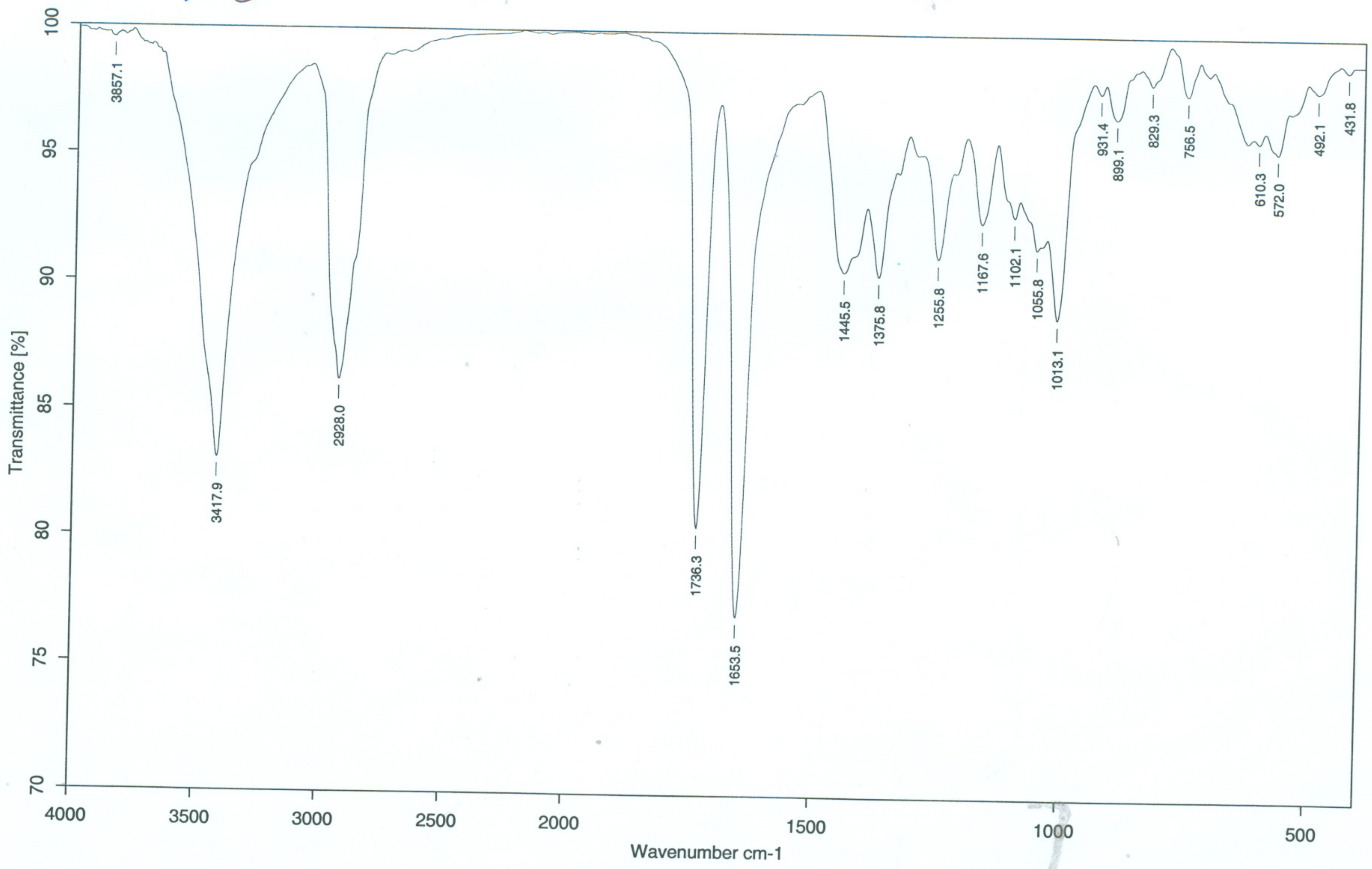

Sample :	30-D/MAHWISH	Spectrum	: 30-D.0 (in D:\IRSTUDENT)
Measured :	14/07/2016 on VECTOR22	Technic	: Solid
Resolution :	4 cm-1 (10 scans)	Analyst	: ZA/JAM

AVANCE AV-400 MHz
Lab # 115

MAHWISH/DR.IQBAL/30.D
1H/.

For C 13

Comp. 8

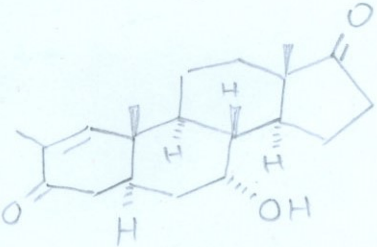

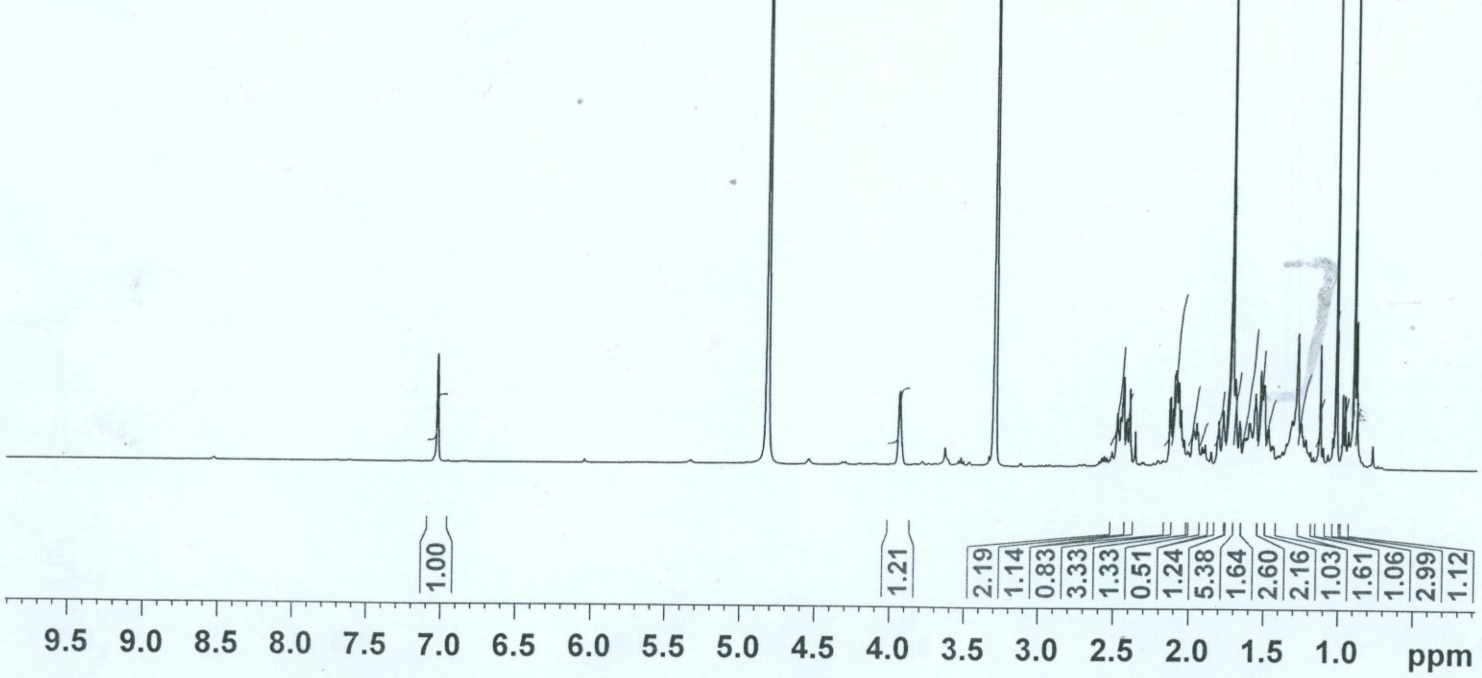

NAME	may27-16
EXPNO	6
PROCNO	1
Date_	20160527
Time	13.32
INSTRUM	spect
PROBHD	5 mm SEI 1H-13
PULPROG	zg30
TD	65536
SOLVENT	MeOD
NS	128
DS	0
SWH	8012.820 Hz
FIDRES	0.122266 Hz
AQ	4.0894966 sec
RG	228.1
DW	62.400 usec
DE	6.50 usec
TE	300.0 K
D1	2.00000000 sec
TD0	1

======== CHANNEL f1 ========

NUC1	1H
P1	10.80 usec
PL1	3.00 dB
SFO1	400.0332002 MHz
SI	32768
SF	400.0300087 MHz
WDW	EM
SSB	0
LB	0.30 Hz
GB	0
PC	0.20

Mahwish / Dr. Iqbal / 30-D
BB

Comp. 8

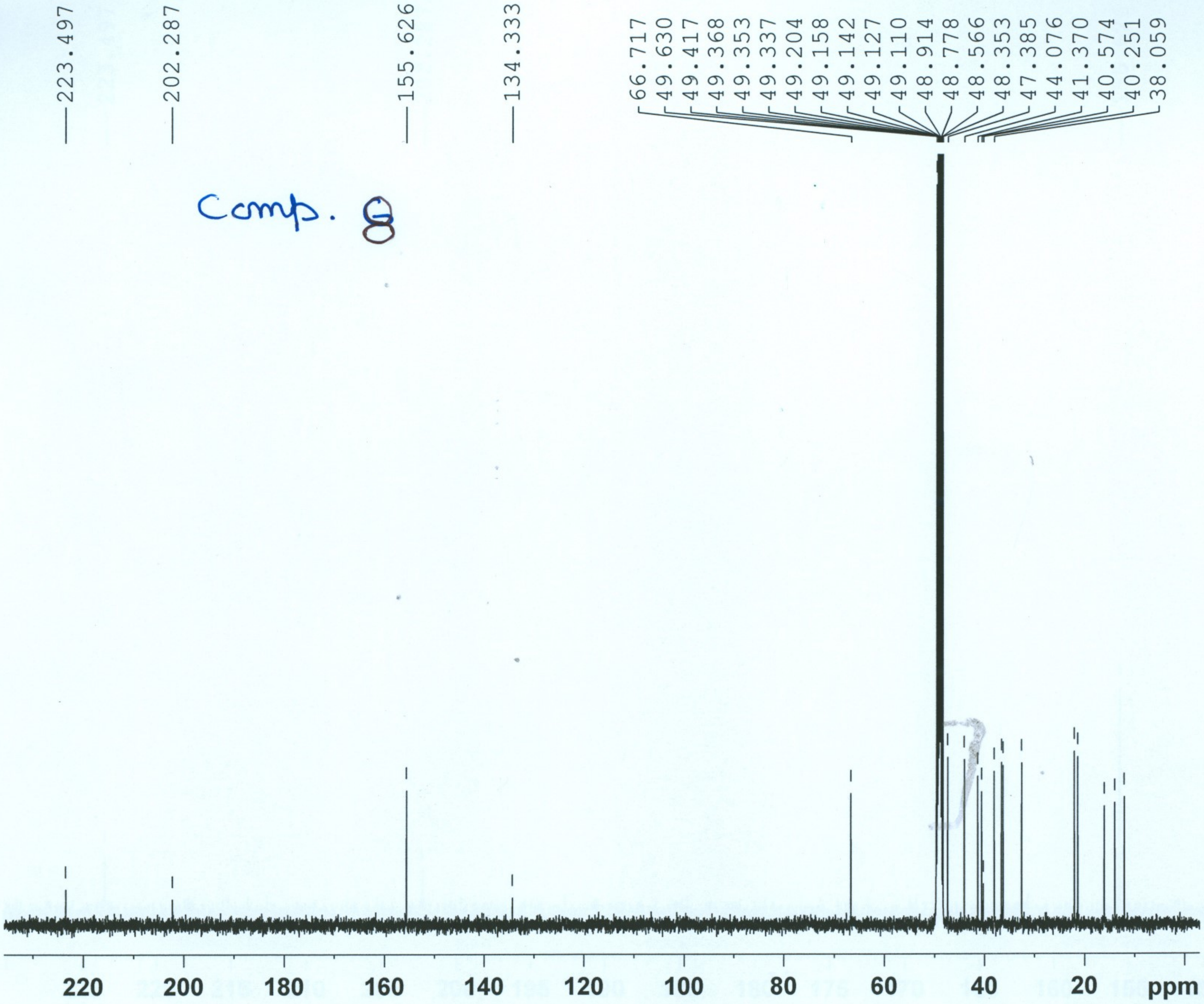

AVANCE AV-III HD
400 MHz
LAB #109A

```
Current Data Parameters
NAME              jun17-16
EXPNO                    6
PROCNO                   1

F2 - Acquisition Parameters
Date_             20160618
Time                 14.53
INSTRUM              spect
PROBHD    5 mm SEI 1H/D-
PULPROG               zgpg
TD                   32768
SOLVENT               MeOD
NS                   20480
DS                       4
SWH              24038.461 Hz
FIDRES            0.733596 Hz
AQ               0.6815744 sec
RG                  202.75
DW                  20.800 usec
DE                    6.50 usec
TE                   298.6 K
D1              1.50000000 sec
D11             0.03000000 sec
TD0                     20

======== CHANNEL f1 ========
SFO1           100.6746353 MHz
NUC1                   13C
P1                   11.00 usec
PLW1          150.00000000 W

======== CHANNEL f2 ========
SFO2           400.3316013 MHz
NUC2                    1H
CPDPRG[2           waltz16
PCPD2                90.00 usec
PLW2           20.00000000 W
PLW12           0.08984600 W
PLW13           0.07277500 W

F2 - Processing parameters
SI                   32768
SF             100.6629178 MHz
WDW                     EM
SSB      0
LB                    1.00 Hz
GB       0
PC                    1.40
```

Mahwish / Dr. Iqbal / 30-D
Dept90

comp. 8

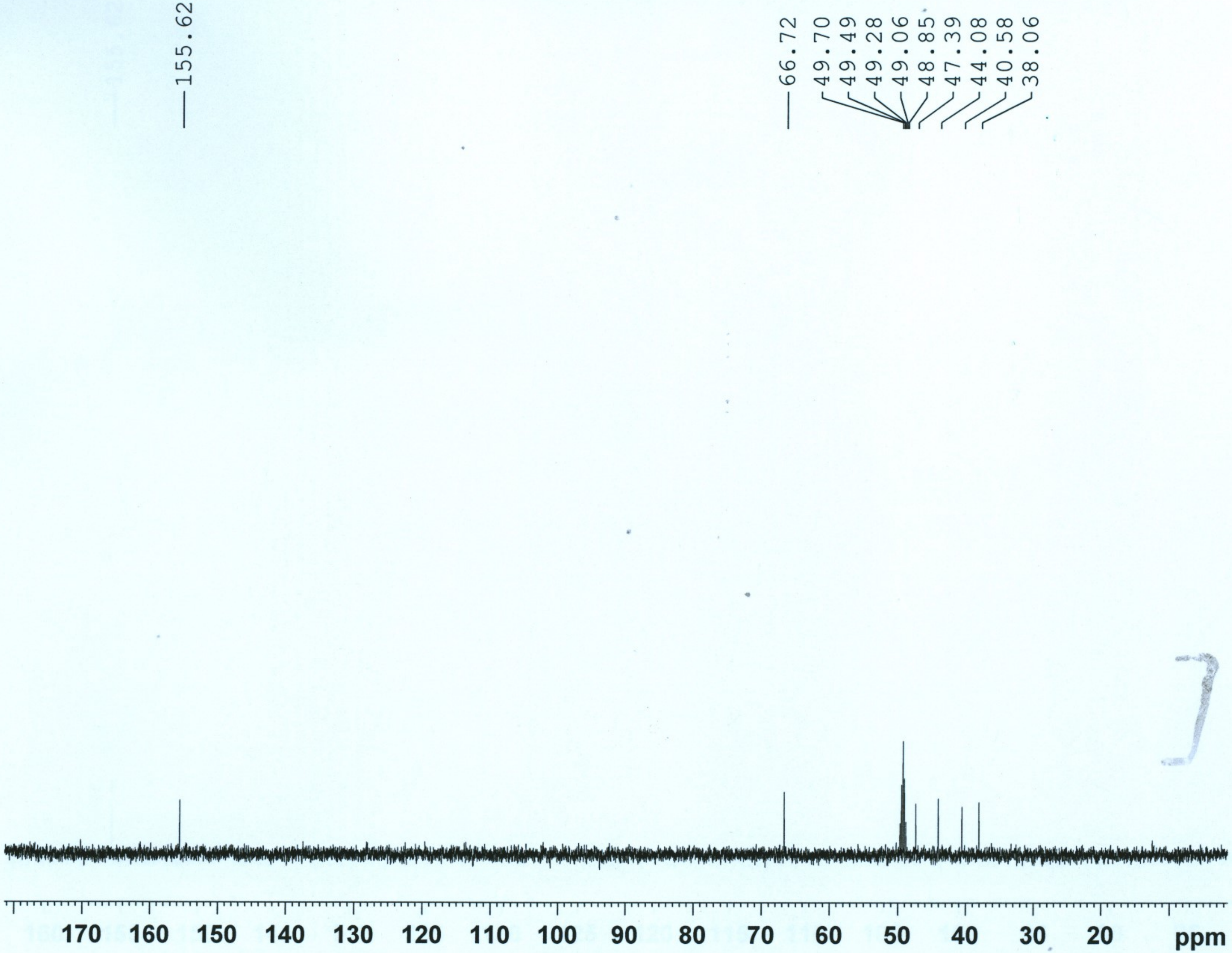

AVANCE AV-III HD
400 MHz
LAB #109A

```
Current Data Parameters
NAME              jun17-16
EXPNO                    8
PROCNO                   1

F2 - Acquisition Parameters
Date_             20160619
Time                 10.43
INSTRUM              spect
PROBHD     5 mm SEI 1H/D-
PULPROG           deptsp90
TD                   32768
SOLVENT               MeOD
NS                    1886
DS                       4
SWH              18115.941 Hz
FIDRES            0.552855 Hz
AQ               0.9043968 sec
RG                  202.75
DW                  27.600 usec
DE                    6.50 usec
TE                   299.1 K
CNST2          142.0000000
D1              1.50000000 sec
D2              0.00352113 sec
D12             0.00002000 sec
TD0                      4

======== CHANNEL f1 ========
SFO1        100.6721187 MHz
NUC1                    13C
P1                    11.00 usec
P13                 2000.00 usec
PLW0       0 W
PLW1        150.00000000 W
SPNAM[5]      Crp60comp.4
SPOAL5                0.500
SPOFFS5    0 Hz
SPW5         27.73100090 W

======== CHANNEL f2 ========
SFO2        400.3316013 MHz
NUC2                     1H
CPDPRG[2            waltz16
P3                     6.80 usec
P4                    13.60 usec
PCPD2                 90.00 usec
PLW2            20.00000000 W
PLW12            0.11417000 W

F2 - Processing parameters
SI                    32768
SF            100.6629178 MHz
WDW                      EM
SSB        0
LB                     1.00 Hz
GB         0
PC                     1.40
```

Mahwish / Dr. Iqbal / 30-D
BB

Comp. 8

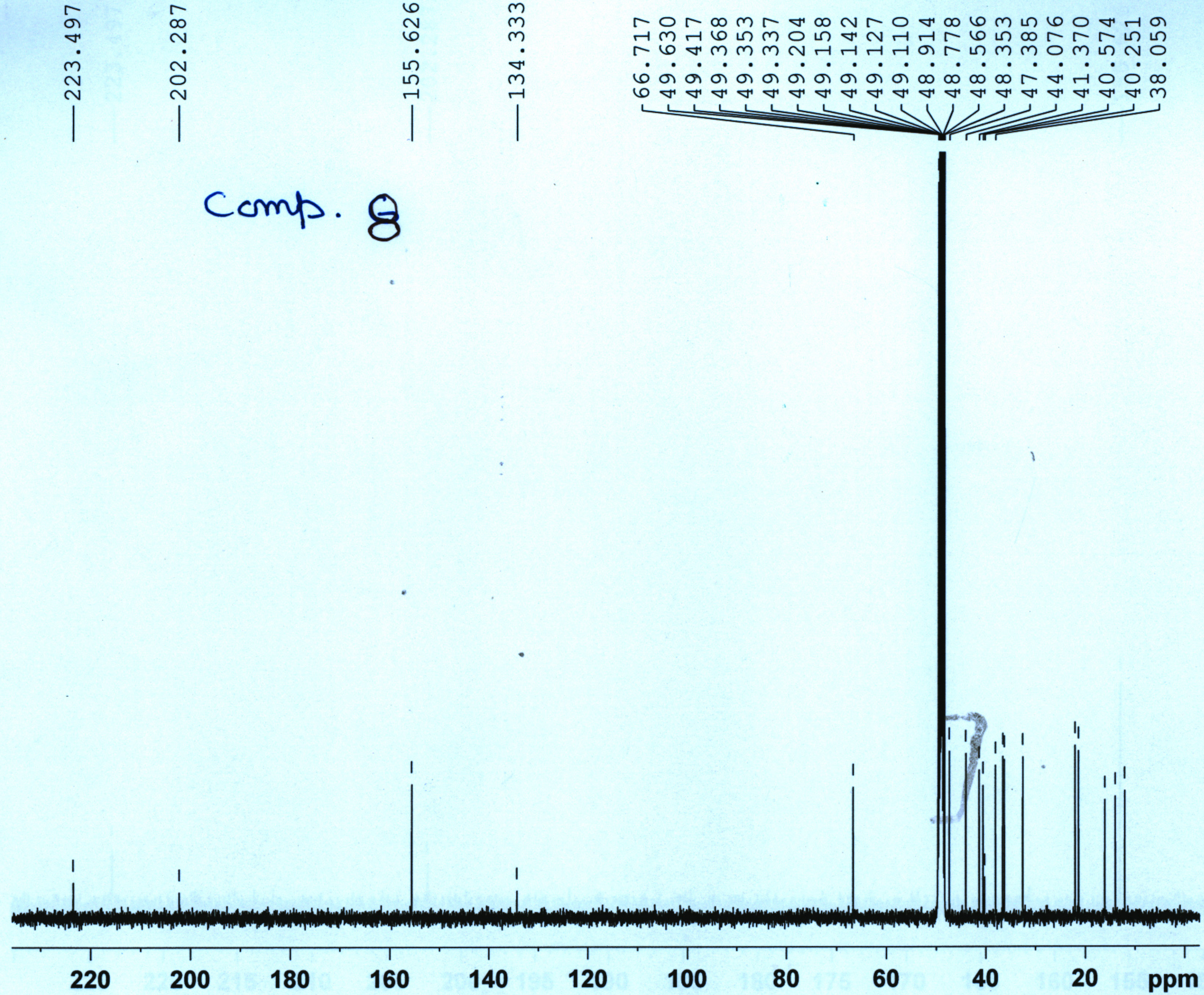

AVANCE AV-III HD
400 MHz
LAB #109A

Current Data Parameters

NAME	jun17-16
EXPNO	6
PROCNO	1

F2 - Acquisition Parameters

Date_	20160618
Time	14.53
INSTRUM	spect
PROBHD	5 mm SEI 1H/D-
PULPROG	zgpg
TD	32768
SOLVENT	MeOD
NS	20480
DS	4
SWH	24038.461 Hz
FIDRES	0.733596 Hz
AQ	0.6815744 sec
RG	202.75
DW	20.800 usec
DE	6.50 usec
TE	298.6 K
D1	1.50000000 sec
D11	0.03000000 sec
TD0	20

======== CHANNEL f1 ========

SFO1	100.6746353 MHz
NUC1	13C
P1	11.00 usec
PLW1	150.00000000 W

======== CHANNEL f2 ========

SFO2	400.3316013 MHz
NUC2	1H
CPDPRG[2	waltz16
PCPD2	90.00 usec
PLW2	20.00000000 W
PLW12	0.08984600 W
PLW13	0.07277500 W

F2 - Processing parameters

SI	32768
SF	100.6629178 MHz
WDW	EM
SSB	0
LB	1.00 Hz
GB	0
PC	1.40

Mahwish / Dr. Iqbal / 30-D
HSQC

Comp. 8

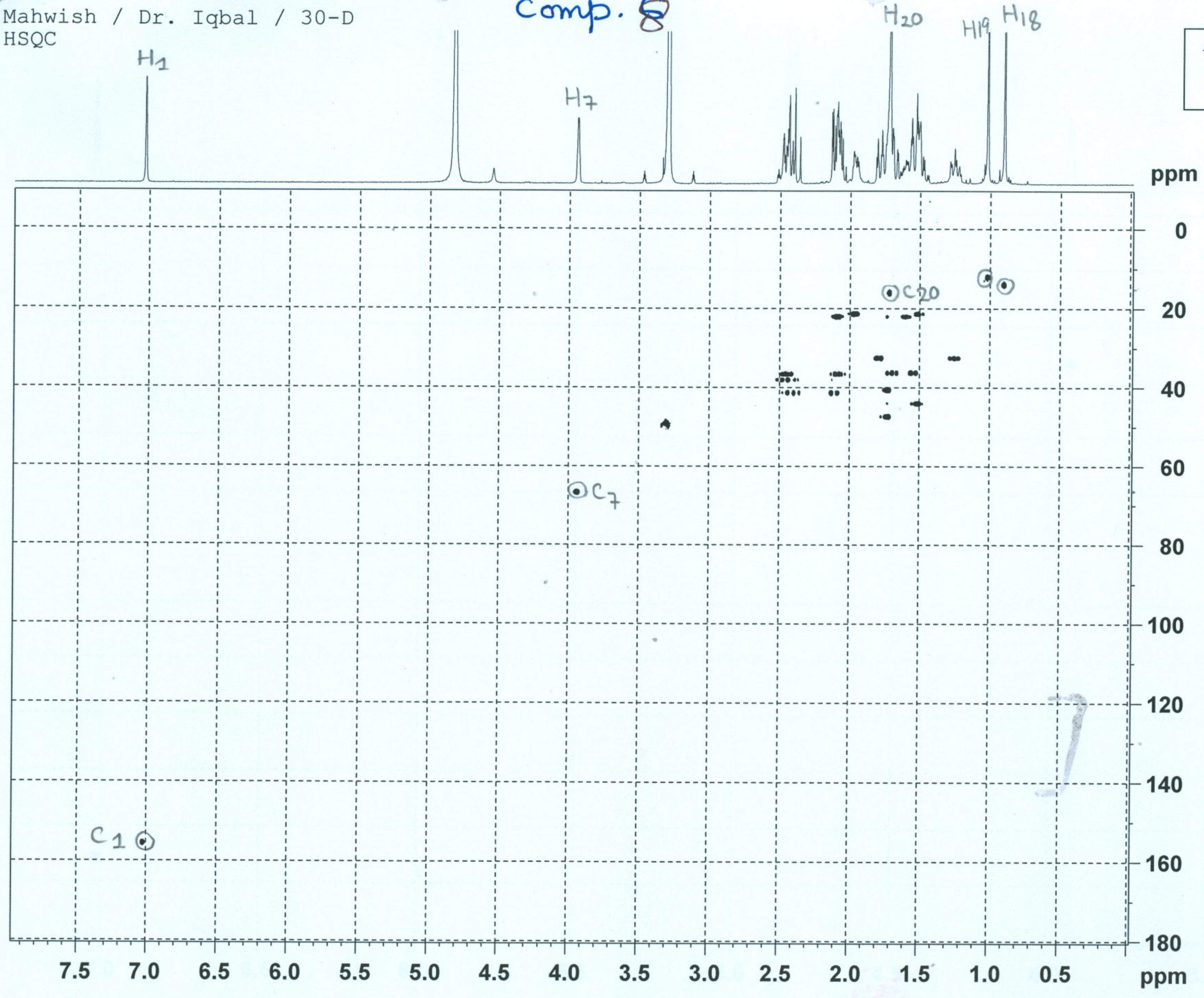

AVANCE AV-III HD
400 MHz
LAB #109A

```
Current Data Parameters
NAME              jun17-16
EXPNO                    4
PROCNO                   1

F2 - Acquisition Parameters
Date_             20160617
Time                 23.12
INSTRUM              spect
PROBHD    5 mm SEI 1H/D-
PULPROG         hsqcetgpsi
TD                    2048
SOLVENT               MeOD
NS                      32
DS                      16
SWH               3201.024 Hz
FIDRES            1.563000 Hz
AQ               0.3198976 sec
RG                  202.75
DW                 156.200 usec
DE                    6.50 usec
TE                   298.8 K
CNST2          145.0000000
D0              0.00000300 sec
D1              1.50000000 sec
D4              0.00172414 sec
D11             0.03000000 sec
D16             0.00020000 sec
D24             0.00089000 sec
IN0             0.00002610 sec
ZGOPTNS

======== CHANNEL f1 ========
SFO1           400.3316013 MHz
NUC1                    1H
P1                    6.60 usec
P2                   13.20 usec
P28                1000.00 usec
PLW1           20.00000000 W

======== CHANNEL f2 ========
SFO2           100.6715147 MHz
NUC2                   13C
CPDPRG[2              garp
P3                   11.00 usec
P4                   22.00 usec
PCPD2                84.00 usec
PLW2          150.00000000 W
PLW12           2.57229996 W

====== GRADIENT CHANNEL =====
GPNAM[1]        SMSQ10.100
GPNAM[2]        SMSQ10.100
GPZ1                 80.00 %
GPZ2                 20.10 %
P16                1000.00 usec

F1 - Acquisition parameters
TD                     256
SFO1              100.6715 MHz
FIDRES           74.832375 Hz
SW                 190.293 ppm
FnMODE      Echo-Antiecho

F2 - Processing parameters
SI                    1024
SF             400.3300117 MHz
WDW                  QSINE
SSB                      2
LB              0 Hz
GB              0
PC                    1.40

F1 - Processing parameters
SI                    1024
MC2         echo-antiecho
SF             100.6629178 MHz
WDW                  QSINE
SSB                      2
LB              0 Hz
GB              0
```

Mahwish / Dr. Iqbal / 30-D
HMBC

comp. 8

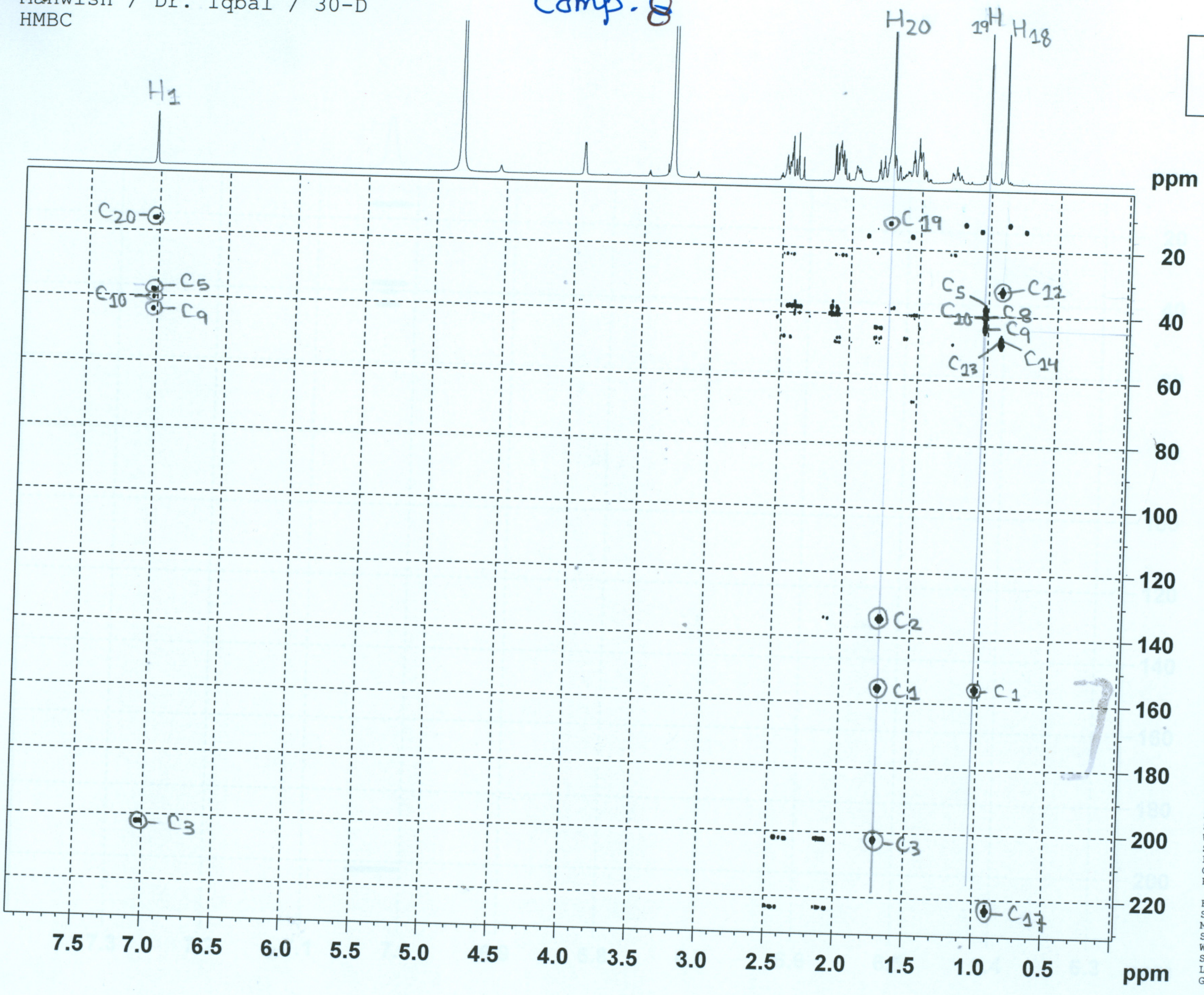

AVANCE AV-III HD
400 MHz
LAB #109A

Current Data Parameters	
NAME	jun17-16
EXPNO	5
PROCNO	1
F2 - Acquisition Parameters	
Date_	20160618
Time	3.26
INSTRUM	spect
PROBHD	5 mm SEI 1H/D-
PULPROG	hmbcgplpndqf
TD	2048
SOLVENT	MeOD
NS	64
DS	16
SWH	3201.024 Hz
FIDRES	1.563000 Hz
AQ	0.3198976 sec
RG	202.75
DW	156.200 usec
DE	6.50 usec
TE	299.0 K
CNST2	145.0000000
CNST13	10.0000000
D0	0.00000300 sec
D1	2.00000000 sec
D2	0.00344828 sec
D6	0.05000000 sec
D16	0.00020000 sec
IN0	0.00002160 sec
======== CHANNEL f1 ========	
SFO1	400.3316013 MHz
NUC1	1H
P1	6.70 usec
P2	13.40 usec
PLW1	20.00000000 W
======== CHANNEL f2 ========	
SFO2	100.6746353 MHz
NUC2	13C
P3	11.00 usec
PLW2	150.00000000 W
====== GRADIENT CHANNEL =====	
GPNAM[1]	SMSQ10.100
GPNAM[2]	SMSQ10.100
GPNAM[3]	SMSQ10.100
GPZ1	50.00 %
GPZ2	30.00 %
GPZ3	40.10 %
P16	1000.00 usec
F1 - Acquisition parameters	
TD	256
SFO1	100.6746 MHz
FIDRES	90.422455 Hz
SW	229.930 ppm
FnMODE	QF
F2 - Processing parameters	
SI	2048
SF	400.3300117 MHz
WDW	SINE
SSB	0
LB	0 Hz
GB	0
PC	1.40
F1 - Processing parameters	
SI	1024
MC2	QF
SF	100.6629178 MHz
WDW	SINE
SSB	0
LB	0 Hz
GB	0

Mahwish / Dr. Iqbal / 30-D
COSY

comp. 8

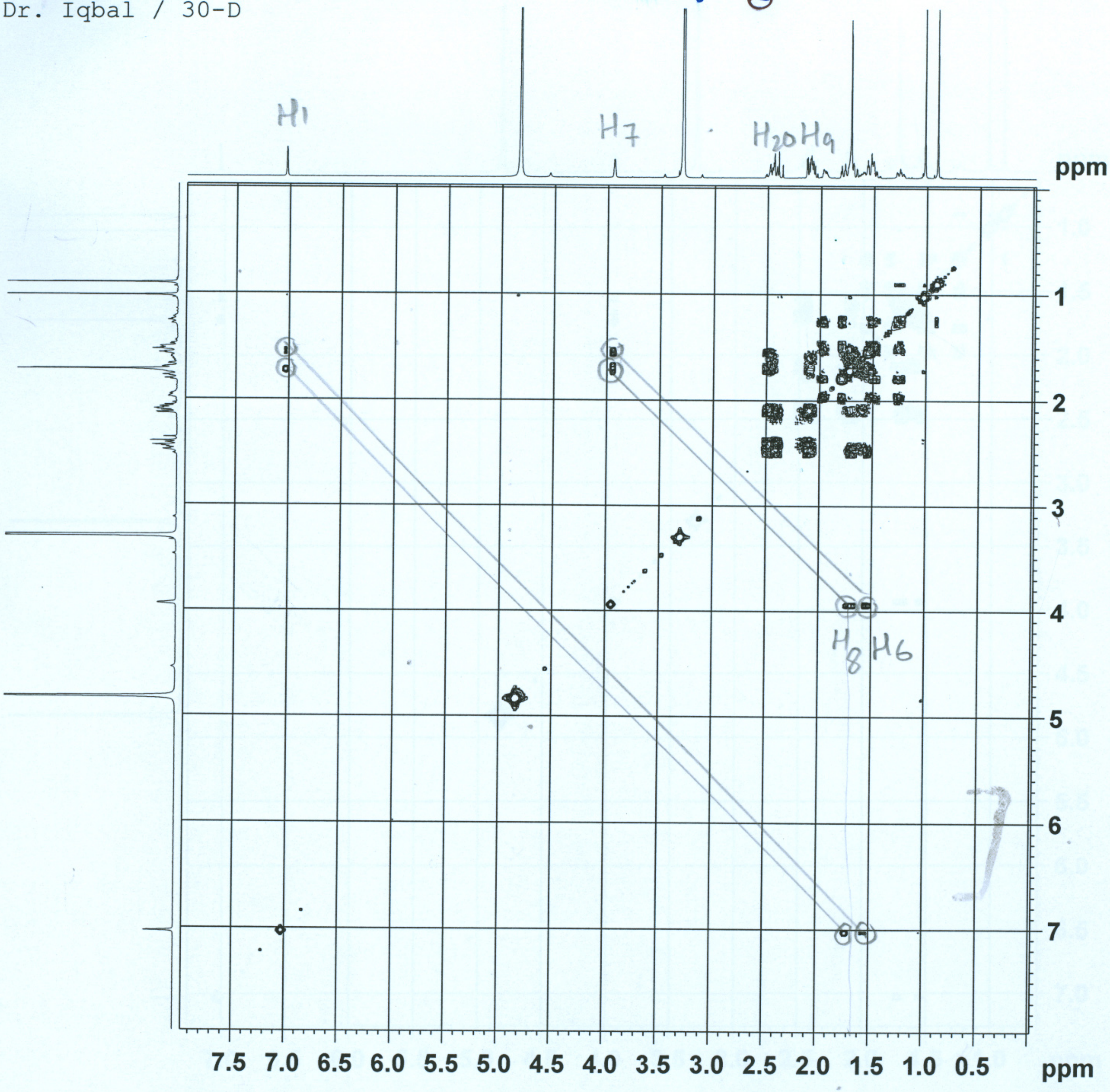

AVANCE AV-III HD
400 MHz
LAB #109A

Current Data Parameters
NAME jun17-16
EXPNO 2
PROCNO 1

F2 - Acquisition Parameters
Date_ 20160617
Time 11.47
INSTRUM spect
PROBHD 5 mm SEI 1H/D-
PULPROG cosygpqf
TD 2048
SOLVENT MeOD
NS 32
DS 4
SWH 3201.024 Hz
FIDRES 1.563000 Hz
AQ 0.3198976 sec
RG 202.75
DW 156.200 usec
DE 6.50 usec
TE 300.2 K
D0 0.00000300 sec
D1 2.00000000 sec
D13 0.00000400 sec
D16 0.00020000 sec
IN0 0.00031240 sec

======== CHANNEL f1 ========
SFO1 400.3316013 MHz
NUC1 1H
P0 6.70 usec
P1 6.70 usec
PLW1 20.00000000 W

====== GRADIENT CHANNEL =====
GPNAM[1] SMSQ10.100
GPZ1 10.00 %
P16 1000.00 usec

F1 - Acquisition parameters
TD 256
SFO1 400.3316 MHz
FIDRES 12.504002 Hz
SW 7.996 ppm
FnMODE QF

F2 - Processing parameters
SI 2048
SF 400.3300117 MHz
WDW QSINE
SSB 0
LB 0 Hz
GB 0
PC 1.40

F1 - Processing parameters
SI 2048
MC2 QF
SF 400.3300117 MHz
WDW QSINE
SSB 0
LB 0 Hz
GB 0

Comp. 8

Mahwish / Dr. Iqbal / 30-D
NOESY

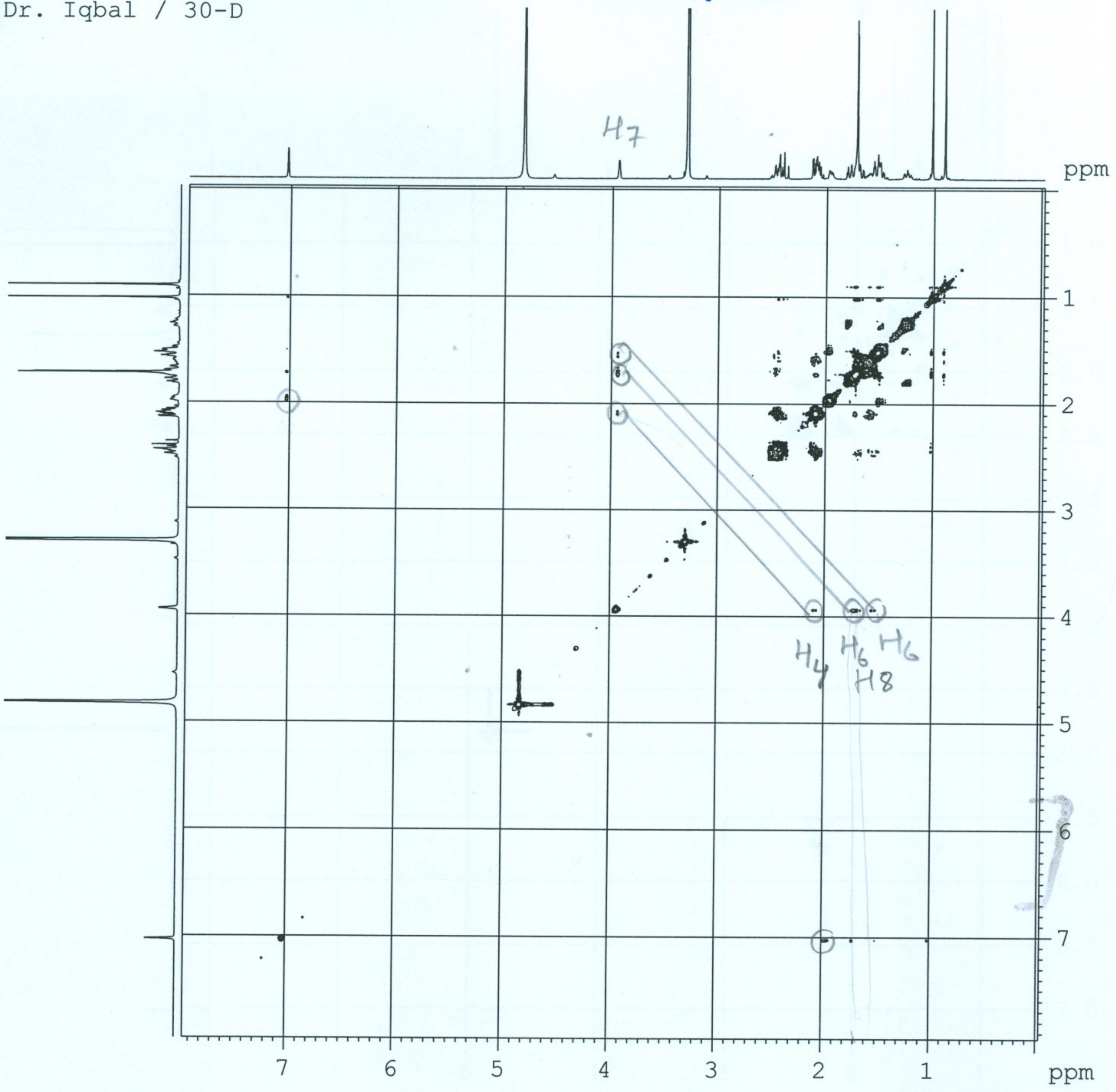

AVANCE AV-III HD
400 MHz
LAB #109A

Current Data Parameters
NAME jun17-16
EXPNO 3
PROCNO 1

F2 - Acquisition Parameters
Date_ 20160617
Time 17.11
INSTRUM spect
PROBHD 5 mm SEI 1H/D-
PULPROG noesygpph
TD 2048
SOLVENT MeOD
NS 32
DS 8
SWH 3201.024 Hz
FIDRES 1.563000 Hz
AQ 0.3198976 sec
RG 202.75
DW 156.200 usec
DE 6.50 usec
TE 298.8 K
D0 0.00014767 sec
D1 2.00000000 sec
D8 0.30000001 sec
D16 0.00020000 sec
IN0 0.00031240 sec

======== CHANNEL f1 ========
SFO1 400.3316013 MHz
NUC1 1H
P1 6.70 usec
P2 13.40 usec
PLW1 20.00000000 W

====== GRADIENT CHANNEL =====
GPNAM[1] SMSQ10.100
GPZ1 40.00 %
P16 1000.00 usec

F1 - Acquisition parameters
TD 256
SFO1 400.3316 MHz
FIDRES 12.504002 Hz
SW 7.996 ppm
FnMODE States-TPPI

F2 - Processing parameters
SI 1024
SF 400.3300117 MHz
WDW QSINE
SSB 2
LB 0 Hz
GB 0
PC 1.40

F1 - Processing parameters
SI 1024
MC2 States-TPPI
SF 400.3300117 MHz
WDW QSINE
SSB 2
LB 0 Hz
GB 0
