# Supplementary material for: Bio-Catalytic Structural Transformation of Anti-cancer Steroid, Drostanolone Enanthate with Cephalosporium aphidicola and Fusarium lini, and Cytotoxic Potential Evaluation of Its Metabolites against Certain Cancer Cell Lines
Source: Front Pharmacol. 2017 Dec 20;8:900. doi: 10.3389/fphar.2017.00900 (PMC5742531; doi:10.3389/fphar.2017.00900)
Supplement: Supplementary file 9 [file DataSheet9.PDF]

File Name	:	d:\mswin\data\11-d-4.mss
Creation Date/Time	:	16.06.16 at 21:50:16
File Type	:	Lo-Res Mass Data (Centroid)
File Source	:	Acquired on MASPEC system [msw/A091]
File Title	:	MAHWISH / DR. M. IQBAL
Operator	:	Barkat Ali
Instrument	:	MAT312---EI

SCAN GRAPH. Flagging=M/z.

Scan 6-3:05. Entries=124. 100% Int.=11772.

comp. 9

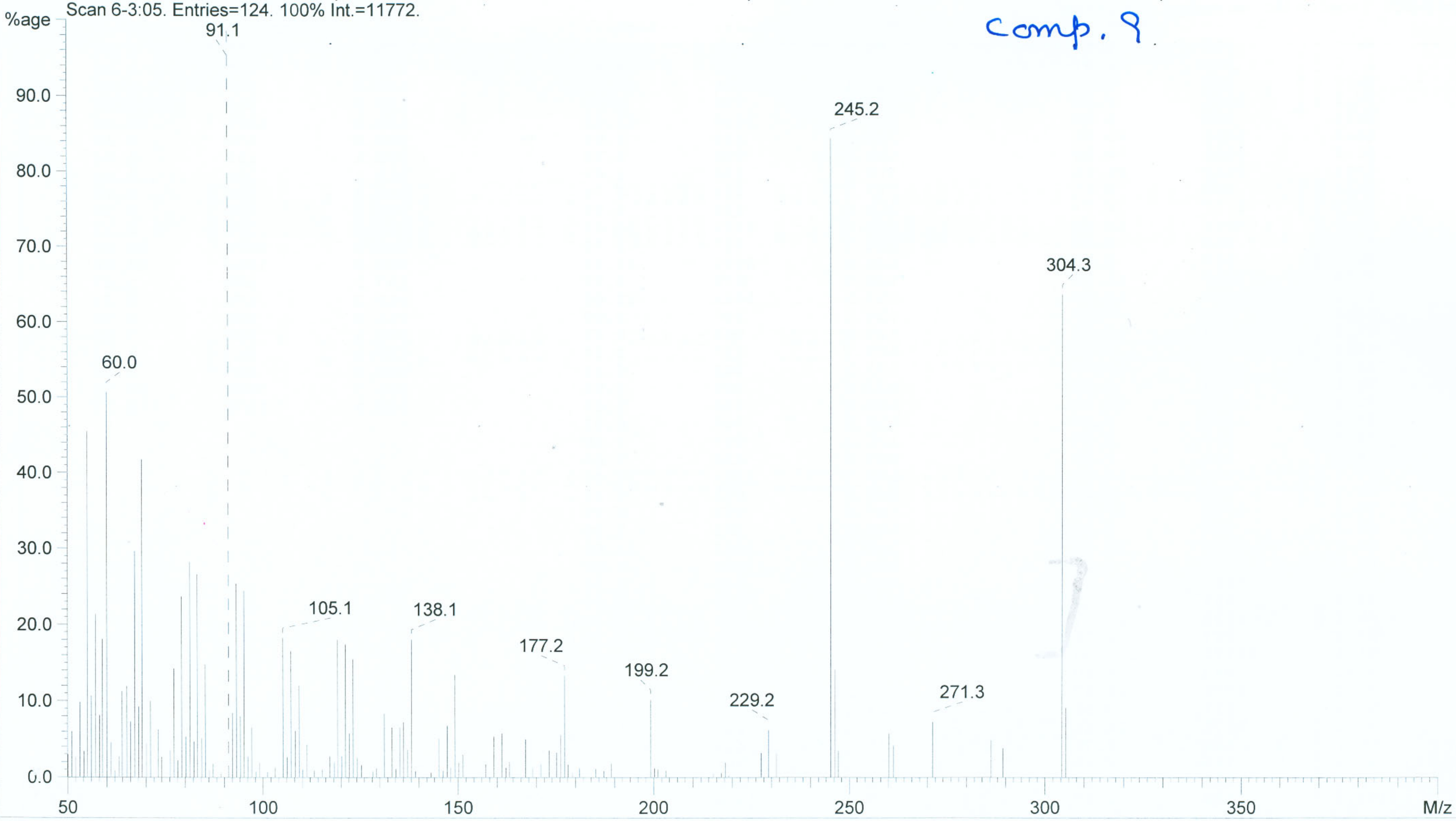

Comp-9

Mass	Relative Intensity	Theoretical Mass	Delta [ppm]	Delta [mmu]	RDB	Composition
197.1359	1.1	197.1330	14.3	2.8	7.5	$C_{15}H_{17}$
199.1505	14.0	199.1487	9.3	1.9	6.5	$C_{15}H_{19}$
200.1554	5.3	200.1565	-5.5	-1.1	6.0	$C_{15}H_{20}$
201.1649	6.0	201.1643	3.1	0.6	5.5	$C_{15}H_{21}$
202.1702	2.1	202.1722	-9.5	-1.9	5.0	$C_{15}H_{22}$
203.1439	3.7	203.1436	1.7	0.3	5.5	$C_{14}H_{19}O_{1}$
203.1781	1.9	203.1800	-9.2	-1.9	4.5	$C_{15}H_{23}$
204.1495	2.8	204.1514	-9.5	-1.9	5.0	$C_{14}H_{20}O_{1}$
205.1546	1.5	205.1592	-22.5	-4.6	4.5	$C_{14}H_{21}O_{1}$
205.1925	1.1	205.1956	-15.3	-3.1	3.5	$C_{15}H_{25}$
213.1640	1.7	213.1643	-1.6	-0.3	6.5	$C_{16}H_{21}$
215.1444	1.2	215.1436	3.8	0.8	6.5	$C_{15}H_{19}O_{1}$
215.1804	1.6	215.1800	1.9	0.4	5.5	$C_{16}H_{23}$
217.1595	4.4	217.1592	1.1	0.2	5.5	$C_{15}H_{21}O_{1}$
217.1965	2.2	217.1956	4.2	0.9	4.5	$C_{16}H_{25}$
218.1659	8.1	218.1671	-5.1	-1.1	5.0	$C_{15}H_{22}O_{1}$
219.1742	2.9	219.1749	-3.0	-0.7	4.5	$C_{15}H_{23}O_{1}$
220.1825	1.9	220.1827	-0.9	-0.2	4.0	$C_{15}H_{24}O_{1}$
227.1820	7.1	227.1800	8.8	2.0	6.5	$C_{17}H_{23}$
228.1858	1.6	228.1878	-8.6	-2.0	6.0	$C_{17}H_{24}$
229.1605	1.2	229.1592	5.6	1.3	6.5	$C_{16}H_{21}O_{1}$
229.1957	12.8	229.1956	0.4	0.1	5.5	$C_{17}H_{25}$
230.2007	2.7	230.2035	-11.9	-2.7	5.0	$C_{17}H_{26}$
231.1758	9.2	231.1749	3.9	0.9	5.5	$C_{16}H_{23}O_{1}$
232.1823	3.4	232.1827	-1.8	-0.4	5.0	$C_{16}H_{24}O_{1}$
233.1559	1.8	233.1542	7.6	1.8	5.5	$C_{15}H_{21}O_{2}$
233.1896	1.1	233.1905	-3.8	-0.9	4.5	$C_{16}H_{25}O_{1}$
242.2036	1.1	242.2035	0.6	0.1	6.0	$C_{18}H_{26}$
243.1752	1.6	243.1749	1.2	0.3	6.5	$C_{17}H_{23}O_{1}$
243.2108	1.7	243.2113	-1.8	-0.4	5.5	$C_{18}H_{27}$
244.1830	1.9	244.1827	1.3	0.3	6.0	$C_{17}H_{24}O_{1}$
245.1915	100.0	245.1905	4.1	1.0	5.5	$C_{17}H_{25}O_{1}$
246.1955	20.8	246.1984	-11.4	-2.8	5.0	$C_{17}H_{26}O_{1}$
247.2063	12.2	247.2062	0.4	0.1	4.5	$C_{17}H_{27}O_{1}$
248.2103	2.7	248.2140	-15.0	-3.7	4.0	$C_{17}H_{28}O_{1}$
253.1981	1.7	253.1956	9.8	2.5	7.5	$C_{19}H_{25}$
258.2026	1.6	258.1984	16.4	4.2	6.0	$C_{18}H_{26}O_{1}$
260.2198	15.6	260.2140	22.4	5.8	5.0	$C_{18}H_{28}O_{1}$
261.2269	9.2	261.2218	19.5	5.1	4.5	$C_{18}H_{29}O_{1}$
262.2297	1.7	262.2297	-0.0	-0.0	4.0	$C_{18}H_{30}O_{1}$
269.1867	2.5	269.1905	-14.4	-3.9	7.5	$C_{19}H_{25}O_{1}$
271.1917	10.4	271.1909	2.9	0.8	2.5	$C_{15}H_{27}O_{4}$
272.1876	2.4					
286.1918	7.9	286.1933	-5.2	-1.5	7.0	$C_{19}H_{26}O_{2}$
287.1663	3.1	287.1647	5.4	1.6	7.5	$C_{18}H_{23}O_{3}$
287.1908	2.5	287.1858	17.3	5.0	2.5	$C_{15}H_{27}O_{5}$
289.1947	7.6	289.1956	-3.4	-1.0	10.5	$C_{22}H_{25}$
		289.2015	-23.7	-6.8	1.5	$C_{15}H_{29}O_{5}$
290.2061	1.6	290.2035	9.1	2.6	10.0	$C_{22}H_{26}$
		290.2093	-11.1	-3.2	1.0	$C_{15}H_{30}O_{5}$
302.2271	1.9	302.2246	8.3	2.5	6.0	$C_{20}H_{30}O_{2}$
304.2403	49.7	304.2402	0.1	0.0	5.0	$C_{20}H_{32}O_{2}$
305.2431	10.9	305.2481	-16.3	-5.0	4.5	$C_{20}H_{33}O_{2}$
306.2465	1.4	306.2406	19.1	5.9	0.0	$C_{16}H_{34}O_{5}$

AVANCE AV-400 MHz
Lab # 115

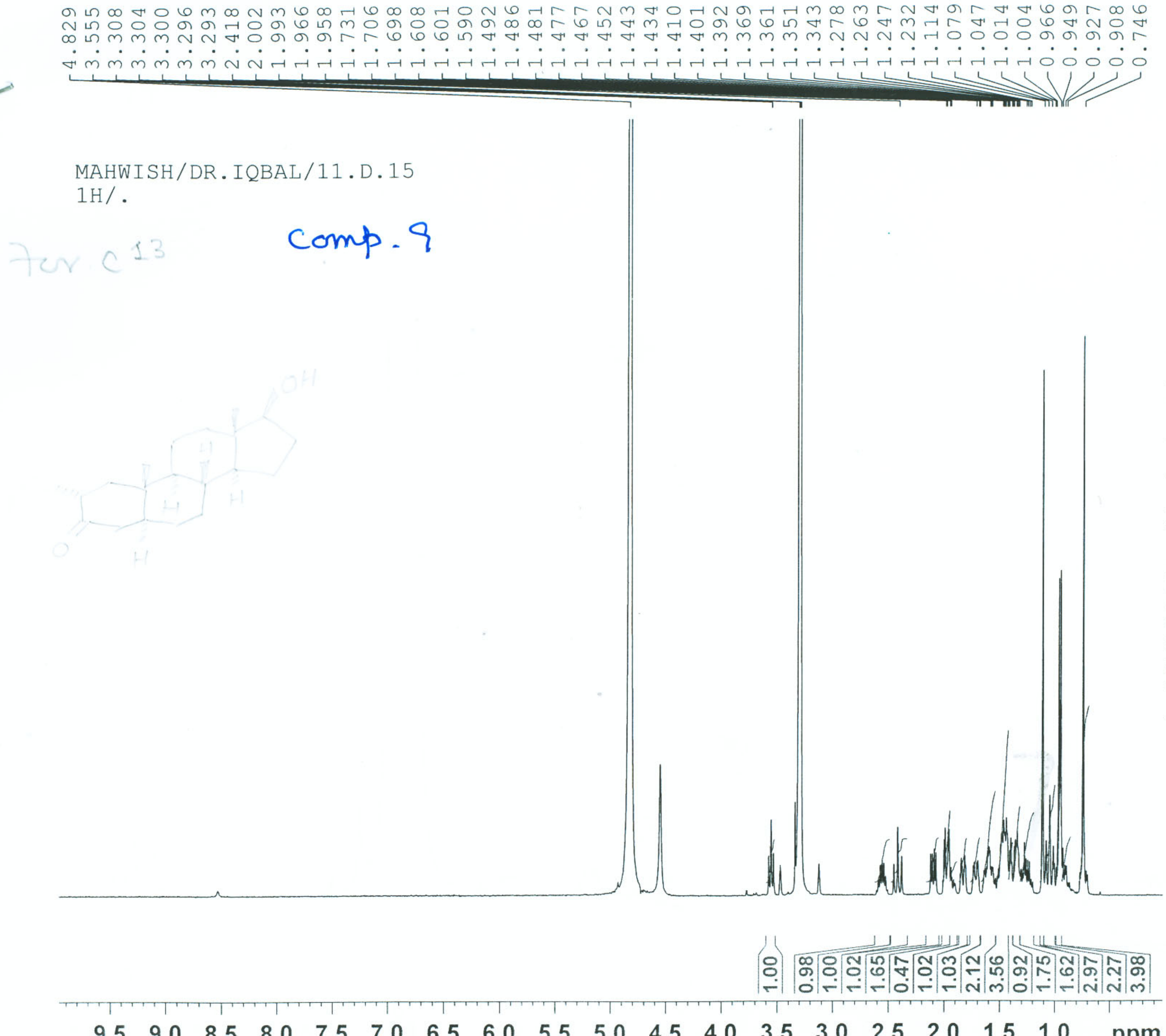

NAME	june06-16	
EXPNO	8	
PROCNO	1	
Date_	20160606	
Time	14.06	
INSTRUM	spect	
PROBHD	5 mm SEI 1H-13	
PULPROG	zg30	
TD	65536	
SOLVENT	MeOD	
NS	128	
DS	0	
SWH	8012.820	Hz
FIDRES	0.122266	Hz
AQ	4.0894966	sec
RG	256	
DW	62.400	usec
DE	6.50	usec
TE	300.0	K
D1	2.00000000	sec
TD0	1	

======== CHANNEL f1 ========

NUC1	1H	
P1	10.80	usec
PL1	3.00	dB
SFO1	400.0332002	MHz
SI	32768	
SF	400.0300087	MHz
WDW	EM	
SSB	0	
LB	0.30	Hz
GB	0	
PC	0.20	

MAHWISH / Dr. IQBAL / 11-D-15 / CD3OD
BB

Avance 500MHz Cryo-Probe

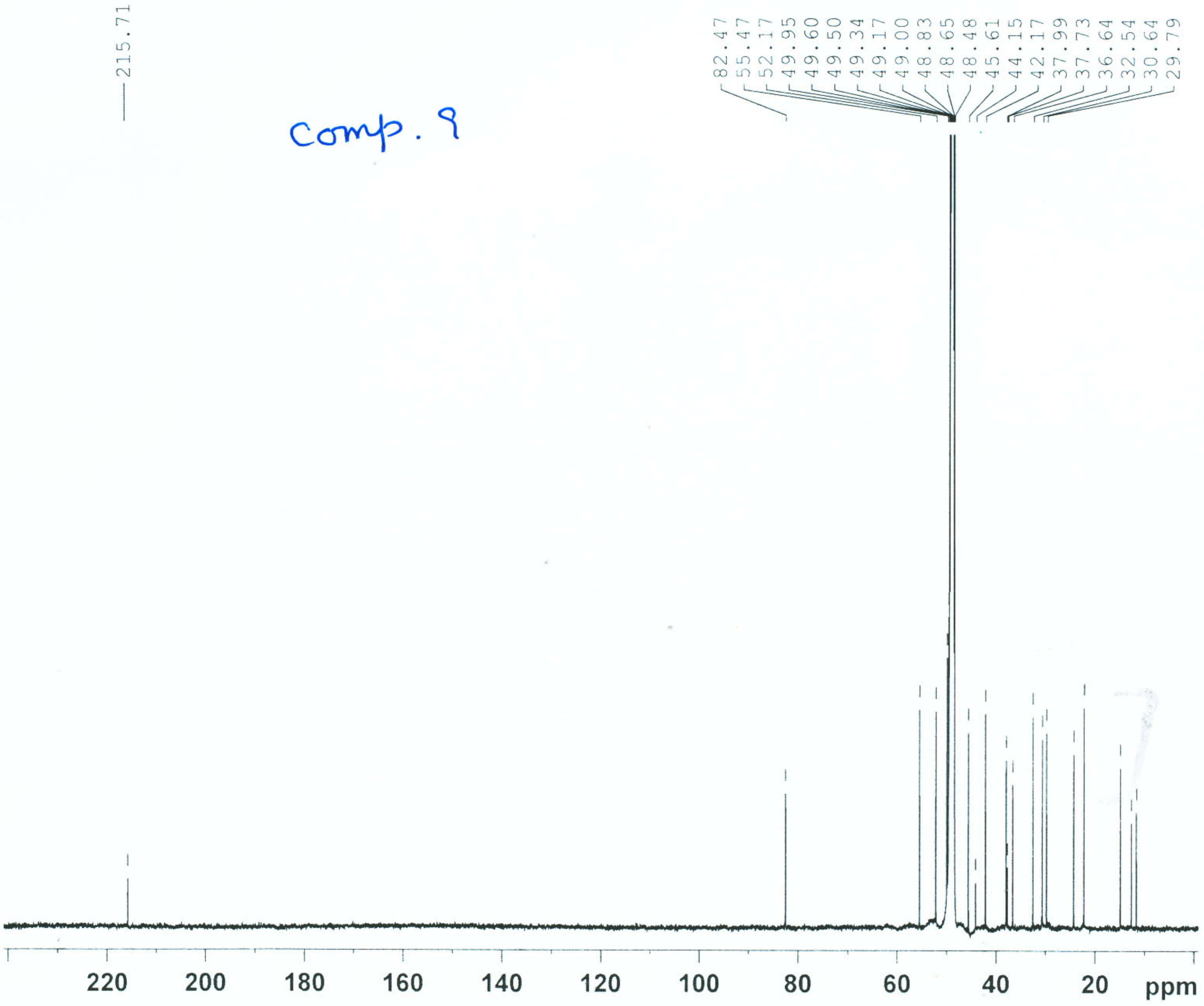

NAME	July01-16	
EXPNO	1	
PROCNO	1	
Date_	20160702	
Time	1.30	
INSTRUM	spect	
PROBHD	5 mm CPDUL 13C	
PULPROG	zgpg	
TD	32768	
SOLVENT	MeOD	
NS	16384	
DS	2	
SWH	31446.541	Hz
FIDRES	0.959672	Hz
AQ	0.5210771	sec
RG	32768	
DW	15.900	usec
DE	6.50	usec
TE	298.0	K
D1	1.50000000	sec
D11	0.03000000	sec
TD0	16	

======== CHANNEL f1 ========

NUC1	13C	
P1	10.30	usec
PL1	5.00	dB
PL1W	21.04969788	W
SFO1	125.8231760	MHz

======== CHANNEL f2 ========

CPDPRG2	waltz16	
NUC2	1H	
PCPD2	80.00	usec
PL2	-0.50	dB
PL12	13.11	dB
PL13	24.00	dB
PL2W	14.09191513	W
PL12W	0.61371964	W
PL13W	0.05000000	W
SFO2	500.3335023	MHz
SI	16384	
SF	125.8079011	MHz
WDW	EM	
SSB	0	
LB	1.50	Hz
GB	0	
PC	1.00	

MAHWISH / Dr. IQBAL / 11-D-15 / CD3OD
DEPT90

comp. 9

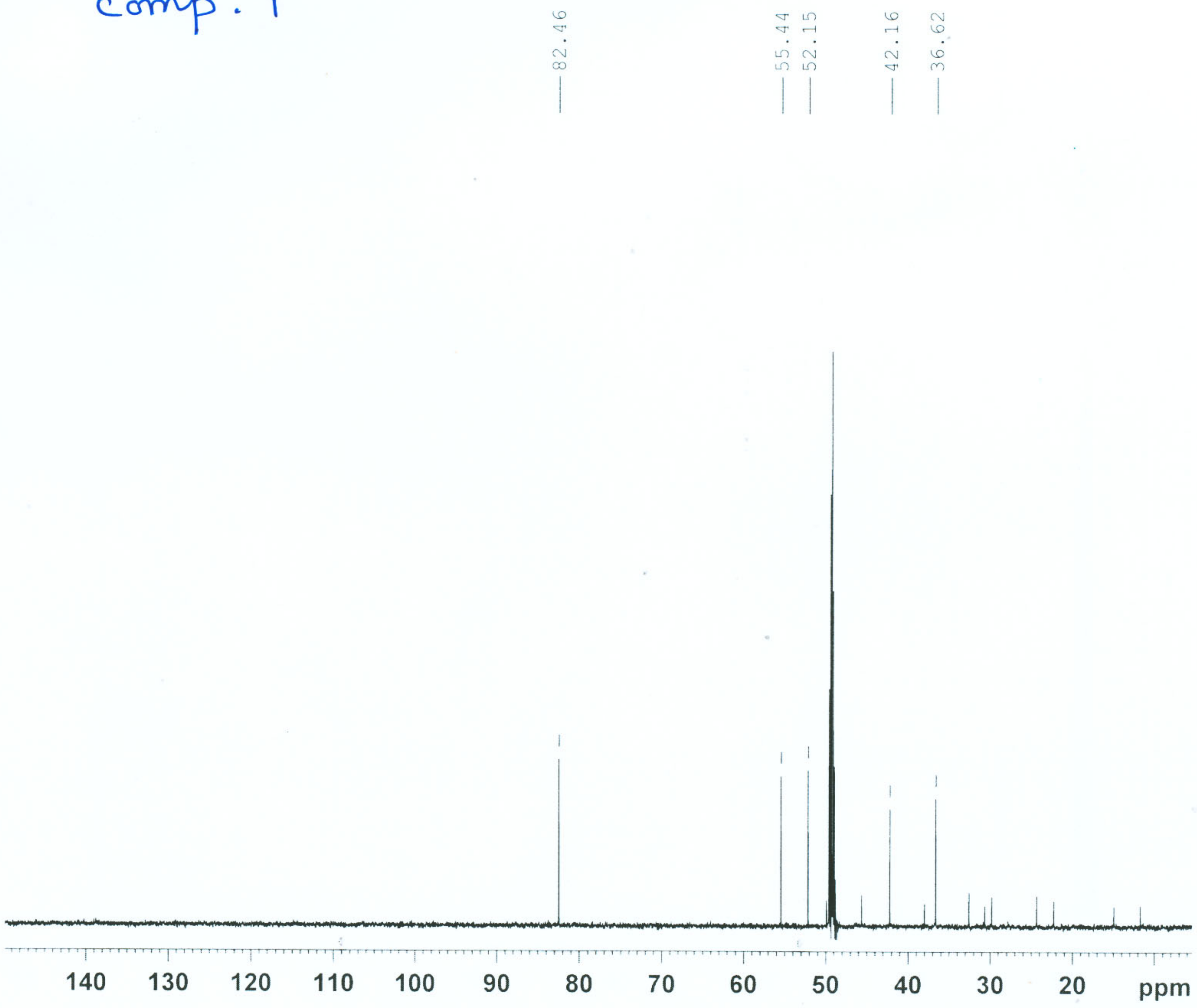

Avance 500MHz Cryo-Probe

NAME	July01-16
EXPNO	3
PROCNO	1
Date_	20160702
Time	16.02
INSTRUM	spect
PROBHD	5 mm CPDUL 13C
PULPROG	deptsp90
TD	32768
SOLVENT	MeOD
NS	4096
DS	2
SWH	25252.525 Hz
FIDRES	0.770646 Hz
AQ	0.6488762 sec
RG	32768
DW	19.800 usec
DE	6.50 usec
TE	290.8 K
CNST2	145.0000000
D1	1.50000000 sec
D2	0.00344828 sec
D12	0.00002000 sec
TD0	4

======== CHANNEL f1 ========

NUC1	13C
P1	10.30 usec
P12	2000.00 usec
PL0	120.00 dB
PL1	5.00 dB
PL0W	0.00000000 W
PL1W	21.04969788 W
SFO1	125.8206598 MHz
SP2	12.90 dB
SPNAM2	Crp60comp.4
SPOAL2	0.500
SPOFFS2	0.00 Hz

======== CHANNEL f2 ========

CPDPRG2	waltz16
NUC2	1H
P3	16.70 usec
P4	33.40 usec
PCPD2	80.00 usec
PL2	-0.50 dB
PL12	13.11 dB
PL2W	14.09191513 W
PL12W	0.61371964 W
SFO2	500.3330020 MHz
SI	16384
SF	125.8079011 MHz
WDW	EM
SSB	0
LB	1.00 Hz
GB	0
PC	1.00

Mahwish / Dr. Iqbal / 11-D-15
HSQC

Comp. 9

AVANCE AV-III HD
400 MHz
LAB #109A

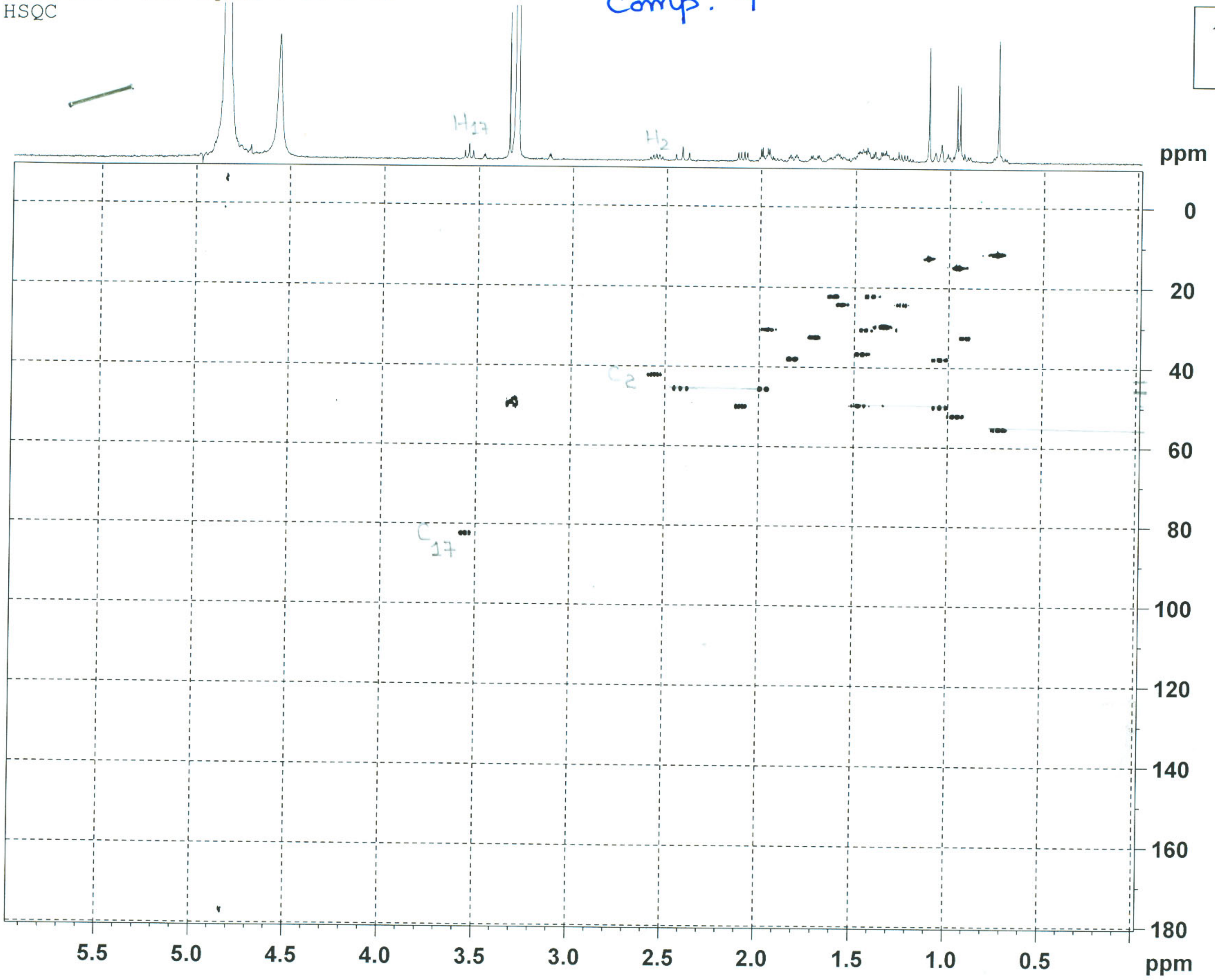

Current Data Parameters
NAME jun19-16
EXPNO 4
PROCNO 1

F2 - Acquisition Parameters
Date_ 20160619
Time 23.29
INSTRUM spect
PROBHD 5 mm SEI 1H/D-
PULPROG hsqcetgpsi
TD 2048
SOLVENT MeOD
NS 32
DS 16
SWH 2400.768 Hz
FIDRES 1.172250 Hz
AQ 0.4265301 sec
RG 202.75
DW 208.267 usec
DE 6.50 usec
TE 298.9 K
CNST2 145.0000000
D0 0.00000300 sec
D1 1.50000000 sec
D4 0.00172414 sec
D11 0.03000000 sec
D16 0.00020000 sec
D24 0.00089000 sec
IN0 0.00002610 sec
ZGOPTNS

======== CHANNEL f1 ========
SFO1 400.3312010 MHz
NUC1 1H
P1 6.60 usec
P2 13.20 usec
P28 1000.00 usec
PLW1 20.00000000 W

======== CHANNEL f2 ========
SFO2 100.6715147 MHz
NUC2 13C
CPDPRG[2 garp
P3 11.00 usec
P4 22.00 usec
PCPD2 84.00 usec
PLW2 150.00000000 W
PLW12 2.57229996 W

====== GRADIENT CHANNEL =====
GPNAM[1] SMSQ10.100
GPNAM[2] SMSQ10.100
GPZ1 80.00 %
GPZ2 20.10 %
P16 1000.00 usec

F1 - Acquisition parameters
TD 256
SFO1 100.6715 MHz
FIDRES 74.832375 Hz
SW 190.293 ppm
FnMODE Echo-Antiecho

F2 - Processing parameters
SI 1024
SF 400.3300117 MHz
WDW QSINE
SSB 2
LB 0 Hz
GB 0
PC 1.40

F1 - Processing parameters
SI 1024
MC2 echo-antiecho
SF 100.6629178 MHz
WDW QSINE
SSB 2
LB 0 Hz
GB 0

Mahwish / Dr. Iqbal / II-D-15
HMBC

Comp. 9

AVANCE AV-III HD
400 MHz
LAB #109A

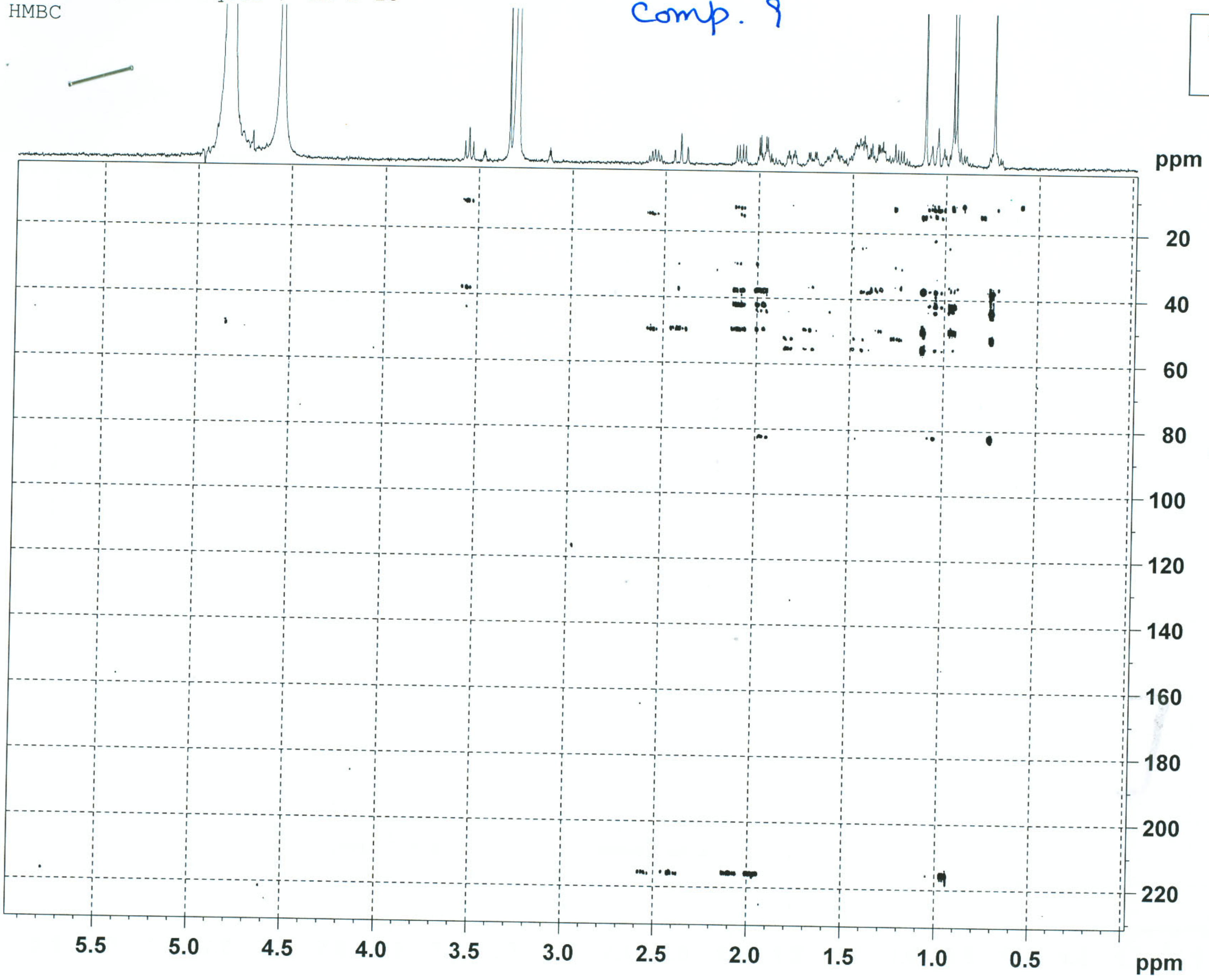

Current Data Parameters
NAME	jun19-16
EXPNO	5
PROCNO	1

F2 - Acquisition Parameters
Date_	20160620
Time	3.57
INSTRUM	spect
PROBHD	5 mm SEI 1H/D-
PULPROG	hmbcgplpndqf
TD	2048
SOLVENT	MeOD
NS	64
DS	16
SWH	2400.768 Hz
FIDRES	1.172250 Hz
AQ	0.4265301 sec
RG	202.75
DW	208.267 usec
DE	6.50 usec
TE	299.1 K
CNST2	145.0000000
CNST13	10.0000000
D0	0.00000300 sec
D1	2.00000000 sec
D2	0.00344828 sec
D6	0.05000000 sec
D16	0.00020000 sec
IN0	0.00002160 sec

======== CHANNEL f1 ========
SFO1	400.3312010 MHz
NUC1	1H
P1	6.70 usec
P2	13.40 usec
PLW1	20.00000000 W

======== CHANNEL f2 ========
SFO2	100.6746353 MHz
NUC2	13C
P3	11.00 usec
PLW2	150.00000000 W

====== GRADIENT CHANNEL =====
GPNAM[1]	SMSQ10.100
GPNAM[2]	SMSQ10.100
GPNAM[3]	SMSQ10.100
GPZ1	50.00 %
GPZ2	30.00 %
GPZ3	40.10 %
P16	1000.00 usec

F1 - Acquisition parameters
TD	256
SFO1	100.6746 MHz
FIDRES	90.422455 Hz
SW	229.930 ppm
FnMODE	QF

F2 - Processing parameters
SI	2048
SF	400.3300117 MHz
WDW	SINE
SSB	0
LB	0 Hz
GB	0
PC	1.40

F1 - Processing parameters
SI	1024
MC2	QF
SF	100.6629178 MHz
WDW	SINE
SSB	0
LB	0 Hz
GB	0

Mahwish / Dr. Iqbal / 11-D-15
COSY

Comp. 9

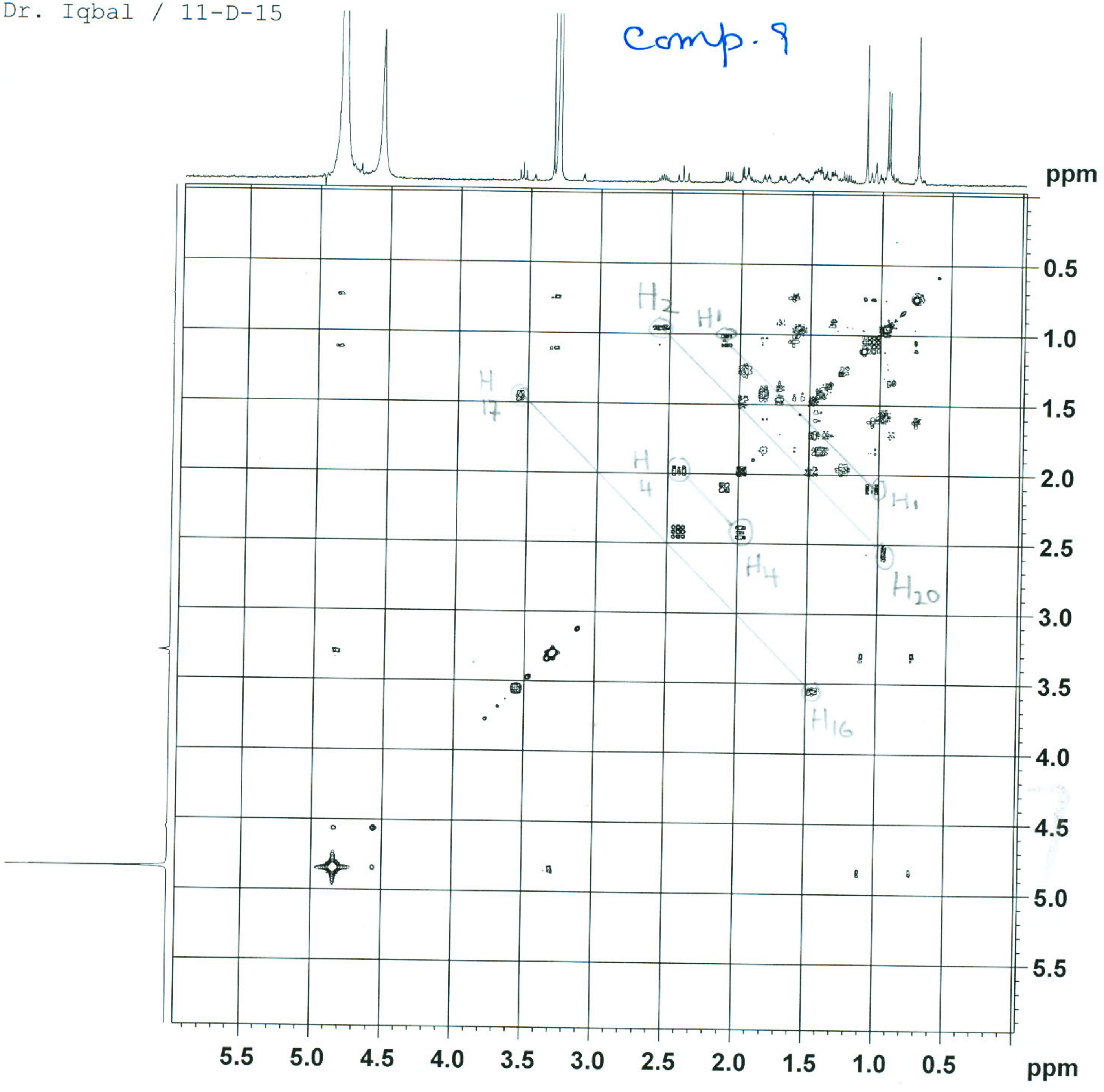

AVANCE AV-III HD
400 MHz
LAB #109A

```
Current Data Parameters
NAME              jun19-16
EXPNO                    2
PROCNO                   1

F2 - Acquisition Parameters
Date_             20160619
Time                 11.32
INSTRUM              spect
PROBHD   5 mm SEI 1H/D-
PULPROG           cosygpqf
TD                    2048
SOLVENT               MeOD
NS                      32
DS                       4
SWH             2400.768 Hz
FIDRES          1.172250 Hz
AQ             0.4265301 sec
RG                  202.75
DW               208.267 usec
DE                  6.50 usec
TE                 299.5 K
D0            0.00000300 sec
D1            2.00000000 sec
D13           0.00000400 sec
D16           0.00020000 sec
IN0           0.00041660 sec

======== CHANNEL f1 ========
SFO1         400.3312010 MHz
NUC1                    1H
P0                    6.70 usec
P1                    6.70 usec
PLW1           20.00000000 W

====== GRADIENT CHANNEL =====
GPNAM[1]        SMSQ10.100
GPZ1                 10.00 %
P16                1000.00 usec

F1 - Acquisition parameters
TD                     256
SFO1              400.3312 MHz
FIDRES            9.376500 Hz
SW                   5.996 ppm
FnMODE                  QF

F2 - Processing parameters
SI                    2048
SF             400.3300117 MHz
WDW                  QSINE
SSB        0
LB         0 Hz
GB         0
PC                    1.40

F1 - Processing parameters
SI                    2048
MC2                     QF
SF             400.3300117 MHz
WDW                  QSINE
SSB        0
LB         0 Hz
GB         0
```

Mahwish / Dr. Iqbal / 11-D-15
NOESY

Comp. 9

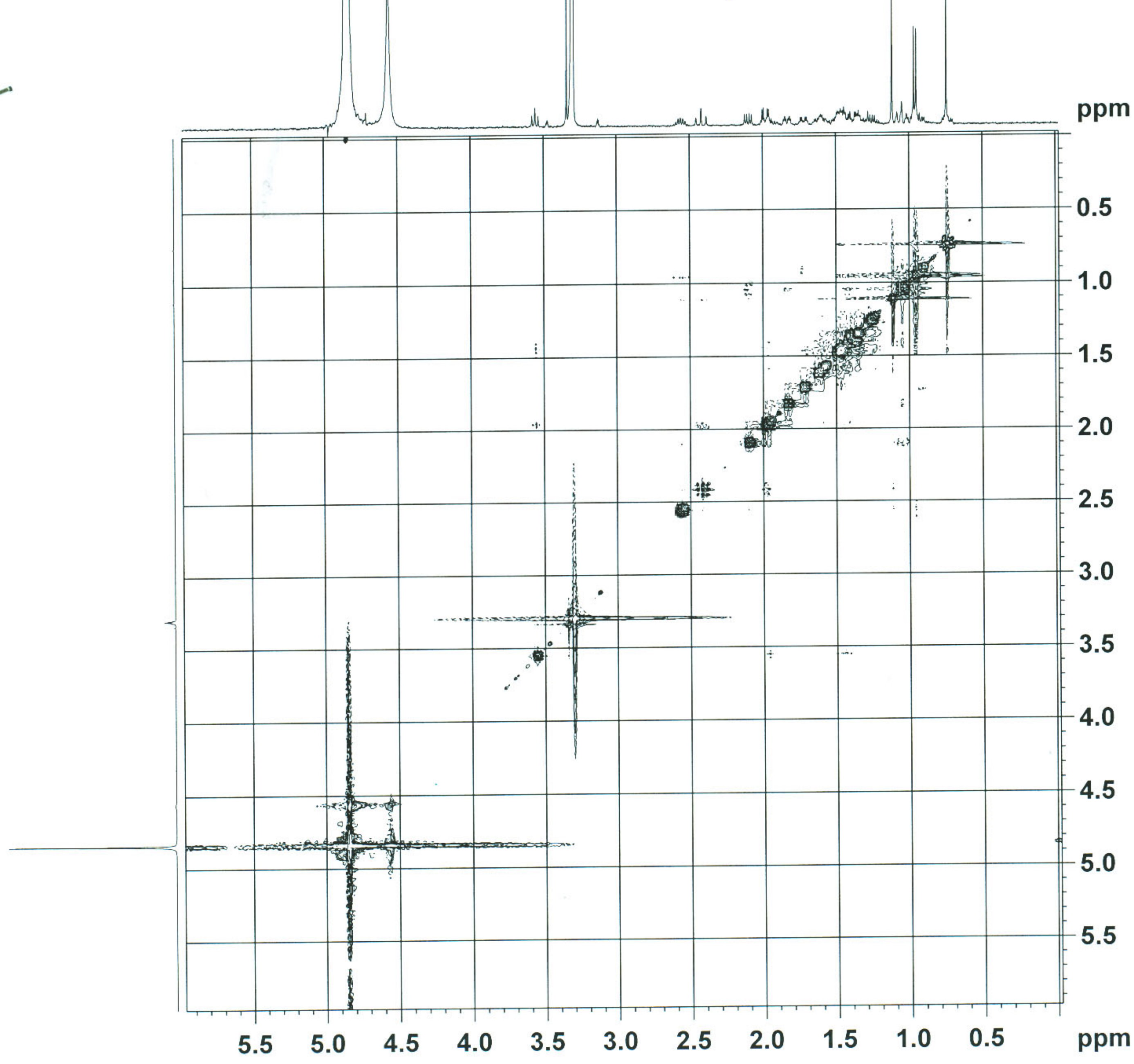

AVANCE AV-III HD
400 MHz
LAB #109A

Current Data Parameters
NAME jun19-16
EXPNO 3
PROCNO 1

F2 - Acquisition Parameters
Date_ 20160619
Time 17.12
INSTRUM spect
PROBHD 5 mm SEI 1H/D-
PULPROG noesygpph
TD 2048
SOLVENT MeOD
NS 32
DS 8
SWH 2400.768 Hz
FIDRES 1.172250 Hz
AQ 0.4265301 sec
RG 202.75
DW 208.267 usec
DE 6.50 usec
TE 299.1 K
D0 0.00019977 sec
D1 2.00000000 sec
D8 0.30000001 sec
D16 0.00020000 sec
IN0 0.00041660 sec

======== CHANNEL f1 ========
SFO1 400.3312010 MHz
NUC1 1H
P1 6.70 usec
P2 13.40 usec
PLW1 20.00000000 W

====== GRADIENT CHANNEL =====
GPNAM[1] SMSQ10.100
GPZ1 40.00 %
P16 1000.00 usec

F1 - Acquisition parameters
TD 256
SFO1 400.3312 MHz
FIDRES 9.376500 Hz
SW 5.996 ppm
FnMODE States-TPPI

F2 - Processing parameters
SI 1024
SF 400.3300117 MHz
WDW QSINE
SSB 2
LB 0 Hz
GB 0
PC 1.40

F1 - Processing parameters
SI 1024
MC2 States-TPPI
SF 400.3300117 MHz
WDW QSINE
SSB 2
LB 0 Hz
GB 0
